# Simultaneous Transcriptional Profiling of Bacteria and Their Host Cells

**DOI:** 10.1371/journal.pone.0080597

**Published:** 2013-12-04

**Authors:** Michael S. Humphrys, Todd Creasy, Yezhou Sun, Amol C. Shetty, Marcus C. Chibucos, Elliott F. Drabek, Claire M. Fraser, Umar Farooq, Naomi Sengamalay, Sandy Ott, Huizhong Shou, Patrik M. Bavoil, Anup Mahurkar, Garry S. A. Myers

**Affiliations:** 1 Institute for Genome Sciences, University of Maryland School of Medicine, Baltimore, Maryland, United States of America; 2 Department of Microbiology & Immunology, University of Maryland School of Medicine, Baltimore, Maryland, United States of America; 3 Department of Microbial Pathogenesis, University of Maryland Dental School, Baltimore, Maryland, United States of America; Midwestern University, United States of America

## Abstract

We developed an RNA-Seq-based method to simultaneously capture prokaryotic and eukaryotic expression profiles of cells infected with intracellular bacteria. As proof of principle, this method was applied to *Chlamydia trachomatis*-infected epithelial cell monolayers *in vitro*, successfully obtaining transcriptomes of both *C. trachomatis* and the host cells at 1 and 24 hours post-infection. Chlamydiae are obligate intracellular bacterial pathogens that cause a range of mammalian diseases. In humans chlamydiae are responsible for the most common sexually transmitted bacterial infections and trachoma (infectious blindness). Disease arises by adverse host inflammatory reactions that induce tissue damage & scarring. However, little is known about the mechanisms underlying these outcomes. *Chlamydia* are genetically intractable as replication outside of the host cell is not yet possible and there are no practical tools for routine genetic manipulation, making genome-scale approaches critical. The early timeframe of infection is poorly understood and the host transcriptional response to chlamydial infection is not well defined. Our simultaneous RNA-Seq method was applied to a simplified *in vitro* model of chlamydial infection. We discovered a possible chlamydial strategy for early iron acquisition, putative immune dampening effects of chlamydial infection on the host cell, and present a hypothesis for *Chlamydia*-induced fibrotic scarring through runaway positive feedback loops. In general, simultaneous RNA-Seq helps to reveal the complex interplay between invading bacterial pathogens and their host mammalian cells and is immediately applicable to any bacteria/host cell interaction.

## Introduction

Bacterial pathogens subvert host eukaryotic cellular pathways for survival and replication; in turn, infected host cells respond to the invading pathogen through cascading changes in gene expression. Deciphering these complex temporal and spatial dynamics to identify novel bacterial virulence factors or host response pathways is crucial for improved diagnostics and therapeutics. Microarrays have been the predominant methodology for determining gene expression profiles [1], revealing a diversity of bacterial pathogenic mechanisms [2] and commonalities of the complex global host response to infection [3]. However, microarrays are inadequate for profiling both prokaryotic and eukaryotic RNA from infected cells, as they are limited to what can be printed and detected on the array. Technical limitations such as high background signals and cross-hybridization also limits their dynamic range [4]. Consequently, array analyses of host-pathogen interactions have typically examined either the pathogen or the host, but usually not both simultaneously.

The few studies that examine both bacterial and host cell transcriptional responses separate the prokaryotic and eukaryotic messenger RNA (mRNA) prior to microarray profiling (for example [5]–[7]). Sufficient prokaryotic mRNA for hybridization can be difficult to obtain unless axenic culture or selective amplification [8] is used or, in the case of intracellular bacteria, *in vitro* infections are established with high multiplicities of infection (MOI). High MOIs may not represent natural infection levels, distorting expression profiles. The early events following invasion are often poorly characterized, as the small number of organisms yields insufficient transcripts for microarray detection. Furthermore, standard microarrays are restricted to existing genome annotation [1] and cannot detect novel RNA moieties that are not printed on the array. Tiling arrays overcome this limitation and have been successfully applied to bacteria, revealing antisense RNA expression and other non-coding RNA (ncRNA) transcripts [9]–[13]. However, the large size of eukaryotic genomes makes tiling arrays [14] prohibitively expensive for host gene expression studies. Tag-based sequencing methods [15] alleviate these problems to some extent, allowing individual transcripts to be digitally counted with a broad dynamic range. Nevertheless, as these approaches only sample a small region of a transcript, they cannot capture the full diversity of RNA classes and isoforms.

RNA-Seq, or deep sequencing of cDNA libraries by next-generation sequencing, circumvents many of the problems associated with microarray profiling or tag-based sequencing. RNA-Seq can comprehensively and systematically define the transcriptome of an organism with minimal bias [1], [16]–[18], across different experimental conditions or cell types [17], [18] without probe design or cross-hybridization problems. RNA-Seq data are consistent with microarray results [19]–[24] but are more sensitive, with essentially an infinite dynamic range. RNA-Seq is annotation-independent [18], allowing novel transcript discovery without being reliant on array design or preexisting annotation. Unlike tag sequencing, RNA-Seq can distinguish different mRNA isoforms and ncRNA, and can identify splice junctions and transcript boundaries [25], [26].

Despite these advantages, RNA-Seq profiling of both prokaryotic and eukaryotic gene expression from bacteria-infected cells is technically challenging. Total RNA extracted from infected cells is a heterogeneous mixture of many host and bacterial RNA moieties. Ribosomal RNA (rRNA) is the most abundant, representing up to 98% of total RNA [27]; however several RNA classes are now recognized, encompassing diverse sizes with many functions that remain to be elucidated [28]. Bacterial mRNA is typically a minor fraction of an infected cell, even under optimized *in vitro* conditions, and especially in early infection periods where bacterial numbers can be low. In contrast to eukaryotic mRNA, prokaryotic mRNA are often polycistronic and typically lack a polyadenylated tail, which precludes hybridization capture, cDNA synthesis or amplification using poly(T) oligomers. Thus, any analysis strategy that examines the polyadenylated eukaryotic fraction alone will not recover the full diversity of RNA in an infected cell, missing bacterial mRNA, bacterial ncRNA and eukaryotic ncRNA.

Members of the genus *Chlamydia* are obligate intracellular bacteria that cause the most common human sexually transmitted bacterial infections and a range of mammalian diseases with inflammatory etiologies. Infection is frequently asymptomatic and is an outcome of a complex dialogue between the host and *Chlamydia* [29]. In humans, disease sequelae results from long-term infections or re-infections that induce tissue damage and scarring [30].

*Chlamydia* has a unique biphasic developmental cycle that alternates between distinct forms. The infectious elementary body (EB) enters the host cell and sequesters within a modified membrane-bound inclusion where it decondenses into the non-infectious replicating form, the reticulate body (RB). From within this unique compartment, chlamydiae exploit the host cell by hijacking host organelles and metabolites [29]. Following replication, RBs differentiate back into infectious EBs, which are dispersed following cell lysis. Interconversion occurs asynchronously; by later infection times, chlamydial inclusions contain a variety of EBs, RBs and intermediate forms at various developmental stages. A reversible stress-response state, characterized by morphologically aberrant, non-infectious forms, can be induced *in vitro* by addition of stressors such as cytokines, antibiotics or by nutrient restriction (reviewed by [31]).

*Chlamydia* remains intractable to classic genetic manipulation, as replication outside of a mammalian cell is not yet possible and, despite advances in chlamydial transformation [32]–[34], routine genetic manipulation has not yet been achieved. With these limitations, genomic-scale approaches have been invaluable (reviewed by [35]). A series of elegant microarray profiling experiments outlined the chlamydial transcriptional landscape over the course of *in vitro* infection [36]–[38], and in response to various perturbations [37], [39]–[42]. These analyses show substantial chlamydial gene expression by 6–8 hpi, continuing through to a maximum by 24 hpi [36], [38] when most genes are expressed [36], [38].

The critical early (1 to 3 hpi) and immediate-early (<1 hpi) periods of *C. trachomatis* infection have not been comprehensively characterized by these high throughput approaches, with only one study examining the 1 hpi period [36]. As only a small number of infecting organisms are present, the limitations inherent to microarrays prevent accurate early transcript detection. Belland *et* *al* [36] found twenty-nine *C. trachomatis* D genes detectably expressed at 1 hpi in HeLa 229 cells, but only by using an MOI of 100. RNA-Seq on purified EBs and RBs of *C. trachomatis* [43] and *C. pneumoniae* [44] allowed transcriptional start site mapping and the identification of novel chlamydial ncRNAs at mid- and late periods in the developmental cycle, but not earlier than 24 hpi.

The host cell transcriptional response to *Chlamydia* infection has been studied using a variety of host cell types, patient tissues, chlamydial strains and times post infection using arrayed subsets of human genes [45]–[54]. The varying genes and the different methodologies and strains used limits their comparative utility. Generally, up-regulation of host genes involved in cytokine expression, inflammation, signal transduction and innate immunity, and down-regulation of genes involved in metabolism and cell cycle regulation was observed. The earliest time of *C. trachomatis* infection was 2 hpi [49], [54], with few differentially expressed host genes (<20); the early host response to *C. trachomatis* infection was subsequently described as “quiescent” [54]. RNA-Seq has not been applied to *Chlamydia*-infected host cells.

In this study, we employed simultaneous depletion of both *Chlamydia* and human rRNA by affinity-based counter selection [55] to enrich prokaryotic and eukaryotic RNA from infected cells. Deep sequencing of these enriched fractions captured both chlamydial and human transcriptomes from infected cells at the immediate-early and mid- periods of *in vitro* infection, providing proof-of-principle of the simultaneous RNA-Seq approach. In addition, this validated data provides novel insights into chlamydial biology and the host epithelial cell response *in vitro*.

## Results and Discussion

**Figure 1:**
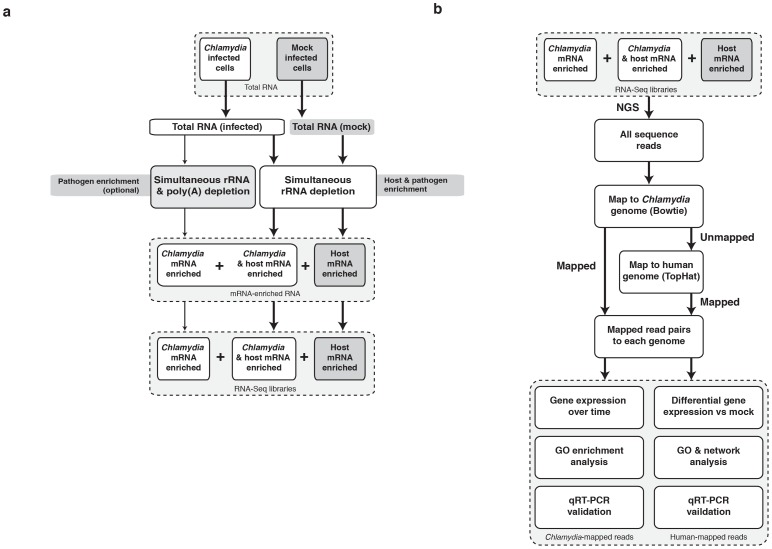
The simultaneous RNA-Seq pipeline. (a) Laboratory pipeline for simultaneous depletion of rRNA from prokaryotic and eukaryotic RNA mixtures. The enriched mRNA is used to create RNA-Seq libraries. (b) Bioinformatics pipeline for sequential mapping and analysis of simultaneous RNA-Seq data.

**Figure 2:**
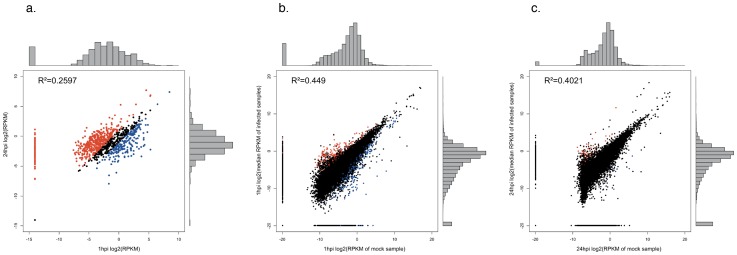
Distribution of RNA-Seq data. (a) *Chlamydia* 1 hpi versus 24 hpi. Chlamydial genes above the cutoff of RPKM≥0.1 and a minimum of 10 mapped reads are highlighted in blue and red at 1 and 24 hpi respectively; and host cells at (b) 1hpi relative to mock and (c) 24 hpi relative to mock. Significantly differentially expressed host cell transcripts (FDR≤0.05 and LFC≥2.0) between the mock and infected conditions are plotted in red (up-regulated) and blue (down-regulated). Pearson's correlation (R^2^) between replicates is indicated for each.

We synchronously infected HEp-2 cell monolayers with *C. trachomatis* serovar E EBs (MOI∼1). Replicate infections and mock-infected controls were established. Infections and controls were harvested at 1 and 24 hours post infection (hpi), encompassing infection, (1 hpi), differentiation and replication (24 hpi). Total RNA was extracted and split into two fractions. Both were subjected to simultaneous rRNA depletion; one fraction was optionally subjected to poly(A) subtraction to further increase the yield of bacterial mRNA (**Figure 1**). Depleted fractions were combined and RNA-Seq libraries constructed. Reads from deep sequencing were mapped to the human genome (release hg19) and the *C. trachomatis* serovar E genome (**Figure 1**), yielding ∼1.1 billion uniquely mapped Illumina HiSeq2000 sequence reads (**Table 1**). Reciprocal mapping demonstrated that no reads mapped to the other genome. Normalized RPKM (reads per kilobase per million mapped reads) [17] values were determined (**Table S1**) and the distribution plotted (**Figure 2**). To validate RNA-Seq expression, we examined fifteen immediate-early *Chlamydia* genes with a range of RPKMs by quantitative reverse transcriptase PCR (qRT-PCR). A strong correlation was found between normalized sequence coverage depth and qRT-PCR transcript abundance at 1 hpi (R^2^ = 0.89; **Figure S2**), demonstrating that even with low numbers of infecting organisms, RNA-Seq on the infected cell detects real immediate-early chlamydial gene expression.

**Table 1:** Summary of chlamydial and human unique mapped reads at (a) 1 hpi and (b) 24 hpi.

(a)	Mapped Reads				
Condition (1 hpi)	*Chlamydia*	(%)	Human	(%)	Total Unique Mapped Reads
Mock-infected	0	0.00	88,361,892	100.00	88,361,892
*Chlamydia*-infected replicate 1	11,436	0.01	80,639,860	99.99	80,651,296
*Chlamydia*-infected replicate 2	36,602	0.02	166,631,650	99.98	166,668,252
*Chlamydia*-infected replicate 3	83,854	0.02	352,554,875	99.98	352,638,729
*Chlamydia*-infected replicate 4	nd		114,708,905	99.98	114,709,004.98
**Total (infected)**	**131,892**	**0.02**	**714,535,284**	**99.98**	**714,667,176**
**Total (mock + infected)**	**131,892**		**802,897,716**		**803,029,608**

(b)					
Condition (24 hpi)	*Chlamydia*	(%)	Human	(%)	Total Unique Mapped Reads
Mock-infected	0	0.00	102,957,300		102,957,300
*Chlamydia*-infected replicate 1	3,343,180	24.72	10,180,999	75.28	13,524,179
*Chlamydia*-infected replicate 2	7,887,664	27.14	21,175,585	72.86	29,063,249
*Chlamydia*-infected replicate 3	7,297,404	32.31	15,290,960	67.69	22,588,364
*Chlamydia*-infected replicate 4	nd		143,854,131	100	143,854,131
**Total (infected)**	**18,528,248**	**28.4**	**190,501,675**	**71.6**	**208,875,075**
**Total (mock + infected)**	**18,528,248**		**483,960,650**		**520,862,298**
**Total (1 hpi +24 hpi)**	**18,660,140**		**1,096,356,691**		**1,114,861,983**

### Simultaneous transcriptional profiling by RNA-Seq

The technical potential of performing RNA-Seq on bacterial pathogens and their host cells simultaneously, as realized here, was recently assessed in a thought experiment [56]. One million non-rRNA bacterial reads and 100 million non-rRNA host cell reads were estimated to be required, although no data was presented in support [56]. These estimates do not account for different pathogens, MOI or changes in pathogen numbers over the course of infection. With the exception of *Chlamydia* reads at 1 hpi (**Table 1**), we exceed these estimates for both *Chlamydia* and the host cell. Obtaining as many sequence reads as possible is obviously desirable, however a limiting number of available organisms per cell restricts sequence yields at 1 hpi.

In this study, limiting numbers of *Chlamydia* at 1 hpi arises primarily because replication has not commenced post-invasion and also because we use an MOI of 1. Natural chlamydial infections are likely to occur at a much lower MOI. However using an MOI of less than 1 in *in vitro* infections will lead to fewer infected cells; these uninfected cells will alter the expression profile obtained, which is a summation of transcripts over the total cells sampled. To alleviate this summation effect in future, we are applying our simultaneous RNA-Seq method to single cells infected with *Chlamydia*. Using higher MOIs to inflate the pathogen transcript count will also distort expression profiles and moves the already simplified *in vitro* infection model further away from natural infections. We chose an MOI of 1 to ensure the maximum numbers of cells were infected while minimizing pathogen transcript inflation. With these limitations in mind, we find that applying a stringent cutoff to *Chlamydia* sequence reads from synchronized infections yields substantial insight into early chlamydial transcription.

### Chlamydial transcription at 1hpi

We first examined chlamydial gene expression at the immediate-early infection time (1 hpi). At this point of infection prior to bacterial replication, we reasoned there would be few chlamydial transcripts. More sequencing was performed (relative to 24 hpi) to increase chlamydial transcript recovery. Poly(A) depletion on a portion of each sample was also employed (**Figure 1**). As expected, a low number of *Chlamydia*-specific transcripts were found at 1 hpi (total of 131,892 reads), representing a small percentage of total mapped reads (0.02%; **Table 1**).

*Chlamydia* at early stages of infection contain mRNA carried over from the previous developmental cycle [57]. These carryover transcripts may not result in early protein synthesis, and may degrade early in the developmental cycle [57]. To examine carryover versus new transcripts at 1 hpi, we selected 11 immediate-early genes identified by RNA-Seq, including known early and carryover genes and performed qRT-PCR over a course of infection, including the infecting EB seed population (**Figure S3**). As described by Belland (2003) [36], a decrease in copy number over time is assumed to indicate carry-over transcripts, while an increase indicates new expression. Seven genes showed a decrease in copy number at 1hpi, suggesting these are carryover genes. Four genes increase at 1hpi, and likely represent new transcription [36]. The known early and carry-over genes exhibit the expected pattern of increased and decreased transcription respectively (**Figure S3**). Thus, the set of immediate-early genes that we identify includes both new and carry-over transcripts.

Using a standard cutoff of RPKM≥0.1 and a minimum of 10 mapped sequence reads [18], we detect expression of 399±34 chlamydial genes at 1 hpi (**Table 2** **; Table S2**). This represents approximately 44% of genes in the chlamydial genome. Microarray studies detected only 29 genes (3.2% of chlamydial genes) at 1 hpi using a hundred-fold higher MOI [36], highlighting the sensitivity and dynamic range of RNA-Seq. 24/29 microarray-detected genes are found at this cutoff. We next examined highly expressed genes at 1 hpi. Highly expressed genes are defined by an RPKM≥1.0 and a minimum of 50 mapped reads (**Table 2** **; Table S2**). With this stringent cutoff, 153±12 chlamydial genes (17.0% of all chlamydial genes) are highly expressed at 1hpi, again contrasting with the previous finding of 29 genes by microarray [36]. 20/29 microarray-detected genes are found at this cutoff. The genes expressed at 1 hpi by microarray [36] but not found in our RNA-Seq data at either cutoff are hypothetical genes or have putative metabolic functions (**Table S3**). Their absence in our data may arise from chlamydial strain or host cell variation, or other experimental differences such as the high MOI (∼100) used in the original study [36]. We focus on the highly expressed subset from this point forward.

**Table 2:** Number of chlamydial genes expressed at 1 and 24 hpi, by cutoff and by replicate (R).

	Standard gene expression cutoff				“Highly expressed” gene cutoff			
	>0.1 RPKM (10 reads minimum)				>1 RPKM (50 reads minimum)			
	R1	R2	R3	Avg (std dev)	R1	R2	R3	Avg (std dev)
**1 hpi**	205*	365	432	399 (34)	37*	141	165	153 (12)
**24 hpi**	815	802	811	809 (5)	219	221	219	220 (1)

* Excluded (insufficient reads).

We find immediate-early expression of the chlamydial general secretory (Sec) pathway (*SecD*, *E*, *F*, *G* and *Y*). Fifty-one proteins that form the proteinaceous components of the 30S and 50S ribosomal subunits are highly transcribed at 1 hpi and 24 hpi (**Table S2**). This supplies the core components of the chlamydial ribosome, supporting previous observations of early chlamydial protein synthesis [58], [59]. Fifty-six highly expressed genes are found at 1 hpi alone (**Figure S1; Table S2**). Known early genes are within this subset, including the proposed master regulator *euo* [60], [61] and the secreted inclusion proteins *incD*, *E*, *F* and *G* [36], [62]. Many are novel at this immediate-early time. Twenty-five of the unique 1 hpi highly expressed genes are hypothetical genes (**Table S2**). Thirteen of these have not previously identified as immediate-early genes, representing uncharacterized biological functionality within this phase of infection. Gene Ontology (GO) analysis of the remaining non-hypothetical genes reveals primarily metabolic and catabolic functions including *biosynthetic process*, *transmembrane transport* and *carbohydrate metabolic process*, consistent with an auxotrophic bacterium establishing an infection within a host cell (**Table S4**).

Acquisition of host cell nutrients is critical for chlamydial survival; many of the up-regulated immediate-early genes are directly relevant to this need. These include *npt1* (CT065; ADP/ATP translocase) and *npt2* (CT495; nucleoside phosphate transporter), which enable parasitism of host energy and nucleotides [36], [63]. In addition, we identify numerous transferases, transporters, permeases, proteases and other factors putatively involved in the interconversion or translocation of host metabolites (**Table S2**), consistent with the GO term enrichment analysis.

Several chlamydial genes predicted to encode riboflavin biosynthetic enzymes (*ribBA*, *ribC* and *ribH*) are highly expressed at 1 hpi (**Table S2**). Riboflavin biosynthesis is linked to iron acquisition in several bacterial pathogens [64], [65], where riboflavin is an electron donor for the crucial reduction step of Fe^3+^ to Fe^2+^. Iron is essential for chlamydial growth (reviewed by [31]), but how iron is acquired from the host cell is unclear. Typical strategies of iron acquisition do not apply as siderophore biosynthetic enzymes or host iron-binding receptors are not present, although a chlamydial metal ATP-binding-cassette (ABC) permease system (*ytgABCD*) is implicated in iron transport and regulation [66], [67]. *ribBA* encodes an bifunctional enzyme with GTP cyclohydrolase and 3,4-dihydroxy-2-butanone-4-phosphate synthase (DHBP) synthase activities. These catalyze the initial rate limiting steps of the two riboflavin biosynthesis pathways [68], [69]. Bifunctional *ribBA* is found in *Helicobacter pylori*; the bifunctionality enables a rapid co-regulated riboflavin biosynthetic response to iron-induced stress [65]. Immediate-early expression of a bifunctional *ribBA* and other riboflavin biosynthetic enzymes may be part of a chlamydial strategy to rapidly obtain soluble iron from the host cell.

### Chlamydial transcription at 24 hpi

In contrast to the low number of *Chlamydia*-specific reads at 1 hpi, over 18 million reads were uniquely mapped at 24 hpi (**Table 1**). *Chlamydia* sequence reads at 24 hpi represent a higher proportion of total mapped reads (28.4% versus 0.02% at 1 hpi; **Table 1**). This is consistent with peak chlamydial gene expression in the *in vitro* developmental cycle and highlights why this timepoint has been well defined by previous microarray studies. Using RPKM≥0.1 and a minimum of 10 mapped reads, 809±5 of 898 genes were detectably expressed by 24 hpi, representing 90.2% of the genome (**Table 2** **; Table S2**). As noted by Belland *et* *al* [36], transcription of this number of chlamydial genes by this stage of the *in vitro* lifecycle highlights the degree of optimization of the reduced *Chlamydia* genome.

Using RPKM≥1.0 and a minimum of 50 mapped reads, 220±1 genes are highly expressed by 24 hpi (24.5%; **Table 2** **; Table S2**). 109 are also highly expressed at 1 hpi (**Figure S2; Table S3**). 112 highly expressed genes are found only at 24 hpi (**Fig S1; Table S2**), including 34 hypothetical genes with no known function. Together with genes expressed only at 1 hpi, this confirms the broad pattern of temporal gene expression observed by earlier microarray analyses [36]–[38]. Applying simultaneous RNA-Seq to more infection timepoints should improve our understanding of these temporal gene expression profiles. The later timepoints of *in vitro* chlamydial infection such as 24 hpi are better characterized, as the large amount of chlamydial transcripts at these times falls well within the limits of microarray analysis. Nevertheless, *ahpC* (CT603; thioredoxin peroxidase), *trxA* (CT539; thioredoxin) and a predicted ferredoxin (CT312), all with putative antioxidant properties, are expressed at 24 hpi but have not been previously described. Host cells quickly produce reactive oxygen species (ROS) on chlamydial infection [70]. Increased expression of these genes by 24 hpi may be an expedient chlamydial response to ROS bursts and oxidative stress.

### The effect of *in vitro* chlamydial infection on host cell transcription

Host cell transcription responding to infection was examined by differential expression (DE) analysis of unique mapped reads from infected HEp-2 cells and a time-matched mock-infected HEp-2 control (**Table 1**). Using a false discovery rate (FDR) cutoff of ≤0.05 and log fold change (LFC) cutoff of ≥2.0, we identify 622 DE host transcripts at 1 hpi and 87 at 24 hpi (**Table S5**). 82 genes are differentially expressed at both times, with 4 genes differentially expressed at 24 hpi alone (**Figure S1**), suggesting that the establishment of infection has a greater effect on host cell transcription. To validate RNA-Seq expression levels of host genes, we selected twenty-four host genes (twelve each at 1 hpi and 24 hpi) with a range of RPKM values. A strong correlation was found between RNA-Seq normalized sequence coverage depth and qRT-PCR transcript abundance at both 1 and 24 hpi (R^2^ = 0.61; **Figure S4**). Overrepresented Gene Ontology (GO) terms for all DE host genes with annotation reveals a wide variety of functions, including *inflammatory response*, *immune response* and *anti-apoptosis* (**Table S3**), consistent with *Chlamydia*-induced immunopathologic processes.

The early host cell response to infection is not “quiescent” [54]. Some of the host cell responses observed may be elicited by chlamydiae to promote survival and replication. However, the host response measured here by comparing mock-infected cell lysates to infected cell lysates, includes both specific reactions to *Chlamydia* and non-specific cellular reactions to a phagocytosed foreign body. The key events of chlamydial uptake and endosomal trafficking, amongst others, may not be discernable from non-specific cellular responses in this experimental design. RNA-Seq experiments using opsonized latex beads and UV-killed EBs are in progress and should permit differentiation of non-specific responses. With these caveats, we still observe a diverse and dramatic host transcriptional response to chlamydial infection that has not been previously described. This encompasses many cellular pathways and functions, including growth factors, altered intercellular junctions and adhesion, disruptions to Wnt and Notch signaling, extensive cytoskeletal remodeling, lipid trafficking, transcriptional regulation and non-coding RNA (**Text S1**).

Chlamydial disease is an adverse outcome of host inflammation (reviewed by [71]). Repeated stimulation, from either long-term infection or successive re-infections, leads to tissue damage and scarring. There is evidence that *Chlamydia* exploits immune and inflammatory pathways for survival [30], [72], [73]. Under the cellular paradigm of chlamydial pathogenesis [74], infected epithelial cells are the first responders to chlamydial infection, initiating and promoting the immune response [30], [72], [73], [75]. Subverting or dampening this response may contribute to the adverse consequences of infection. We identify numerous host cell transcriptional responses to infection, including putative modulation of innate responses through cytokines, chemokines, immune signaling molecules such as sphingosine 1-phosphate, semaphorins, damage-associated molecular patterns (DAMPs), and the inflammasome, all of which can be interpreted in the context of immune dampening (**Text S1**). We again note that these transcriptional responses are derived from a highly simplified *in vitro* model of chlamydial infection within epithelial cell monolayers. While this *in vitro* model is widely used in the study of chlamydial biology, natural infection is more complex and dynamic, with many different cell types in tissues and the immune system interacting with infected cells. Nevertheless, with this global perspective of the *in vitro* host cell transcriptional response to infection, we identify a subset of differentially expressed genes that may provide novel insight into chlamydial scarring (**Table S5**).

### Many extracellular matrix components are differentially expressed on chlamydial infection

The extracellular matrix (ECM) is the dynamic interdependent network of proteins, proteoglycans and glycoproteins that enmeshes epithelial cells and tissues [76]. The ECM has a major role in cellular adhesion, patterning and architecture, and “outside-in” signal transduction [76]. In our RNA-Seq data, many host ECM moieties are differentially expressed, supporting a dramatic and rapid remodeling of the extracellular milieu in response to chlamydial infection, including mucins (**Text S1**), metalloproteinases, numerous collagens and several fibrosis-associated moieties.

Epithelial cells, PMNs and other immune cells produce molecules that remodel the ECM, notably the zinc-dependent matrix metalloproteinases (MMPs) [77]–[79]. MMPs influence cell behavior by releasing growth factors and biologically active peptides from the ECM and by regulating inflammatory mediators [80]. The behavior of these molecules is linked to chlamydial scarring. MMP-9 (gelatinase B) is implicated in infected murine oviduct fibrosis [81]. Increased activity of *MMP-9* was found in human endothelial cells infected with *C. pneumoniae* [82] and in the conjunctiva of trachoma patients [46]. MMP-2 (gelatinase A) and MMP-9 were identified in infected human fallopian tube organ cultures [83]. MMP2 and MMP9 activity induce tenascin-C expression, which in turn induces further MMP expression, creating a positive feedback loop of MMP/tenascin-C activity that could contribute to chlamydial scarring (see below). We observe up-regulation of *MMP-2* at both 1 and 24 hpi. *MMP-9* expression is not detected and may be specifically secreted by PMNs or other immune cells rather than epithelial cells. Testican-1/SPOCK1 (sparc/osteonectin, cwcv and kazal-like domains proteoglycan 1), a highly conserved chimeric proteoglycan that regulates MMP-2, is also up-regulated at 1 hpi [84]. Conversely *MMP-28* (epilysin), expressed in normal tissues [85] and thought to participate in tissue homeostasis, is down-regulated in infected cells at 1hpi. Membrane-bound *MMP-24* is down-regulated at 1hpi.

In addition to these MMP expression patterns, we find differential expression of other proteinases that are previously unreported in the context of chlamydial infection. Six members of the ADAM (A Disintegrin And Metalloproteinase) and ADAMTS (A Disintegrin And Metalloproteinase with ThromboSpondin motifs) [86], [87] families of proteinases exhibit differential expression at both 1 and 24 hpi. The membrane-bound ADAM proteins activate zymogens such as TNF-α, and participate in cell adhesion via integrin interaction [86], [87]. ADAM proteins also participate in activation of the conserved Notch signaling pathways (see Text S1) [88]. ADAMTS are secreted proteins that modulate the ECM by cleavage of procollagen and proteoglycans [86]; these fragments may act as ligands for further inflammatory signaling. *ADAM33* and *ADAMTSL4* are down-regulated at 1 hpi, whereas *ADAM12*, *ADAM19*, *ADAMTS3* and *ADAMTS6* are up-regulated at both 1 and 24 hpi.

Collagens are a major component of ECM scaffolding, conferring tensile strength and viscoelasticity [89]; collagens also interact with integrins and other signaling receptors [90]. Immunohistochemical examination of conjunctiva from patients with active trachoma previously showed new (type V) and increased (type I, III and IV) collagen deposition [91]. Remarkably, eight members of the collagen superfamily are differentially expressed in our data, indicating that collagen deposition processes are initiated very early in infection rather than as a late consequence of disease progression. *COL3A1, COL4A1, COL4A2, COL5A1, COL5A3*, and *COL16A1* are all up-regulated at 1 hpi only; *COL25A1* is up-regulated at both 1 and 24hpi. *COL15A1* is down-regulated at 1 hpi. Collagens are subdivided based upon their supramolecular assemblies: *COL3A1*, *COL5A1* and *COL5A3* are fibril-forming; *COL4A1* and *COL4A2* are network-forming; *COL15A1* is a multiplexin, containing multiple triple-helix domains (collagenous domains) interrupted by non-collagenous domains; *COL16A1* is fibril-associated; and *COL25A1* is membrane-associated [89]. The type IV collagens, including *COL4A1* and *COL4A2*, are part of the basement membrane, an ECM layer that coats the basal aspect of epithelial cells [89].

Several other basement membrane components [92] are differentially expressed early in infection, notably subunits of the laminin heterotrimer (*LAMA4* and *LAMC3*), and nidogen (*NID1*) which crosslinks collagen IV and laminin. *LAMA4* and *NID1* are strongly up-regulated at 1hpi, whereas *LAMC3* is down-regulated. *LAMA4* is also strongly up-regulated at 24 hpi. Fibulin-5 (*FBLN5*) and hemicentin (*HMCN1*), both secreted glycoproteins that interact and crosslink with other members of the ECM [93] are strongly up-regulated. These dramatic early expression changes of numerous ECM moieties may underlie chlamydial disease outcomes through fibrotic scar formation.

### Hypothesis – mechanisms for chlamydia-induced fibrotic scarring

Long-term chlamydial infection (or re-infection) that is untreated or undetected will produce disease sequelae arising from collagenous scar formation on mucosal surfaces. For trachoma, scarring of the conjunctiva causes the eyelid to roll inwards by scar contraction (entropion). The eyelashes subsequently abrade the cornea (trichiasis), resulting in corneal opacity and blindness [94]. In genital infections of women, pelvic inflammatory disease and ascending infection precede scar formation in fallopian tubes, leading to tubal infertility, hydrosalpinx or ectopic pregnancy [30]. The molecular processes that lead to these adverse outcomes are largely unknown. Combined with the transcriptional changes in ECM components described above, we identify possible positive feedback mechanisms for chlamydiae-induced fibrotic scarring that center upon tenascin C, gremlin1 and TGF-β.

**Figure 3:**
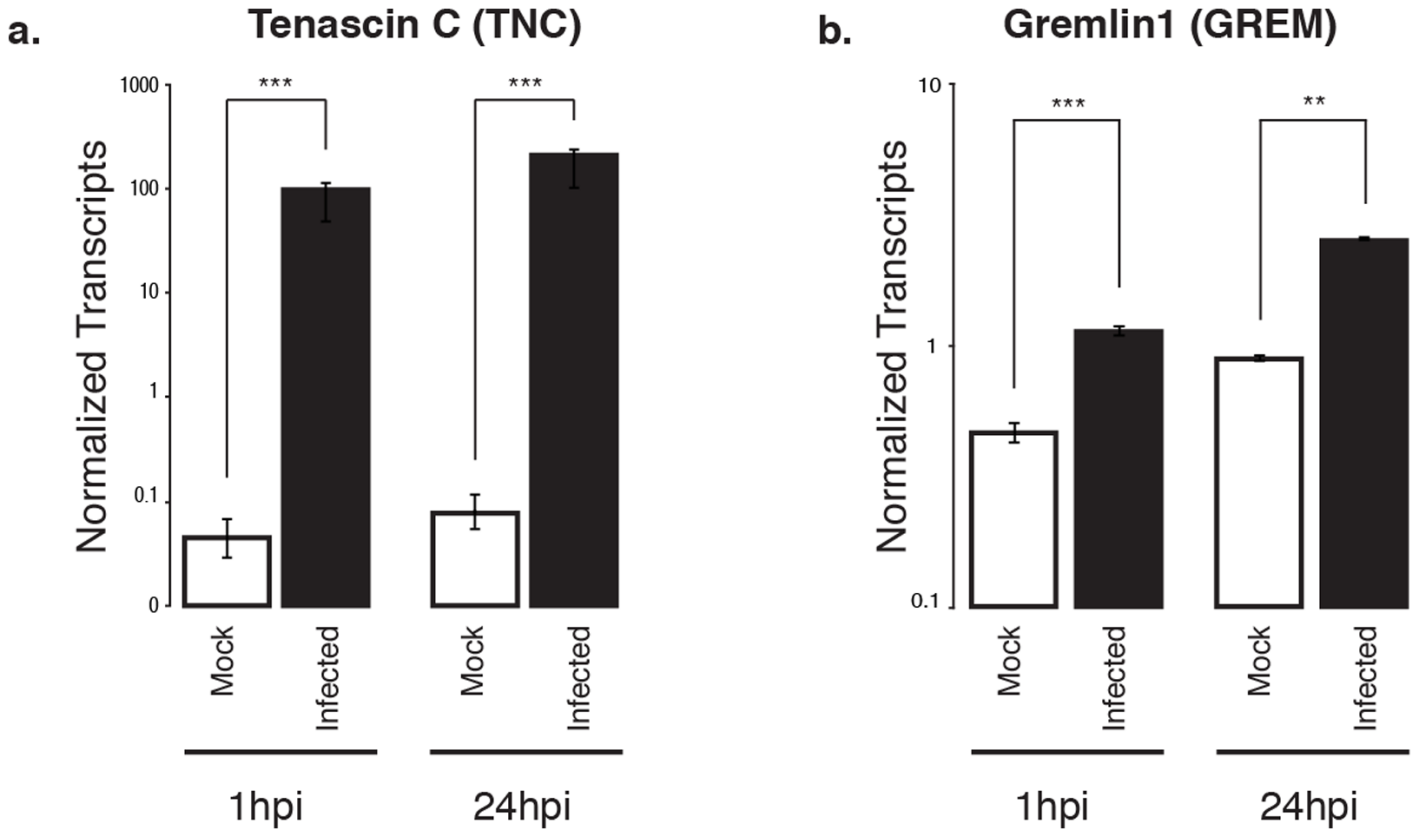
Confirmation of differential expression for selected pro-fibrotic genes over time. (a) Gremlin1 and (b) tenascin-C in *Chlamydia*-infected cells at 1 and 24 hpi, compared to mock-infected cells. Values are based on fold changes calculated from absolute quantitation of each gene of interest, normalized to human ATP synthase 6. Asterisks indicate statistically significant differences as calculated by Student's t test (***: p<0.0001; **: p<0.002). Error bars represent standard deviation over a minimum of 2 biological replicates.

Two members of the tenascin ECM glycoprotein family are notably differentially expressed upon chlamydial infection relative to mock-infected cells: tenascin-C (*TNC*) is up-regulated, whereas tenascin-X (*TNXB*) is down-regulated. Upregulation of *TNC* on chlamydial infection at both 1 and 24 hpi was confirmed by qRT-PCR (**Figure 3**). TNC is a pleiotropic protein with multiple binding domains, including EGF and fibronectin repeats that are subject to alternative splicing. It has numerous potential glycosylation sites, creating the potential for many isoforms [95], [96]. TNC has a hexameric protein organization that may enable extensive cross-linking. It promiscuously interacts with many ECM architectural molecules and receptors, and thus participates in both structural and signaling processes [95], [96].

TNC is not found in healthy tissues but is transiently expressed on cellular injury, and mediates global fibrotic processes as part of tissue repair [96]. After repair is complete, TNC expression normally decreases. However, abnormal persistent TNC expression is correlated with excessive matrix deposition that leads to collagenous scar formation in several fibrotic diseases [96] – this suggests an equivalent role in chlamydial scarring. Production of a tenascin protein has been previously observed by immunohistochemical studies of conjunctival biopsies taken from patients with trachomatous conjunctivitis [97]. Higher expression is also found in chronic cardiac conditions, and is a reliable indicator of poor patient prognosis [95], [96]. Abnormal TNC expression also drives matrix degradation in arthritic diseases, and fibrosis in response to infections, including lung damage from tuberculosis and HPV lesions in cervical epithelia [95], [96]. Moreover, TNC is associated with inflammatory processes, including TLR4 induction of pro-inflammatory cytokines, re-epithelialization, and tissue remodeling [95], [96], [98]. During acute inflammatory events, TNC expression is concentrated in regions of increased immune cell infiltration, and is particularly associated with PMN infiltration [96]. PMN recruitment is a well-known feature of the immune response to chlamydial infection [71].

*TNC* expression is linked to TGF-β-mediated fibrosis and induces *TGF-β* expression [95], [96], [99], [100]. A central role for *TGF-β* in chlamydial disease outcomes has been previously discussed (reviewed by [101]). Increased expression of *TNC* on chlamydial infection may create a positive feedback loop that ultimately results in increasing amounts of collagen and other ECM components being deposited. We find strongly increased expression of *TGF-β2* at 1 hpi; increased expression of multiple collagen family members was noted above. A central role for TGF-β-mediated fibrosis is further supported by potential microRNA-mediated alterations of TGF-β expression in trachoma patients [102]. Another positive feedback loop has been posited for the interaction between *TNC* and MMPs induced by inflammation (see above) [96]. Fragments of collagen and other ECM components produced by MMPs will further stimulate inflammation [80]. In addition, Wnt signaling pathways intersect with TGF-β-mediated fibrosis [103]; we observe differential expression of several components of Wnt signaling (**Text S1**).

Thus, we find transcriptional evidence of several paracrine responses to early chlamydial infection that intersect with TNC and TGF-β, and which may induce scarring through uncontrolled positive feedback loops. As an aside, persistent *TNC* expression has also been linked to atherosclerosis, where it contributes to both plaque formation and rupture [95], [98]. In this study, we used a genital serovar of *C. trachomatis*, however, *C. pneumoniae* is controversially linked to several chronic conditions with inflammatory etiologies, including atherosclerosis. Chlamydial dysregulation of *TNC* and other positive feedback loop participants may provide an insight into the correlation of *C. pneumoniae* with atherosclerosis and other multifactorial inflammatory diseases.

Building on this theme of dysregulated cellular processes contributing to fibrosis, at both 1 and 24 hpi we also observe strongly increased expression of gremlin (*GREM1*), an antagonist of bone morphogenetic protein (BMP) receptors [104]. Upregulation of *GREM1* at both 1 and 24 hpi was confirmed by qRT-PCR (**Figure 3**). BMPs are members of the TGF-β receptor superfamily and are key participants in tissue remodeling [105]. GREM1 is a cysteine knot-secreted protein that is implicated in the prevention of epithelial regeneration and participates in the epithelial-to-mesenchymal transition that converts epithelial cells to fibrotic myofibroblasts [104]. Transient overexpression of *GREM1* in rat lungs induces epithelial injury and reversible pulmonary fibrosis [106]. *GREM1* overexpression has been identified as a direct inducer of fibrosis in diseases with fibrotic etiologies, including asbestosis, pulmonary sarcoidosis, idiopathic pulmonary fibrosis, glomerulonephritis, cirrhosis, and hepatic fibrosis [104], [107]. In addition, *GREM1* overexpression will elicit TGF-β-induced fibrosis in the lung; in turn, TGF-β itself induces *GREM1* production [107]. This suggests another positive feedback loop, again centered on TGF-β and its ligands, that may influence chlamydial scarring sequelae.

In summary, validated RNA-Seq analyses of *Chlamydia*-infected epithelial cells demonstrate remarkable early increased expression of host genes directly associated with fibrosis and collagenous scarring. Combined with increased expression of fibril- and network-forming collagens and other ECM constituents, this is relevant to the long term scarring sequelae of chlamydial disease. From these findings, we hypothesize that dysregulated early persistent expression of at least *TNC*, *GREM1*, *TGF-β2* and various proteases in infected epithelial cells mediates *Chlamydia*-induced tissue damage.

**Figure 4:**
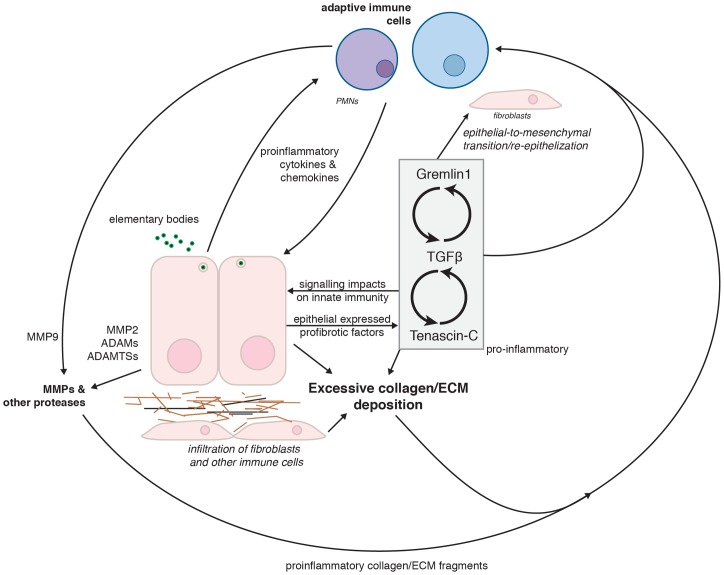
A proposed model of chlamydial-induced fibrosis and chronic scarring through the induction of multiple positive feedback loops. Infection of epithelial cells by *Chlamydia* leads to production of proinflammatory cytokines and chemokines that lead to recruitment and activation of immune cells. Recruited immune cells and infected epithelial cells secrete pro-fibrotic matrix metalloproteases (MMPs) that act upon the extracellular matrix (ECM), including collagens. The breakdown products of these proteases are also pro-inflammatory. Infected epithelial cells express the pro-fibrotic molecules TGF-β, Gremlin1 and Tenascin-C; expression of each amplifies the other, creating a series of nested positive feedback loops that increase the deposition of collagens and other ECM components, which in turn further induce immune cell recruitment and activation.

We further hypothesize that in susceptible individuals, these dysregulated genes establish a series of interlocked positive feedback loops that disrupt the homeostasis of multiple pathways, ultimately resulting in increased deposition of collagen and other ECM constituents (**Figure 4**). Ongoing inflammatory stimulation by lengthy infection or re-infection and recruitment of immune cells that participate in the fibrotic response, such as collagen-depositing fibroblasts, could exacerbate scarring through positive feedback loop reinforcement. As not all individuals develop scarring sequelae, host factors are likely to influence disease outcomes [30], [71]. We postulate that these susceptibility differences may be mediated by host genetic variation that influences the impact of these feedback loops by increased degree and/or period of aberrant expression, increasing collagen deposition rates and propensity for scarring. These hypotheses are currently being tested in both *in vitro* and *in vivo* systems that better capture the complexity of natural chlamydial infection.

## Conclusions

We developed and applied the simultaneous RNA-Seq method, using *Chlamydia*-infected cells as proof of principle. Despite a low MOI and substantial amounts of eukaryotic RNA, our method readily distinguishes chlamydial and host expression, yielding a detailed view of both host and pathogen transcription particularly in the poorly characterized early stages of infection. A substantial transcriptional program is rapidly initiated by *Chlamydia* following adherence and uptake, including carry-over transcripts from the infecting EBs and new transcription. In addition to the core components of the chlamydial ribosome, the Sec pathway and numerous novel hypothetical genes, we identify early expression of a bifunctional riboflavin biosynthetic enzyme that may mediate soluble iron acquisition from the host cell.

The epithelial host cell response to chlamydial infection *in vitro* is rapid and dramatic. A central paradox of chlamydial infection is that the immune response contributes to disease pathology. We find transcriptional evidence, within the constraints of a simplified model of infection, for attempted immune dampening through alterations in antimicrobial peptide and mucin expression, by mitigation of innate immunity and potential interference with signaling pathways, and by possible differential recruitment or repulsion of immune cell subsets. We identified and validated abnormal early transcription of host factors linked to scarring in numerous other fibrotic conditions. *Chlamydia*-induced aberrant expression of these factors may induce positive feedback loops that amplify tissue damage. Continuing reinforcement of these feedback loops may also provide an explanation for disease severity from long-term infection or re-infection.

Remarkably, transcription of these putative immune dampening and tissue damaging factors are evident as early as 1 hpi. Thus, depending on host factors influencing immune dampening or the severity of the fibrotic response, we speculate that the initial infection insult could be sufficient to commit a host into the responses that ultimately result in scarring. Subtle alterations of such a multidimensional equilibrium between the host cell and the invader may permit pathogen clearance on the one hand, or enable ongoing cryptic infection (or reinfection) with the resulting scarring sequelae. Genotypic variability of both infected individuals and chlamydial strains are likely to be major factors governing these equilibrium states.

We have focused on *Chlamydia*-infected cancerous epithelial cells *in vitro*. Natural chlamydial infection *in vivo* occurs with fewer organisms and fewer infected cells in a complex and dynamic host environment, often with other bacterial species in close proximity. With appropriate sequencing depth, simultaneous transcriptional profiling by RNA-Seq could be used to examine *Chlamydia* and infected primary host cells from *ex vivo* human tissue or *in vivo* animal models, and ideally, single infected cells. Beyond *Chlamydia*, this approach is applicable to any bacteria (or bacterial community) that interact with eukaryotic cells, encompassing parasitic, commensal or mutualistic lifestyles. Using simultaneous RNA-Seq to compare experimental or environmental conditions, such as different wild-type or recombinant pathogens infecting the same cell type, or the same strain infecting a variety of cell lines or knockouts will give significant insight into bacterial virulence factors and the dynamic host response.

## Methods

### Preparation of *C. trachomatis* EBs and mock lysates

Monolayers of HEp-2 cells were infected with *C. trachomatis* serovar E in SPG as previously described [108]. Additional monolayers were mock-infected with SPG only. The infection was allowed to proceed 48 hours prior to EB harvest, as previously described [108]. *C. trachomatis* EBs and mock-infected cell lysates were subsequently used to infect fresh HEp-2 monolayers.

### Infection time course

HEp-2 cells (American Type Culture Collection, ATCC No. CCL-23) were grown as monolayers in 6×100 mm TC dishes until 90% confluent. Monolayers were infected with *C. trachomatis* serovar E in 3.5 mL SPG buffer for an MOI∼1 as previously described [108], using centrifugation to synchronize infections. Infections and subsequent culture were performed in the absence of cycloheximide or DEAE dextran. A matching number of HEp-2 monolayers were also mock-infected using uninfected cell lysates. Each treatment was incubated at 25°C for 2 h and subsequently washed twice with SPG to remove dead or non-viable EBs. 10 mL fresh medium (DMEM+10% FBS, 25 μg/ml gentamycin, 1.25 μg/ml Fungizone) was added and cell monolayers incubated at 37°C with 5% CO_2_. Three infected and mock-infected dishes per timepoint were harvested post-infection by scraping and resuspending in 150 μL sterile PBS. Resuspended samples were stored at −80°C.

### RNA purification

Total RNA was purified from frozen HEp-2/*C. trachomatis* lysates using the MasterPure RNA Purification kit (Epicentre, Cat. No. MCR85102). Carryover DNA was treated twice with Turbo DNA-free DNase (Ambion, Cat. No. AM1907), according to the manufacturer's protocol for rigorous sample treatment. Total genomic DNA removal was verified by qPCR.

### Simultaneous Eukaryotic and Prokaryotic Ribosomal RNA depletion

Human and Gram-negative bacterial ribosomal RNA were depleted from each sample using Ribo-Zero rRNA Removal (Human/Mouse/Rat and Gram-negative) kits. An equivalent volume of the Ribo-Zero beads from each kit was combined, allowing removal of both human and bacterial rRNA simultaneously. The remainder of the protocol was followed as per the manufacturers instructions. After rRNA reduction, each sample was optionally split and one half subjected to poly-A depletion by the Poly(A)Purist Mag purification kit (Ambion) to further enrich bacterial transcripts. Briefly, poly(A) tailed mRNAs were bound to magnetic beads and removed from solution using a magnet. Poly(A)-depleted and rRNA-depleted eluates were further purified using Zymo-Spin IC columns (Zymo Research) before being combined for library construction. 1 μL of each final RNA eluate was assayed with a RNA Nano chip on an Agilent BioAnalyzer (Agilent Technologies) prior to RNA-Seq library construction and sequencing.

### Quantitative PCR

Complete DNA removal was verified by Taqman (Applied Biosystems) assays for human beta-actin, ATP synthase 6, and 18S rRNA genes. Each assay was performed on an ABI 7900HT instrument according to the manufacturer's instructions (Applied Biosystems). Primers and probes were selected for *C. trachomatis* genes and human genes using PrimerExpress (Applied Biosystems). Assays were performed on an ABI 7900HT instrument according to the manufacturer's instructions for gene expression assays (Applied Biosystems). *C. trachomatis* and human gene expression values were normalized against 16S or 18S rRNA copy number as appropriate. Primer and probe sequences are listed in **Table S6**.

### Sequencing

Illumina mRNA-Seq libraries were prepared from rRNA-depleted samples using the TruSeq RNA Sample Prep kit (Illumina, San Diego, CA) per the manufacturer's protocol with IGS-specific optimizations. Adapters containing 6 nucleotide indexes were ligated to the double-stranded cDNA. The DNA was purified with AMPure XT beads (Beckman Coulter Genomics, Danvers, MA) between enzymatic reactions and size selection steps (∼250 to 300 bp). Libraries were initially sequenced using the Illumina MiSeq sequencer for quality control. MiSeq sequencing results were used to estimate sequencing depth from HiSeq2000 sequencing. Libraries were subsequently sequenced using the 100 bp paired-end protocol on an Illumina HiSeq2000 sequencer. Raw data was processed using Illumina's RTA and CASAVA pipeline software, which includes image analysis, base calling, sequence quality scoring, and index demultiplexing. FastQC (http://www.bioinformatics.bbsrc.ac.uk/projects/fastqc/) and in-house pipelines were used for sequence assessment and quality control. These pipelines report numerous quality metrics and perform a Megablast-based contamination screen. By default, our quality control pipeline assesses basecall quality and truncates reads where the median Phred-like quality score falls below Q20.

### Bioinformatic analyses

Sequence reads were first mapped to the *C. trachomatis* D (NC_000117.1) reference genome using Bowtie v 0.12.7 [109] (maximum number of mismatches  = 2; number of alignments permitted per read  = 1). The remaining sequence reads were aligned to the human (hg19) reference genome using TopHat version 1.3.2 [110] (maximum number of mismatches  = 2; segment length  = 30; maximum multi-hits per read  = 25; maximum intron length  = 50000). Reciprocal mappings were also performed to check that *Chlamydia* reads did not map to the human genome (and vice versa). Mapped RNA-Seq reads were visualized using the Integrative Genomics Viewer [111]. For human reads, the number of reads mapped to each gene was counted by HTSeq (http://www-huber.embl.de/users/anders/HTSeq/) against gene annotation file for build GRCh37/hg19 from Ensembl (http://www.ensembl.org). Read count was used to represent gene expression level. Data normalization and differential expression (DE) analysis were done using the methods implemented in DESeq R package [112]. Briefly, read counts of samples were normalized for sequencing depth and distortion caused by highly differentially expressed genes. A negative binomial (NB) model was used to test the significance of differential expression between two conditions. A cutoff FDR (False Discovery Rate) of less than 0.05 and a log fold change >2.0 was used to select significant DE genes. For *C. trachomatis* reads, RPKMs of features for each sample were divided by their 75^th^ percentile and log_2_ transformed. GO enrichment analysis for human DE genes was performed using the goseq R package [113], normalizing for gene length bias. A cutoff of FDR less than 0.1 was used to select significantly enriched GO categories.

For both human and *C. trachomatis* genes, Gene Ontology annotations and associated data were extracted and arranged into a tab-delimited file corresponding to the GO annotation file (GAF) 2.0 format (http://www.geneontology.org/GO.format.gaf-2_0.shtml#fields). An in-house custom GO ontology was used for *C. trachomatis*. For human, the current GO ontology and generic slim, a subset of the ontology that contains selected high-level terms, were downloaded from http://www.geneontology.org (April 2012). The Perl module map2slim, which maps a gene association file containing annotations to the full GO to terms in a slim, was downloaded, installed and run with the “-c” and “-t” options to generate a count of the number of distinct gene products that either are directly associated to a given slim term or would be associated to a child of this term in the full ontology (http://search.cpan.org/~cmungall/go-perl/scripts/map2slim). Any GO slim terms with zero associations were removed from the resulting table.

## Supporting Information

Figure S1 Gene distributions between timepoints. (a) Unique and shared chlamydial genes highly expressed (RPKM≥1.0 and a minimum of 50 mapped reads) at 1hpi and 24hpi. (b) Unique and shared differentially expressed (FDR≤0.05 and LFC≥2.0) human genes at 1 and 24 hpi.
(PDF)

Figure S2 Expression levels of selected *Chlamydia* genes compared to matching RPKM values. Total RNA was prepared from biological duplicate infections (MOI = 1) at 1 hpi. Quantitative RT-PCR assays were performed on 15 *Chlamydia* genes. Taqman assays were designed for genes CT81, CT500, CT229, CT875, CT734, CT446, CT577, CT18, CT864, CT665, CT834, CT391, CT216, CT705, and CT416. Chlamydial gene expression is plotted against the RPKM for each gene. qRT-PCR data is expressed as 1/log2 Ct and normalized to 16S rRNA.
(PDF)

Figure S3 Expression of 11 *C. trachomatis* E genes from 0 to 16 hpi as detected by qRT- PCR. Infections were performed using *C. trachomatis* (MOI = 1) harvested at 1, 2, 4, 8, and 16 hpi and assayed using gene-specific Taqman primer/probe assays. RNA prepared from purified EBs was used as t = 0. All data are representative of three biological replicates and have been normalized against *C. trachomatis* 16S rRNA transcript abundance. Expression levels are represented as 1/Log Ct. Green arrows indicate increased expression from t = 0 to t = 1 hpi; red arrows indicate decreased expression over the same time.
(PDF)

Figure S4 Quantitative real-time PCR (qRT-PCR) verification of genes detected by simultaneous RNA-Seq. (a) Selected human genes. RPKM values were calculated from human-mapped RNA-Seq reads, and compared to qRT-PCR data for 13 genes. Black circles represent genes examined at 1 hpi (CCL20, cxcl3, Elf3, ERRFI1, ETS1, IL1A, IL8, Lamb3, serpine1, tnc, TNNC1 & TRPV3). Red circles represent genes examined at 24 hpi (BIRC3, CCL20, cxcl3, ELF3, ETS1, IL1A, IL8, Lamb3, serpine1, tnc, tnnc1 & TRPV3). (b) Human gene target relative expression at 1 and 24 hpi. Infected treatments at 1 hpi (dark grey) and 24 hpi (light grey) were normalized relative to mock-infected treatments and expressed as fold change from the mock-infected state. Taqman assays were designed to the following human genes: ccl20, cxcl3, elf3, ERRFI1, ETS1, IL1A, IL18, LAMB3, serpine1, tnc, tnnc1, and TRPV3.
(PDF)

Table S1 RPKM metrics for *Chlamydia* and host cell gene expression at 1 and 24 hpi.
(PDF)

Table S2 Expression of chlamydial genes at various cutoffs. (a) Chlamydial genes at 1 hpi above 0.1 RPKM & 10 mapped reads, ordered by RPKM. (b) Chlamydial genes at 1 hpi above 1 RPKM & 50 mapped reads, ordered by RPKM. (c) Highly expressed chlamydial genes found only at 1 hpi, ordered by RPKM. (d) Chlamydial genes at 24 hpi above 0.1 RPKM & 10 mapped reads, ordered by RPKM. (e) Chlamydial genes at 24 hpi above 1 RPKM & 50 mapped reads, ordered by RPKM. (f) Highly expressed chlamydial genes found at both 1 hpi and 24 hpi, ordered by RPKM. (g) Highly expressed chlamydial genes found only at 24 hpi, ordered by RPKM. Temporal gene expression is noted, if known, from Belland et al (2003) and Nicholson et al (2003).
(PDF)

Table S3 Annotation of previously detected chlamydial genes and Gene Ontology enrichment. (a) Genes detected as expressed at 1 hpi by Belland et al (2003) but not present in hRNA-Seq (RPKM>0.1 and 10 mapped reads) (b) Genes detected as expressed at 1 hpi by Belland et al (2003) but not present in hRNA-Seq at 1 hpi (RPKM>1.0 and 50 mapped reads (c) GO-term enrichment for differentially expressed (versus mock infected) host non-hypothetical genes at 1 hpi (d) GO-term enrichment for differentially expressed (versus mock infected) host non-hypothetical genes at 24 hpi.
(PDF)

Table S4 GO-term enrichment for *Chlamydia* non-hypothetical genes at 1 hpi.
(PDF)

Table S5 Differentially expressed host cell genes at 1 and 24 hpi. (a) Human DE genes at 1 hpi (relative to mock). FDR<0.05 and LFC>2.0. (**b**) Human DE genes at 24 hpi (relative to mock). FDR<0.05 and LFC>2.0. Sorted by gene description.
(PDF)

Table S6 Primer and probe sets used in this study. (a) Custom Taqman primers and probes used for *Chlamydia* qRT-PCR assays. (**b**) Predesigned Taqman primer and probe sets used for human qRT-PCR assays.
(PDF)

Text S1 (PDF)
