# Supplementary figures and images for: Simultaneous Transcriptional Profiling of Bacteria and Their Host Cells
Source: PLoS One. 2013 Dec 4;8(12):e80597. doi: 10.1371/journal.pone.0080597 (PMC3851178; doi:10.1371/journal.pone.0080597)

a.

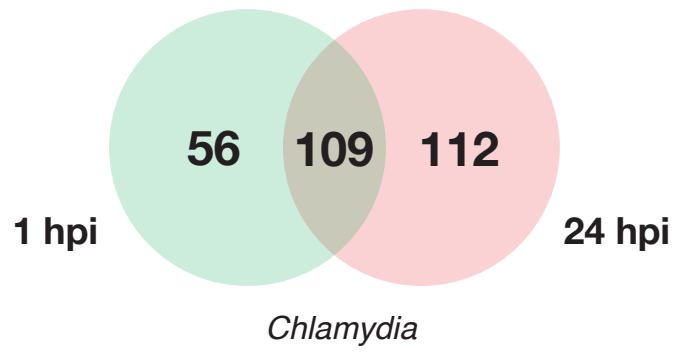

b.

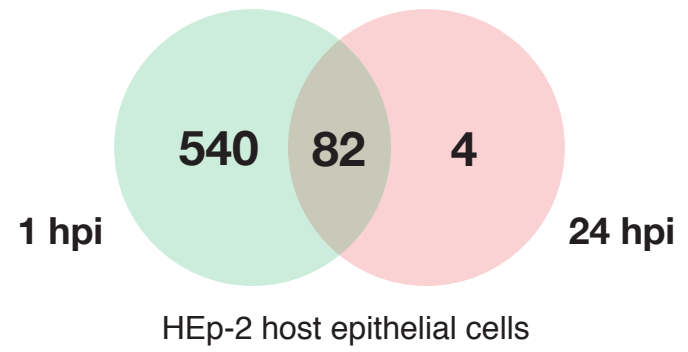

Supplement: Figure S1 — Gene distributions between timepoints. (a) Unique and shared chlamydial genes highly expressed (RPKM≥1.0 and a minimum of 50 mapped reads) at 1hpi and 24hpi. (b) Unique and shared differentially expressed (FDR≤0.05 and LFC≥2.0) human genes at 1 and 24 hpi. (PDF) [file pone.0080597.s001.pdf]

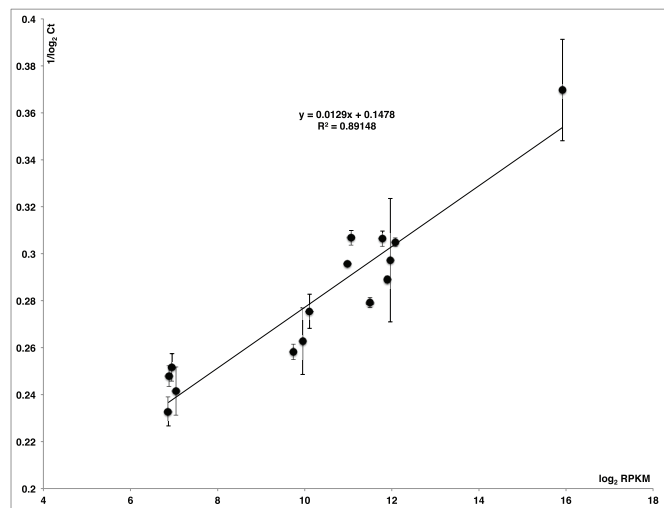

Additional Figure 2

Supplement: Figure S2 — Expression levels of selected Chlamydia genes compared to matching RPKM values. Total RNA was prepared from biological duplicate infections (MOI = 1) at 1 hpi. Quantitative RT-PCR assays were performed on 15 Chlamydia genes. Taqman assays were designed for genes CT81, CT500, CT229, CT875, CT734, CT446, CT577, CT18, CT864, CT665, CT834, CT391, CT216, CT705, and CT416. Chlamydial gene expression is plotted against the RPKM for each gene. qRT-PCR data is expressed as 1/log2 Ct and normalized to 16S rRNA. (PDF) [file pone.0080597.s002.pdf]

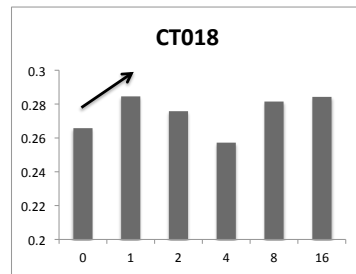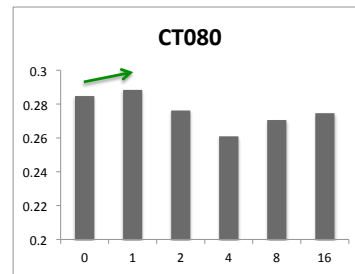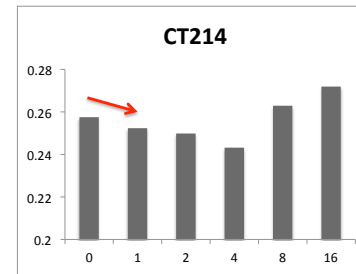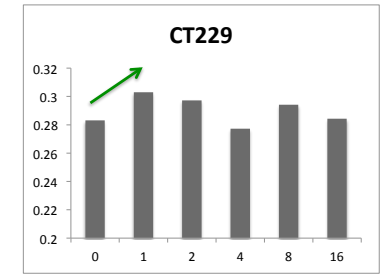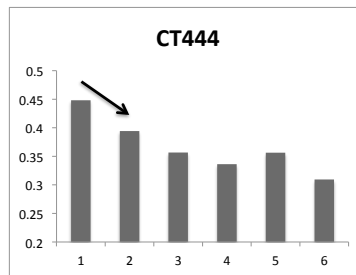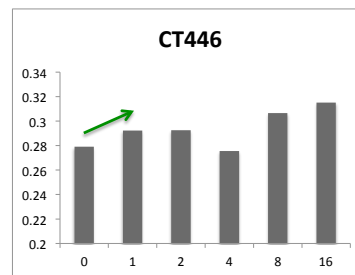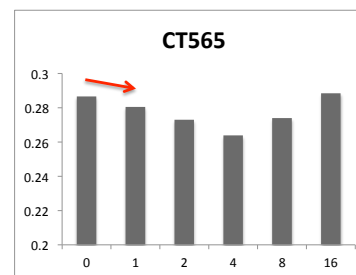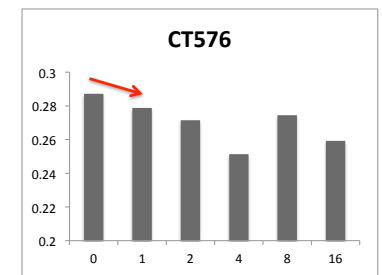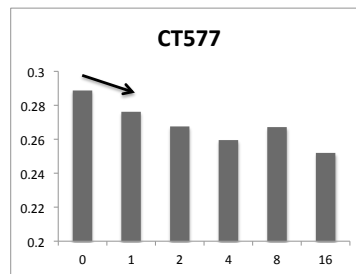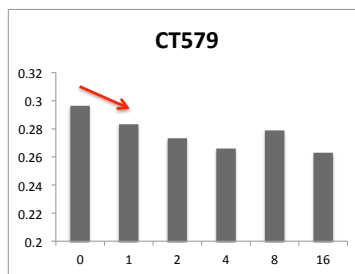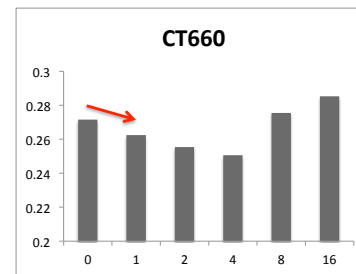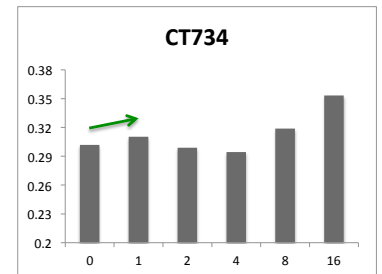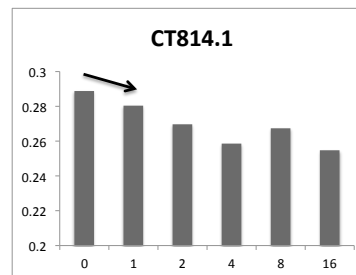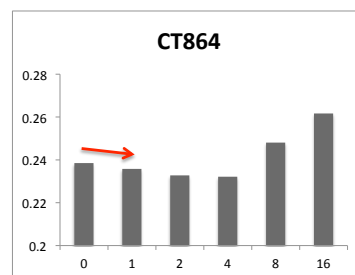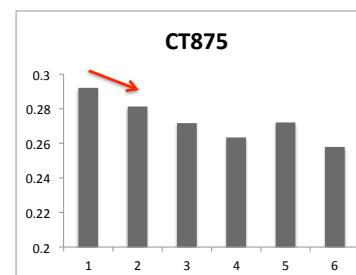

**Additional Figure 3**

Supplement: Figure S3 — Expression of 11 C. trachomatis E genes from 0 to 16 hpi as detected by qRT- PCR. Infections were performed using C. trachomatis (MOI = 1) harvested at 1, 2, 4, 8, and 16 hpi and assayed using gene-specific Taqman primer/probe assays. RNA prepared from purified EBs was used as t = 0. All data are representative of three biological replicates and have been normalized against C. trachomatis 16S rRNA transcript abundance. Expression levels are represented as 1/Log Ct. Green arrows indicate increased expression from t = 0 to t = 1 hpi; red arrows indicate decreased expression over the same time. (PDF) [file pone.0080597.s003.pdf]

a

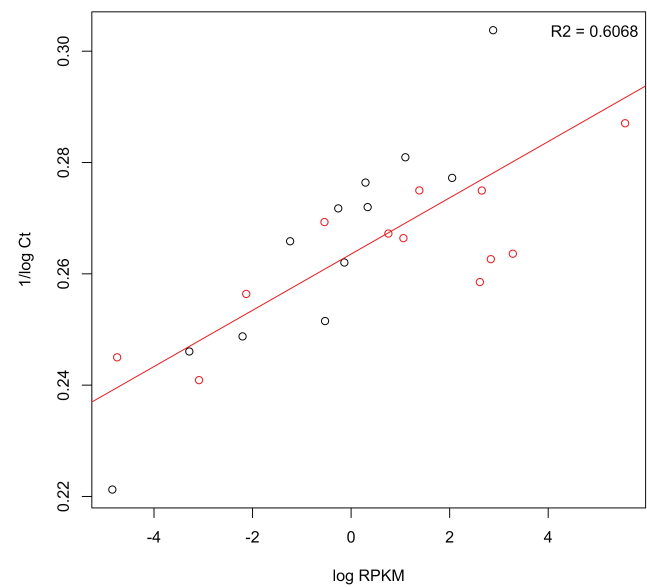

b

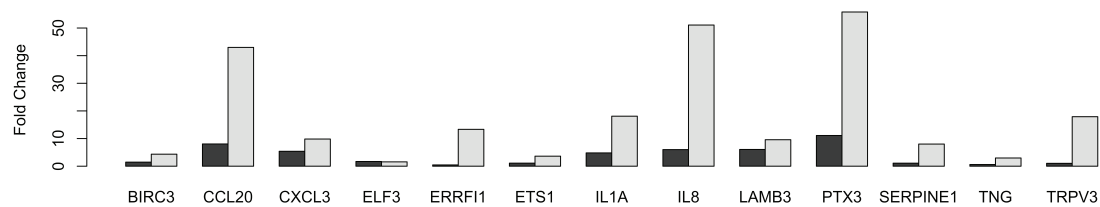

Supplement: Figure S4 — Quantitative real-time PCR (qRT-PCR) verification of genes detected by simultaneous RNA-Seq. (a) Selected human genes. RPKM values were calculated from human-mapped RNA-Seq reads, and compared to qRT-PCR data for 13 genes. Black circles represent genes examined at 1 hpi (CCL20, cxcl3, Elf3, ERRFI1, ETS1, IL1A, IL8, Lamb3, serpine1, tnc, TNNC1 & TRPV3). Red circles represent genes examined at 24 hpi (BIRC3, CCL20, cxcl3, ELF3, ETS1, IL1A, IL8, Lamb3, serpine1, tnc, tnnc1 & TRPV3). (b) Human gene target relative expression at 1 and 24 hpi. Infected treatments at 1 hpi (dark grey) and 24 hpi (light grey) were normalized relative to mock-infected treatments and expressed as fold change from the mock-infected state. Taqman assays were designed to the following human genes: ccl20, cxcl3, elf3, ERRFI1, ETS1, IL1A, IL18, LAMB3, serpine1, tnc, tnnc1, and TRPV3. (PDF) [file pone.0080597.s004.pdf]
