# Supplementary material for: Simultaneous Transcriptional Profiling of Bacteria and Their Host Cells
Source: PLoS One. 2013 Dec 4;8(12):e80597. doi: 10.1371/journal.pone.0080597 (PMC3851178; doi:10.1371/journal.pone.0080597)
Supplement: Table S1 — RPKM metrics for Chlamydia and host cell gene expression at 1 and 24 hpi. (PDF) [file pone.0080597.s005.pdf]

Table S1. RPKM metrics for *Chlamydia* and host cell expression at 1 and 24 hpi

	**mean**	**SD**	**median**
Chlamydia			
**1 hpi**	1.998	12.515	0.226
**24 hpi**	1.881	12.441	0.411
**Human**			
**1 hpi *Chlamydia*-infected**	21.564	1213.702	0.297
**1 hpi mock-infected**	26.490	1260.899	0.236
**24 hpi *Chlamydia*-infected**	37.416	2420.190	0.335
**24 hpi mock-infected**	24.020	840.446	0.455
