# Supplementary material for: Simultaneous Transcriptional Profiling of Bacteria and Their Host Cells
Source: PLoS One. 2013 Dec 4;8(12):e80597. doi: 10.1371/journal.pone.0080597 (PMC3851178; doi:10.1371/journal.pone.0080597)
Supplement: Table S2 — Expression of chlamydial genes at various cutoffs. (a) Chlamydial genes at 1 hpi above 0.1 RPKM & 10 mapped reads, ordered by RPKM. (b) Chlamydial genes at 1 hpi above 1 RPKM & 50 mapped reads, ordered by RPKM. (c) Highly expressed chlamydial genes found only at 1 hpi, ordered by RPKM. (d) Chlamydial genes at 24 hpi above 0.1 RPKM & 10 mapped reads, ordered by RPKM. (e) Chlamydial genes at 24 hpi above 1 RPKM & 50 mapped reads, ordered by RPKM. (f) Highly expressed chlamydial genes found at both 1 hpi and 24 hpi, ordered by RPKM. (g) Highly expressed chlamydial genes found only at 24 hpi, ordered by RPKM. Temporal gene expression is noted, if known, from Belland et al (2003) and Nicholson et al (2003). (PDF) [file pone.0080597.s006.pdf]

Table S2 (a) Chlamydial genes at 1 hpi above 0.1 RPKM & 10 mapped reads, ordered by RPKM. (b) Chlamydial genes at 1 hpi above 1 RPKM & 50 mapped reads, ordered by RPKM . (c) Highly expressed chlamydial genes found only at 1 hpi, ordered by RPKM. (d) Chlamydial genes at 24 hpi above 0.1 RPKM & 10 mapped reads, ordered by RPKM. (e) Chlamydial genes at 24 hpi above 1 RPKM & 50 mapped reads, ordered by RPKM. (f) Highly expressed chlamydial genes found at both 1 hpi and 24 hpi, ordered by RPKM. (g)Highly expressed chlamydial genes found only at 24 hpi, ordered by RPKM. Temporal gene expression is noted, if known, from Belland et al (2003) and Nicholson et al (2003].

(a)

Gene ID	Gene Symbol	Read Count	RPKM	Gene Name	Belland Classifier	Nicholson Classifier
CT444	omcA	2306	87.7747516	9kDa-cysteine-rich lipoprotein	Late	Late
CT229		2383	38.4263261	hypothetical protein	Early	
CT081		1177	37.4773225	hypothetical protein		Very late
CT603	ahpC	1599	28.5122655	thioredoxin peroxidase		
CT065		3849	26.89672	ADP/ATP translocase	Early	
CT734		1484	22.1086671	hypothetical protein	Early	Midlate
CT617	rpsT	624	22.0062654	30S ribosomal protein S20		
CT786	rpmJ	284	21.9935572	50S ribosomal protein L36		
CT446	euo	1006	19.5368495	hypothetical protein	Early	
CT117		560	17.595967	inclusion membrane protein F	Early	
CT116		661	17.4526724	inclusion membrane protein E	Early	
CT134		702	17.3484556	hypothetical protein		
CT228		908	17.0485483	hypothetical protein	Early	
CT785	rpmH	173	14.5362477	50S ribosomal protein L34		
CT843	rpsO	345	14.4435029	30S ribosomal protein S15		
CT794.1		374	13.5264828	hypothetical protein		
CT500	ndk	569	13.3095475	nucleoside diphosphate kinase		Midlate
CT018		508	13.060744	hypothetical protein		
CT508	rs11	463	12.6846902	30S ribosomal protein S11		
CT602		458	12.0117368	hypothetical protein		
CT787	rpsN	291	11.3135052	30S ribosomal protein S14		
CT524	rpsS	249	11.0926694	30S ribosomal protein S19		
CT802	rpsR	255	10.6373142	30S ribosomal protein S18		
CT801	rpsF	320	10.614898	30S ribosomal protein S6		
CT080	ltuB	281	10.511727	late transcription unit B protein	Late	Late
CT316	rplL	339	10.494848	50S ribosomal protein L7/L12		
CT135		1002	10.1194002	hypothetical protein		
CT022	rpmE2	327	9.9843869	50S ribosomal protein L31 type B		
CT342	rpsU	142	9.8207615	30S ribosomal protein S21		
CT509	rpsM	319	9.3592016	30S ribosomal protein S13		
CT439m	rpsL	303	8.9849504	30S ribosomal protein S12		
CT795		383	8.8247604	hypothetical protein	Early	
CT525	rplB	735	8.7146543	50S ribosomal protein L2		
CT526	rplW	246	8.4948625	50S ribosomal protein L23		
CT834	rpmI	140	8.2391597	50S ribosomal protein L35		
CT569		270	8.1425376	general secretion pathway protein G		
CT784	rnpA	254	7.9963338	ribonuclease P		
CT115		330	7.9944854	inclusion membrane protein D	Early	
CT319	rplK	300	7.7923786	50S ribosomal protein L11		
CT421.1		101	7.7402745	hypothetical protein		
CT317	rplJ	374	7.6551861	50S ribosomal protein L10		
CT523	rplV	188	7.4062165	50S ribosomal protein L22		
CT265	accA	642	7.2790527	acetyl-CoA carboxylase carboxyltransferase subunit alpha		
CT810	rpmF	114	7.1804193	50S ribosomal protein L32		

CT398.1		896	7.1613702	Ribonuclease P RNA		
CT421.2		99	7.0918647	hypothetical protein		
CT438	rs7	291	6.9443567	30S ribosomal protein S7		
CT568		311	6.9425411	hypothetical protein		
CT659		152	6.8134292	hypothetical protein	Late	Late
CT150	rpmG	85	6.7045597	50S ribosomal protein L33		
CT318	rplA	397	6.6948475	50S ribosomal protein L1		
CT518	rplN	210	6.3617879	50S ribosomal protein L14		
CT443	omcB	1039	6.1710133	60kD cysteine-rich outer membrane protein	Late	Late
CT322	tuf	670	6.1607117	elongation factor Tu		
CT026	rpsP	178	6.0953965	30S ribosomal protein S16		
CT125	rplM	256	6.0072041	50S ribosomal protein L13		
CT402	lpxK	698	5.9644665	tetraacyldisaccharide 4'-kinase		
CT513	rplR	193	5.9340593	50S ribosomal protein L18		
CT618		415	5.881029	hypothetical protein		Midlate
CT803	rplI	277	5.84832	50S ribosomal protein L9		
CT519	rpsQ	135	5.6762988	30S ribosomal protein S17		
CT506	rplQ	221	5.6699312	50S ribosomal protein L17		
CT528	rplC	308	5.6467981	50S ribosomal protein L3		
CT507	rpoA	625	5.6401849	DNA-directed RNA polymerase subunit alpha		
CT517	rplX	153	5.6058867	50S ribosomal protein L24		
CT098	rpsA	882	5.5326116	30S ribosomal protein S1		
CT409		710	5.5246412	amino acid permease		
CT647		303	5.4690497	hypothetical protein		
CT147		2156	5.4657397	hypothetical protein	Early	Very late
CT691		346	5.33416	hypothetical protein		
CT565		212	5.2395889	hypothetical protein	Late	Late
CT799	ctc	288	5.1598131	50S ribosomal protein L25/general stress protein Ctc		
CT835	rplT	163	5.0284141	50S ribosomal protein L20		
CT512	rpsE	213	4.9882387	30S ribosomal protein S5		
CT126	rpsI	149	4.9733706	30S ribosomal protein S9		
CT420	rplU	156	4.862071	50S ribosomal protein L21		
CT833	infC	223	4.8054049	translation initiation factor IF-3		
CT326.2		79	4.762166	hypothetical protein		
CT567		239	4.7369079	hypothetical protein		
CT086	rpmB	113	4.6541717	50S ribosomal protein L28		
CT398		334	4.6252794	hypothetical protein		
CT522	rpsC	277	4.5980187	30S ribosomal protein S3		
CT221	yqfU	387	4.5267681	hypothetical protein		
CT511	rplO	188	4.5056723	50S ribosomal protein L15		
CT118		242	4.3247408	inclusion membrane protein G	Early	
CT514	rplF	223	4.3073965	50S ribosomal protein L6		
CT648		493	4.19834	hypothetical protein		
CT516	rplE	189	4.1667211	50S ribosomal protein L5		
CT436	rpsJ	110	4.1026185	30S ribosomal protein S10		
CT249		128	4.0990471	hypothetical protein		Very late
CT573		467	4.0737107	hypothetical protein		
CT527	rplD	274	4.0619628	50S ribosomal protein L4		
CT437	fusA	777	4.0478134	elongation factor G		
CT572	gspD	844	4.0006388	general secretion pathway protein D		
CT626	rpsD	230	3.9992337	30S ribosomal protein S4		
CT520	rpmC	83	3.9330767	50S ribosomal protein L29		

CT377	ltuA	76	3.9194882	late transcription unit A protein		Midlate
CT571	gspE	558	3.9171093	general secretion pathway protein E		
CT832	nusB	180	3.8710798	transcription antitermination protein NusB		
CT515	rpsH	154	3.8421699	30S ribosomal protein S8		
CT341	dnaJ	379	3.7427793	chaperone protein DnaJ		
CT343		192	3.3995728	O-sialoglycoprotein endopeptidase family protein		
CT735	dagA_2	444	3.1230752	Na( )-linked D-alanine glycine permease	Early	
CT111	groES	91	3.082376	co-chaperonin GroES	Early	
CT419	rpmA	66	3.0421696	50S ribosomal protein L27		
CT095	rbfA	113	3.0155688	ribosome-binding factor A		
CT498	gidA	484	3.0051616	tRNA uridine 5-carboxymethylaminomethyl modification enzyme GidA		
CT723	yjbC	224	2.9879276	ribosomal large subunit pseudouridine synthase B		
CT028	rplS	109	2.9638218	50S ribosomal protein L19		
CT775		198	2.9076679	1-acyl-sn-glycerol-3-phosphate acyltransferase		
CT314	rpoC	1098	2.8219162	DNA-directed RNA polymerase subunit beta'		
CT521	rplP	131	2.7961337	50S ribosomal protein L16		
CT169	trpR	82	2.7728044	Trp operon repressor		
CT867		254	2.7474293	hypothetical protein	Late	
CT570	gspF	283	2.7060197	general secretion pathway protein F		
CT327	trpC	150	2.7059324	N-(5'-phosphoribosyl)anthranilate isomerase		
CT809		66	2.6971523	hypothetical protein		
CT529		272	2.6939747	hypothetical protein	Early	
CT566		263	2.6882372	hypothetical protein		
CT110	groEL	384	2.6829135	chaperonin GroEL	Early	Midcycle
CT096	infB	611	2.5912625	translation initiation factor IF-2		
CT097	nusA	270	2.4909793	transcription elongation factor NusA		
CT315	rpoB	812	2.475575	DNA-directed RNA polymerase subunit beta		
CT321	secE	59	2.4504893	preprotein translocase subunit SecE		
CT025	ffh	288	2.4388213	signal recognition particle, subunit FFH/SRP54		
CT510	secY	299	2.4092502	preprotein translocase subunit SecY		
CT401	gltT	315	2.3706737	glutamate symport		
CT842	pnp	442	2.3693412	polynucleotide phosphorylase/polyadenylase		Midlate
CT285	lplA_1	145	2.3525831	lipoate-protein ligase A		
CT136		160	2.304708	lysophospholipase esterase		
CT496	pgsA_1	112	2.2922814	CDP-diacylglycerol--glycerol-3-phosphate 3-phosphatidyltransferase		Midlate
CT788		100	2.2701766	leader (60) peptide-periplasmic		
CT768		342	2.1965764	hypothetical protein		Midlate
CT505	gapA	187	2.1909558	glyceraldehyde-3-phosphate dehydrogenase		Midlate
CT808	cafE	330	2.1492329	ribonuclease E		
CT383		118	2.0917038	hypothetical protein		
CT320	nusG	106	2.051111	transcription antitermination protein NusG		
CT504		135	1.995123	hypothetical protein		Midlate
CT310	atpE	124	1.9894525	V-type ATP synthase subunit E		
CT405	ribC	89	1.9839052	riboflavin synthase subunit alpha		
CT353	def	74	1.9784532	peptide deformylase		
CT027	trmD	197	1.9611041	tRNA (guanine-N(1)-)-methyltransferase/unknown domain fusion protein		
CT292	dut	89	1.9591035	deoxyuridine 5'-triphosphate nucleotidohydrolase		
CT046	hctB	113	1.9300894	histone-like protein 2	Late	Late
CT005		176	1.926978	hypothetical protein		Late

CT288		307	1.9231752	hypothetical protein	Early	Late
CT287	mnmA	201	1.9185056	tRNA-specific 2-thiouridylase MnmA		
CT182	kdsB	142	1.8348909	3-deoxy-manno-octulosonate cytidylyltransferase		
CT836	pheS	182	1.8241992	phenylalanyl-tRNA synthetase subunit alpha		
CT214		278	1.7854991	hypothetical protein	Late	Late
CT800	pth	74	1.764235	peptidyl-tRNA hydrolase		
CT579		223	1.7564259	hypothetical protein	Late	Late
CT495		249	1.7408536	ADP/ATP translocase	Early	
CT017		188	1.7345002	hypothetical protein		Late
CT554	brnQ	204	1.7029149	branched chain amino acid ABC transporter carrier protein		
CT841	ftsH	450	1.6985594	ATP-dependent zinc protease		Late
CT492	coaE	89	1.6979562	dephospho-CoA kinase		Midlate II
CT731	ribA/ribB	202	1.6974322	bifunctional 3,4-dihydroxy-2-butanone 4-phosphate synthase/GTP cyclohydrolase II protein		
CT736	ybcL	93	1.6969612	hypothetical protein		
CT678	pyrH	109	1.6788329	uridylate kinase		
CT029	rnhB	100	1.6384954	ribonuclease HII		
CT359		100	1.6359142	hypothetical protein		
CT066		74	1.565509	hypothetical protein		Midlate
CT133		110	1.5653594	rRNA methylase	Early	
CT180	tauB	94	1.5420385	nitrate/iron ABC transporter ATPase		
CT703	engA	197	1.4893178	GTP-binding protein EngA		
CT284		211	1.4880551	phospholipase D		
CT348	yjjK	204	1.4578815	ABC transporter ATPase		
CT094	truB	98	1.4465584	tRNA pseudouridine synthase B		
CT538		83	1.3874105	hypothetical protein		
CT129	glnP	95	1.3764455	amino acid ABC transporter ATPase		
CT875		154	1.3742533	hypothetical protein		Late
CT776	aas	213	1.3444105	long chain fatty acid--[acyl-carrier-protein] ligase		Late
CT035		94	1.3245183	biotin protein ligase	Early	Very late
CT217	ydaO	70	1.3228236	PP-loop superfamily ATPase		
CT601	papQ	71	1.3158358	invasin repeat-containing phosphatase		
CT364	dapB	83	1.3137126	dihydrodipicolinate reductase		
CT811	plsX	98	1.2130594	putative glycerol-3-phosphate acyltransferase PlsX		
CT429		112	1.2109531	hypothetical protein		
CT313	tal	123	1.2007853	transaldolase B		Midlate
CT264	msbA	229	1.1727573	transport ATP binding protein		
CT543	hisS	109	1.1594402	histidyl-tRNA synthetase		
CT034	ytfF	110	1.1488303	cationic amino acid transporter		
CT820	ftsY	90	1.1437511	cell division protein FtsY		
CT397	vacB	199	1.1199961	exoribonuclease II		
CT445	gltX	138	1.1180688	glutamyl-tRNA synthetase		
CT410	pcnB_1	119	1.107962	polyA polymerase		
CT183	pyrG	147	1.0955577	CTP synthetase		
CT613	folP	116	1.093358	dihydropteroate synthase		
CT422		56	1.0884551	metalloenzyme		
CT857		129	1.0657594	Na /H antiporter		
CT293	accD	104	1.0473762	acetyl-CoA carboxylase subunit beta		
CT515	rpsH	5266	1.1688084	30S ribosomal protein S8		
CT514	rplF	7425	1.1655267	50S ribosomal protein L6		
CT351a	secG	4203	1.1609324	preprotein translocase subunit SecG		
CT275	dnaA	18449	1.1588853	chromosomal replication initiation protein		

CT858		25646	1.146175	protease		
CT353	def	7131	1.1425044	peptide deformylase		
CT550		5688	1.1359986	hypothetical protein		Midlate
CT672	fliN	15008	1.1273801	type III secretion system protein		Midlate
CT582	minD	9935	1.1060616	chromosome partitioning ATPase-CHLTR plasmid protein homolog GP5D		
CT522	rpsC	8196	1.0991597	30S ribosomal protein S3		
CT321	secE	3168	1.0987296	preprotein translocase subunit SecE		
CT364	dapB	9725	1.0956429	dihydrodipicolinate reductase		
CT670		6527	1.0825593	hypothetical protein		Midlate
CT031		3872	1.0804687	hypothetical protein		
CT543	hisS	15751	1.0767884	histidyl-tRNA synthetase		
CT012	ybbP	9956	1.0632189	hypothetical protein		
CT294	sodM	7590	1.0585806	superoxide dismutase		
CT533	lpxC	10731	1.0577092	UDP-3-O-[3-hydroxymyristoyl] N-acetylglucosamine deacetylase		
CT094	truB	8753	1.0539123	tRNA pseudouridine synthase B		
CT253		8157	1.041969	hypothetical protein		
CT046	hctB	8006	1.0412061	histone-like protein 2	Late	Late
CT069	ytgC	16666	1.0305912	integral membrane protein		
CT090	lcrD	26550	1.0293166	low calcium response D		Midlate
CT674	yscC	34454	1.024511	Yop proteins translocation protein C/general secretion pathway protein		Midlate II
CT320	nusG	6491	1.0224418	transcription antitermination protein NusG		
CT070	ytgD	11759	1.0208357	integral membrane protein		
CT291	ptsN_2	5515	1.0153351	PTS IIA protein		
CT535	yciA	5518	1.0032389	acyl-CoA hydrolase		Midlate
CT044	ssb	5479	0.9904083	single-stranded DNA-binding protein		
CT327	trpC	7187	0.9840054	N-(5'-phosphoribosyl)anthranilate isomerase		
CT476		11122	0.9823909	hypothetical protein		Midlate
CT821	sucC	12691	0.9762476	succinyl-CoA synthetase subunit beta		
CT027	trmD	11482	0.96954	tRNA (guanine-N(1)-)-methyltransferase/unknown domain fusion protein		
CT502	ruvC	5580	0.9694396	Holliday junction resolvase		
CT557	lpdA	15592	0.9681899	dihydrolipoamide dehydrogenase		
CT251		26994	0.9536828	putative inner membrane protein translocase component YidC		
CT540	yibK	5075	0.9465289	rRNA methylase		
CT532	fabZ	5043	0.9388933	(3R)-hydroxymyristoyl-ACP dehydratase		
CT521	rplP	4418	0.9382922	50S ribosomal protein L16		
CT268	amiA	8178	0.9377575	N-acetylmuramoyl-L-alanine amidase		
CT362	lysC	13835	0.9376548	aspartate kinase		
CT189	gyrA_1	28606	0.9348411	DNA gyrase subunit A		
CT282	gcsH	3820	0.9194834	glycine cleavage system protein H		
CT273		6007	0.9185747	hypothetical protein		Midlate
CT700		15172	0.9137172	hypothetical protein		Midlate
CT872	pmpH	33051	0.912142	polymorphic outer membrane protein	Late	Midlate
CT295	mrsA_1	19168	0.9095698	phosphoglucomutase		
CT354	ksgA	8424	0.9040696	dimethyladenosine transferase		
CT740	dmpP	13855	0.9024917	Na( )-translocating NADH-quinone reductase subunit F		
CT266		12514	0.8951058	hypothetical protein		
CT836	pheS	10563	0.8913559	phenylalanyl-tRNA synthetase subunit alpha		
CT218	surE	8721	0.8879608	stationary phase survival protein SurE		Midlate
CT669	yscN	13889	0.8864376	type III secretion system ATPase		Midlate

CT461	yaeI	9943	0.8823883	phosphohydrolase		
CT278	nqr2	15927	0.868281	Na( )-translocating NADH-quinone reductase subunit B		Midlate
CT142		8598	0.867902	hypothetical protein		Midlate II
CT563	yscS	2881	0.8626409	Yop proteins translocation protein S		
CT480	oppA_4	20784	0.8541989	oligopeptide binding lipoprotein	Early	Midlate
CT728		7705	0.8515256	hypothetical protein		
CT618		7634	0.850625	hypothetical protein		Midlate
CT823	htrA	14896	0.8489177	DO Serine protease		
CT029	rnhB	6397	0.8433331	ribonuclease HII		
CT395	grpE	5975	0.8398377	HSP-70 cofactor		Late
CT283		20733	0.8392204	hypothetical protein		
CT531	lpxA	8592	0.8391524	UDP-N-acetylglucosamine acyltransferase		Midlate
CT841	ftsH	27925	0.8387935	ATP-dependent zinc protease		Late
CT759	nlpD	7179	0.8331568	muramidase		
CT652.1		1788	0.8330283	hypothetical protein		
CT457	yebC	6588	0.8321948	hypothetical protein		Late
CT675	karG	10730	0.8303431	ATP:guanido phosphotransferase		
CT710	pckA	15918	0.8270178	phosphoenolpyruvate carboxykinase		Midlate
CT071	yaeM	10984	0.8255771	1-deoxy-D-xylulose 5-phosphate reductoisomerase		
CT587	eno	12754	0.8210394	phosphopyruvate hydratase		
CT117		2974	0.8148149	inclusion membrane protein F	Early	
CT832	nusB	4738	0.8066813	transcription antitermination protein NusB		
CT376	mdhC	9064	0.8018162	malate dehydrogenase	Early	Midlate
CT585	trpS	9791	0.8008935	tryptophanyl-tRNA synthetase		
CT014	cydB	10078	0.8007174	cytochrome d ubiquinol oxidase subunit II		Midlate
CT773	ldh	9443	0.800319	leucine dehydrogenase		
CT025	ffh	12260	0.7985362	signal recognition particle, subunit FFH/SRP54		
CT397	vacB	18971	0.7968309	exoribonuclease II		
CT486	fliY	7469	0.7947251	glutamine-binding protein		
CT542	aspS	15770	0.7903122	aspartyl-tRNA synthetase		
CT875		11136	0.7901522	hypothetical protein		Late
CT286	clpC	25165	0.7893601	ClpC protease ATPase		Midlate II
CT378	pgi	14454	0.7854645	glucose-6-phosphate isomerase		Midlate
CT601	papQ	5643	0.7845385	invasin repeat-containing phosphatase		
CT795		4731	0.7844806	hypothetical protein	Early	
CT349	maf	5394	0.782514	Maf-like protein		
CT871	pmpG	27645	0.7725738	polymorphic outer membrane protein		Midlate
CT330		2448	0.7701522	hypothetical protein		
CT113	clpB	23628	0.7697211	Clp protease ATPase		
CT606.1		2022	0.7680308	hypothetical protein		
CT536	dnaQ_2	6877	0.755695	DNA polymerase III subunit epsilon		
CT215	dhnA	9436	0.7493686	fructose-bisphosphate aldolase		Midlate
CT401	gltT	10188	0.7492583	glutamate symport		
CT023	prfA	9631	0.7473857	peptide chain release factor 1		
CT265	accA	8748	0.7431151	acetyl-CoA carboxylase carboxyltransferase subunit alpha		
CT404		7043	0.7423473	N6-adenine-specific DNA methylase		
CT776	aas	13814	0.7354244	long chain fatty acid--[acyl-carrier-protein] ligase		Late
CT849		3954	0.7340779	hypothetical protein		
CT422		4102	0.7297329	metalloenzyme		
CT625	nfo	7106	0.7290546	endonuclease IV		
CT122	efp_1	4787	0.7276601	elongation factor P		
CT630	cpxR	5609	0.7228291	HTH transcriptional regulatory protein and receiver domain		

CT657		2761	0.72082	hypothetical protein		
CT750	tktB	16109	0.7170629	transketolase		
CT733		11091	0.7126125	hypothetical protein		Late
CT072	yaeL	15073	0.7059336	metalloprotease		
CT239	fabH	7402	0.7006368	3-oxoacyl-(acyl carrier protein) synthase III		Midlate
CT455	murA	10427	0.6933554	UDP-N-acetylglucosamine 1-carboxyvinyltransferase		Midlate
CT344	lon	19769	0.6918818	ATP-dependent protease La		Midlate
CT421		5722	0.6909607	hypothetical protein		
CT479	oppB_2	12021	0.6888977	oligopeptide permease		
CT237	fabG	5803	0.6870492	3-ketoacyl-(acyl-carrier-protein) reductase		Midlate
CT003	gatA	12398	0.6869712	aspartyl/glutamyl-tRNA amidotransferase subunit A		
CT822	sucD	6586	0.6836571	succinyl-CoA synthetase subunit alpha		
CT212		3573	0.6834509	hypothetical protein		Late
CT797	pgsA_2	4700	0.6830251	CDP-diacylglycerol--glycerol-3-phosphate 3-phosphatidyltransferase		
CT260		4034	0.6735139	hypothetical protein		
CT627	yceA	8230	0.6725201	hypothetical protein		
CT190	gyrB	19288	0.6690327	DNA gyrase subunit B		
CT053		3555	0.6679867	hypothetical protein		Midlate
CT116		3172	0.6639256	inclusion membrane protein E	Early	
CT030	gmk	4928	0.6615267	guanylate kinase		
CT432	glyA	11340	0.6573544	serine hydroxymethyltransferase		Midlate
CT804	ychB	6647	0.6563911	4-diphosphocytidyl-2-C-methyl-D-erythritol kinase		
CT303		3980	0.6560009	hypothetical protein		
CT654		3709	0.6558302	hypothetical protein		
CT408	lspA	3720	0.6512336	lipoprotein signal peptidase		Midlate
CT560		6169	0.6500661	hypothetical protein		
CT185	zwf	11936	0.6426318	glucose-6-phosphate 1-dehydrogenase		
CT358		3829	0.6343157	hypothetical protein		
CT704	pcnB_2	9144	0.6329503	polyA polymerase		
CT735	dagA_2	9486	0.6305126	Na( )-linked D-alanine glycine permease	Early	
CT623		10090	0.628459	hypothetical protein		
CT276		4132	0.6281464	hypothetical protein		
CT004	gatB	11233	0.6269354	aspartyl/glutamyl-tRNA amidotransferase subunit B		
CT820	ftsY	6232	0.6266811	cell division protein FtsY		
CT758	murD	9220	0.6240406	UDP-N-acetylmuramoyl-L-alanyl-D-glutamate synthetase		
CT089	lcrE	9325	0.6221447	low calcium response E		Midlate
CT363	asd	7327	0.6218461	aspartate-semialdehyde dehydrogenase		
CT448	secD/secF	30554	0.6211635	bifunctional preprotein translocase subunit SecD/SecF		
CT399	yrbH	7129	0.6210097	GutQ/KpsF family sugar-P isomerase		
CT326.2		1309	0.6200574	hypothetical protein		
CT445	gltX	11370	0.6196809	glutamyl-tRNA synthetase		
CT290	ptsN_1	4700	0.6191686	PTS IIA protein HTH DNA-binding domain		
CT403	yjfH	5672	0.6174237	rRNA methylase		
CT413	pmpB	37899	0.6159743	putative outer membrane protein B		Midlate
CT304	atpK	3006	0.6126116	V-type ATP synthase subunit K		
CT040	ruvB	6919	0.6120047	Holliday junction DNA helicase RuvB		Late
CT634		9651	0.6039711	Na( )-translocating NADH-quinone reductase subunit A		Midlate
CT137	ywlC	5839	0.6024462	SuA5 superfamily-related protein		
CT075	dnaN	9062	0.60223	DNA polymerase III subunit beta		
CT216	xasA	10208	0.5956258	glutamate/gamma-aminobutyrate antiporter		Midlate
CT332	pykF	9902	0.5916113	pyruvate kinase		

CT281	nqr5	5145	0.5910161	Na( )-translocating NADH-quinone reductase subunit E	
CT337	ptsH	2274	0.5882068	phosphocarrier protein HPr	
CT143		5720	0.5873744	hypothetical protein	Midlate II
CT271		1849	0.5865432	hypothetical protein	
CT311		4748	0.583206	hypothetical protein	
CT310	atpE	4234	0.5830783	V-type ATP synthase subunit E	
CT091	yscU	7200	0.582205	type III secretion system protein	
CT583	gp6D	5289	0.5820642	virulence plasmid protein pGP6-D-related protein	
CT608	uvrD	13326	0.5814687	DNA helicase	
CT631		1607	0.5692171	hypothetical protein	
CT808	cafE	10571	0.5691118	ribonuclease E	
CT811	plsX	6551	0.5675709	putative glycerol-3-phosphate acyltransferase PlsX	
CT816	glmS	12201	0.564756	glucosamine--fructose-6-phosphate aminotransferase	Midlate
CT719	fliF	6217	0.5641287	type III secretion system protein	
CT409		8768	0.557485	amino acid permease	
CT032	metG	11386	0.5563218	methionyl-tRNA synthetase	
CT045	pepA	9917	0.5562577	leucyl aminopeptidase	Midlate
CT178		7697	0.5548816	hypothetical protein	
CT615	rpoD	11485	0.5513154	RNA polymerase sigma factor	
CT869	pmpE	19054	0.5502338	polymorphic outer membrane protein	Late
CT428	ubiE	4407	0.5484824	ubiquinone/menaquinone biosynthesis methyltransferase	
CT261	dnaQ_1	4434	0.5477207	DNA polymerase III subunit epsilon	
CT123	accB	3185	0.545436	acetyl-CoA carboxylase biotin carboxyl carrier protein subunit	
CT151		9724	0.5444222	hypothetical protein	
CT466		2039	0.5442333	hypothetical protein	
CT102		2958	0.5439093	hypothetical protein	
CT729	serS	8144	0.5378491	seryl-tRNA synthetase	
CT373		3764	0.5377769	hypothetical protein	Midlate
CT866	glgB	13654	0.5363302	glycogen branching enzyme	
CT731	ribA/ribB	7970	0.5349674	bifunctional 3,4-dihydroxy-2-butanone 4-phosphate synthase/GTP cyclohydrolase II protein	
CT690	dppD	5900	0.5337531	peptide ABC transporter ATPase	
CT099	trxB	6736	0.5329169	thioredoxin reductase	
CT853		3792	0.5274442	hypothetical protein	
CT530	fmt	5952	0.5266046	methionyl-tRNA formyltransferase	
CT011		7988	0.5262418	hypothetical protein	
CT464		4090	0.5223911	hydrolase, haloacid dehalogenase-like family	
CT696		7234	0.5221324	hypothetical protein	
CT287	mnmA	6673	0.5199122	tRNA-specific 2-thiouridylase MnmA	
CT293	accD	5608	0.5186432	acetyl-CoA carboxylase subunit beta	
CT629	glmU	3734	0.5146657	glucosamine-1-phosphate acetyltransferase	
CT643	topA	15566	0.5110588	DNA topoisomerase I/SWI domain fusion protein	Late
CT490		1539	0.5073052	hypothetical protein	
CT280	nqr4	3945	0.5060098	Na( )-translocating NADH-quinone reductase subunit D	
CT188	tmk	3578	0.5021614	thymidylate kinase	
CT062	tyrS	6979	0.4995997	tyrosyl-tRNA synthetase	Midlate
CT752	efp_2	3221	0.4992798	elongation factor P	Midlate
CT478	oppC_2	10244	0.4952013	oligopeptide permease	
CT121	araD	3978	0.4908316	ribulose-phosphate 3-epimerase	
CT632		9227	0.4904951	hypothetical protein	
CT186	devB	4473	0.4896205	6-phosphogluconolactonase	
CT606	yggV	3508	0.4891117	putative deoxyribonucleotide triphosphate pyrophosphatase	

CT124	accC	7786	0.4888949	acetyl-CoA carboxylase biotin carboxylase subunit		
CT737		3663	0.4883873	SET domain containing protein		
CT772	ppa	3731	0.4847587	inorganic pyrophosphatase		
CT179		2938	0.4809041	hypothetical protein		
CT658	sfhB	5562	0.4805112	ribosomal large subunit pseudouridine synthase D		
CT447	recJ	9659	0.4798463	ssDNA exonuclease		
CT388		1594	0.4790909	hypothetical protein		
CT064	lepA	10142	0.4775047	GTP-binding protein LepA		
CT191		1970	0.4753534	hypothetical protein		
CT328	tpiA	4418	0.4726208	triosephosphate isomerase		Midlate
CT112	pepF	10253	0.4707544	oligoendopeptidase F		
CT248	glgP	13521	0.4689512	glycogen phosphorylase		Late
CT223		4651	0.4679806	hypothetical protein		
CT568		2448	0.4665883	hypothetical protein		
CT501	ruvA	3233	0.46638	Holliday junction DNA helicase RuvA		
CT712		6330	0.4645087	hypothetical protein	Late	Late
CT683		5259	0.4637615	TPR-motif-containing protein		
CT781	lysS	8514	0.4616821	lysyl-tRNA synthetase		
CT727	zntA	11052	0.461189	metal transport P-type ATPase		
CT141	secA_1	2429	0.4596886	protein translocase		
CT176	dsbB	2209	0.4520519	putative disulfide oxidoreductase		
CT762	murC/ddlA	12791	0.4501852	bifunctional D-alanyl-alanine synthetase A/UDP-N-acetylmuramate--L-alanine ligase		
CT274		2063	0.4459109	hypothetical protein		
CT569		1675	0.4441434	general secretion pathway protein G		
CT558	lipA	4656	0.4407191	lipoyl synthase		
CT254		4008	0.4405389	hypothetical protein		
CT104	fabI	4672	0.4353836	enoyl-(acyl carrier protein) reductase		
CT777	bioF	5756	0.4349837	8-amino-7-oxononanoate synthase		
CT182	kdsB	3946	0.4334897	3-deoxy-manno-octulosonate cytidylyltransferase		
CT435	cysJ	5373	0.4323888	sulfite reductase		
CT562	yscR	4425	0.4295247	type III secretion system protein		
CT343		3159	0.4254714	O-sialoglycoprotein endopeptidase family protein		
CT144		4163	0.4233208	hypothetical protein		Midlate II
CT753		1123	0.4228033	hypothetical protein		
CT380	phnP	3927	0.4219895	metal-dependent hydrolase		
CT564	yyscT	4372	0.420643	Yop proteins translocation protein T		
CT864	xerD	4363	0.4196691	site-specific tyrosine recombinase XerD		Late
CT689	dppF	3841	0.4170842	peptide ABC transporter ATPase		
CT870	pmpF	14606	0.4168276	polymorphic outer membrane protein		Midlate
CT451	cdsA	4619	0.4164956	phosphatidate cytidylyltransferase		
CT494	sohB	4843	0.4159425	protease		Midlate II
CT749	alaS	13424	0.4141237	alanyl-tRNA synthetase		
CT211		2727	0.4138184	hypothetical protein		
CT576	lcrH_1	3385	0.4119391	low calcium response protein H	Late	Late
CT874	pmpI	13112	0.4118268	polymorphic outer membrane protein		Midlate
CT471		2844	0.4086567	hypothetical protein		
CT375		4934	0.4086315	D-amino acid dehydrogenase	Early	Late
CT187	dnaX_1	4041	0.4073285	DNA polymerase III subunit delta'		
CT485	hemH	4536	0.4070157	ferrochelatase		
CT444.1		1000	0.4057652	hypothetical protein		
CT128	adk	3483	0.4052732	adenylate kinase		

CT225		1733	0.4034372	hypothetical protein		
CT462	ispD	2997	0.4030244	2-C-methyl-D-erythritol 4-phosphate cytidylyltransferase		
CT073		4720	0.4026073	OMP [leader (19) peptide]		Late
CT581	thrS	9116	0.4025994	threonyl-tRNA synthetase		
CT203		3498	0.4021381	hypothetical protein		
CT007		4582	0.4012493	hypothetical protein		
CT817	tyrP_1	5601	0.4012117	tyrosine transporter		
CT452	cmk	3001	0.4008297	cytidylate kinase		
CT016		3159	0.4004206	hypothetical protein		Late
CT599	tolB	5863	0.4000129	translocation protein TolB		
CT410	pcnB_1	5799	0.400008	polyA polymerase		
CT210	hemL	5828	0.399743	glutamate-1-semialdehyde aminotransferase		
CT551	dacC	4778	0.3996084	D-alanyl-D-alanine carboxypeptidase		
CT377	ltuA	614	0.3989196	late transcription unit A protein		Midlate
CT547		4159	0.398025	hypothetical protein		
CT393	proS	8323	0.3958995	prolyl-tRNA synthetase		
CT264	msbA	9109	0.3940785	transport ATP binding protein		
CT628	ispA	4074	0.3940433	dimethylallyltransferase		
CT114		6638	0.3921833	hypothetical protein		
CT722	pgm	3035	0.3903935	phosphoglyceromutase		
CT636	greA	9807	0.3873291	transcript cleavage factor/unknown domain fusion protein		
CT453	plsC	2853	0.3854495	1-acyl-sn-glycerol-3-phosphate acyltransferase		
CT306	atpD	2924	0.3806123	V-type ATP synthase subunit D		
CT250	dnaA	6120	0.3801993	chromosomal replication initiation protein		
CT814.1		1653	0.3796641	hypothetical protein	Late	
CT024		3734	0.376916	N5-glutamine S-adenosyl-L-methionine-dependent methyltransferase		
CT660	gyrA_2	6577	0.3768013	DNA topoisomerase IV subunit A	Late	Late
CT103		4060	0.3756602	HAD family phosphatase/phosphatase		
CT038		1543	0.3752529	hypothetical protein		
CT653	yhbG	3182	0.3750792	ABC transporter ATP-binding protein		
CT850		5112	0.3742739	hypothetical protein	Early	
CT425		8319	0.3739003	hypothetical protein		
CT372		5365	0.3736488	hypothetical protein		Midlate
CT546		4054	0.3732836	hypothetical protein	Late	Late
CT829	trmB	2955	0.3706048	tRNA (guanine-N(7)-)-methyltransferase		
CT549	rsbW	1991	0.370141	sigma regulatory factor-histidine kinase		
CT641	ygeD	7251	0.3700856	efflux protein		
CT279	nqr3	4236	0.3698926	Na( )-translocating NADH-quinone reductase subunit C		
CT446	euo	2326	0.3685475	hypothetical protein	Early	
CT034	ytfF	4331	0.3680107	cationic amino acid transporter		
CT673	pkn5	6368	0.3672972	S/T protein kinase		
CT812	pmpD	19558	0.3649884	polymorphic outer membrane protein		
CT574	pepP	4676	0.3645196	aminopeptidase P		
CT019	ileS	13259	0.3629205	isoleucyl-tRNA synthetase		
CT118		2223	0.3614338	inclusion membrane protein G	Early	
CT009	yfgA	1764	0.3581835	HTH transcriptional regulator		
CT782	cysS	6272	0.3578625	cysteinyl-tRNA synthetase		
CT434	ispF	2150	0.3545767	2-C-methyl-D-erythritol 2,4-cyclodiphosphate synthase		
CT245	pdhA	4311	0.3506622	pyruvate dehydrogenase E1 component alpha subunit		
CT450	yaeS	3110	0.3491009	undecaprenyl pyrophosphate synthase		
CT207	pfkA_2	6946	0.3483596	diphosphate--fructose-6-phosphate 1-phosphotransferase		

CT414	pmpC	22572	0.3471195	putative outer membrane protein C		Midlate
CT357		1284	0.3460634	hypothetical protein		
CT307	atpB	5524	0.3459628	V-type ATP synthase subunit B		
CT145		7356	0.3444141	Serine/threonine protein kinase		
CT272	mraW	3662	0.3439276	S-adenosyl-methyltransferase MraW		
CT693	pgk	4542	0.3431838	phosphoglycerate kinase		Late
CT789		996	0.3428773	hypothetical protein		
CT761	murG	4017	0.3405911	undecaprenyldiphospho-muramoylpentapeptide beta-N-acetylglucosaminyltransferase		
CT760	ftsW	4357	0.3398325	cell division protein FtsW		
CT100	acpS	1397	0.3395732	4'-phosphopantetheinyl transferase		
CT596	exbB	2754	0.3369491	polysaccharide transporter		
CT238	fabD	3389	0.3366875	acyl-carrier-protein S-malonyltransferase		Midlate
CT336	ptsI	6533	0.3361421	phosphoenolpyruvate-protein phosphotransferase		
CT302	valS	10741	0.3344971	valyl-tRNA synthetase		
CT865		3770	0.3301376	hypothetical protein		
CT477	ada	2007	0.3288016	methylated-DNA protein - cysteine methyltransferase		
CT751	amn	3127	0.3223967	AMP nucleosidase		
CT697	nth	2193	0.3217049	endonuclease III		
CT825		4751	0.3214358	hypothetical protein		
CT056		2667	0.3208982	hypothetical protein		
CT637	tyrB	4550	0.3202653	aromatic amino acid aminotransferase		
CT499	lplA_2	2712	0.32025	lipoate-protein ligase A		
CT493	polA	9657	0.3196767	DNA polymerase I		Late
CT258	yhfO	4191	0.3191975	NifS family pyridoxal phosphate-dependent protein		
CT134		1609	0.3189933	hypothetical protein		
CT579		5238	0.3170557	hypothetical protein	Late	Late
CT577		1395	0.3167684	hypothetical protein		Late
CT148	mhpA	5656	0.3156369	FAD-dependent monooxygenase		
CT194	mgtE	5461	0.3144538	Mg2 transporter		
CT814		1565	0.3133599	hypothetical protein	Late	Late
CT076	smpB	1610	0.3128356	SsrA-binding protein		
CT394	hrcA	4449	0.3120359	heat-inducible transcription repressor		Late
CT732	ribH	1689	0.3119077	6,7-dimethyl-8-ribityllumazine synthase		
CT379	hflX	5052	0.3119004	GTP binding protein		
CT567		1886	0.3116997	hypothetical protein		
CT084		3993	0.3116736	phopholipase D superfamily protein		Late
CT635		1663	0.3107204	hypothetical protein		Midlate
CT463	truA	2842	0.3101611	tRNA pseudouridine synthase A		
CT138		3509	0.3096179	microsomal dipeptidase		
CT711		8358	0.3088497	hypothetical protein		Late
CT340	pdhA/B	7368	0.308743	2-oxoisovalerate dehydrogenase alpha subunit		
CT374	arcD	5258	0.3073178	arginine/ornithine antiporter		
CT679	tsf	3166	0.3067647	elongation factor Ts		
CT256		4397	0.3059165	hypothetical protein		
CT214		5982	0.3032452	hypothetical protein	Late	Late
CT472		2837	0.3032118	hypothetical protein		
CT400	sucB_2	4006	0.302543	branched-chain alpha-keto acid dehydrogenase subunit E2		
CT263		2118	0.3025216	hypothetical protein		
CT057	gcpE	6487	0.3016625	4-hydroxy-3-methylbut-2-en-1-yl diphosphate synthase		
CT140	ypdP	2480	0.3011226	hypothetical protein		Late
CT748	mfd	11798	0.3003734	transcription-repair coupling factor		

CT467	atoS	3422	0.2982915	2-component regulatory system-sensor histidine kinase		
CT624	mviN	5653	0.2978966	integral membrane protein		
CT545	dnaE	13014	0.2974277	DNA polymerase III subunit alpha		
CT703	engA	5115	0.2971275	GTP-binding protein EngA		
CT217	ydaO	2366	0.2959351	PP-loop superfamily ATPase		
CT688	parB	3096	0.2951474	chromosome partitioning protein		
CT575	mutL	5731	0.29319	DNA mismatch repair protein		
CT497	dnaB	4633	0.2910327	replicative DNA helicase		
CT329	xseA	5105	0.2905075	exodeoxyribonuclease VII large subunit		
CT815	glmM	4487	0.2897044	phosphoglucosamine mutase		
CT115		1440	0.288664	inclusion membrane protein D	Early	
CT262		2596	0.2885514	hypothetical protein		
CT018		1544	0.2869658	hypothetical protein		
CT252	lgt	2571	0.2839016	prolipoprotein diacylglyceryl transferase		
CT845		885	0.2833383	hypothetical protein		
CT335		1010	0.2821186	hypothetical protein		
CT774	cysQ	3095	0.281679	3'(2'),5'-bisphosphate nucleotidase	Early	
CT181		2492	0.2806766	hypothetical protein		Late
CT857		4268	0.2794381	Na /H antiporter		
CT220	ubiX	1818	0.2782167	aromatic acid decarboxylase		
CT848		1652	0.2779484	hypothetical protein		Late
CT458	YhhY	1576	0.2769883	ribosomal-protein-alanine acetyltransferase		Late
CT529		2839	0.2768478	hypothetical protein	Early	
CT063	gnd	4433	0.2765677	6-phosphogluconate dehydrogenase		
CT454	argS	5361	0.2755128	arginyl-tRNA synthetase		
CT684		4603	0.2753095	cysteine desulfurase activator complex subunit SufB		
CT153		7850	0.2745301	MAC/perforin family protein		
CT297	rnc	2272	0.273808	ribonuclease III		
CT738	yycJ	2490	0.2733444	Zn-dependent hydrolase		
CT093	ribF	2842	0.2699016	bifunctional riboflavin kinase/FMN adenylyltransferase		
CT255		1240	0.2689725	hypothetical protein		
CT305	atpI	6006	0.2677309	V-type ATP synthase subunit I		
CT747	hemE	3142	0.2661826	uroporphyrinogen decarboxylase		
CT247	pdhC	4162	0.265296	branched-chain alpha-keto acid dehydrogenase subunit E2		
CT209	leuS	7504	0.2652656	leucyl-tRNA synthetase		
CT108	ybgI	2340	0.2650438	Acr family transporter		
CT055	sucB_1	3607	0.2649814	dihydrolipoamide succinyltransferase		
CT806	ptr	8992	0.2637693	insulinase family/protease III		
CT851	map	2684	0.2633291	methionine aminopeptidase	Early	
CT593.1		535	0.263319	hypothetical protein		
CT360		1857	0.2630741	hypothetical protein		Midlate
CT129	glnP	1905	0.2621251	amino acid ABC transporter ATPase		
CT644	yohI	3112	0.2619993	oxidoreductase		
CT805		4035	0.2588508	hypothetical protein		
CT839		3092	0.2583614	hypothetical protein		
CT765	rsbV_2	1010	0.2582369	anti-sigma F factor antagonist		
CT578		4700	0.2564476	hypothetical protein	Late	Late
CT746	hemN_2	4153	0.2562028	coproporphyrinogen III oxidase		
CT246	pdhB	3059	0.2544424	pyruvate dehydrogenase E1 component beta subunit		
CT390	aspC	3386	0.2543222	L,L-diaminopimelate aminotransferase		
CT726	rodA	3539	0.2537359	rod shape protein		
CT257		3535	0.253551	hypothetical protein		

CT640	recC	8446	0.2524235	exodeoxyribonuclease V gamma chain		
CT571	gspE	4191	0.2516693	general secretion pathway protein E		
CT165		1233	0.250691	hypothetical protein		
CT792	mutS	7366	0.2505168	DNA mismatch repair protein MutS		
CT249		1026	0.2498815	hypothetical protein		Very late
CT423		3011	0.2492884	CBS domain-containing protein		
CT184	yqgF	1382	0.247963	Holliday junction resolvase-like protein		
CT101		1331	0.2477702	hypothetical protein		
CT418	obgE	2759	0.2468727	GTPase ObgE		
CT357R		814	0.2458171	hypothetical protein		
CT718		1432	0.2439438	hypothetical protein		
CT259		1942	0.2433062	PP2C phosphatase family protein		Midlate
CT205	pfkA_1	4677	0.2425351	diphosphate--fructose-6-phosphate 1-phosphotransferase		
CT682	pbpB	9550	0.242075	penicillin-binding protein		
CT594	ycfH	2222	0.2414618	PHP superfamily hydrolase		
CT213	rpiA	1940	0.241378	ribose-5-phosphate isomerase A		Late
CT875		1744	0.2409584	hypothetical protein		Late
CT298	radA	3735	0.2406721	DNA repair protein RadA		
CT204	ybhI	3924	0.2403154	dicarboxylate translocator		
CT232	incB	921	0.2384298	inclusion membrane protein B		
CT015	phoH	3546	0.2383474	ATPase		
CT622		5637	0.2373209	hypothetical protein		Late
CT590		7982	0.2371093	hypothetical protein		Midlate
CT475	pheT	6554	0.236442	phenylalanyl-tRNA synthetase subunit beta		
CT308	atpA	4739	0.2360453	V-type ATP synthase subunit A		
CT200	oppC_1	2311	0.2358847	oligopeptide permease		
CT199	oppB_1	2585	0.235836	oligopeptide permease		
CT389		3314	0.2328829	hypothetical protein		
CT350		4586	0.2325987	hypothetical protein		
CT198	oppA_3	4179	0.2318529	oligopeptide binding protein		
CT135		2861	0.2314952	hypothetical protein		
CT855	fumC	3688	0.2304661	fumarate hydratase		
CT227		1072	0.229581	hypothetical protein		
CT146	ligA	5287	0.2290061	NAD-dependent DNA ligase LigA		
CT288		4660	0.2280746	hypothetical protein	Early	Late
CT197	gcp_1	2673	0.228024	putative DNA-binding/iron metalloprotein/AP endonuclease		
CT495		4218	0.2275143	ADP/ATP translocase	Early	
CT796	glyQ	7949	0.2267664	glycyl-tRNA synthetase		
CT235		1100	0.226229	CRP family transcriptional regulator		
CT838		2950	0.2254509	hypothetical protein		
CT791	uvrC	4633	0.2237993	excinuclease ABC subunit C		
CT794	dnaG	4405	0.2231074	DNA primase		
CT847		1379	0.2226799	hypothetical protein	Late	
CT484		2589	0.2219261	hypothetical protein		
CT417		2234	0.2210668	metal transport protein		
CT130	glnQ	1806	0.2207897	amino acid ABC transporter ATPase		
CT818	tyrP_2	2823	0.2201534	tyrosine transporter		
CT572	gspD	5550	0.2200094	general secretion pathway protein D		
CT650	recA	2749	0.2199874	recombinase A		Midlate
CT676		1316	0.2198088	hypothetical protein		
CT766	miaA	2642	0.2193513	tRNA delta(2)-isopentenylpyrophosphate transferase		
CT035		2042	0.2169937	biotin protein ligase	Early	Very late

CT284		3529	0.2161889	phospholipase D		
CT826	pssA	2022	0.2157327	CDP-diacylglycerol--serine O-phosphatidyltransferase		
CT127	ydhO	2086	0.2155457	polysaccharide hydrolase invasin repeat-containing protein		
CT620		6468	0.2128328	hypothetical protein		Late
CT229		1583	0.211273	hypothetical protein	Early	
CT456		7582	0.2109943	hypothetical protein		Late
CT604	groEL_2	3888	0.2109403	HSP-60		
CT277		1623	0.2095808	hypothetical protein		
CT496.1		318	0.2089303	hypothetical protein		
CT856	ychM	3994	0.2072116	sulfate transporter		
CT616		2947	0.2066732	hypothetical protein		
CT698	trmE	3139	0.2065532	tRNA modification GTPase TrmE		
CT483		889	0.2060415	hypothetical protein		
CT059	fer	613	0.2058454	ferredoxin		
CT685		1808	0.2057169	ABC transporter ATP-binding protein		
CT301	pknD	6482	0.2040098	serine/threonine-protein kinase		
CT566		2408	0.2037279	hypothetical protein		
CT240	recR	1378	0.2027102	recombination protein RecR		
CT331	dxs	4408	0.20156	1-deoxy-D-xylulose-5-phosphate synthase		
CT052	hemN_1	2472	0.1994579	coproporphyrinogen III oxidase		Late
CT206		1937	0.1985244	acyltransferase family protein		
CT183	pyrG	4053	0.1974824	CTP synthetase		
CT767		2362	0.1972917	hypothetical protein		
CT633	hemB	2292	0.1972412	delta-aminolevulinic acid dehydratase		
CT201	oppD	1933	0.1972003	oligopeptide transport ATPase		
CT088	sycE	946	0.1970457	secretion chaperone		
CT677	frr	1291	0.1963105	ribosome recycling factor		
CT221.1		295	0.1962349	hypothetical protein		
CT270	pbp3	4226	0.1947033	penicillin-binding protein		
CT586	uvrB	4497	0.1946438	excinuclease ABC subunit B		
CT387		4533	0.1942975	hypothetical protein		
CT109		1805	0.1939423	hypothetical protein		
CT807	plsB	2316	0.1926208	glycerol-3-phosphate acyltransferase		
CT488		1701	0.1926079	hypothetical protein		
CT844	yfhC	1058	0.1925757	cytosine deaminase		
CT481		1608	0.1923757	hypothetical protein		
CT010	htrB	3066	0.1920098	lipid A biosynthesis lauroyl acyltransferase		
CT573		2563	0.190617	hypothetical protein		
CT384		3503	0.1882578	hypothetical protein		
CT427		1791	0.1877179	hypothetical protein		
CT008	rnhB_1	1939	0.1873852	ribonuclease HIII		
CT714	gpsA	2067	0.1870994	NAD(P)H-dependent glycerol-3-phosphate dehydrogenase		
CT309		1691	0.1869014	hypothetical protein		
CT061	fliA	1632	0.1865393	RNA polymerase sigma factor sigma-28		
CT208	gseA	2466	0.1857514	3-deoxy-D-manno-octulosonic-acid transferase		
CT742	ygcA	2489	0.1839789	rRNA methyltransferase		
CT147		9577	0.1830891	hypothetical protein	Early	Very late
CT846		1387	0.1828684	hypothetical protein		
CT739	ftsK	4818	0.1808686	cell division protein FtsK		
CT580		2001	0.1790066	hypothetical protein		
CT426		2247	0.1745589	hypothetical protein		
CT745	hemG	2503	0.1738093	protoporphyrinogen oxidase		

CT391		2047	0.1728824	hypothetical protein		
CT033	recD_1	4455	0.1721867	exodeoxyribonuclease V alpha chain		
CT854		3535	0.1719491	ABC transporter permease fused to pyrimidine biosynthesis enzyme		
CT152	ycfV	1338	0.1711803	ABC transporter ATPase		
CT687	yfhO_1	2409	0.1707869	cysteine desulfurase		
CT755	groEL_3	2898	0.1706142	60 kDa chaperonin GroEL		
CT837		4162	0.1703334	hypothetical protein		
CT338		868	0.1678384	hypothetical protein		
CT459	prfB	2087	0.1672115	peptide chain release factor 2		Late
CT285	lplA_1	1414	0.1665363	lipoate-protein ligase A		
CT570	gspF	2020	0.1664476	general secretion pathway protein F		
CT346	elaC	1731	0.1659126	ribonuclease Z		Midlate
CT730	ribD	2197	0.1644965	riboflavin deaminase		
CT555		6927	0.1632644	SWI/SNF family helicase		
CT548		1101	0.1621617	hypothetical protein		
CT020	lepB	3533	0.1618725	signal peptidase I		
CT721	yfhO_2	2143	0.1617244	NifS-related protein		
CT809		590	0.1610418	hypothetical protein		
CT164		513	0.1609203	hypothetical protein		
CT595	dsbD	4034	0.1606704	Thio:disulfide interchange protein		
CT489	glgC	2348	0.1571834	glucose-1-phosphate adenylyltransferase		Late
CT171	trpA	1417	0.1565569	tryptophan synthase subunit alpha		
CT415	yebL	1530	0.1564621	solute-binding protein		
CT756	murF	2479	0.1560224	UDP-N-acetylmuramoyl-tripeptide--D-alanyl-D- alanine ligase		
CT534	lnt	2984	0.1559149	apolipoprotein N-acyltransferase		
CT299	hemC	1282	0.1553369	porphobilinogen deaminase		
CT725	birA	905	0.1537204	biotin--protein ligase		
CT325		766	0.1535967	hypothetical protein		
CT863		2595	0.1534407	hypothetical protein		
CT365		3076	0.1529762	hypothetical protein	Early	Late
CT695		2068	0.1505797	hypothetical protein		Late
CT366	aroA	2266	0.1502589	3-phosphoshikimate 1-carboxyvinyltransferase		
CT383		1193	0.1493602	hypothetical protein		
CT867		1788	0.1492081	hypothetical protein	Late	
CT473		484	0.1482725	hypothetical protein	Early	
CT202	oppF	1311	0.147785	oligopeptide transport ATPase		
CT085		2960	0.1475025	hypothetical protein		
CT591	sdhB	1128	0.1464399	succinate dehydrogenase iron-sulfur subunit		
CT609	rpoN	2236	0.14533	RNA polymerase factor sigma-54		
CT642		1424	0.1450648	hypothetical protein		
CT226		865	0.1419387	hypothetical protein		
CT233	incC	842	0.1408246	inclusion membrane protein C		Early
CT382	aroG	1374	0.1408199	3-deoxy-7-phosphoheptulonate synthase		
CT054	sucA	4784	0.140411	2-oxoglutarate dehydrogenase E1 component		
CT720		1286	0.1401839	NifU-related protein		
CT231		2431	0.1398317	sodium-dependent amino acid transporter		
CT862	lcrH_2	939	0.1393661	type III secretion chaperone		
CT167		2061	0.1392746	hypothetical protein		
CT224		691	0.1392554	hypothetical protein		
CT351		3241	0.139119	hypothetical protein		
CT133		1285	0.1385044	rRNA methylase	Early	

CT222		588	0.1380921	hypothetical protein		
CT083		797	0.1371928	hypothetical protein		Late
CT416		1102	0.1360971	ABC transporter ATPase		
CT724		818	0.1360734	hypothetical protein		
CT440		555	0.1356558	hypothetical protein		
CT078	folD	1275	0.1354628	bifunctional 5,10-methylene-tetrahydrofolate dehydrogenase/ 5,10-methylene-tetrahydrofolate cyclohydrolase		
CT699	psdD	1467	0.1353472	phosphatidylserine decarboxylase		
CT487	yhhF	891	0.1337155	methyltransferase		
CT219	ubiA	1377	0.132743	prenyltransferase		
CT778	priA	3461	0.1319877	primosome assembly protein PriA		
CT092	ychF	1639	0.1317055	GTP-dependent nucleic acid-binding protein EngD		
CT754	icc	1233	0.1310967	phosphohydrolase		
CT619		4090	0.1301223	hypothetical protein		Late
CT470	recO	1049	0.1289733	DNA repair protein RecO		
CT831	murB	1154	0.1287518	UDP-N-acetylenolpyruvoylglucosamine reductase		
CT367	aroL	777	0.1282924	shikimate kinase		
CT042	glgX	3011	0.1271869	glycogen hydrolase (debranching)		
CT860		2155	0.1260855	hypothetical protein		
CT605	ybbC	1739	0.1257743	hypothetical protein		
CT840	mesJ	1479	0.1250478	PP-loop superfamily ATPase		Late
CT647		752	0.1247678	hypothetical protein		
CT074	recF	1611	0.1246503	recombination protein F		
CT757	mraY	1455	0.1246252	phospho-N-acetylmuramoyl-pentapeptide- transferase		
CT345		535	0.1242902	hypothetical protein		
CT411	lpxB	2574	0.1220138	lipid-A-disaccharide synthase		
CT119	incA	1094	0.1219109	inclusion membrane protein A		
CT244		1765	0.1212154	hypothetical protein		
CT588	rbsU	2677	0.1211403	sigma regulatory family protein-PP2C phosphatase		
CT079		561	0.121091	hypothetical protein		
CT597	exbD	516	0.1210728	biopolymer transport protein		
CT060	flhA	2459	0.1189491	type III secretion system protein		
CT139	oppA_1	1719	0.1185962	oligopeptide binding protein		
CT077	yojL	1180	0.1184424	hypothetical protein		
CT465		828	0.1171992	hypothetical protein		
CT131		4604	0.1157573	transmembrane protein		
CT193	tgt	1476	0.1153986	queuine tRNA-ribosyltransferase		
CT047		1244	0.1140327	hypothetical protein		
CT175	oppA_2	2040	0.1110075	oligopeptide binding protein permease		Early
CT607	ung	847	0.1100326	uracil-DNA glycosylase		
CT638		1042	0.1095896	rod shape-determining protein MreC		
CT779		776	0.1082689	hypothetical protein		
CT468	atoC	1434	0.108057	2-component regulatory system-ATPase		
CT370	aroDE	1775	0.1063869	bifunctional 3-dehydroquinate dehydratase/shikimate dehydrogenase protein		
CT195		1274	0.1062684	hypothetical protein		
CT441	tsp	2573	0.1058073	tail-specific protease		Late
CT662	hemA	1176	0.1055538	glutamyl-tRNA reductase		Midlate
CT639	recB	3694	0.1055358	exodeoxyribonuclease V beta chain		
CT234		3436	0.1053527	hypothetical protein		
CT648		1438	0.102275	hypothetical protein	Early	
CT168		324	0.1012498	hypothetical protein		

(b)

Gene ID	Gene Symbol	Read Count	RPKM	Gene Name	Belland Classifier	Nicholson Classifier
CT444	omcA	2306	87.7747516	9kDa-cysteine-rich lipoprotein	Late	Late
CT229		2383	38.4263261	hypothetical protein	Early	
CT081		1177	37.4773225	hypothetical protein		Very late
CT603	ahpC	1599	28.5122655	thioredoxin peroxidase		
CT065		3849	26.89672	ADP/ATP translocase	Early	
CT734		1484	22.1086671	hypothetical protein	Early	Midlate
CT617	rpsT	624	22.0062654	30S ribosomal protein S20		
CT786	rpmJ	284	21.9935572	50S ribosomal protein L36		
CT446	euo	1006	19.5368495	hypothetical protein	Early	
CT117		560	17.595967	inclusion membrane protein F	Early	
CT116		661	17.4526724	inclusion membrane protein E	Early	
CT134		702	17.3484556	hypothetical protein		
CT228		908	17.0485483	hypothetical protein	Early	
CT785	rpmH	173	14.5362477	50S ribosomal protein L34		
CT843	rpsO	345	14.4435029	30S ribosomal protein S15		
CT794.1		374	13.5264828	hypothetical protein		
CT500	ndk	569	13.3095475	nucleoside diphosphate kinase		Midlate
CT018		508	13.060744	hypothetical protein		
CT508	rs11	463	12.6846902	30S ribosomal protein S11		
CT602		458	12.0117368	hypothetical protein		
CT787	rpsN	291	11.3135052	30S ribosomal protein S14		
CT524	rpsS	249	11.0926694	30S ribosomal protein S19		
CT802	rpsR	255	10.6373142	30S ribosomal protein S18		
CT801	rpsF	320	10.614898	30S ribosomal protein S6		
CT080	ltuB	281	10.511727	late transcription unit B protein	Late	Late
CT316	rplL	339	10.494848	50S ribosomal protein L7/L12		
CT135		1002	10.1194002	hypothetical protein		
CT022	rpmE2	327	9.9843869	50S ribosomal protein L31 type B		
CT342	rpsU	142	9.8207615	30S ribosomal protein S21		
CT509	rpsM	319	9.3592016	30S ribosomal protein S13		
CT439m	rpsL	303	8.9849504	30S ribosomal protein S12		
CT795		383	8.8247604	hypothetical protein	Early	
CT525	rplB	735	8.7146543	50S ribosomal protein L2		
CT526	rplW	246	8.4948625	50S ribosomal protein L23		
CT834	rpmI	140	8.2391597	50S ribosomal protein L35		
CT569		270	8.1425376	general secretion pathway protein G		
CT784	rnpA	254	7.9963338	ribonuclease P		
CT115		330	7.9944854	inclusion membrane protein D	Early	
CT319	rplK	300	7.7923786	50S ribosomal protein L11		
CT421.1		101	7.7402745	hypothetical protein		
CT317	rplJ	374	7.6551861	50S ribosomal protein L10		
CT523	rplV	188	7.4062165	50S ribosomal protein L22		
CT265	accA	642	7.2790527	acetyl-CoA carboxylase carboxyltransferase subunit alpha		
CT810	rpmF	114	7.1804193	50S ribosomal protein L32		
CT398.1		896	7.1613702	Ribonuclease P RNA		
CT421.2		99	7.0918647	hypothetical protein		
CT438	rs7	291	6.9443567	30S ribosomal protein S7		
CT568		311	6.9425411	hypothetical protein		

CT659		152	6.8134292	hypothetical protein	Late	Late
CT150	rpmG	85	6.7045597	50S ribosomal protein L33		
CT318	rplA	397	6.6948475	50S ribosomal protein L1		
CT518	rplN	210	6.3617879	50S ribosomal protein L14		
CT443	omcB	1039	6.1710133	60kD cysteine-rich outer membrane protein	Late	Late
CT322	tuf	670	6.1607117	elongation factor Tu		
CT026	rpsP	178	6.0953965	30S ribosomal protein S16		
CT125	rplM	256	6.0072041	50S ribosomal protein L13		
CT402	lpxK	698	5.9644665	tetraacyldisaccharide 4'-kinase		
CT513	rplR	193	5.9340593	50S ribosomal protein L18		
CT618		415	5.881029	hypothetical protein		Midlate
CT803	rplI	277	5.84832	50S ribosomal protein L9		
CT519	rpsQ	135	5.6762988	30S ribosomal protein S17		
CT506	rplQ	221	5.6699312	50S ribosomal protein L17		
CT528	rplC	308	5.6467981	50S ribosomal protein L3		
CT507	rpoA	625	5.6401849	DNA-directed RNA polymerase subunit alpha		
CT517	rplX	153	5.6058867	50S ribosomal protein L24		
CT098	rpsA	882	5.5326116	30S ribosomal protein S1		
CT409		710	5.5246412	amino acid permease		
CT647		303	5.4690497	hypothetical protein		
CT147		2156	5.4657397	hypothetical protein	Early	Very late
CT691		346	5.33416	hypothetical protein		
CT565		212	5.2395889	hypothetical protein	Late	Late
CT799	ctc	288	5.1598131	50S ribosomal protein L25/general stress protein Ctc		
CT835	rplT	163	5.0284141	50S ribosomal protein L20		
CT512	rpsE	213	4.9882387	30S ribosomal protein S5		
CT126	rpsI	149	4.9733706	30S ribosomal protein S9		
CT420	rplU	156	4.862071	50S ribosomal protein L21		
CT833	infC	223	4.8054049	translation initiation factor IF-3		
CT326.2		79	4.762166	hypothetical protein		
CT567		239	4.7369079	hypothetical protein		
CT086	rpmB	113	4.6541717	50S ribosomal protein L28		
CT398		334	4.6252794	hypothetical protein		
CT522	rpsC	277	4.5980187	30S ribosomal protein S3		
CT221	yqfU	387	4.5267681	hypothetical protein		
CT511	rplO	188	4.5056723	50S ribosomal protein L15		
CT118		242	4.3247408	inclusion membrane protein G	Early	
CT514	rplF	223	4.3073965	50S ribosomal protein L6		
CT648		493	4.19834	hypothetical protein		
CT516	rplE	189	4.1667211	50S ribosomal protein L5		
CT436	rpsJ	110	4.1026185	30S ribosomal protein S10		
CT249		128	4.0990471	hypothetical protein		Very late
CT573		467	4.0737107	hypothetical protein		
CT527	rplD	274	4.0619628	50S ribosomal protein L4		
CT437	fusA	777	4.0478134	elongation factor G		
CT572	gspD	844	4.0006388	general secretion pathway protein D		
CT626	rpsD	230	3.9992337	30S ribosomal protein S4		
CT520	rpmC	83	3.9330767	50S ribosomal protein L29		
CT377	ltuA	76	3.9194882	late transcription unit A protein		Midlate
CT571	gspE	558	3.9171093	general secretion pathway protein E		
CT832	nusB	180	3.8710798	transcription antitermination protein NusB		
CT515	rpsH	154	3.8421699	30S ribosomal protein S8		

CT341	dnaJ	379	3.7427793	chaperone protein DnaJ		
CT343		192	3.3995728	O-sialoglycoprotein endopeptidase family protein		
CT735	dagA_2	444	3.1230752	Na( )-linked D-alanine glycine permease	Early	
CT111	groES	91	3.082376	co-chaperonin GroES	Early	
CT419	rpmA	66	3.0421696	50S ribosomal protein L27		
CT095	rbfA	113	3.0155688	ribosome-binding factor A		
CT498	gidA	484	3.0051616	tRNA uridine 5-carboxymethylaminomethyl modification enzyme GidA		
CT723	yjbC	224	2.9879276	ribosomal large subunit pseudouridine synthase B		
CT028	rplS	109	2.9638218	50S ribosomal protein L19		
CT775		198	2.9076679	1-acyl-sn-glycerol-3-phosphate acyltransferase		
CT314	rpoC	1098	2.8219162	DNA-directed RNA polymerase subunit beta'		
CT521	rplP	131	2.7961337	50S ribosomal protein L16		
CT169	trpR	82	2.7728044	Trp operon repressor		
CT867		254	2.7474293	hypothetical protein	Late	
CT570	gspF	283	2.7060197	general secretion pathway protein F		
CT327	trpC	150	2.7059324	N-(5'-phosphoribosyl)anthranilate isomerase		
CT809		66	2.6971523	hypothetical protein		
CT529		272	2.6939747	hypothetical protein	Early	
CT566		263	2.6882372	hypothetical protein		
CT110	groEL	384	2.6829135	chaperonin GroEL	Early	Midcycle
CT096	infB	611	2.5912625	translation initiation factor IF-2		
CT097	nusA	270	2.4909793	transcription elongation factor NusA		
CT315	rpoB	812	2.475575	DNA-directed RNA polymerase subunit beta		
CT321	secE	59	2.4504893	preprotein translocase subunit SecE		
CT025	ffh	288	2.4388213	signal recognition particle, subunit FFH/SRP54		
CT510	secY	299	2.4092502	preprotein translocase subunit SecY		
CT401	gltT	315	2.3706737	glutamate symport		
CT842	pnp	442	2.3693412	polynucleotide phosphorylase/polyadenylase		Midlate
CT285	lplA_1	145	2.3525831	lipoate-protein ligase A		
CT136		160	2.304708	lysophospholipase esterase		
CT496	pgsA_1	112	2.2922814	CDP-diacylglycerol--glycerol-3-phosphate 3-phosphatidyltransferase		Midlate
CT788		100	2.2701766	leader (60) peptide-periplasmic		
CT768		342	2.1965764	hypothetical protein		Midlate
CT505	gapA	187	2.1909558	glyceraldehyde-3-phosphate dehydrogenase		Midlate
CT808	cafE	330	2.1492329	ribonuclease E		
CT383		118	2.0917038	hypothetical protein		
CT320	nusG	106	2.051111	transcription antitermination protein NusG		
CT504		135	1.995123	hypothetical protein		Midlate
CT310	atpE	124	1.9894525	V-type ATP synthase subunit E		
CT405	ribC	89	1.9839052	riboflavin synthase subunit alpha		
CT353	def	74	1.9784532	peptide deformylase		
CT027	trmD	197	1.9611041	tRNA (guanine-N(1)-)-methyltransferase/unknown domain fusion protein		
CT292	dut	89	1.9591035	deoxyuridine 5'-triphosphate nucleotidohydrolase		
CT046	hctB	113	1.9300894	histone-like protein 2	Late	Late
CT005		176	1.926978	hypothetical protein		Late
CT288		307	1.9231752	hypothetical protein	Early	Late
CT287	mnmA	201	1.9185056	tRNA-specific 2-thiouridylase MnmA		
CT182	kdsB	142	1.8348909	3-deoxy-manno-octulosonate cytidylyltransferase		
CT836	pheS	182	1.8241992	phenylalanyl-tRNA synthetase subunit alpha		
CT214		278	1.7854991	hypothetical protein	Late	Late
CT800	pth	74	1.764235	peptidyl-tRNA hydrolase		
CT579		223	1.7564259	hypothetical protein	Late	Late

CT495		249	1.7408536	ADP/ATP translocase	Early	
CT017		188	1.7345002	hypothetical protein		Late
CT554	brnQ	204	1.7029149	branched chain amino acid ABC transporter carrier protein		
CT841	ftsH	450	1.6985594	ATP-dependent zinc protease		Late
CT492	coaE	89	1.6979562	dephospho-CoA kinase		Midlate II
CT731	ribA/ribB	202	1.6974322	bifunctional 3,4-dihydroxy-2-butanone 4-phosphate synthase/GTP cyclohydrolase II protein		
CT736	ybcL	93	1.6969612	hypothetical protein		
CT678	pyrH	109	1.6788329	uridylate kinase		
CT029	rnhB	100	1.6384954	ribonuclease HII		
CT359		100	1.6359142	hypothetical protein		
CT066		74	1.565509	hypothetical protein		Midlate
CT133		110	1.5653594	rRNA methylase	Early	
CT180	tauB	94	1.5420385	nitrate/iron ABC transporter ATPase		
CT703	engA	197	1.4893178	GTP-binding protein EngA		
CT284		211	1.4880551	phospholipase D		
CT348	yjjK	204	1.4578815	ABC transporter ATPase		
CT094	truB	98	1.4465584	tRNA pseudouridine synthase B		
CT538		83	1.3874105	hypothetical protein		
CT129	glnP	95	1.3764455	amino acid ABC transporter ATPase		
CT875		154	1.3742533	hypothetical protein		Late
CT776	aas	213	1.3444105	long chain fatty acid--[acyl-carrier-protein] ligase		Late
CT035		94	1.3245183	biotin protein ligase	Early	Very late
CT217	ydaO	70	1.3228236	PP-loop superfamily ATPase		
CT601	papQ	71	1.3158358	invasin repeat-containing phosphatase		
CT364	dapB	83	1.3137126	dihydrodipicolinate reductase		
CT811	plsX	98	1.2130594	putative glycerol-3-phosphate acyltransferase PlsX		
CT429		112	1.2109531	hypothetical protein		
CT313	tal	123	1.2007853	transaldolase B		Midlate
CT264	msbA	229	1.1727573	transport ATP binding protein		
CT543	hisS	109	1.1594402	histidyl-tRNA synthetase		
CT034	ytfF	110	1.1488303	cationic amino acid transporter		
CT820	ftsY	90	1.1437511	cell division protein FtsY		
CT397	vacB	199	1.1199961	exoribonuclease II		
CT445	gltX	138	1.1180688	glutamyl-tRNA synthetase		
CT410	pcnB_1	119	1.107962	polyA polymerase		
CT183	pyrG	147	1.0955577	CTP synthetase		
CT613	folP	116	1.093358	dihydropteroate synthase		
CT422		56	1.0884551	metalloenzyme		
CT857		129	1.0657594	Na /H antiporter		
CT293	accD	104	1.0473762	acetyl-CoA carboxylase subunit beta		

(c)

Gene ID	Gene Symbol	Read Count	RPKM	Gene Name	Belland Classifier	Nicholson Classifier
CT229		2383	38.4263261	hypothetical protein	Early	
CT446	euo	1006	19.5368495	hypothetical protein	Early	
CT117		560	17.595967	inclusion membrane protein F	Early	
CT116		661	17.4526724	inclusion membrane protein E	Early	
CT134		702	17.3484556	hypothetical protein		
CT794.1		374	13.5264828	hypothetical protein		
CT018		508	13.060744	hypothetical protein		
CT135		1002	10.1194002	hypothetical protein		

CT795		383	8.8247604	hypothetical protein	Early	
CT569		270	8.1425376	general secretion pathway protein G		
CT115		330	7.9944854	inclusion membrane protein D	Early	
CT265	accA	642	7.2790527	acetyl-CoA carboxylase carboxyltransferase subunit alpha		
CT568		311	6.9425411	hypothetical protein		
CT618		415	5.881029	hypothetical protein		Midlate
CT409		710	5.5246412	amino acid permease		
CT647		303	5.4690497	hypothetical protein		
CT147		2156	5.4657397	hypothetical protein	Early	Very late
CT326.2		79	4.762166	hypothetical protein		
CT567		239	4.7369079	hypothetical protein		
CT118		242	4.3247408	inclusion membrane protein G	Early	
CT648		493	4.19834	hypothetical protein		
CT249		128	4.0990471	hypothetical protein		Very late
CT573		467	4.0737107	hypothetical protein		
CT572	gspD	844	4.0006388	general secretion pathway protein D		
CT377	ltuA	76	3.9194882	late transcription unit A protein		Midlate
CT571	gspE	558	3.9171093	general secretion pathway protein E		
CT832	nusB	180	3.8710798	transcription antitermination protein NusB		
CT343		192	3.3995728	O-sialoglycoprotein endopeptidase family protein		
CT735	dagA_2	444	3.1230752	Na( )-linked D-alanine glycine permease	Early	
CT723	yjbC	224	2.9879276	ribosomal large subunit pseudouridine synthase B		
CT521	rplP	131	2.7961337	50S ribosomal protein L16		
CT867		254	2.7474293	hypothetical protein	Late	
CT570	gspF	283	2.7060197	general secretion pathway protein F		
CT327	trpC	150	2.7059324	N-(5'-phosphoribosyl)anthranilate isomerase		
CT809		66	2.6971523	hypothetical protein		
CT529		272	2.6939747	hypothetical protein	Early	
CT566		263	2.6882372	hypothetical protein		
CT025	ffh	288	2.4388213	signal recognition particle, subunit FFH/SRP54		
CT401	gltT	315	2.3706737	glutamate symport		
CT285	lplA_1	145	2.3525831	lipoate-protein ligase A		
CT808	cafE	330	2.1492329	ribonuclease E		
CT383		118	2.0917038	hypothetical protein		
CT310	atpE	124	1.9894525	V-type ATP synthase subunit E		
CT027	trmD	197	1.9611041	tRNA (guanine-N(1)-)-methyltransferase/unknown domain fusion protein		
CT005		176	1.926978	hypothetical protein		Late
CT288		307	1.9231752	hypothetical protein	Early	Late
CT287	mnmA	201	1.9185056	tRNA-specific 2-thiouridylase MnmA		
CT182	kdsB	142	1.8348909	3-deoxy-manno-octulosonate cytidylyltransferase		
CT836	pheS	182	1.8241992	phenylalanyl-tRNA synthetase subunit alpha		
CT214		278	1.7854991	hypothetical protein	Late	Late
CT579		223	1.7564259	hypothetical protein	Late	Late
CT495		249	1.7408536	ADP/ATP translocase	Early	
CT841	ftsH	450	1.6985594	ATP-dependent zinc protease		Late
CT731	ribA/ribB	202	1.6974322	bifunctional 3,4-dihydroxy-2-butanone 4-phosphate synthase/GTP cyclohydrolase II protein		
CT029	rnhB	100	1.6384954	ribonuclease HII		
CT133		110	1.5653594	rRNA methylase	Early	

(d)

Gene ID	Gene Symbol	Read Count	RPKM	Gene Name	Belland Classifier	Nicholson Classifier
CT444	omcA	127957	41.3098306	9kDa-cysteine-rich lipoprotein	Late	Late

CT398.1		643636	40.5691459	Ribonuclease P RNA		
CT480.1		73708	38.6236333	hypothetical protein		
CT081		73971	20.2924577	hypothetical protein		Very late
CT843	rpsO	50153	15.5987122	30S ribosomal protein S15		
CT500	ndk	76467	15.3072401	nucleoside diphosphate kinase		Midlate
CT786	rpmJ	20419	12.5496176	50S ribosomal protein L36		
CT565		62310	12.0735255	hypothetical protein	Late	Late
CT267	ihfA	40326	11.3153837	integration host factor alpha-subunit		Midlate
CT802	rpsR	26456	9.4316339	30S ribosomal protein S18		
CT691		71911	8.8706087	hypothetical protein		
CT001		24863	8.5347422	hypothetical protein		Late
CT801	rpsF	29993	7.5920442	30S ribosomal protein S6		
CT460		22915	7.5726935	SWIB (YM74) complex protein		
CT617	rpsT	27018	7.5392159	30S ribosomal protein S20		
CT236	acpP	20443	7.3940661	acyl carrier protein		Late
CT785	rpmH	9254	6.1648024	50S ribosomal protein L34		
CT342	rpsU	12427	5.9863725	30S ribosomal protein S21		
CT787	rpsN	19929	5.6307961	30S ribosomal protein S14		
CT110	groEL	118535	5.6049313	chaperonin GroEL	Early	Midcycle
CT313	tal	60213	5.2089728	transaldolase B		Midlate
CT803	rplI	29363	5.0321246	50S ribosomal protein L9		
CT421.1		9013	4.8485025	hypothetical protein		
CT659		12601	4.6324643	hypothetical protein	Late	
CT666		13854	4.6052756	hypothetical protein		Midlate
CT443	omcB	93722	4.5613878	60kD cysteine-rich outer membrane protein	Late	Late
CT421.2		7988	4.225818	hypothetical protein		
CT665		12322	4.1355825	hypothetical protein		Midlate
CT080	ltuB	13295	4.0063474	late transcription unit B protein	Late	Late
CT508	rs11	18331	3.9853128	30S ribosomal protein S11		
CT022	rpmE2	15024	3.9273089	50S ribosomal protein L31 type B		
CT667		20465	3.9027617	hypothetical protein		Midlate
CT769	ybeB	16012	3.8385283	iojap superfamily protein		
CT784	rnpA	15941	3.7951152	ribonuclease P		
CT439m	rpsL	16299	3.765468	30S ribosomal protein S12		
CT316	rplL	17851	3.7372915	50S ribosomal protein L7/L12		
CT603	ahpC	25986	3.6922763	thioredoxin peroxidase		
CT734		28454	3.6534982	hypothetical protein	Early	Midlate
CT312		15384	3.5870277	ferredoxin		
CT398		32071	3.4770503	hypothetical protein		
CT834	rpmI	7760	3.4706333	50S ribosomal protein L35		
CT111	groES	13068	3.3814939	co-chaperonin GroES	Early	
CT125	rplM	18752	3.372223	50S ribosomal protein L13		
CT798	glgA	55307	3.3212414	glycogen synthase	Late	Midlate II
CT610		25742	3.160319	hypothetical protein		Midlate
CT438	rs7	16920	3.1323866	30S ribosomal protein S7		
CT799	ctc	19657	3.0598038	50S ribosomal protein L25/general stress protein Ctc		
CT538		25296	2.977701	hypothetical protein		
CT436	rpsJ	11541	2.9189718	30S ribosomal protein S10		
CT509	rpsM	12326	2.8410084	30S ribosomal protein S13		
CT043		16792	2.8024988	hypothetical protein		Midlate
CT098	rpsA	57918	2.7880614	30S ribosomal protein S1		
CT322	tuf	39654	2.7333683	elongation factor Tu		

CT431	clpP_1	18178	2.6612342	ATP-dependent Clp protease proteolytic subunit		Midlate
CT492	coaE	18910	2.6478869	dephospho-CoA kinase		Midlate II
CT556		13990	2.6421887	hypothetical protein		
CT842	pnp	65530	2.6250557	polynucleotide phosphorylase/polyadenylase		Midlate
CT524	rpsS	7858	2.616983	30S ribosomal protein S19		
CT525	rplB	25344	2.5759787	50S ribosomal protein L2		
CT526	rplW	10018	2.5756043	50S ribosomal protein L23		
CT743	hctA	12364	2.5526242	histone-like developmental protein	Late	
CT086	rpmB	8004	2.533681	50S ribosomal protein L28		
CT126	rpsI	11841	2.5242464	30S ribosomal protein S9		
CT420	rplU	9496	2.4953092	50S ribosomal protein L21		
CT429		28343	2.449707	hypothetical protein		
CT833	infC	14340	2.4247053	translation initiation factor IF-3		
CT810	rpmF	4904	2.3450274	50S ribosomal protein L32		
CT559	yscJ	26652	2.3395025	Yop proteins translocation lipoprotein J		Midlate
CT790		13678	2.3306351	hypothetical protein		
CT437	fusA	56695	2.2873179	elongation factor G		
CT835	rplT	9723	2.2648471	50S ribosomal protein L20		
CT348	yjjK	42774	2.2642508	ABC transporter ATPase		
CT317	rplJ	14199	2.2620724	50S ribosomal protein L10		
CT783		27295	2.2464743	disulfide bond isomerase	Late	Midlate II
CT066		12552	2.245391	hypothetical protein		Midlate
CT503		14131	2.197132	hypothetical protein		
CT289		29146	2.1952028	hypothetical protein		Midlate
CT770	fabF	31571	2.1720252	3-oxoacyl-(acyl carrier protein) synthase II		Midlate
CT663		9604	2.169652	hypothetical protein		Midlate
CT602		10266	2.1656992	hypothetical protein		
CT706	clpP	15625	2.1351812	ATP-dependent Clp protease proteolytic subunit		
CT319	rplK	10789	2.1327013	50S ribosomal protein L11		
CT655	kdsA	20420	2.1252619	2-dehydro-3-deoxyphosphooctonate aldolase		
CT355		25003	2.1249158	hypothetical protein		
CT496	pgsA_1	12332	2.1034452	CDP-diacylglycerol--glycerol-3-phosphate 3-phosphatidyltransferase		Midlate
CT506	rplQ	10804	2.0885137	50S ribosomal protein L17		
CT613	folP	32485	2.0593882	dihydropteroate synthase		
CT507	rpoA	28260	2.0554132	DNA-directed RNA polymerase subunit alpha		
CT314	rpoC	101160	2.0481621	DNA-directed RNA polymerase subunit beta'		
CT819	yccA	17201	2.035041	transport permease		Midlate
CT292	dut	10150	2.0202431	deoxyuridine 5'-triphosphate nucleotidohydrolase		
CT705	clpX	30160	1.9804951	ATP-dependent protease ATP-binding subunit ClpX		
CT150	rpmG	3497	1.9714612	50S ribosomal protein L33		
CT095	rbfA	8586	1.957213	ribosome-binding factor A		
CT707	tig	31162	1.9523891	trigger factor		
CT382.1		4576	1.9399025	hypothetical protein		
CT242		12065	1.9111798	OmpH-like Outer membrane protein		
CT681	ompA	26486	1.8772277	major outer membrane protein		Midlate II
CT505	gapA	22374	1.8710189	glyceraldehyde-3-phosphate dehydrogenase		Midlate
CT504		19186	1.8621851	hypothetical protein		Midlate
CT671		19248	1.8448947	hypothetical protein		Midlate
CT827	nrdA	66934	1.8041895	ribonucleotide-diphosphate reductase subunit alpha		Midlate
CT709	mreB	22827	1.7965789	rod shape-determining protein MreB		Midlate
CT519	rpsQ	5215	1.7934929	30S ribosomal protein S17		

CT511	rplO	8952	1.7781633	50S ribosomal protein L15		
CT626	rpsD	13328	1.769718	30S ribosomal protein S4		
CT402	lpxK	25131	1.758224	tetraacyldisaccharide 4'-kinase		
CT708		73565	1.7512154	SWF/SNF family helicase		Midlate
CT169	trpR	5995	1.7440721	Trp operon repressor		
CT771		8984	1.7423986	hydrolase/phosphatase-like protein		
CT780		9902	1.7366949	protein disulfide isomerase	Late	
CT692		25736	1.735956	inorganic phosphate transporter		
CT136		14582	1.7323721	lysophospholipase esterase		
CT775		15346	1.7311383	1-acyl-sn-glycerol-3-phosphate acyltransferase		Late
CT067	ytgA	19285	1.7263126	solute protein binding family		
CT381	artJ	15481	1.7256006	arginine binding protein		
CT523	rplV	6267	1.678718	50S ribosomal protein L22		
CT539	trxA	5950	1.6672268	thioredoxin		
CT849.1		3435	1.6661718	hypothetical protein		
CT518	rplN	7009	1.6647633	50S ribosomal protein L14		
CT359		10826	1.6335693	hypothetical protein		
CT269	murE	27455	1.6315372	UDP-N-acetylmuramoylalanyl-D-glutamate--2, 6-diaminopimelate ligase		
CT713	porB	18682	1.6249335	outer membrane protein B		Midlate II
CT407	dksA	7333	1.6126414	dnaK suppressor protein		
CT828	nrdB	19763	1.6079871	ribonucleotide-diphosphate reductase subunit beta		Midlate
CT668		12754	1.607224	hypothetical protein		Midlate
CT318	rplA	13557	1.599722	50S ribosomal protein L1		
CT065		31944	1.5940853	ADP/ATP translocase	Early	
CT068	ytgB_1	14494	1.5713382	rRNA methylase		
CT396	dnaK	39262	1.5631737	molecular chaperone DnaK		Late
CT097	nusA	24379	1.5514612	transcription elongation factor NusA		
CT768		30374	1.5508332	hypothetical protein		Midlate
CT513	rplR	6587	1.5496041	50S ribosomal protein L18		
CT664		43145	1.5306274	FHA domain-containing protein		Midlate
CT611		12728	1.5232445	hypothetical protein		Midlate
CT491	rho	24735	1.52082	transcription termination factor Rho		Midlate II
CT656		5184	1.5200637	hypothetical protein		
CT013	cydA	23762	1.5193675	cytochrome d ubiquinol oxidase subunit I		Midlate
CT702		9089	1.5067171	hypothetical protein		Very late
CT498	gidA	32573	1.4899686	tRNA uridine 5-carboxymethylaminomethyl modification enzyme GidA		
CT520	rpmC	3668	1.4659831	50S ribosomal protein L29		
CT800	pth	9027	1.4655789	peptidyl-tRNA hydrolase		
CT544	uhpC	22947	1.4611911	hexosphosphate transport		Midlate
CT614	folX	6459	1.458779	dihydroneopterin aldolase		
CT341	dnaJ	19346	1.455396	chaperone protein DnaJ		
CT228		10010	1.4414367	hypothetical protein	Early	
CT424	rsbV_1	5684	1.4408667	anti-sigma F factor antagonist		
CT512	rpsE	8292	1.4311387	30S ribosomal protein S5		
CT554	brnQ	20984	1.4190678	branched chain amino acid ABC transporter carrier protein		
CT241	yaeT	40128	1.4146311	OMP85 family membrane protein		Midlate
CT541	mip	12354	1.4021371	peptidyl-prolyl cis-trans isomerase		
CT516	rplE	8744	1.3927382	50S ribosomal protein L5		
CT584		8912	1.3746344	hypothetical protein		Midlate
CT527	rplD	10483	1.3738614	50S ribosomal protein L4		

CT419	rpmA	3704	1.3681151	50S ribosomal protein L27	
CT352		4852	1.3506071	hypothetical protein	
CT386		13814	1.3446012	metal dependent hydrolase	Midlate
CT315	rpoB	62593	1.3394806	DNA-directed RNA polymerase subunit beta	
CT763		6707	1.33786	hypothetical protein	
CT741	yajC	5403	1.3342188	preprotein translocase subunit YajC	
CT430	dapF	12769	1.3072251	diaminopimelate epimerase	Midlate
CT824		43965	1.3024114	zinc metalloprotease	
CT537	yjeE	7033	1.2983311	ATPase or kinase	
CT528	rplC	9377	1.2870041	50S ribosomal protein L3	
CT221	yqfU	13208	1.2866999	hypothetical protein	
CT510	secY	20405	1.2790332	preprotein translocase subunit SecY	
CT561	yscL	9730	1.2751667	type III secretion system protein	Midlate
CT002	gatC	4653	1.2667892	aspartyl/glutamyl-tRNA amidotransferase subunit C	
CT385	ycfF	4888	1.2636651	Hit family hydrolase	Midlate
CT028	rplS	5197	1.2635789	50S ribosomal protein L19	
CT517	rplX	4944	1.260762	50S ribosomal protein L24	
CT026	rpsP	4861	1.2550304	30S ribosomal protein S16	
CT296		6696	1.2533908	hypothetical protein	
CT621		36663	1.2508202	hypothetical protein	Midlate
CT096	infB	40639	1.2503143	translation initiation factor IF-2	
CT788		7254	1.2441536	leader (60) peptide-periplasmic	
CT361	dapA	12236	1.242059	dihydrodipicolinate synthase	
CT405	ribC	8550	1.2357354	riboflavin synthase subunit alpha	
CT701	secA	41278	1.2354756	preprotein translocase subunit SecA	Midlate
CT600	pal	8397	1.2331379	peptidoglycan-associated lipoprotein	Midlate
CT180	tauB	10117	1.2311003	nitrate/iron ABC transporter ATPase	
CT177	dsbG	10154	1.2199798	disulfide bond chaperone	
CT017		19053	1.2116205	hypothetical protein	Late
CT859	ispH	13814	1.2067763	4-hydroxy-3-methylbut-2-enyl diphosphate reductase	
CT243	lpxD	15139	1.2063048	UDP-3-O-[3-hydroxymyristoyl] glucosamine N-acyltransferase	
CT612	folA	6723	1.2057099	dihydrofolate reductase	
CT406	nrdR	6677	1.1991416	transcriptional regulator NrdR	
CT678	pyrH	10330	1.1971297	uridylate kinase	
CT039	dcd	8053	1.1968164	deoxycytidine triphosphate deaminase	
CT482		9093	1.19145	hypothetical protein	
CT323	infA	3171	1.1861734	translation initiation factor IF-1	
CT041		10990	1.1817881	hypothetical protein	Late
CT736	ybcL	6083	1.1795911	hypothetical protein	
CT515	rpsH	5266	1.1688084	30S ribosomal protein S8	
CT514	rplF	7425	1.1655267	50S ribosomal protein L6	
CT351a	secG	4203	1.1609324	preprotein translocase subunit SecG	
CT275	dnaA	18449	1.1588853	chromosomal replication initiation protein	
CT858		25646	1.146175	protease	
CT353	def	7131	1.1425044	peptide deformylase	
CT550		5688	1.1359986	hypothetical protein	Midlate
CT672	fliN	15008	1.1273801	type III secretion system protein	Midlate
CT582	minD	9935	1.1060616	chromosome partitioning ATPase-CHLTR plasmid protein homolog GP5D	
CT522	rpsC	8196	1.0991597	30S ribosomal protein S3	
CT321	secE	3168	1.0987296	preprotein translocase subunit SecE	
CT364	dapB	9725	1.0956429	dihydrodipicolinate reductase	

CT670		6527	1.0825593	hypothetical protein		Midlate
CT031		3872	1.0804687	hypothetical protein		
CT543	hisS	15751	1.0767884	histidyl-tRNA synthetase		
CT012	ybbP	9956	1.0632189	hypothetical protein		
CT294	sodM	7590	1.0585806	superoxide dismutase		
CT533	lpxC	10731	1.0577092	UDP-3-O-[3-hydroxymyristoyl] N-acetylglucosamine deacetylase		
CT094	truB	8753	1.0539123	tRNA pseudouridine synthase B		
CT253		8157	1.041969	hypothetical protein		
CT046	hctB	8006	1.0412061	histone-like protein 2	Late	Late
CT069	ytgC	16666	1.0305912	integral membrane protein		
CT090	lcrD	26550	1.0293166	low calcium response D		Midlate
CT674	yscC	34454	1.024511	Yop proteins translocation protein C/general secretion pathway protein		Midlate II
CT320	nusG	6491	1.0224418	transcription antitermination protein NusG		
CT070	ytgD	11759	1.0208357	integral membrane protein		
CT291	ptsN_2	5515	1.0153351	PTS IIA protein		
CT535	yciA	5518	1.0032389	acyl-CoA hydrolase		Midlate
CT044	ssb	5479	0.9904083	single-stranded DNA-binding protein		
CT327	trpC	7187	0.9840054	N-(5'-phosphoribosyl)anthranilate isomerase		
CT476		11122	0.9823909	hypothetical protein		Midlate
CT821	sucC	12691	0.9762476	succinyl-CoA synthetase subunit beta		
CT027	trmD	11482	0.96954	tRNA (guanine-N(1)-)-methyltransferase/unknown domain fusion protein		
CT502	ruvC	5580	0.9694396	Holliday junction resolvase		
CT557	lpdA	15592	0.9681899	dihydrolipoamide dehydrogenase		
CT251		26994	0.9536828	putative inner membrane protein translocase component YidC		
CT540	yibK	5075	0.9465289	rRNA methylase		
CT532	fabZ	5043	0.9388933	(3R)-hydroxymyristoyl-ACP dehydratase		
CT521	rplP	4418	0.9382922	50S ribosomal protein L16		
CT268	amiA	8178	0.9377575	N-acetylmuramoyl-L-alanine amidase		
CT362	lysC	13835	0.9376548	aspartate kinase		
CT189	gyrA_1	28606	0.9348411	DNA gyrase subunit A		
CT282	gcsH	3820	0.9194834	glycine cleavage system protein H		
CT273		6007	0.9185747	hypothetical protein		Midlate
CT700		15172	0.9137172	hypothetical protein		Midlate
CT872	pmpH	33051	0.912142	polymorphic outer membrane protein	Late	Midlate
CT295	mrsA_1	19168	0.9095698	phosphoglucomutase		
CT354	ksgA	8424	0.9040696	dimethyladenosine transferase		
CT740	dmpP	13855	0.9024917	Na( )-translocating NADH-quinone reductase subunit F		
CT266		12514	0.8951058	hypothetical protein		
CT836	pheS	10563	0.8913559	phenylalanyl-tRNA synthetase subunit alpha		
CT218	surE	8721	0.8879608	stationary phase survival protein SurE		Midlate
CT669	yscN	13889	0.8864376	type III secretion system ATPase		Midlate
CT461	yaeI	9943	0.8823883	phosphohydrolase		
CT278	nqr2	15927	0.868281	Na( )-translocating NADH-quinone reductase subunit B		Midlate
CT142		8598	0.867902	hypothetical protein		Midlate II
CT563	yscS	2881	0.8626409	Yop proteins translocation protein S		
CT480	oppA_4	20784	0.8541989	oligopeptide binding lipoprotein	Early	Midlate
CT728		7705	0.8515256	hypothetical protein		
CT618		7634	0.850625	hypothetical protein		Midlate
CT823	htrA	14896	0.8489177	DO Serine protease		
CT029	rnhB	6397	0.8433331	ribonuclease HII		

CT395	grpE	5975	0.8398377	HSP-70 cofactor		Late
CT283		20733	0.8392204	hypothetical protein		
CT531	lpxA	8592	0.8391524	UDP-N-acetylglucosamine acyltransferase		Midlate
CT841	ftsH	27925	0.8387935	ATP-dependent zinc protease		Late
CT759	nlpD	7179	0.8331568	muramidase		
CT652.1		1788	0.8330283	hypothetical protein		
CT457	yebC	6588	0.8321948	hypothetical protein		Late
CT675	karG	10730	0.8303431	ATP:guanido phosphotransferase		
CT710	pckA	15918	0.8270178	phosphoenolpyruvate carboxykinase		Midlate
CT071	yaeM	10984	0.8255771	1-deoxy-D-xylulose 5-phosphate reductoisomerase		
CT587	eno	12754	0.8210394	phosphopyruvate hydratase		
CT117		2974	0.8148149	inclusion membrane protein F	Early	
CT832	nusB	4738	0.8066813	transcription antitermination protein NusB		
CT376	mdhC	9064	0.8018162	malate dehydrogenase	Early	Midlate
CT585	trpS	9791	0.8008935	tryptophanyl-tRNA synthetase		
CT014	cydB	10078	0.8007174	cytochrome d ubiquinol oxidase subunit II		Midlate
CT773	ldh	9443	0.800319	leucine dehydrogenase		
CT025	ffh	12260	0.7985362	signal recognition particle, subunit FFH/SRP54		
CT397	vacB	18971	0.7968309	exoribonuclease II		
CT486	fliY	7469	0.7947251	glutamine-binding protein		
CT542	aspS	15770	0.7903122	aspartyl-tRNA synthetase		
CT875		11136	0.7901522	hypothetical protein		Late
CT286	clpC	25165	0.7893601	ClpC protease ATPase		Midlate II
CT378	pgi	14454	0.7854645	glucose-6-phosphate isomerase		Midlate
CT601	papQ	5643	0.7845385	invasin repeat-containing phosphatase		
CT795		4731	0.7844806	hypothetical protein	Early	
CT349	maf	5394	0.782514	Maf-like protein		
CT871	pmpG	27645	0.7725738	polymorphic outer membrane protein		Midlate
CT330		2448	0.7701522	hypothetical protein		
CT113	clpB	23628	0.7697211	Clp protease ATPase		
CT606.1		2022	0.7680308	hypothetical protein		
CT536	dnaQ_2	6877	0.755695	DNA polymerase III subunit epsilon		
CT215	dhnA	9436	0.7493686	fructose-bisphosphate aldolase		Midlate
CT401	gltT	10188	0.7492583	glutamate symport		
CT023	prfA	9631	0.7473857	peptide chain release factor 1		
CT265	accA	8748	0.7431151	acetyl-CoA carboxylase carboxyltransferase subunit alpha		
CT404		7043	0.7423473	N6-adenine-specific DNA methylase		
CT776	aas	13814	0.7354244	long chain fatty acid--[acyl-carrier-protein] ligase		Late
CT849		3954	0.7340779	hypothetical protein		
CT422		4102	0.7297329	metalloenzyme		
CT625	nfo	7106	0.7290546	endonuclease IV		
CT122	efp_1	4787	0.7276601	elongation factor P		
CT630	cpxR	5609	0.7228291	HTH transcriptional regulatory protein and receiver domain		
CT657		2761	0.72082	hypothetical protein		
CT750	tktB	16109	0.7170629	transketolase		
CT733		11091	0.7126125	hypothetical protein		Late
CT072	yaeL	15073	0.7059336	metalloprotease		
CT239	fabH	7402	0.7006368	3-oxoacyl-(acyl carrier protein) synthase III		Midlate
CT455	murA	10427	0.6933554	UDP-N-acetylglucosamine 1-carboxyvinyltransferase		Midlate
CT344	lon	19769	0.6918818	ATP-dependent protease La		Midlate
CT421		5722	0.6909607	hypothetical protein		
CT479	oppB_2	12021	0.6888977	oligopeptide permease		

CT237	fabG	5803	0.6870492	3-ketoacyl-(acyl-carrier-protein) reductase		Midlate
CT003	gatA	12398	0.6869712	aspartyl/glutamyl-tRNA amidotransferase subunit A		
CT822	sucD	6586	0.6836571	succinyl-CoA synthetase subunit alpha		
CT212		3573	0.6834509	hypothetical protein		Late
CT797	pgsA_2	4700	0.6830251	CDP-diacylglycerol--glycerol-3-phosphate 3-phosphatidyltransferase		
CT260		4034	0.6735139	hypothetical protein		
CT627	yceA	8230	0.6725201	hypothetical protein		
CT190	gyrB	19288	0.6690327	DNA gyrase subunit B		
CT053		3555	0.6679867	hypothetical protein		Midlate
CT116		3172	0.6639256	inclusion membrane protein E	Early	
CT030	gmk	4928	0.6615267	guanylate kinase		
CT432	glyA	11340	0.6573544	serine hydroxymethyltransferase		Midlate
CT804	ychB	6647	0.6563911	4-diphosphocytidyl-2-C-methyl-D-erythritol kinase		
CT303		3980	0.6560009	hypothetical protein		
CT654		3709	0.6558302	hypothetical protein		
CT408	lspA	3720	0.6512336	lipoprotein signal peptidase		Midlate
CT560		6169	0.6500661	hypothetical protein		
CT185	zwf	11936	0.6426318	glucose-6-phosphate 1-dehydrogenase		
CT358		3829	0.6343157	hypothetical protein		
CT704	pcnB_2	9144	0.6329503	polyA polymerase		
CT735	dagA_2	9486	0.6305126	Na( )-linked D-alanine glycine permease	Early	
CT623		10090	0.628459	hypothetical protein		
CT276		4132	0.6281464	hypothetical protein		
CT004	gatB	11233	0.6269354	aspartyl/glutamyl-tRNA amidotransferase subunit B		
CT820	ftsY	6232	0.6266811	cell division protein FtsY		
CT758	murD	9220	0.6240406	UDP-N-acetylmuramoyl-L-alanyl-D-glutamate synthetase		
CT089	lcrE	9325	0.6221447	low calcium response E		Midlate
CT363	asd	7327	0.6218461	aspartate-semialdehyde dehydrogenase		
CT448	secD/secF	30554	0.6211635	bifunctional preprotein translocase subunit SecD/SecF		
CT399	yrbH	7129	0.6210097	GutQ/KpsF family sugar-P isomerase		
CT326.2		1309	0.6200574	hypothetical protein		
CT445	gltX	11370	0.6196809	glutamyl-tRNA synthetase		
CT290	ptsN_1	4700	0.6191686	PTS IIA protein HTH DNA-binding domain		
CT403	yjfH	5672	0.6174237	rRNA methylase		
CT413	pmpB	37899	0.6159743	putative outer membrane protein B		Midlate
CT304	atpK	3006	0.6126116	V-type ATP synthase subunit K		
CT040	ruvB	6919	0.6120047	Holliday junction DNA helicase RuvB		Late
CT634		9651	0.6039711	Na( )-translocating NADH-quinone reductase subunit A		Midlate
CT137	ywlC	5839	0.6024462	SuA5 superfamily-related protein		
CT075	dnaN	9062	0.60223	DNA polymerase III subunit beta		
CT216	xasA	10208	0.5956258	glutamate/gamma-aminobutyrate antiporter		Midlate
CT332	pykF	9902	0.5916113	pyruvate kinase		
CT281	nqr5	5145	0.5910161	Na( )-translocating NADH-quinone reductase subunit E		
CT337	ptsH	2274	0.5882068	phosphocarrier protein HPr		
CT143		5720	0.5873744	hypothetical protein		Midlate II
CT271		1849	0.5865432	hypothetical protein		
CT311		4748	0.583206	hypothetical protein		
CT310	atpE	4234	0.5830783	V-type ATP synthase subunit E		
CT091	yscU	7200	0.582205	type III secretion system protein		
CT583	gp6D	5289	0.5820642	virulence plasmid protein pGP6-D-related protein		
CT608	uvrD	13326	0.5814687	DNA helicase		

CT631		1607	0.5692171	hypothetical protein	
CT808	cafE	10571	0.5691118	ribonuclease E	
CT811	plsX	6551	0.5675709	putative glycerol-3-phosphate acyltransferase PlsX	
CT816	glmS	12201	0.564756	glucosamine--fructose-6-phosphate aminotransferase	Midlate
CT719	fliF	6217	0.5641287	type III secretion system protein	
CT409		8768	0.557485	amino acid permease	
CT032	metG	11386	0.5563218	methionyl-tRNA synthetase	
CT045	pepA	9917	0.5562577	leucyl aminopeptidase	Midlate
CT178		7697	0.5548816	hypothetical protein	
CT615	rpoD	11485	0.5513154	RNA polymerase sigma factor	
CT869	pmpE	19054	0.5502338	polymorphic outer membrane protein	Late
CT428	ubiE	4407	0.5484824	ubiquinone/menaquinone biosynthesis methyltransferase	
CT261	dnaQ_1	4434	0.5477207	DNA polymerase III subunit epsilon	
CT123	accB	3185	0.545436	acetyl-CoA carboxylase biotin carboxyl carrier protein subunit	
CT151		9724	0.5444222	hypothetical protein	
CT466		2039	0.5442333	hypothetical protein	
CT102		2958	0.5439093	hypothetical protein	
CT729	serS	8144	0.5378491	seryl-tRNA synthetase	
CT373		3764	0.5377769	hypothetical protein	Midlate
CT866	glgB	13654	0.5363302	glycogen branching enzyme	
CT731	ribA/ribB	7970	0.5349674	bifunctional 3,4-dihydroxy-2-butanone 4-phosphate synthase/GTP cyclohydrolase II protein	
CT690	dppD	5900	0.5337531	peptide ABC transporter ATPase	
CT099	trxB	6736	0.5329169	thioredoxin reductase	
CT853		3792	0.5274442	hypothetical protein	
CT530	fmt	5952	0.5266046	methionyl-tRNA formyltransferase	
CT011		7988	0.5262418	hypothetical protein	
CT464		4090	0.5223911	hydrolase, haloacid dehalogenase-like family	
CT696		7234	0.5221324	hypothetical protein	
CT287	mnmA	6673	0.5199122	tRNA-specific 2-thiouridylase MnmA	
CT293	accD	5608	0.5186432	acetyl-CoA carboxylase subunit beta	
CT629	glmU	3734	0.5146657	glucosamine-1-phosphate acetyltransferase	
CT643	topA	15566	0.5110588	DNA topoisomerase I/SWI domain fusion protein	Late
CT490		1539	0.5073052	hypothetical protein	
CT280	nqr4	3945	0.5060098	Na( )-translocating NADH-quinone reductase subunit D	
CT188	tmk	3578	0.5021614	thymidylate kinase	
CT062	tyrS	6979	0.4995997	tyrosyl-tRNA synthetase	Midlate
CT752	efp_2	3221	0.4992798	elongation factor P	Midlate
CT478	oppC_2	10244	0.4952013	oligopeptide permease	
CT121	araD	3978	0.4908316	ribulose-phosphate 3-epimerase	
CT632		9227	0.4904951	hypothetical protein	
CT186	devB	4473	0.4896205	6-phosphogluconolactonase	
CT606	yggV	3508	0.4891117	putative deoxyribonucleotide triphosphate pyrophosphatase	
CT124	accC	7786	0.4888949	acetyl-CoA carboxylase biotin carboxylase subunit	
CT737		3663	0.4883873	SET domain containing protein	
CT772	ppa	3731	0.4847587	inorganic pyrophosphatase	
CT179		2938	0.4809041	hypothetical protein	
CT658	sfhB	5562	0.4805112	ribosomal large subunit pseudouridine synthase D	
CT447	recJ	9659	0.4798463	ssDNA exonuclease	
CT388		1594	0.4790909	hypothetical protein	
CT064	lepA	10142	0.4775047	GTP-binding protein LepA	
CT191		1970	0.4753534	hypothetical protein	

CT328	tpiA	4418	0.4726208	triosephosphate isomerase		Midlate
CT112	pepF	10253	0.4707544	oligoendopeptidase F		
CT248	glgP	13521	0.4689512	glycogen phosphorylase		Late
CT223		4651	0.4679806	hypothetical protein		
CT568		2448	0.4665883	hypothetical protein		
CT501	ruvA	3233	0.46638	Holliday junction DNA helicase RuvA		
CT712		6330	0.4645087	hypothetical protein	Late	Late
CT683		5259	0.4637615	TPR-motif-containing protein		
CT781	lysS	8514	0.4616821	lysyl-tRNA synthetase		
CT727	zntA	11052	0.461189	metal transport P-type ATPase		
CT141	secA_1	2429	0.4596886	protein translocase		
CT176	dsbB	2209	0.4520519	putative disulfide oxidoreductase		
CT762	murC/ddlA	12791	0.4501852	bifunctional D-alanyl-alanine synthetase A/UDP-N-acetylmuramate--L-alanine ligase		
CT274		2063	0.4459109	hypothetical protein		
CT569		1675	0.4441434	general secretion pathway protein G		
CT558	lipA	4656	0.4407191	lipoyl synthase		
CT254		4008	0.4405389	hypothetical protein		
CT104	fabI	4672	0.4353836	enoyl-(acyl carrier protein) reductase		
CT777	bioF	5756	0.4349837	8-amino-7-oxononanoate synthase		
CT182	kdsB	3946	0.4334897	3-deoxy-manno-octulosonate cytidylyltransferase		
CT435	cysJ	5373	0.4323888	sulfite reductase		
CT562	yscR	4425	0.4295247	type III secretion system protein		
CT343		3159	0.4254714	O-sialoglycoprotein endopeptidase family protein		
CT144		4163	0.4233208	hypothetical protein		Midlate II
CT753		1123	0.4228033	hypothetical protein		
CT380	phnP	3927	0.4219895	metal-dependent hydrolase		
CT564	yyscT	4372	0.420643	Yop proteins translocation protein T		
CT864	xerD	4363	0.4196691	site-specific tyrosine recombinase XerD		Late
CT689	dppF	3841	0.4170842	peptide ABC transporter ATPase		
CT870	pmpF	14606	0.4168276	polymorphic outer membrane protein		Midlate
CT451	cdsA	4619	0.4164956	phosphatidate cytidylyltransferase		
CT494	sohB	4843	0.4159425	protease		Midlate II
CT749	alaS	13424	0.4141237	alanyl-tRNA synthetase		
CT211		2727	0.4138184	hypothetical protein		
CT576	lcrH_1	3385	0.4119391	low calcium response protein H	Late	Late
CT874	pmpI	13112	0.4118268	polymorphic outer membrane protein		Midlate
CT471		2844	0.4086567	hypothetical protein		
CT375		4934	0.4086315	D-amino acid dehydrogenase	Early	Late
CT187	dnaX_1	4041	0.4073285	DNA polymerase III subunit delta'		
CT485	hemH	4536	0.4070157	ferrochelatase		
CT444.1		1000	0.4057652	hypothetical protein		
CT128	adk	3483	0.4052732	adenylate kinase		
CT225		1733	0.4034372	hypothetical protein		
CT462	ispD	2997	0.4030244	2-C-methyl-D-erythritol 4-phosphate cytidylyltransferase		
CT073		4720	0.4026073	OMP [leader (19) peptide]		Late
CT581	thrS	9116	0.4025994	threonyl-tRNA synthetase		
CT203		3498	0.4021381	hypothetical protein		
CT007		4582	0.4012493	hypothetical protein		
CT817	tyrP_1	5601	0.4012117	tyrosine transporter		
CT452	cmk	3001	0.4008297	cytidylate kinase		
CT016		3159	0.4004206	hypothetical protein		Late

CT599	tolB	5863	0.4000129	translocation protein TolB		
CT410	pcnB_1	5799	0.400008	polyA polymerase		
CT210	hemL	5828	0.399743	glutamate-1-semialdehyde aminotransferase		
CT551	dacC	4778	0.3996084	D-alanyl-D-alanine carboxypeptidase		
CT377	ltuA	614	0.3989196	late transcription unit A protein		Midlate
CT547		4159	0.398025	hypothetical protein		
CT393	proS	8323	0.3958995	prolyl-tRNA synthetase		
CT264	msbA	9109	0.3940785	transport ATP binding protein		
CT628	ispA	4074	0.3940433	dimethylallyltransferase		
CT114		6638	0.3921833	hypothetical protein		
CT722	pgm	3035	0.3903935	phosphoglyceromutase		
CT636	greA	9807	0.3873291	transcript cleavage factor/unknown domain fusion protein		
CT453	plsC	2853	0.3854495	1-acyl-sn-glycerol-3-phosphate acyltransferase		
CT306	atpD	2924	0.3806123	V-type ATP synthase subunit D		
CT250	dnaA	6120	0.3801993	chromosomal replication initiation protein		
CT814.1		1653	0.3796641	hypothetical protein	Late	
CT024		3734	0.376916	N5-glutamine S-adenosyl-L-methionine-dependent methyltransferase		
CT660	gyrA_2	6577	0.3768013	DNA topoisomerase IV subunit A	Late	Late
CT103		4060	0.3756602	HAD family phosphatase/phosphatase		
CT038		1543	0.3752529	hypothetical protein		
CT653	yhbG	3182	0.3750792	ABC transporter ATP-binding protein		
CT850		5112	0.3742739	hypothetical protein	Early	
CT425		8319	0.3739003	hypothetical protein		
CT372		5365	0.3736488	hypothetical protein		Midlate
CT546		4054	0.3732836	hypothetical protein	Late	Late
CT829	trmB	2955	0.3706048	tRNA (guanine-N(7)-)-methyltransferase		
CT549	rsbW	1991	0.370141	sigma regulatory factor-histidine kinase		
CT641	ygeD	7251	0.3700856	efflux protein		
CT279	nqr3	4236	0.3698926	Na( )-translocating NADH-quinone reductase subunit C		
CT446	euo	2326	0.3685475	hypothetical protein	Early	
CT034	ytfF	4331	0.3680107	cationic amino acid transporter		
CT673	pkn5	6368	0.3672972	S/T protein kinase		
CT812	pmpD	19558	0.3649884	polymorphic outer membrane protein		
CT574	pepP	4676	0.3645196	aminopeptidase P		
CT019	ileS	13259	0.3629205	isoleucyl-tRNA synthetase		
CT118		2223	0.3614338	inclusion membrane protein G	Early	
CT009	yfgA	1764	0.3581835	HTH transcriptional regulator		
CT782	cysS	6272	0.3578625	cysteinyl-tRNA synthetase		
CT434	ispF	2150	0.3545767	2-C-methyl-D-erythritol 2,4-cyclodiphosphate synthase		
CT245	pdhA	4311	0.3506622	pyruvate dehydrogenase E1 component alpha subunit		
CT450	yaeS	3110	0.3491009	undecaprenyl pyrophosphate synthase		
CT207	pfkA_2	6946	0.3483596	diphosphate--fructose-6-phosphate 1-phosphotransferase		
CT414	pmpC	22572	0.3471195	putative outer membrane protein C		Midlate
CT357		1284	0.3460634	hypothetical protein		
CT307	atpB	5524	0.3459628	V-type ATP synthase subunit B		
CT145		7356	0.3444141	Serine/threonine protein kinase		
CT272	mraW	3662	0.3439276	S-adenosyl-methyltransferase MraW		
CT693	pgk	4542	0.3431838	phosphoglycerate kinase		Late
CT789		996	0.3428773	hypothetical protein		
CT761	murG	4017	0.3405911	undecaprenyldiphospho-muramoylpentapeptide beta-N-acetylglucosaminyltransferase		

CT760	ftsW	4357	0.3398325	cell division protein FtsW		
CT100	acpS	1397	0.3395732	4'-phosphopantetheinyl transferase		
CT596	exbB	2754	0.3369491	polysaccharide transporter		
CT238	fabD	3389	0.3366875	acyl-carrier-protein S-malonyltransferase		Midlate
CT336	ptsI	6533	0.3361421	phosphoenolpyruvate-protein phosphotransferase		
CT302	valS	10741	0.3344971	valyl-tRNA synthetase		
CT865		3770	0.3301376	hypothetical protein		
CT477	ada	2007	0.3288016	methylated-DNA protein - cysteine methyltransferase		
CT751	amn	3127	0.3223967	AMP nucleosidase		
CT697	nth	2193	0.3217049	endonuclease III		
CT825		4751	0.3214358	hypothetical protein		
CT056		2667	0.3208982	hypothetical protein		
CT637	tyrB	4550	0.3202653	aromatic amino acid aminotransferase		
CT499	lplA_2	2712	0.32025	lipoate-protein ligase A		
CT493	polA	9657	0.3196767	DNA polymerase I		Late
CT258	yhfO	4191	0.3191975	NifS family pyridoxal phosphate-dependent protein		
CT134		1609	0.3189933	hypothetical protein		
CT579		5238	0.3170557	hypothetical protein	Late	Late
CT577		1395	0.3167684	hypothetical protein		Late
CT148	mhpA	5656	0.3156369	FAD-dependent monooxygenase		
CT194	mgtE	5461	0.3144538	Mg2 transporter		
CT814		1565	0.3133599	hypothetical protein	Late	Late
CT076	smpB	1610	0.3128356	SsrA-binding protein		
CT394	hrcA	4449	0.3120359	heat-inducible transcription repressor		Late
CT732	ribH	1689	0.3119077	6,7-dimethyl-8-ribityllumazine synthase		
CT379	hflX	5052	0.3119004	GTP binding protein		
CT567		1886	0.3116997	hypothetical protein		
CT084		3993	0.3116736	phopholipase D superfamily protein		Late
CT635		1663	0.3107204	hypothetical protein		Midlate
CT463	truA	2842	0.3101611	tRNA pseudouridine synthase A		
CT138		3509	0.3096179	microsomal dipeptidase		
CT711		8358	0.3088497	hypothetical protein		Late
CT340	pdhA/B	7368	0.308743	2-oxoisovalerate dehydrogenase alpha subunit		
CT374	arcD	5258	0.3073178	arginine/ornithine antiporter		
CT679	tsf	3166	0.3067647	elongation factor Ts		
CT256		4397	0.3059165	hypothetical protein		
CT214		5982	0.3032452	hypothetical protein	Late	Late
CT472		2837	0.3032118	hypothetical protein		
CT400	sucB_2	4006	0.302543	branched-chain alpha-keto acid dehydrogenase subunit E2		
CT263		2118	0.3025216	hypothetical protein		
CT057	gcpE	6487	0.3016625	4-hydroxy-3-methylbut-2-en-1-yl diphosphate synthase		
CT140	ypdP	2480	0.3011226	hypothetical protein		Late
CT748	mfd	11798	0.3003734	transcription-repair coupling factor		
CT467	atoS	3422	0.2982915	2-component regulatory system-sensor histidine kinase		
CT624	mviN	5653	0.2978966	integral membrane protein		
CT545	dnaE	13014	0.2974277	DNA polymerase III subunit alpha		
CT703	engA	5115	0.2971275	GTP-binding protein EngA		
CT217	ydaO	2366	0.2959351	PP-loop superfamily ATPase		
CT688	parB	3096	0.2951474	chromosome partitioning protein		
CT575	mutL	5731	0.29319	DNA mismatch repair protein		
CT497	dnaB	4633	0.2910327	replicative DNA helicase		
CT329	xseA	5105	0.2905075	exodeoxyribonuclease VII large subunit		

CT815	glmM	4487	0.2897044	phosphoglucosamine mutase		
CT115		1440	0.288664	inclusion membrane protein D	Early	
CT262		2596	0.2885514	hypothetical protein		
CT018		1544	0.2869658	hypothetical protein		
CT252	lgt	2571	0.2839016	prolipoprotein diacylglyceryl transferase		
CT845		885	0.2833383	hypothetical protein		
CT335		1010	0.2821186	hypothetical protein		
CT774	cysQ	3095	0.281679	3'(2'),5'-bisphosphate nucleotidase	Early	
CT181		2492	0.2806766	hypothetical protein		Late
CT857		4268	0.2794381	Na /H antiporter		
CT220	ubiX	1818	0.2782167	aromatic acid decarboxylase		
CT848		1652	0.2779484	hypothetical protein		Late
CT458	YhhY	1576	0.2769883	ribosomal-protein-alanine acetyltransferase		Late
CT529		2839	0.2768478	hypothetical protein	Early	
CT063	gnd	4433	0.2765677	6-phosphogluconate dehydrogenase		
CT454	argS	5361	0.2755128	arginyl-tRNA synthetase		
CT684		4603	0.2753095	cysteine desulfurase activator complex subunit SufB		
CT153		7850	0.2745301	MAC/perforin family protein		
CT297	rnc	2272	0.273808	ribonuclease III		
CT738	yycJ	2490	0.2733444	Zn-dependent hydrolase		
CT093	ribF	2842	0.2699016	bifunctional riboflavin kinase/FMN adenylyltransferase		
CT255		1240	0.2689725	hypothetical protein		
CT305	atpI	6006	0.2677309	V-type ATP synthase subunit I		
CT747	hemE	3142	0.2661826	uroporphyrinogen decarboxylase		
CT247	pdhC	4162	0.265296	branched-chain alpha-keto acid dehydrogenase subunit E2		
CT209	leuS	7504	0.2652656	leucyl-tRNA synthetase		
CT108	ybgI	2340	0.2650438	Acr family transporter		
CT055	sucB_1	3607	0.2649814	dihydrolipoamide succinyltransferase		
CT806	ptr	8992	0.2637693	insulinase family/protease III		
CT851	map	2684	0.2633291	methionine aminopeptidase	Early	
CT593.1		535	0.263319	hypothetical protein		
CT360		1857	0.2630741	hypothetical protein		Midlate
CT129	glnP	1905	0.2621251	amino acid ABC transporter ATPase		
CT644	yohI	3112	0.2619993	oxidoreductase		
CT805		4035	0.2588508	hypothetical protein		
CT839		3092	0.2583614	hypothetical protein		
CT765	rsbV_2	1010	0.2582369	anti-sigma F factor antagonist		
CT578		4700	0.2564476	hypothetical protein	Late	Late
CT746	hemN_2	4153	0.2562028	coproporphyrinogen III oxidase		
CT246	pdhB	3059	0.2544424	pyruvate dehydrogenase E1 component beta subunit		
CT390	aspC	3386	0.2543222	L,L-diaminopimelate aminotransferase		
CT726	rodA	3539	0.2537359	rod shape protein		
CT257		3535	0.253551	hypothetical protein		
CT640	recC	8446	0.2524235	exodeoxyribonuclease V gamma chain		
CT571	gspE	4191	0.2516693	general secretion pathway protein E		
CT165		1233	0.250691	hypothetical protein		
CT792	mutS	7366	0.2505168	DNA mismatch repair protein MutS		
CT249		1026	0.2498815	hypothetical protein		Very late
CT423		3011	0.2492884	CBS domain-containing protein		
CT184	yqgF	1382	0.247963	Holliday junction resolvase-like protein		
CT101		1331	0.2477702	hypothetical protein		
CT418	obgE	2759	0.2468727	GTPase ObgE		

CT357R		814	0.2458171	hypothetical protein		
CT718		1432	0.2439438	hypothetical protein		
CT259		1942	0.2433062	PP2C phosphatase family protein		Midlate
CT205	pfkA_1	4677	0.2425351	diphosphate--fructose-6-phosphate 1-phosphotransferase		
CT682	pbpB	9550	0.242075	penicillin-binding protein		
CT594	ycfH	2222	0.2414618	PHP superfamily hydrolase		
CT213	rpiA	1940	0.241378	ribose-5-phosphate isomerase A		Late
CT875		1744	0.2409584	hypothetical protein		Late
CT298	radA	3735	0.2406721	DNA repair protein RadA		
CT204	ybhI	3924	0.2403154	dicarboxylate translocator		
CT232	incB	921	0.2384298	inclusion membrane protein B		
CT015	phoH	3546	0.2383474	ATPase		
CT622		5637	0.2373209	hypothetical protein		Late
CT590		7982	0.2371093	hypothetical protein		Midlate
CT475	pheT	6554	0.236442	phenylalanyl-tRNA synthetase subunit beta		
CT308	atpA	4739	0.2360453	V-type ATP synthase subunit A		
CT200	oppC_1	2311	0.2358847	oligopeptide permease		
CT199	oppB_1	2585	0.235836	oligopeptide permease		
CT389		3314	0.2328829	hypothetical protein		
CT350		4586	0.2325987	hypothetical protein		
CT198	oppA_3	4179	0.2318529	oligopeptide binding protein		
CT135		2861	0.2314952	hypothetical protein		
CT855	fumC	3688	0.2304661	fumarate hydratase		
CT227		1072	0.229581	hypothetical protein		
CT146	ligA	5287	0.2290061	NAD-dependent DNA ligase LigA		
CT288		4660	0.2280746	hypothetical protein	Early	Late
CT197	gcp_1	2673	0.228024	putative DNA-binding/iron metalloprotein/AP endonuclease		
CT495		4218	0.2275143	ADP/ATP translocase	Early	
CT796	glyQ	7949	0.2267664	glycyl-tRNA synthetase		
CT235		1100	0.226229	CRP family transcriptional regulator		
CT838		2950	0.2254509	hypothetical protein		
CT791	uvrC	4633	0.2237993	excinuclease ABC subunit C		
CT794	dnaG	4405	0.2231074	DNA primase		
CT847		1379	0.2226799	hypothetical protein	Late	
CT484		2589	0.2219261	hypothetical protein		
CT417		2234	0.2210668	metal transport protein		
CT130	glnQ	1806	0.2207897	amino acid ABC transporter ATPase		
CT818	tyrP_2	2823	0.2201534	tyrosine transporter		
CT572	gspD	5550	0.2200094	general secretion pathway protein D		
CT650	recA	2749	0.2199874	recombinase A		Midlate
CT676		1316	0.2198088	hypothetical protein		
CT766	miaA	2642	0.2193513	tRNA delta(2)-isopentenylpyrophosphate transferase		
CT035		2042	0.2169937	biotin protein ligase	Early	Very late
CT284		3529	0.2161889	phospholipase D		
CT826	pssA	2022	0.2157327	CDP-diacylglycerol--serine O-phosphatidyltransferase		
CT127	ydhO	2086	0.2155457	polysaccharide hydrolase invasin repeat-containing protein		
CT620		6468	0.2128328	hypothetical protein		Late
CT229		1583	0.211273	hypothetical protein	Early	
CT456		7582	0.2109943	hypothetical protein		Late
CT604	groEL_2	3888	0.2109403	HSP-60		
CT277		1623	0.2095808	hypothetical protein		
CT496.1		318	0.2089303	hypothetical protein		

CT856	ychM	3994	0.2072116	sulfate transporter		
CT616		2947	0.2066732	hypothetical protein		
CT698	trmE	3139	0.2065532	tRNA modification GTPase TrmE		
CT483		889	0.2060415	hypothetical protein		
CT059	fer	613	0.2058454	ferredoxin		
CT685		1808	0.2057169	ABC transporter ATP-binding protein		
CT301	pknD	6482	0.2040098	serine/threonine-protein kinase		
CT566		2408	0.2037279	hypothetical protein		
CT240	recR	1378	0.2027102	recombination protein RecR		
CT331	dxs	4408	0.20156	1-deoxy-D-xylulose-5-phosphate synthase		
CT052	hemN_1	2472	0.1994579	coproporphyrinogen III oxidase		Late
CT206		1937	0.1985244	acyltransferase family protein		
CT183	pyrG	4053	0.1974824	CTP synthetase		
CT767		2362	0.1972917	hypothetical protein		
CT633	hemB	2292	0.1972412	delta-aminolevulinic acid dehydratase		
CT201	oppD	1933	0.1972003	oligopeptide transport ATPase		
CT088	sycE	946	0.1970457	secretion chaperone		
CT677	frr	1291	0.1963105	ribosome recycling factor		
CT221.1		295	0.1962349	hypothetical protein		
CT270	pbp3	4226	0.1947033	penicillin-binding protein		
CT586	uvrB	4497	0.1946438	excinuclease ABC subunit B		
CT387		4533	0.1942975	hypothetical protein		
CT109		1805	0.1939423	hypothetical protein		
CT807	plsB	2316	0.1926208	glycerol-3-phosphate acyltransferase		
CT488		1701	0.1926079	hypothetical protein		
CT844	yfhC	1058	0.1925757	cytosine deaminase		
CT481		1608	0.1923757	hypothetical protein		
CT010	htrB	3066	0.1920098	lipid A biosynthesis lauroyl acyltransferase		
CT573		2563	0.190617	hypothetical protein		
CT384		3503	0.1882578	hypothetical protein		
CT427		1791	0.1877179	hypothetical protein		
CT008	rnhB_1	1939	0.1873852	ribonuclease HIII		
CT714	gpsA	2067	0.1870994	NAD(P)H-dependent glycerol-3-phosphate dehydrogenase		
CT309		1691	0.1869014	hypothetical protein		
CT061	fliA	1632	0.1865393	RNA polymerase sigma factor sigma-28		
CT208	gseA	2466	0.1857514	3-deoxy-D-manno-octulosonic-acid transferase		
CT742	ygcA	2489	0.1839789	rRNA methyltransferase		
CT147		9577	0.1830891	hypothetical protein	Early	Very late
CT846		1387	0.1828684	hypothetical protein		
CT739	ftsK	4818	0.1808686	cell division protein FtsK		
CT580		2001	0.1790066	hypothetical protein		
CT426		2247	0.1745589	hypothetical protein		
CT745	hemG	2503	0.1738093	protoporphyrinogen oxidase		
CT391		2047	0.1728824	hypothetical protein		
CT033	recD_1	4455	0.1721867	exodeoxyribonuclease V alpha chain		
CT854		3535	0.1719491	ABC transporter permease fused to pyrimidine biosynthesis enzyme		
CT152	ycfV	1338	0.1711803	ABC transporter ATPase		
CT687	yfhO_1	2409	0.1707869	cysteine desulfurase		
CT755	groEL_3	2898	0.1706142	60 kDa chaperonin GroEL		
CT837		4162	0.1703334	hypothetical protein		
CT338		868	0.1678384	hypothetical protein		

CT459	prfB	2087	0.1672115	peptide chain release factor 2		Late
CT285	lplA_1	1414	0.1665363	lipoate-protein ligase A		
CT570	gspF	2020	0.1664476	general secretion pathway protein F		
CT346	elaC	1731	0.1659126	ribonuclease Z		Midlate
CT730	ribD	2197	0.1644965	riboflavin deaminase		
CT555		6927	0.1632644	SWI/SNF family helicase		
CT548		1101	0.1621617	hypothetical protein		
CT020	lepB	3533	0.1618725	signal peptidase I		
CT721	yfhO_2	2143	0.1617244	NifS-related protein		
CT809		590	0.1610418	hypothetical protein		
CT164		513	0.1609203	hypothetical protein		
CT595	dsbD	4034	0.1606704	Thio:disulfide interchange protein		
CT489	glgC	2348	0.1571834	glucose-1-phosphate adenylyltransferase		Late
CT171	trpA	1417	0.1565569	tryptophan synthase subunit alpha		
CT415	yebL	1530	0.1564621	solute-binding protein		
CT756	murF	2479	0.1560224	UDP-N-acetylmuramoyl-tripeptide--D-alanyl-D- alanine ligase		
CT534	lnt	2984	0.1559149	apolipoprotein N-acyltransferase		
CT299	hemC	1282	0.1553369	porphobilinogen deaminase		
CT725	birA	905	0.1537204	biotin--protein ligase		
CT325		766	0.1535967	hypothetical protein		
CT863		2595	0.1534407	hypothetical protein		
CT365		3076	0.1529762	hypothetical protein	Early	Late
CT695		2068	0.1505797	hypothetical protein		Late
CT366	aroA	2266	0.1502589	3-phosphoshikimate 1-carboxyvinyltransferase		
CT383		1193	0.1493602	hypothetical protein		
CT867		1788	0.1492081	hypothetical protein	Late	
CT473		484	0.1482725	hypothetical protein	Early	
CT202	oppF	1311	0.147785	oligopeptide transport ATPase		
CT085		2960	0.1475025	hypothetical protein		
CT591	sdhB	1128	0.1464399	succinate dehydrogenase iron-sulfur subunit		
CT609	rpoN	2236	0.14533	RNA polymerase factor sigma-54		
CT642		1424	0.1450648	hypothetical protein		
CT226		865	0.1419387	hypothetical protein		
CT233	incC	842	0.1408246	inclusion membrane protein C		Early
CT382	aroG	1374	0.1408199	3-deoxy-7-phosphoheptulonate synthase		
CT054	sucA	4784	0.140411	2-oxoglutarate dehydrogenase E1 component		
CT720		1286	0.1401839	NifU-related protein		
CT231		2431	0.1398317	sodium-dependent amino acid transporter		
CT862	lcrH_2	939	0.1393661	type III secretion chaperone		
CT167		2061	0.1392746	hypothetical protein		
CT224		691	0.1392554	hypothetical protein		
CT351		3241	0.139119	hypothetical protein		
CT133		1285	0.1385044	rRNA methylase	Early	
CT222		588	0.1380921	hypothetical protein		
CT083		797	0.1371928	hypothetical protein		Late
CT416		1102	0.1360971	ABC transporter ATPase		
CT724		818	0.1360734	hypothetical protein		
CT440		555	0.1356558	hypothetical protein		
CT078	folD	1275	0.1354628	bifunctional 5,10-methylene-tetrahydrofolate dehydrogenase/ 5,10-methylene-tetrahydrofolate cyclohydrolase		
CT699	psdD	1467	0.1353472	phosphatidylserine decarboxylase		
CT487	yhhF	891	0.1337155	methyltransferase		

CT219	ubiA	1377	0.132743	prenyltransferase		
CT778	priA	3461	0.1319877	primosome assembly protein PriA		
CT092	ychF	1639	0.1317055	GTP-dependent nucleic acid-binding protein EngD		
CT754	icc	1233	0.1310967	phosphohydrolase		
CT619		4090	0.1301223	hypothetical protein		Late
CT470	recO	1049	0.1289733	DNA repair protein RecO		
CT831	murB	1154	0.1287518	UDP-N-acetylenolpyruvoylglucosamine reductase		
CT367	aroL	777	0.1282924	shikimate kinase		
CT042	glgX	3011	0.1271869	glycogen hydrolase (debranching)		
CT860		2155	0.1260855	hypothetical protein		
CT605	ybbC	1739	0.1257743	hypothetical protein		
CT840	mesJ	1479	0.1250478	PP-loop superfamily ATPase		Late
CT647		752	0.1247678	hypothetical protein		
CT074	recF	1611	0.1246503	recombination protein F		
CT757	mraY	1455	0.1246252	phospho-N-acetylmuramoyl-pentapeptide- transferase		
CT345		535	0.1242902	hypothetical protein		
CT411	lpxB	2574	0.1220138	lipid-A-disaccharide synthase		
CT119	incA	1094	0.1219109	inclusion membrane protein A		
CT244		1765	0.1212154	hypothetical protein		
CT588	rbsU	2677	0.1211403	sigma regulatory family protein-PP2C phosphatase		
CT079		561	0.121091	hypothetical protein		
CT597	exbD	516	0.1210728	biopolymer transport protein		
CT060	flhA	2459	0.1189491	type III secretion system protein		
CT139	oppA_1	1719	0.1185962	oligopeptide binding protein		
CT077	yojL	1180	0.1184424	hypothetical protein		
CT465		828	0.1171992	hypothetical protein		
CT131		4604	0.1157573	transmembrane protein		
CT193	tgt	1476	0.1153986	queuine tRNA-ribosyltransferase		
CT047		1244	0.1140327	hypothetical protein		
CT175	oppA_2	2040	0.1110075	oligopeptide binding protein permease		Early
CT607	ung	847	0.1100326	uracil-DNA glycosylase		
CT638		1042	0.1095896	rod shape-determining protein MreC		
CT779		776	0.1082689	hypothetical protein		
CT468	atoC	1434	0.108057	2-component regulatory system-ATPase		
CT370	aroDE	1775	0.1063869	bifunctional 3-dehydroquinate dehydratase/shikimate dehydrogenase protein		
CT195		1274	0.1062684	hypothetical protein		
CT441	tsp	2573	0.1058073	tail-specific protease		Late
CT662	hemA	1176	0.1055538	glutamyl-tRNA reductase		Midlate
CT639	recB	3694	0.1055358	exodeoxyribonuclease V beta chain		
CT234		3436	0.1053527	hypothetical protein		
CT648		1438	0.102275	hypothetical protein	Early	
CT168		324	0.1012498	hypothetical protein		

(e)

Gene ID	Gene Symbol	Read Count	RPKM	Gene Name	Belland Classifier	Nicholson Classifier
CT444	omcA	127957	41.3098306	9kDa-cysteine-rich lipoprotein	Late	Late
CT398.1		643636	40.5691459	Ribonuclease P RNA		
CT480.1		73708	38.6236333	hypothetical protein		
CT081		73971	20.2924577	hypothetical protein		Very late
CT843	rpsO	50153	15.5987122	30S ribosomal protein S15		
CT500	ndk	76467	15.3072401	nucleoside diphosphate kinase		Midlate

CT786	rpmJ	20419	12.5496176	50S ribosomal protein L36		
CT565		62310	12.0735255	hypothetical protein		Late
CT267	ihfA	40326	11.3153837	integration host factor alpha-subunit		Midlate
CT802	rpsR	26456	9.4316339	30S ribosomal protein S18		
CT691		71911	8.8706087	hypothetical protein		
CT001		24863	8.5347422	hypothetical protein		Late
CT801	rpsF	29993	7.5920442	30S ribosomal protein S6		
CT460		22915	7.5726935	SWIB (YM74) complex protein		
CT617	rpsT	27018	7.5392159	30S ribosomal protein S20		
CT236	acpP	20443	7.3940661	acyl carrier protein		Late
CT785	rpmH	9254	6.1648024	50S ribosomal protein L34		
CT342	rpsU	12427	5.9863725	30S ribosomal protein S21		
CT787	rpsN	19929	5.6307961	30S ribosomal protein S14		
CT110	groEL	118535	5.6049313	chaperonin GroEL	Early	Midcycle
CT313	tal	60213	5.2089728	transaldolase B		Midlate
CT803	rplI	29363	5.0321246	50S ribosomal protein L9		
CT421.1		9013	4.8485025	hypothetical protein		
CT659		12601	4.6324643	hypothetical protein	Late	Late
CT666		13854	4.6052756	hypothetical protein		Midlate
CT443	omcB	93722	4.5613878	60kD cysteine-rich outer membrane protein	Late	Late
CT421.2		7988	4.225818	hypothetical protein		
CT665		12322	4.1355825	hypothetical protein		Midlate
CT080	ltuB	13295	4.0063474	late transcription unit B protein	Late	Late
CT508	rs11	18331	3.9853128	30S ribosomal protein S11		
CT022	rpmE2	15024	3.9273089	50S ribosomal protein L31 type B		
CT667		20465	3.9027617	hypothetical protein		Midlate
CT769	ybeB	16012	3.8385283	iojap superfamily protein		
CT784	rnpA	15941	3.7951152	ribonuclease P		
CT439m	rpsL	16299	3.765468	30S ribosomal protein S12		
CT316	rplL	17851	3.7372915	50S ribosomal protein L7/L12		
CT603	ahpC	25986	3.6922763	thioredoxin peroxidase		
CT734		28454	3.6534982	hypothetical protein	Early	Midlate
CT312		15384	3.5870277	ferredoxin		
CT398		32071	3.4770503	hypothetical protein		
CT834	rpmI	7760	3.4706333	50S ribosomal protein L35		
CT111	groES	13068	3.3814939	co-chaperonin GroES	Early	
CT125	rplM	18752	3.372223	50S ribosomal protein L13		
CT798	glgA	55307	3.3212414	glycogen synthase	Late	Midlate II
CT610		25742	3.160319	hypothetical protein		Midlate
CT438	rs7	16920	3.1323866	30S ribosomal protein S7		
CT799	ctc	19657	3.0598038	50S ribosomal protein L25/general stress protein Ctc		
CT538		25296	2.977701	hypothetical protein		
CT436	rpsJ	11541	2.9189718	30S ribosomal protein S10		
CT509	rpsM	12326	2.8410084	30S ribosomal protein S13		
CT043		16792	2.8024988	hypothetical protein		Midlate
CT098	rpsA	57918	2.7880614	30S ribosomal protein S1		
CT322	tuf	39654	2.7333683	elongation factor Tu		
CT431	clpP_1	18178	2.6612342	ATP-dependent Clp protease proteolytic subunit		Midlate
CT492	coaE	18910	2.6478869	dephospho-CoA kinase		Midlate II
CT556		13990	2.6421887	hypothetical protein		
CT842	pnp	65530	2.6250557	polynucleotide phosphorylase/polyadenylase		Midlate
CT524	rpsS	7858	2.616983	30S ribosomal protein S19		

CT525	rplB	25344	2.5759787	50S ribosomal protein L2		
CT526	rplW	10018	2.5756043	50S ribosomal protein L23		
CT743	hctA	12364	2.5526242	histone-like developmental protein	Late	
CT086	rpmB	8004	2.533681	50S ribosomal protein L28		
CT126	rpsI	11841	2.5242464	30S ribosomal protein S9		
CT420	rplU	9496	2.4953092	50S ribosomal protein L21		
CT429		28343	2.449707	hypothetical protein		
CT833	infC	14340	2.4247053	translation initiation factor IF-3		
CT810	rpmF	4904	2.3450274	50S ribosomal protein L32		
CT559	yscJ	26652	2.3395025	Yop proteins translocation lipoprotein J		Midlate
CT790		13678	2.3306351	hypothetical protein		
CT437	fusA	56695	2.2873179	elongation factor G		
CT835	rplT	9723	2.2648471	50S ribosomal protein L20		
CT348	yjjK	42774	2.2642508	ABC transporter ATPase		
CT317	rplJ	14199	2.2620724	50S ribosomal protein L10		
CT783		27295	2.2464743	disulfide bond isomerase	Late	Midlate II
CT066		12552	2.245391	hypothetical protein		Midlate
CT503		14131	2.197132	hypothetical protein		
CT289		29146	2.1952028	hypothetical protein		Midlate
CT770	fabF	31571	2.1720252	3-oxoacyl-(acyl carrier protein) synthase II		Midlate
CT663		9604	2.169652	hypothetical protein		Midlate
CT602		10266	2.1656992	hypothetical protein		
CT706	clpP	15625	2.1351812	ATP-dependent Clp protease proteolytic subunit		
CT319	rplK	10789	2.1327013	50S ribosomal protein L11		
CT655	kdsA	20420	2.1252619	2-dehydro-3-deoxyphosphooctonate aldolase		
CT355		25003	2.1249158	hypothetical protein		
CT496	pgsA_1	12332	2.1034452	CDP-diacylglycerol--glycerol-3-phosphate 3-phosphatidyltransferase		Midlate
CT506	rplQ	10804	2.0885137	50S ribosomal protein L17		
CT613	folP	32485	2.0593882	dihydropteroate synthase		
CT507	rpoA	28260	2.0554132	DNA-directed RNA polymerase subunit alpha		
CT314	rpoC	101160	2.0481621	DNA-directed RNA polymerase subunit beta'		
CT819	yccA	17201	2.035041	transport permease		Midlate
CT292	dut	10150	2.0202431	deoxyuridine 5'-triphosphate nucleotidohydrolase		
CT705	clpX	30160	1.9804951	ATP-dependent protease ATP-binding subunit ClpX		
CT150	rpmG	3497	1.9714612	50S ribosomal protein L33		
CT095	rbfA	8586	1.957213	ribosome-binding factor A		
CT707	tig	31162	1.9523891	trigger factor		
CT382.1		4576	1.9399025	hypothetical protein		
CT242		12065	1.9111798	OmpH-like Outer membrane protein		
CT681	ompA	26486	1.8772277	major outer membrane protein		Midlate II
CT505	gapA	22374	1.8710189	glyceraldehyde-3-phosphate dehydrogenase		Midlate
CT504		19186	1.8621851	hypothetical protein		Midlate
CT671		19248	1.8448947	hypothetical protein		Midlate
CT827	nrdA	66934	1.8041895	ribonucleotide-diphosphate reductase subunit alpha		Midlate
CT709	mreB	22827	1.7965789	rod shape-determining protein MreB		Midlate
CT519	rpsQ	5215	1.7934929	30S ribosomal protein S17		
CT511	rplO	8952	1.7781633	50S ribosomal protein L15		
CT626	rpsD	13328	1.769718	30S ribosomal protein S4		
CT402	lpxK	25131	1.758224	tetraacyldisaccharide 4'-kinase		
CT708		73565	1.7512154	SWF/SNF family helicase		Midlate
CT169	trpR	5995	1.7440721	Trp operon repressor		

CT771		8984	1.7423986	hydrolase/phosphatase-like protein		
CT780		9902	1.7366949	protein disulfide isomerase	Late	
CT692		25736	1.735956	inorganic phosphate transporter		
CT136		14582	1.7323721	lysophospholipase esterase		
CT775		15346	1.7311383	1-acyl-sn-glycerol-3-phosphate acyltransferase		Late
CT067	ytgA	19285	1.7263126	solute protein binding family		
CT381	artJ	15481	1.7256006	arginine binding protein		
CT523	rplV	6267	1.678718	50S ribosomal protein L22		
CT539	trxA	5950	1.6672268	thioredoxin		
CT849.1		3435	1.6661718	hypothetical protein		
CT518	rplN	7009	1.6647633	50S ribosomal protein L14		
CT359		10826	1.6335693	hypothetical protein		
CT269	murE	27455	1.6315372	UDP-N-acetylmuramoylalanyl-D-glutamate--2, 6-diaminopimelate ligase		
CT713	porB	18682	1.6249335	outer membrane protein B		Midlate II
CT407	dksA	7333	1.6126414	dnaK suppressor protein		
CT828	nrdB	19763	1.6079871	ribonucleotide-diphosphate reductase subunit beta		Midlate
CT668		12754	1.607224	hypothetical protein		Midlate
CT318	rplA	13557	1.599722	50S ribosomal protein L1		
CT065		31944	1.5940853	ADP/ATP translocase	Early	
CT068	ytgB_1	14494	1.5713382	rRNA methylase		
CT396	dnaK	39262	1.5631737	molecular chaperone DnaK		Late
CT097	nusA	24379	1.5514612	transcription elongation factor NusA		
CT768		30374	1.5508332	hypothetical protein		Midlate
CT513	rplR	6587	1.5496041	50S ribosomal protein L18		
CT664		43145	1.5306274	FHA domain-containing protein		Midlate
CT611		12728	1.5232445	hypothetical protein		Midlate
CT491	rho	24735	1.52082	transcription termination factor Rho		Midlate II
CT656		5184	1.5200637	hypothetical protein		
CT013	cydA	23762	1.5193675	cytochrome d ubiquinol oxidase subunit I		Midlate
CT702		9089	1.5067171	hypothetical protein		Very late
CT498	gidA	32573	1.4899686	tRNA uridine 5-carboxymethylaminomethyl modification enzyme GidA		
CT520	rpmC	3668	1.4659831	50S ribosomal protein L29		
CT800	pth	9027	1.4655789	peptidyl-tRNA hydrolase		
CT544	uhpC	22947	1.4611911	hexosphosphate transport		Midlate
CT614	folX	6459	1.458779	dihydroneopterin aldolase		
CT341	dnaJ	19346	1.455396	chaperone protein DnaJ		
CT228		10010	1.4414367	hypothetical protein	Early	
CT424	rsbV_1	5684	1.4408667	anti-sigma F factor antagonist		
CT512	rpsE	8292	1.4311387	30S ribosomal protein S5		
CT554	brnQ	20984	1.4190678	branched chain amino acid ABC transporter carrier protein		
CT241	yaeT	40128	1.4146311	OMP85 family membrane protein		Midlate
CT541	mip	12354	1.4021371	peptidyl-prolyl cis-trans isomerase		
CT516	rplE	8744	1.3927382	50S ribosomal protein L5		
CT584		8912	1.3746344	hypothetical protein		Midlate
CT527	rplD	10483	1.3738614	50S ribosomal protein L4		
CT419	rpmA	3704	1.3681151	50S ribosomal protein L27		
CT352		4852	1.3506071	hypothetical protein		
CT386		13814	1.3446012	metal dependent hydrolase		Midlate
CT315	rpoB	62593	1.3394806	DNA-directed RNA polymerase subunit beta		
CT763		6707	1.33786	hypothetical protein		

CT741	yajC	5403	1.3342188	preprotein translocase subunit YajC	
CT430	dapF	12769	1.3072251	diaminopimelate epimerase	Midlate
CT824		43965	1.3024114	zinc metalloprotease	
CT537	yjeE	7033	1.2983311	ATPase or kinase	
CT528	rplC	9377	1.2870041	50S ribosomal protein L3	
CT221	yqfU	13208	1.2866999	hypothetical protein	
CT510	secY	20405	1.2790332	preprotein translocase subunit SecY	
CT561	yscL	9730	1.2751667	type III secretion system protein	Midlate
CT002	gatC	4653	1.2667892	aspartyl/glutamyl-tRNA amidotransferase subunit C	
CT385	ycfF	4888	1.2636651	Hit family hydrolase	Midlate
CT028	rplS	5197	1.2635789	50S ribosomal protein L19	
CT517	rplX	4944	1.260762	50S ribosomal protein L24	
CT026	rpsP	4861	1.2550304	30S ribosomal protein S16	
CT296		6696	1.2533908	hypothetical protein	
CT621		36663	1.2508202	hypothetical protein	Midlate
CT096	infB	40639	1.2503143	translation initiation factor IF-2	
CT788		7254	1.2441536	leader (60) peptide-periplasmic	
CT361	dapA	12236	1.242059	dihydrodipicolinate synthase	
CT405	ribC	8550	1.2357354	riboflavin synthase subunit alpha	
CT701	secA	41278	1.2354756	preprotein translocase subunit SecA	Midlate
CT600	pal	8397	1.2331379	peptidoglycan-associated lipoprotein	Midlate
CT180	tauB	10117	1.2311003	nitrate/iron ABC transporter ATPase	
CT177	dsbG	10154	1.2199798	disulfide bond chaperone	
CT017		19053	1.2116205	hypothetical protein	Late
CT859	ispH	13814	1.2067763	4-hydroxy-3-methylbut-2-enyl diphosphate reductase	
CT243	lpxD	15139	1.2063048	UDP-3-O-[3-hydroxymyristoyl] glucosamine N-acyltransferase	
CT612	folA	6723	1.2057099	dihydrofolate reductase	
CT406	nrdR	6677	1.1991416	transcriptional regulator NrdR	
CT678	pyrH	10330	1.1971297	uridylate kinase	
CT039	dcd	8053	1.1968164	deoxycytidine triphosphate deaminase	
CT482		9093	1.19145	hypothetical protein	
CT323	infA	3171	1.1861734	translation initiation factor IF-1	
CT041		10990	1.1817881	hypothetical protein	Late
CT736	ybcL	6083	1.1795911	hypothetical protein	
CT515	rpsH	5266	1.1688084	30S ribosomal protein S8	
CT514	rplF	7425	1.1655267	50S ribosomal protein L6	
CT351a	secG	4203	1.1609324	preprotein translocase subunit SecG	
CT275	dnaA	18449	1.1588853	chromosomal replication initiation protein	
CT858		25646	1.146175	protease	
CT353	def	7131	1.1425044	peptide deformylase	
CT550		5688	1.1359986	hypothetical protein	Midlate
CT672	fliN	15008	1.1273801	type III secretion system protein	Midlate
CT582	minD	9935	1.1060616	chromosome partitioning ATPase-CHLTR plasmid protein homolog GP5D	
CT522	rpsC	8196	1.0991597	30S ribosomal protein S3	
CT321	secE	3168	1.0987296	preprotein translocase subunit SecE	
CT364	dapB	9725	1.0956429	dihydrodipicolinate reductase	
CT670		6527	1.0825593	hypothetical protein	Midlate
CT031		3872	1.0804687	hypothetical protein	
CT543	hisS	15751	1.0767884	histidyl-tRNA synthetase	
CT012	ybbP	9956	1.0632189	hypothetical protein	
CT294	sodM	7590	1.0585806	superoxide dismutase	

CT533	lpxC	10731	1.0577092	UDP-3-O-[3-hydroxymyristoyl] N-acetylglucosamine deacetylase		
CT094	truB	8753	1.0539123	tRNA pseudouridine synthase B		
CT253		8157	1.041969	hypothetical protein		
CT046	hctB	8006	1.0412061	histone-like protein 2	Late	Late
CT069	ytgC	16666	1.0305912	integral membrane protein		
CT090	lcrD	26550	1.0293166	low calcium response D		Midlate
CT674	yscC	34454	1.024511	Yop proteins translocation protein C/general secretion pathway protein		Midlate II
CT320	nusG	6491	1.0224418	transcription antitermination protein NusG		
CT070	ytgD	11759	1.0208357	integral membrane protein		
CT291	ptsN_2	5515	1.0153351	PTS IIA protein		
CT535	yciA	5518	1.0032389	acyl-CoA hydrolase		Midlate

(f)

Gene ID	Gene Symbol	Read Count	RPKM	Gene Name	Belland Classifier	Nicholson Classifier
CT444	omcA	127957	41.3098306	9kDa-cysteine-rich lipoprotein	Late	Late
CT398.1		643636	40.5691459	Ribonuclease P RNA		
CT081		73971	20.2924577	hypothetical protein		Very late
CT843	rpsO	50153	15.5987122	30S ribosomal protein S15		
CT500	ndk	76467	15.3072401	nucleoside diphosphate kinase		Midlate
CT786	rpmJ	20419	12.5496176	50S ribosomal protein L36		
CT565		62310	12.0735255	hypothetical protein		Late
CT802	rpsR	26456	9.4316339	30S ribosomal protein S18		
CT691		71911	8.8706087	hypothetical protein		
CT801	rpsF	29993	7.5920442	30S ribosomal protein S6		
CT617	rpsT	27018	7.5392159	30S ribosomal protein S20		
CT785	rpmH	9254	6.1648024	50S ribosomal protein L34		
CT342	rpsU	12427	5.9863725	30S ribosomal protein S21		
CT787	rpsN	19929	5.6307961	30S ribosomal protein S14		
CT110	groEL	118535	5.6049313	chaperonin GroEL	Early	Midcycle
CT803	rplI	29363	5.0321246	50S ribosomal protein L9		
CT421.1		9013	4.8485025	hypothetical protein		
CT659		12601	4.6324643	hypothetical protein	Late	Late
CT443	omcB	93722	4.5613878	60kD cysteine-rich outer membrane protein	Late	Late
CT421.2		7988	4.225818	hypothetical protein		
CT080	ltuB	13295	4.0063474	late transcription unit B protein	Late	Late
CT508	rs11	18331	3.9853128	30S ribosomal protein S11		
CT022	rpmE2	15024	3.9273089	50S ribosomal protein L31 type B		
CT784	rnpA	15941	3.7951152	ribonuclease P		
CT439m	rpsL	16299	3.765468	30S ribosomal protein S12		
CT316	rplL	17851	3.7372915	50S ribosomal protein L7/L12		
CT603	ahpC	25986	3.6922763	thioredoxin peroxidase		
CT734		28454	3.6534982	hypothetical protein	Early	Midlate
CT398		32071	3.4770503	hypothetical protein		
CT834	rpmI	7760	3.4706333	50S ribosomal protein L35		
CT111	groES	13068	3.3814939	co-chaperonin GroES	Early	
CT125	rplM	18752	3.372223	50S ribosomal protein L13		
CT438	rs7	16920	3.1323866	30S ribosomal protein S7		
CT799	ctc	19657	3.0598038	50S ribosomal protein L25/general stress protein Ctc		
CT436	rpsJ	11541	2.9189718	30S ribosomal protein S10		
CT509	rpsM	12326	2.8410084	30S ribosomal protein S13		
CT098	rpsA	57918	2.7880614	30S ribosomal protein S1		

CT322	tuf	39654	2.7333683	elongation factor Tu		
CT492	coaE	18910	2.6478869	dephospho-CoA kinase		Midlate II
CT842	pnp	65530	2.6250557	polynucleotide phosphorylase/polyadenylase		Midlate
CT524	rpsS	7858	2.616983	30S ribosomal protein S19		
CT525	rplB	25344	2.5759787	50S ribosomal protein L2		
CT526	rplW	10018	2.5756043	50S ribosomal protein L23		
CT086	rpmB	8004	2.533681	50S ribosomal protein L28		
CT126	rpsI	11841	2.5242464	30S ribosomal protein S9		
CT420	rplU	9496	2.4953092	50S ribosomal protein L21		
CT833	infC	14340	2.4247053	translation initiation factor IF-3		
CT810	rpmF	4904	2.3450274	50S ribosomal protein L32		
CT437	fusA	56695	2.2873179	elongation factor G		
CT835	rplT	9723	2.2648471	50S ribosomal protein L20		
CT317	rplJ	14199	2.2620724	50S ribosomal protein L10		
CT066		12552	2.245391	hypothetical protein		Midlate
CT602		10266	2.1656992	hypothetical protein		
CT319	rplK	10789	2.1327013	50S ribosomal protein L11		
CT496	pgsA_1	12332	2.1034452	CDP-diacylglycerol--glycerol-3-phosphate 3-phosphatidyltransferase		Midlate
CT506	rplQ	10804	2.0885137	50S ribosomal protein L17		
CT507	rpoA	28260	2.0554132	DNA-directed RNA polymerase subunit alpha		
CT314	rpoC	101160	2.0481621	DNA-directed RNA polymerase subunit beta'		
CT292	dut	10150	2.0202431	deoxyuridine 5'-triphosphate nucleotidohydrolase		
CT150	rpmG	3497	1.9714612	50S ribosomal protein L33		
CT095	rbfA	8586	1.957213	ribosome-binding factor A		
CT505	gapA	22374	1.8710189	glyceraldehyde-3-phosphate dehydrogenase		Midlate
CT504		19186	1.8621851	hypothetical protein		Midlate
CT519	rpsQ	5215	1.7934929	30S ribosomal protein S17		
CT511	rplO	8952	1.7781633	50S ribosomal protein L15		
CT626	rpsD	13328	1.769718	30S ribosomal protein S4		
CT402	lpxK	25131	1.758224	tetraacyldisaccharide 4'-kinase		
CT169	trpR	5995	1.7440721	Trp operon repressor		
CT136		14582	1.7323721	lysophospholipase esterase		
CT775		15346	1.7311383	1-acyl-sn-glycerol-3-phosphate acyltransferase		Late
CT523	rplV	6267	1.678718	50S ribosomal protein L22		
CT518	rplN	7009	1.6647633	50S ribosomal protein L14		
CT359		10826	1.6335693	hypothetical protein		
CT318	rplA	13557	1.599722	50S ribosomal protein L1		
CT065		31944	1.5940853	ADP/ATP translocase	Early	
CT097	nusA	24379	1.5514612	transcription elongation factor NusA		
CT768		30374	1.5508332	hypothetical protein		Midlate
CT513	rplR	6587	1.5496041	50S ribosomal protein L18		
CT498	gidA	32573	1.4899686	tRNA uridine 5-carboxymethylaminomethyl modification enzyme GidA		
CT520	rpmC	3668	1.4659831	50S ribosomal protein L29		
CT800	pth	9027	1.4655789	peptidyl-tRNA hydrolase		
CT341	dnaJ	19346	1.455396	chaperone protein DnaJ		
CT228		10010	1.4414367	hypothetical protein	Early	
CT512	rpsE	8292	1.4311387	30S ribosomal protein S5		
CT554	brnQ	20984	1.4190678	branched chain amino acid ABC transporter carrier protein		
CT516	rplE	8744	1.3927382	50S ribosomal protein L5		
CT527	rplD	10483	1.3738614	50S ribosomal protein L4		
CT419	rpmA	3704	1.3681151	50S ribosomal protein L27		
CT315	rpoB	62593	1.3394806	DNA-directed RNA polymerase subunit beta		

CT528	rplC	9377	1.2870041	50S ribosomal protein L3		
CT221	yqfU	13208	1.2866999	hypothetical protein		
CT510	secY	20405	1.2790332	preprotein translocase subunit SecY		
CT028	rplS	5197	1.2635789	50S ribosomal protein L19		
CT517	rplX	4944	1.260762	50S ribosomal protein L24		
CT026	rpsP	4861	1.2550304	30S ribosomal protein S16		
CT096	infB	40639	1.2503143	translation initiation factor IF-2		
CT788		7254	1.2441536	leader (60) peptide-periplasmic		
CT405	ribC	8550	1.2357354	riboflavin synthase subunit alpha		
CT180	tauB	10117	1.2311003	nitrate/iron ABC transporter ATPase		
CT017		19053	1.2116205	hypothetical protein		Late
CT678	pyrH	10330	1.1971297	uridylate kinase		
CT736	ybcL	6083	1.1795911	hypothetical protein		
CT515	rpsH	5266	1.1688084	30S ribosomal protein S8		
CT514	rplF	7425	1.1655267	50S ribosomal protein L6		
CT353	def	7131	1.1425044	peptide deformylase		
CT522	rpsC	8196	1.0991597	30S ribosomal protein S3		
CT321	secE	3168	1.0987296	preprotein translocase subunit SecE		
CT046	hctB	8006	1.0412061	histone-like protein 2	Late	Late
CT320	nusG	6491	1.0224418	transcription antitermination protein NusG		

(g)

Gene ID	Gene Symbol	Read Count	RPKM	Gene Name	Belland Classifier	Nicholson Classifier
CT480.1		73708	38.6236333	hypothetical protein		
CT267	ihfA	40326	11.3153837	integration host factor alpha-subunit		Midlate
CT001		24863	8.5347422	hypothetical protein		Late
CT460		22915	7.5726935	SWIB (YM74) complex protein		
CT236	acpP	20443	7.3940661	acyl carrier protein		Late
CT313	tal	60213	5.2089728	transaldolase B		Midlate
CT666		13854	4.6052756	hypothetical protein		Midlate
CT665		12322	4.1355825	hypothetical protein		Midlate
CT667		20465	3.9027617	hypothetical protein		Midlate
CT769	ybeB	16012	3.8385283	iojap superfamily protein		
CT312		15384	3.5870277	ferredoxin		
CT798	glgA	55307	3.3212414	glycogen synthase	Late	Midlate II
CT610		25742	3.160319	hypothetical protein		Midlate
CT538		25296	2.977701	hypothetical protein		
CT043		16792	2.8024988	hypothetical protein		Midlate
CT431	clpP_1	18178	2.6612342	ATP-dependent Clp protease proteolytic subunit		Midlate
CT556		13990	2.6421887	hypothetical protein		
CT743	hctA	12364	2.5526242	histone-like developmental protein	Late	
CT429		28343	2.449707	hypothetical protein		
CT559	yscJ	26652	2.3395025	Yop proteins translocation lipoprotein J		Midlate
CT790		13678	2.3306351	hypothetical protein		
CT348	yjjK	42774	2.2642508	ABC transporter ATPase		
CT783		27295	2.2464743	disulfide bond isomerase	Late	Midlate II
CT503		14131	2.197132	hypothetical protein		
CT289		29146	2.1952028	hypothetical protein		Midlate
CT770	fabF	31571	2.1720252	3-oxoacyl-(acyl carrier protein) synthase II		Midlate
CT663		9604	2.169652	hypothetical protein		Midlate
CT706	clpP	15625	2.1351812	ATP-dependent Clp protease proteolytic subunit		
CT655	kdsA	20420	2.1252619	2-dehydro-3-deoxyphosphooctonate aldolase		

CT355		25003	2.1249158	hypothetical protein		
CT613	folP	32485	2.0593882	dihydropteroate synthase		
CT819	yccA	17201	2.035041	transport permease		Midlate
CT705	clpX	30160	1.9804951	ATP-dependent protease ATP-binding subunit ClpX		
CT707	tig	31162	1.9523891	trigger factor		
CT382.1		4576	1.9399025	hypothetical protein		
CT242		12065	1.9111798	OmpH-like Outer membrane protein		
CT681	ompA	26486	1.8772277	major outer membrane protein		Midlate II
CT671		19248	1.8448947	hypothetical protein		Midlate
CT827	nrdA	66934	1.8041895	ribonucleotide-diphosphate reductase subunit alpha		Midlate
CT709	mreB	22827	1.7965789	rod shape-determining protein MreB		Midlate
CT708		73565	1.7512154	SWF/SNF family helicase		Midlate
CT771		8984	1.7423986	hydrolase/phosphatase-like protein		
CT780		9902	1.7366949	protein disulfide isomerase	Late	
CT692		25736	1.735956	inorganic phosphate transporter		
CT067	ytgA	19285	1.7263126	solute protein binding family		
CT381	artJ	15481	1.7256006	arginine binding protein		
CT539	trxA	5950	1.6672268	thioredoxin		
CT849.1		3435	1.6661718	hypothetical protein		
CT269	murE	27455	1.6315372	UDP-N-acetylmuramoylalanyl-D-glutamate--2, 6-diaminopimelate ligase		
CT713	porB	18682	1.6249335	outer membrane protein B		Midlate II
CT407	dksA	7333	1.6126414	dnaK suppressor protein		
CT828	nrdB	19763	1.6079871	ribonucleotide-diphosphate reductase subunit beta		Midlate
CT668		12754	1.607224	hypothetical protein		Midlate
CT068	ytgB_1	14494	1.5713382	rRNA methylase		
CT396	dnaK	39262	1.5631737	molecular chaperone DnaK		Late
CT664		43145	1.5306274	FHA domain-containing protein		Midlate
CT611		12728	1.5232445	hypothetical protein		Midlate
CT491	rho	24735	1.52082	transcription termination factor Rho		Midlate II
CT656		5184	1.5200637	hypothetical protein		
CT013	cydA	23762	1.5193675	cytochrome d ubiquinol oxidase subunit I		Midlate
CT702		9089	1.5067171	hypothetical protein		Very late
CT544	uhpC	22947	1.4611911	hexosphosphate transport		Midlate
CT614	folX	6459	1.458779	dihydroneopterin aldolase		
CT424	rsbV_1	5684	1.4408667	anti-sigma F factor antagonist		
CT241	yaeT	40128	1.4146311	OMP85 family membrane protein		Midlate
CT541	mip	12354	1.4021371	peptidyl-prolyl cis-trans isomerase		
CT584		8912	1.3746344	hypothetical protein		Midlate
CT352		4852	1.3506071	hypothetical protein		
CT386		13814	1.3446012	metal dependent hydrolase		Midlate
CT763		6707	1.33786	hypothetical protein		
CT741	yajC	5403	1.3342188	preprotein translocase subunit YajC		
CT430	dapF	12769	1.3072251	diaminopimelate epimerase		Midlate
CT824		43965	1.3024114	zinc metalloprotease		
CT537	yjeE	7033	1.2983311	ATPase or kinase		
CT561	yscL	9730	1.2751667	type III secretion system protein		Midlate
CT002	gatC	4653	1.2667892	aspartyl/glutamyl-tRNA amidotransferase subunit C		
CT385	ycfF	4888	1.2636651	Hit family hydrolase		Midlate
CT296		6696	1.2533908	hypothetical protein		
CT621		36663	1.2508202	hypothetical protein		Midlate
CT361	dapA	12236	1.242059	dihydrodipicolinate synthase		
CT701	secA	41278	1.2354756	preprotein translocase subunit SecA		Midlate

CT600	pal	8397	1.2331379	peptidoglycan-associated lipoprotein	Midlate
CT177	dsbG	10154	1.2199798	disulfide bond chaperone	
CT859	ispH	13814	1.2067763	4-hydroxy-3-methylbut-2-enyl diphosphate reductase	
CT243	lpxD	15139	1.2063048	UDP-3-O-[3-hydroxymyristoyl] glucosamine N-acyltransferase	
CT612	folA	6723	1.2057099	dihydrofolate reductase	
CT406	nrdR	6677	1.1991416	transcriptional regulator NrdR	
CT039	dcd	8053	1.1968164	deoxycytidine triphosphate deaminase	
CT482		9093	1.19145	hypothetical protein	
CT323	infA	3171	1.1861734	translation initiation factor IF-1	
CT041		10990	1.1817881	hypothetical protein	Late
CT351a	secG	4203	1.1609324	preprotein translocase subunit SecG	
CT275	dnaA	18449	1.1588853	chromosomal replication initiation protein	
CT858		25646	1.146175	protease	
CT550		5688	1.1359986	hypothetical protein	Midlate
CT672	fliN	15008	1.1273801	type III secretion system protein	Midlate
CT582	minD	9935	1.1060616	chromosome partitioning ATPase-CHLTR plasmid protein homolog GP5D	
CT364	dapB	9725	1.0956429	dihydrodipicolinate reductase	
CT670		6527	1.0825593	hypothetical protein	Midlate
CT031		3872	1.0804687	hypothetical protein	
CT543	hisS	15751	1.0767884	histidyl-tRNA synthetase	
CT012	ybbP	9956	1.0632189	hypothetical protein	
CT294	sodM	7590	1.0585806	superoxide dismutase	
CT533	lpxC	10731	1.0577092	UDP-3-O-[3-hydroxymyristoyl] N-acetylglucosamine deacetylase	
CT094	truB	8753	1.0539123	tRNA pseudouridine synthase B	
CT253		8157	1.041969	hypothetical protein	
CT069	ytgC	16666	1.0305912	integral membrane protein	
CT090	lcrD	26550	1.0293166	low calcium response D	Midlate
CT674	yscC	34454	1.024511	Yop proteins translocation protein C/general secretion pathway protein	Midlate II
CT070	ytgD	11759	1.0208357	integral membrane protein	
CT291	ptsN_2	5515	1.0153351	PTS IIA protein	
CT535	yciA	5518	1.0032389	acyl-CoA hydrolase	Midlate
