# Supplementary material for: Simultaneous Transcriptional Profiling of Bacteria and Their Host Cells
Source: PLoS One. 2013 Dec 4;8(12):e80597. doi: 10.1371/journal.pone.0080597 (PMC3851178; doi:10.1371/journal.pone.0080597)
Supplement: Table S3 — Annotation of previously detected chlamydial genes and Gene Ontology enrichment. (a) Genes detected as expressed at 1 hpi by Belland et al (2003) but not present in hRNA-Seq (RPKM>0.1 and 10 mapped reads) (b) Genes detected as expressed at 1 hpi by Belland et al (2003) but not present in hRNA-Seq at 1 hpi (RPKM>1.0 and 50 mapped reads (c) GO-term enrichment for differentially expressed (versus mock infected) host non-hypothetical genes at 1 hpi (d) GO-term enrichment for differentially expressed (versus mock infected) host non-hypothetical genes at 24 hpi. (PDF) [file pone.0080597.s007.pdf]

Table S3.

(a) Genes detected as expressed at 1 hpi by Belland et al (2003) but not present in hRNA-Seq (RPKM>0.1 and 10 mapped reads) (b) Genes detected as expressed at 1 hpi by Belland et al (2003) but not present in hRNA-Seq at 1 hpi (RPKM>1.0 and 50 mapped reads (c) GO-term enrichment for differentially expressed (versus mock infected) host non-hypothetical genes at 1 hpi (d) GO-term enrichment for differentially expressed (versus mock infected) host non-hypothetical genes at 24 hpi

(a)

Gene ID	Gene Symbol	Function
CT473	*yidD*	Predicted a-hemolysin
CT474		Conserved hypothetical
CT774	*cysQ*	Biphosphate phosphatase
CT850		Conserved hypothetical

(b)

Gene ID	Gene Symbol	Function
CT365		Conserved hypothetical
CT375	*aad*	D-amino acid dehydrogenase
CT376	*mdhC*	Malate dehydrogenase
CT474		Conserved hypothetical
CT480	*oppA.4*	Oligopeptide permease
CT774	*cysQ*	Biphosphate phosphatase
CT850		Conserved hypothetical
CT851	*map*	Methionine aminopeptidase

(c)

GO:0008150 biological_proces	210	387	biological_process
GO:0007568 aging (aging)	8	8	biological_process
GO:0048856 anatomical struct	99	123	biological_process
GO:0048646 anatomical struct	32	32	biological_process
GO:0009058 biosynthetic proc	116	126	biological_process

GO:0005975 carbohydrate me	28	28	biological_process
GO:0009056 catabolic process	28	66	biological_process
GO:0034655 nucleobase-cont	14	40	biological_process
GO:0016887 ATPase activity	12	12	molecular_function
GO:0003924 GTPase activity	24	24	molecular_function
GO:0007155 cell adhesion (ce	51	51	biological_process
GO:0007049 cell cycle (cell cy	21	22	biological_process
GO:0008219 cell death (cell de	61	61	biological_process
GO:0030154 cell differentiation	89	89	biological_process
GO:0051301 cell division (cell	6	6	biological_process
GO:0034330 cell junction orga	8	8	biological_process
GO:0000902 cell morphogenes	34	34	biological_process
GO:0048870 cell motility (cell r	40	40	biological_process
GO:0008283 cell proliferation (	52	52	biological_process
GO:0007267 cell-cell signaling	34	34	biological_process
GO:0022607 cellular compone	21	49	biological_process
GO:0065003 macromolecular	6	31	biological_process
GO:0006461 protein complex	24	24	biological_process
GO:0022618 ribonucleoprote	1	1	biological_process
GO:0034641 cellular nitrogen c	92	142	biological_process
GO:0006259 DNA metabolic p	13	13	biological_process
GO:0004518 nuclease activity	3	3	molecular_function
GO:0006464 cellular protein m	74	74	biological_process
GO:0051276 chromosome org	10	10	biological_process
GO:0007059 chromosome seg	1	1	biological_process
GO:0003013 circulatory syster	17	17	biological_process
GO:0051186 cofactor metaboli	7	7	biological_process
GO:0007010 cytoskeleton orga	29	29	biological_process
GO:0021700 developmental m	3	3	biological_process
GO:0009790 embryo developm	14	14	biological_process

GO:0030234 enzyme regulato	31	31	molecular_function
GO:0030198 extracellular matı	19	19	biological_process
GO:0006091 generation of pre	18	18	biological_process
GO:0040007 growth (growth)	17	17	biological_process
GO:0004386 helicase activity (	2	2	molecular_function
GO:0042592 homeostatic proc	42	42	biological_process
GO:0016810 hydrolase activity	3	3	molecular_function
GO:0016798 hydrolase activity	2	2	molecular_function
GO:0002376 immune system p	70	70	biological_process
GO:0016853 isomerase activit	1	1	molecular_function
GO:0016301 kinase activity (ki	42	42	molecular_function
GO:0016874 ligase activity (lig	12	12	molecular_function
GO:0006629 lipid metabolic pr	29	29	biological_process
GO:0040011 locomotion (locor	31	56	biological_process
GO:0016829 lyase activity (lya	10	10	molecular_function
GO:0061024 membrane organ	28	31	biological_process
GO:0007009 plasma membra	5	5	biological_process
GO:0008168 methyltransferas	1	1	molecular_function
GO:0007005 mitochondrion or	4	4	biological_process
GO:0007067 mitosis (mitosis)	2	2	biological_process
GO:0006397 mRNA processin	1	1	biological_process
GO:0050877 neurological syst	34	34	biological_process
GO:0071941 nitrogen cycle me	1	1	biological_process
GO:0001071 nucleic acid bindi	30	30	molecular_function
GO:0016779 nucleotidyltransfe	2	2	molecular_function
GO:0016491 oxidoreductase a	23	23	molecular_function
GO:0008233 peptidase activity	31	31	molecular_function
GO:0016791 phosphatase acti	7	7	molecular_function
GO:0043473 pigmentation (pig	1	1	biological_process
GO:0000988 protein binding tr	9	9	molecular_function

GO:0006457 protein folding (p	3	3	biological_process
GO:0051604 protein maturatio	9	9	biological_process
GO:0000003 reproduction (rep	30	30	biological_process
GO:0006950 response to stres	103	103	biological_process
GO:0007165 signal transductic	150	154	biological_process
GO:0004871 signal transduce	47	47	molecular_function
GO:0044281 small molecule m	74	99	biological_process
GO:0006520 cellular amino ac	6	6	biological_process
GO:0006790 sulfur compound	7	7	biological_process
GO:0044403 symbiosis, encon	11	11	biological_process
GO:0016746 transferase activi	2	2	molecular_function
GO:0016757 transferase activi	7	7	molecular_function
GO:0006412 translation (trans	6	6	biological_process
GO:0008135 translation factor	1	1	molecular_function
GO:0006810 transport (transpo	86	125	biological_process
GO:0030705 cytoskeleton-dep	2	2	biological_process
GO:0006913 nucleocytoplasm	10	10	biological_process
GO:0006605 protein targeting	9	9	biological_process
GO:0008565 protein transport	1	1	molecular_function
GO:0055085 transmembrane	35	47	biological_process
GO:0022857 transmembrane	37	37	molecular_function
GO:0016192 vesicle-mediatec	47	47	biological_process
GO:0032196 transposition (tra	1	1	biological_process
GO:0005575 cellular_compone	211	406	cellular_component
GO:0005623 cell (cell)	54	343	cellular_component
GO:0005737 cytoplasm (cytop	119	221	cellular_component
GO:0016023 cytoplasmic mer	25	25	cellular_component
GO:0005783 endoplasmic reti	39	39	cellular_component
GO:0005768 endosome (endc	16	16	cellular_component
GO:0005794 Golgi apparatus	44	44	cellular_component

GO:0005739 mitochondrion (r	39	39	cellular_component
GO:0005777 peroxisome (per	1	1	cellular_component
GO:0005840 ribosome (ribosc	1	1	cellular_component
GO:0005773 vacuole (vacuole	0	9	cellular_component
GO:0005764 lysosome (lysos	9	9	cellular_component
GO:0005829 cytosol (cytosol)	62	62	cellular_component
GO:0005576 extracellular regi	75	98	cellular_component
GO:0005615 extracellular spac	44	44	cellular_component
GO:0005622 intracellular (intra	69	282	cellular_component
GO:0005694 chromosome (ch	7	8	cellular_component
GO:0000228 nuclear chromo:	4	4	cellular_component
GO:0005929 cilium (cilium)	3	3	cellular_component
GO:0005856 cytoskeleton (cy	44	50	cellular_component
GO:0005634 nucleus (nucleus	97	106	cellular_component
GO:0005730 nucleolus (nucle	9	9	cellular_component
GO:0005811 lipid particle (lipid	2	2	cellular_component
GO:0005815 microtubule orga	7	7	cellular_component
GO:0005635 nuclear envelope	5	5	cellular_component
GO:0005654 nucleoplasm (nuc	10	10	cellular_component
GO:0043226 organelle (organe	33	238	cellular_component
GO:0005886 plasma membrar	139	139	cellular_component
GO:0043234 protein complex (	56	56	cellular_component
GO:0005578 proteinaceous ex	28	28	cellular_component
GO:0003674 molecular_functio	322	401	molecular_function
GO:0008092 cytoskeletal prote	19	19	molecular_function
GO:0003677 DNA binding (DN	56	56	molecular_function
GO:0019899 enzyme binding (	35	35	molecular_function
GO:0042393 histone binding (l	1	1	molecular_function
GO:0043167 ion binding (ion b	163	163	molecular_function
GO:0008289 lipid binding (lipic	23	23	molecular_function

GO:0030674 protein binding, b	3	3	molecular_function
GO:0003723 RNA binding (RN	7	8	molecular_function
GO:0003729 mRNA binding (r	1	1	molecular_function
GO:0032182 small conjugating	1	1	molecular_function
GO:0005198 structural molecu	20	20	molecular_function
GO:0008134 transcription fact	15	15	molecular_function
GO:0051082 unfolded protein	1	1	molecular_function

(d)

GO:0008150 biological_process	43	77	biological_process
GO:0048856 anatomical struct	22	27	biological_process
GO:0048646 anatomical struct	9	9	biological_process
GO:0009058 biosynthetic proc	17	20	biological_process
GO:0005975 carbohydrate met	5	5	biological_process
GO:0009056 catabolic process	3	14	biological_process
GO:0034655 nucleobase-conta	3	11	biological_process
GO:0016887 ATPase activity (.	5	5	molecular_function
GO:0003924 GTPase activity (	5	5	molecular_function
GO:0007155 cell adhesion (cell	7	7	biological_process
GO:0007049 cell cycle (cell cycl	6	6	biological_process
GO:0008219 cell death (cell de	12	12	biological_process
GO:0030154 cell differentiatior	23	23	biological_process
GO:0051301 cell division (cell c	1	1	biological_process
GO:0034330 cell junction orgai	2	2	biological_process
GO:0000902 cell morphogenes	9	9	biological_process
GO:0048870 cell motility (cell r	8	8	biological_process
GO:0008283 cell proliferation (	12	12	biological_process
GO:0007267 cell-cell signaling	5	5	biological_process
GO:0022607 cellular componei	5	11	biological_process
GO:0065003 macromolecular	1	8	biological_process

GO:0006461 protein complex	7	7	biological_process
GO:0034641 cellular nitrogen c	15	27	biological_process
GO:0006259 DNA metabolic p	2	2	biological_process
GO:0004518 nuclease activity	2	2	molecular_function
GO:0006464 cellular protein m	17	17	biological_process
GO:0051276 chromosome orga	2	2	biological_process
GO:0007059 chromosome segr	1	1	biological_process
GO:0003013 circulatory system	5	5	biological_process
GO:0051186 cofactor metaboli	2	2	biological_process
GO:0007010 cytoskeleton orga	8	8	biological_process
GO:0021700 developmental m	4	4	biological_process
GO:0009790 embryo developm	6	6	biological_process
GO:0030234 enzyme regulator	4	4	molecular_function
GO:0030198 extracellular matr	5	5	biological_process
GO:0006091 generation of pre	2	2	biological_process
GO:0040007 growth (growth)	5	5	biological_process
GO:0004386 helicase activity (h	1	1	molecular_function
GO:0042592 homeostatic proc	9	9	biological_process
GO:0016810 hydrolase activity	1	1	molecular_function
GO:0002376 immune system p	10	10	biological_process
GO:0016301 kinase activity (kir	11	11	molecular_function
GO:0016874 ligase activity (liga	1	1	molecular_function
GO:0006629 lipid metabolic pr	7	7	biological_process
GO:0040011 locomotion (locor	4	10	biological_process
GO:0016829 lyase activity (lyas	3	3	molecular_function
GO:0061024 membrane organi	6	6	biological_process
GO:0008168 methyltransferase	1	1	molecular_function
GO:0007005 mitochondrion or	1	1	biological_process
GO:0007067 mitosis (mitosis)	1	1	biological_process
GO:0050877 neurological syste	6	6	biological_process

GO:0001071 nucleic acid bindi	7	7	molecular_function
GO:0016779 nucleotidyltransfe	1	1	molecular_function
GO:0016491 oxidoreductase ac	2	2	molecular_function
GO:0008233 peptidase activity	7	7	molecular_function
GO:0016791 phosphatase activ	3	3	molecular_function
GO:0000988 protein binding tr	2	2	molecular_function
GO:0006457 protein folding (p	1	1	biological_process
GO:0051604 protein maturatio	2	2	biological_process
GO:0000003 reproduction (rep	11	11	biological_process
GO:0006950 response to stress	16	16	biological_process
GO:0007165 signal transductio	26	27	biological_process
GO:0004871 signal transducer	10	10	molecular_function
GO:0044281 small molecule m	14	22	biological_process
GO:0006520 cellular amino ac	1	1	biological_process
GO:0006790 sulfur compound	1	1	biological_process
GO:0044403 symbiosis, encom	1	1	biological_process
GO:0016757 transferase activit	3	3	molecular_function
GO:0006810 transport (transpo	18	22	biological_process
GO:0030705 cytoskeleton-dep	1	1	biological_process
GO:0006913 nucleocytoplasm	3	3	biological_process
GO:0006605 protein targeting	3	3	biological_process
GO:0055085 transmembrane t	6	11	biological_process
GO:0022857 transmembrane	11	11	molecular_function
GO:0016192 vesicle-mediated	7	7	biological_process
GO:0005575 cellular_compone	40	80	cellular_component
GO:0005623 cell (cell)	9	61	cellular_component
GO:0005737 cytoplasm (cytopl	23	38	cellular_component
GO:0016023 cytoplasmic mem	3	3	cellular_component
GO:0005783 endoplasmic retic	8	8	cellular_component
GO:0005768 endosome (endo	2	2	cellular_component

GO:0005794 Golgi apparatus (	11	11	cellular_component
GO:0005739 mitochondrion (r	7	7	cellular_component
GO:0005840 ribosome (riboso	1	1	cellular_component
GO:0005773 vacuole (vacuole)	0	1	cellular_component
GO:0005764 lysosome (lysosc	1	1	cellular_component
GO:0005829 cytosol (cytosol)	13	13	cellular_component
GO:0005576 extracellular regic	17	23	cellular_component
GO:0005615 extracellular spac	9	9	cellular_component
GO:0005622 intracellular (intra	7	46	cellular_component
GO:0005694 chromosome (ch	1	1	cellular_component
GO:0005856 cytoskeleton (cyt	12	12	cellular_component
GO:0005634 nucleus (nucleus)	17	20	cellular_component
GO:0005730 nucleolus (nucle	1	1	cellular_component
GO:0005635 nuclear envelope	1	1	cellular_component
GO:0005654 nucleoplasm (nuc	3	3	cellular_component
GO:0043226 organelle (organe	9	41	cellular_component
GO:0005886 plasma membran	32	32	cellular_component
GO:0043234 protein complex (	12	12	cellular_component
GO:0005578 proteinaceous ext	9	9	cellular_component
GO:0003674 molecular_functio	60	78	molecular_function
GO:0008092 cytoskeletal prote	5	5	molecular_function
GO:0003677 DNA binding (DNA	10	10	molecular_function
GO:0019899 enzyme binding (e	6	6	molecular_function
GO:0043167 ion binding (ion b	41	41	molecular_function
GO:0008289 lipid binding (lipid	4	4	molecular_function
GO:0005198 structural molecu	3	3	molecular_function
GO:0008134 transcription facto	2	2	molecular_function
GO:0051082 unfolded protein	1	1	molecular_function
