# Supplementary material for: Simultaneous Transcriptional Profiling of Bacteria and Their Host Cells
Source: PLoS One. 2013 Dec 4;8(12):e80597. doi: 10.1371/journal.pone.0080597 (PMC3851178; doi:10.1371/journal.pone.0080597)
Supplement: Table S4 — GO-term enrichment for Chlamydia non-hypothetical genes at 1 hpi. (PDF) [file pone.0080597.s008.pdf]

Table S4. GO-term enrichment for *Chlamydia* non-hypothetical genes at 1 hpi

GO:0008150 biological_process (biological_process)	70	189	biological_process
GO:0048856 anatomical structure development (anatomical structure development)	0	2	biological_process
GO:0009058 biosynthetic process (biosynthetic process)	22	81	biological_process
GO:0005975 carbohydrate metabolic process (carbohydrate metabolic process)	4	4	biological_process
GO:0009056 catabolic process (catabolic process)	3	15	biological_process
GO:0034655 nucleobase-containing compound catabolic process (nucleobase-containing compound catabolic process)	4	12	biological_process
GO:0016887 ATPase activity (ATPase activity)	6	6	molecular_function
GO:0003924 GTPase activity (GTPase activity)	2	2	molecular_function
GO:0051301 cell division (cell division)	1	1	biological_process
GO:0000902 cell morphogenesis (cell morphogenesis)	2	2	biological_process
GO:0048870 cell motility (cell motility)	1	1	biological_process
GO:0071554 cell wall organization or biogenesis (cell wall organization or biogenesis)	1	1	biological_process
GO:0022607 cellular component assembly (cellular component assembly)	1	1	biological_process
GO:0034641 cellular nitrogen compound metabolic process (cellular nitrogen compound metabolic process)	25	43	biological_process
GO:0006259 DNA metabolic process (DNA metabolic process)	1	1	biological_process
GO:0004518 nuclease activity (nuclease activity)	5	5	molecular_function
GO:0006399 tRNA metabolic process (tRNA metabolic process)	7	7	biological_process
GO:0006464 cellular protein modification process (cellular protein modification process)	3	3	biological_process
GO:0051276 chromosome organization (chromosome organization)	1	1	biological_process
GO:0051186 cofactor metabolic process (cofactor metabolic process)	3	3	biological_process
GO:0006091 generation of precursor metabolites and energy (generation of precursor metabolites and energy)	1	1	biological_process
GO:0040007 growth (growth)	1	1	biological_process
GO:0016810 hydrolase activity, acting on carbon-nitrogen (but not peptide) bonds (hydrolase activity, acting on carbon-nitrogen (but not peptide) bonds)	2	2	molecular_function
GO:0016853 isomerase activity (isomerase activity)	2	2	molecular_function
GO:0016301 kinase activity (kinase activity)	4	4	molecular_function
GO:0016874 ligase activity (ligase activity)	7	7	molecular_function
GO:0006629 lipid metabolic process (lipid metabolic process)	6	6	biological_process
GO:0040011 locomotion (locomotion)	0	1	biological_process
GO:0016829 lyase activity (lyase activity)	2	2	molecular_function
GO:0061024 membrane organization (membrane organization)	3	3	biological_process
GO:0008168 methyltransferase activity (methyltransferase activity)	3	3	molecular_function
GO:0016779 nucleotidyltransferase activity (nucleotidyltransferase activity)	5	5	molecular_function
GO:0016491 oxidoreductase activity (oxidoreductase activity)	1	1	molecular_function
GO:0008233 peptidase activity (peptidase activity)	3	3	molecular_function
GO:0006457 protein folding (protein folding)	3	3	biological_process
GO:0006950 response to stress (response to stress)	4	4	biological_process
GO:0044281 small molecule metabolic process (small molecule metabolic process)	12	26	biological_process
GO:0006520 cellular amino acid metabolic process (cellular amino acid metabolic process)	6	6	biological_process
GO:0016765 transferase activity, transferring alkyl or aryl (other than methyl) groups (transferase activity, transferring alkyl or aryl (other than methyl) groups)	2	2	molecular_function
GO:0006412 translation (translation)	59	59	biological_process
GO:0008135 translation factor activity, nucleic acid binding (translation factor activity, nucleic acid binding)	4	4	molecular_function
GO:0006810 transport (transport)	18	18	biological_process
GO:0008565 protein transporter activity (protein transporter activity)	5	5	molecular_function
GO:0055085 transmembrane transport (transmembrane transport)	1	14	biological_process
GO:0022857 transmembrane transporter activity (transmembrane transporter activity)	14	14	molecular_function
GO:0005575 cellular_component (cellular_component)	74	163	cellular_component
GO:0005623 cell (cell)	2	89	cellular_component
GO:0005737 cytoplasm (cytoplasm)	21	75	cellular_component
GO:0005739 mitochondrion (mitochondrion)	1	1	cellular_component

GO:0009536 plastid (plastid)	9	9	cellular_component
GO:0005840 ribosome (ribosome)	51	51	cellular_component
GO:0005829 cytosol (cytosol)	6	6	cellular_component
GO:0030312 external encapsulating structure (external encapsulating structure)	3	3	cellular_component
GO:0005576 extracellular region (extracellular region)	3	3	cellular_component
GO:0005622 intracellular (intracellular)	2	77	cellular_component
GO:0043226 organelle (organelle)	0	51	cellular_component
GO:0005886 plasma membrane (plasma membrane)	11	11	cellular_component
GO:0043234 protein complex (protein complex)	13	13	cellular_component
GO:0003674 molecular_function (molecular_function)	72	181	molecular_function
GO:0003677 DNA binding (DNA binding)	1	1	molecular_function
GO:0043167 ion binding (ion binding)	11	11	molecular_function
GO:0003723 RNA binding (RNA binding)	3	10	molecular_function
GO:0019843 rRNA binding (rRNA binding)	3	3	molecular_function
GO:0005198 structural molecule activity (structural molecule activity)	2	53	molecular_function
GO:0003735 structural constituent of ribosome (structural constituent of ribosome)	51	51	molecular_function
