# Supplementary material for: Simultaneous Transcriptional Profiling of Bacteria and Their Host Cells
Source: PLoS One. 2013 Dec 4;8(12):e80597. doi: 10.1371/journal.pone.0080597 (PMC3851178; doi:10.1371/journal.pone.0080597)
Supplement: Table S5 — Differentially expressed host cell genes at 1 and 24 hpi. (a) Human DE genes at 1 hpi (relative to mock). FDR<0.05 and LFC>2.0. (b) Human DE genes at 24 hpi (relative to mock). FDR<0.05 and LFC>2.0. Sorted by gene description. (PDF) [file pone.0080597.s009.pdf]

Table S5. (a) Human DE genes at 1 hpi (relative to mock). FDR<0.05 and LFC>2.0. Sorted by gene description. (b) Human DE genes at 24 hpi (relative to mock). FDR<0.05 and LFC>2.0. Sorted by gene description.

(a)

Gene ID	Feature	Gene Symbol	Log Fold Change	Read Count (1hpi infected)	Read Count (1 hpi mock)	FDR	Gene Description
ENSG00000138678	protein_coding	AGPAT9	3.3522416	67.1676741	6.577108967	0.01935274	1-acylglycerol-3-phosphate O-acyltransferase 9 [Source:HGNC Symbol;Acc:28157]
ENSG00000111335	protein_coding	OAS2	-3.3747551	1.26812632	13.15421793	0.03166038	2'-5'-oligoadenylate synthetase 2, 69/71kDa [Source:HGNC Symbol;Acc:8087]
ENSG00000135114	protein_coding	OASL	-2.1204932	84.0964725	365.6872586	6.0813E-06	2'-5'-oligoadenylate synthetase-like [Source:HGNC Symbol;Acc:8090]
ENSG00000144130	protein_coding	NT5DC4	-2.3837114	3.78083439	19.7313269	0.02160974	5'-nucleotidase domain containing 4 [Source:HGNC Symbol;Acc:27678]
ENSG00000131016	protein_coding	AKAP12	4.2463888	898.78937	47.35518456	1.4609E-07	A kinase (PRKA) anchor protein 12 [Source:HGNC Symbol;Acc:370]
ENSG00000173210	protein_coding	ABLIM3	3.70008	119.67354	9.207952554	0.0010398	actin binding LIM protein family, member 3 [Source:HGNC Symbol;Acc:29132]
ENSG00000159251	protein_coding	ACTC1	4.9807722	124.608629	3.94626538	2.5312E-05	actin, alpha, cardiac muscle 1 [Source:HGNC Symbol;Acc:143]
ENSG00000151726	protein_coding	ACSL1	3.8418672	75.4470538	5.261687174	0.00478396	acyl-CoA synthetase long-chain family member 1 [Source:HGNC Symbol;Acc:3569]
ENSG00000148848	protein_coding	ADAM12	2.8075691	524.931213	74.97904223	0.00058115	ADAM metallopeptidase domain 12 [Source:HGNC Symbol;Acc:190]
ENSG00000135074	protein_coding	ADAM19	8.0814711	356.311824	1.315421793	1.1129E-11	ADAM metallopeptidase domain 19 [Source:HGNC Symbol;Acc:197]
ENSG00000149451	protein_coding	ADAM33	-3.3500713	1.41901099	14.46963973	0.01136129	ADAM metallopeptidase domain 33 [Source:HGNC Symbol;Acc:15478]
ENSG00000156140	protein_coding	ADAMTS3	Inf	62.453051	0	0.00025937	ADAM metallopeptidase with thrombospondin type 1 motif, 3 [Source:HGNC Symbol;Acc:219]
ENSG00000049192	protein_coding	ADAMTS6	4.2332931	470.074663	24.99301408	4.8203E-07	ADAM metallopeptidase with thrombospondin type 1 motif, 6 [Source:HGNC Symbol;Acc:222]
ENSG00000143382	protein_coding	ADAMTSL4	-2.5522796	23.995848	140.7501319	4.862E-06	ADAMTS-like 4 [Source:HGNC Symbol;Acc:19706]
ENSG00000205696	antisense	ADARB2-AS1	-3.6531796	4.07767875	51.30144995	6.43E-07	ADARB2 antisense RNA 1 (non-protein coding) [Source:HGNC Symbol;Acc:23299]
ENSG00000185736	protein_coding	ADARB2	-4.0360689	23.17315	380.1568983	0	adenosine deaminase, RNA-specific, B2 [Source:HGNC Symbol;Acc:227]
ENSG00000185100	protein_coding	ADSSL1	-2.2749887	19.0249375	92.07952554	0.00164694	adenylosuccinate synthase like 1 [Source:HGNC Symbol;Acc:20093]
ENSG00000139211	protein_coding	AMIGO2	2.9187338	1034.48645	136.8038665	0.00030356	adhesion molecule with Ig-like domain 2 [Source:HGNC Symbol;Acc:24073]
ENSG00000175414	protein_coding	ARL10	3.0412646	140.773288	17.10048332	0.00396989	ADP-ribosylation factor-like 10 [Source:HGNC Symbol;Acc:22042]
ENSG00000185305	protein_coding	ARL15	3.3096337	130.425966	13.15421793	0.00193155	ADP-ribosylation factor-like 15 [Source:HGNC Symbol;Acc:25945]

ENSG00000175906	protein_coding	ARL4D	-2.1230486	14.1925872	61.82482429	0.0145748	ADP-ribosylation factor-like 4D [Source:HGNC Symbol;Acc:656]
ENSG00000150594	protein_coding	ADRA2A	Inf	171.756442	0	8.7015E-09	adrenoceptor alpha 2A [Source:HGNC Symbol;Acc:281]
ENSG00000128165	protein_coding	ADM2	-2.4941216	5.37016298	30.25470125	0.0175954	adrenomedullin 2 [Source:HGNC Symbol;Acc:28898]
ENSG00000166825	protein_coding	ANPEP	5.4357433	113.871967	2.630843587	2.0096E-05	alanyl (membrane) aminopeptidase [Source:HGNC Symbol;Acc:500]
ENSG00000128918	protein_coding	ALDH1A2	Inf	31.4224419	0	0.02077583	aldehyde dehydrogenase 1 family, member A2 [Source:HGNC Symbol;Acc:15472]
ENSG00000187134	protein_coding	AKR1C1	-2.8016024	34.7149457	242.03761	6.55E-09	aldo-keto reductase family 1, member C1 (dihydrodiol dehydrogenase 1; 20-alpha (3-alpha)-hydroxysteroid dehydrogenase) [Source:HGNC
ENSG00000163295	protein_coding	ALPI	-3.5020583	165.561738	1875.791477	0	alkaline phosphatase, intestinal [Source:HGNC Symbol;Acc:437]
ENSG00000162551	protein_coding	ALPL	Inf	77.1630573	0	4.0343E-05	alkaline phosphatase, liver/bone/kidney [Source:HGNC Symbol;Acc:438]
ENSG00000163283	protein_coding	ALPP	-3.4311996	224.260484	2419.060678	0	alkaline phosphatase, placental [Source:HGNC Symbol;Acc:439]
ENSG00000163286	protein_coding	ALPPL2	-3.3950279	15.5053467	163.1123024	3.0216E-11	alkaline phosphatase, placental-like 2 [Source:HGNC Symbol;Acc:441]
ENSG00000126878	protein_coding	AIF1L	-2.9049319	13.1720893	98.65663451	7.0114E-06	allograft inflammatory factor 1-like [Source:HGNC Symbol;Acc:28904]
ENSG00000198796	protein_coding	ALPK2	-3.2929251	93.0098701	911.5873029	0	alpha-kinase 2 [Source:HGNC Symbol;Acc:20565]
ENSG00000008311	protein_coding	AASS	Inf	48.5450412	0	0.00183687	aminoadipate-semialdehyde synthase [Source:HGNC Symbol;Acc:17366]
ENSG00000109321	protein_coding	AREG	-2.5846863	3.50846308	21.0467487	0.04076677	amphiregulin [Source:HGNC Symbol;Acc:651]
ENSG00000187151	protein_coding	ANGPTL5	-2.7774745	2.87775064	19.7313269	0.01694232	angiopoietin-like 5 [Source:HGNC Symbol;Acc:19705]
ENSG00000151150	protein_coding	ANK3	4.8073692	36.8321749	1.315421793	0.04091653	ankyrin 3, node of Ranvier (ankyrin G) [Source:HGNC Symbol;Acc:494]
ENSG00000159712	processed_transcript	ANKRD18CP	-2.3004232	7.47698816	36.83181022	0.01552937	ankyrin repeat domain 18C, pseudogene [Source:HGNC Symbol;Acc:43601]
ENSG00000244509	protein_coding	APOBEC3C	-2.9653668	6.06321794	47.35518456	7.0484E-05	apolipoprotein B mRNA editing enzyme, catalytic polypeptide-like 3C [Source:HGNC Symbol;Acc:17353]
ENSG00000181409	protein_coding	AATK	-3.0633622	5.82241098	48.67060636	0.00010092	apoptosis-associated tyrosine kinase [Source:HGNC Symbol;Acc:21]
ENSG00000130707	protein_coding	ASS1	-3.6938892	75.9301347	982.6200797	0	argininosuccinate synthase 1 [Source:HGNC Symbol;Acc:758]
ENSG00000184867	protein_coding	ARMCX2	Inf	311.675976	0	3.772E-12	armadillo repeat containing, X-linked 2 [Source:HGNC Symbol;Acc:16869]
ENSG00000137486	protein_coding	ARRB1	-2.7526027	13.8582188	93.39494734	2.9123E-05	arrestin, beta 1 [Source:HGNC Symbol;Acc:711]
ENSG00000172379	protein_coding	ARNT2	-3.5378153	11.7790389	136.8038665	5.7032E-11	aryl-hydrocarbon receptor nuclear translocator 2 [Source:HGNC Symbol;Acc:16876]
ENSG00000228253	protein_coding	J01415.25	-2.7116792	47.1884945	309.1241215	3.49E-12	ATP synthase protein 8 [Source:UniProtKB/Swiss-Prot;Acc:P03928]
ENSG00000165029	protein_coding	ABCA1	Inf	600.933332	0	4E-15	ATP-binding cassette, sub-family A (ABC1), member 1 [Source:HGNC Symbol;Acc:29]

ENSG00000167972	protein_coding	ABCA3	-2.5272352	4.79193279	27.62385766	0.01531366	ATP-binding cassette, sub-family A (ABC1), member 3 [Source:HGNC Symbol;Acc:33]
ENSG00000023839	protein_coding	ABCC2	-3.5404325	114.637474	1333.837699	0	ATP-binding cassette, sub-family C (CFTR/MRP), member 2 [Source:HGNC Symbol;Acc:53]
ENSG00000118777	protein_coding	ABCG2	-2.6405825	16.4537578	102.6028999	5.7508E-05	ATP-binding cassette, sub-family G (WHITE), member 2 [Source:HGNC Symbol;Acc:74]
ENSG00000143515	protein_coding	ATP8B2	3.6879474	440.77928	34.20096663	5.7676E-06	ATPase, aminophospholipid transporter, class I, type 8B, member 2 [Source:HGNC Symbol;Acc:13534]
ENSG00000074370	protein_coding	ATP2A3	4.5854708	63.1624966	2.630843587	0.0041469	ATPase, Ca++ transporting, ubiquitous [Source:HGNC Symbol;Acc:813]
ENSG00000158321	protein_coding	AUTS2	Inf	60.2776474	0	0.00050738	autism susceptibility candidate 2 [Source:HGNC Symbol;Acc:14262]
ENSG00000172232	protein_coding	AZU1	-4.4551945	0.53970986	11.83879614	0.00071312	azurocidin 1 [Source:HGNC Symbol;Acc:913]
ENSG00000187172	pseudogene	BAGE2	-3.9433045	6.84072445	105.2337435	4.8253E-11	B melanoma antigen family, member 2 [Source:HGNC Symbol;Acc:15723]
ENSG00000224388	antisense	BACE2-IT1	-3.5426573	4.51521993	52.61687174	2.6144E-06	BACE2 intronic transcript 1 (non-protein coding) [Source:HGNC Symbol;Acc:16024]
ENSG00000104081	protein_coding	BMF	2.8708477	115.466908	15.78506152	0.00986946	Bcl2 modifying factor [Source:HGNC Symbol;Acc:24132]
ENSG00000169255	protein_coding	B3GALNT1	Inf	120.323944	0	3.7786E-07	beta-1,3-N-acetylgalactosaminyltransferase 1 (globoside blood group) [Source:HGNC Symbol;Acc:918]
ENSG00000197580	protein_coding	BCO2	-2.8191787	2.23660318	15.78506152	0.0309895	beta-carotene oxygenase 2 [Source:HGNC Symbol;Acc:18503]
ENSG00000182240	protein_coding	BACE2	-2.3904864	299.293564	1569.2982	1.9021E-10	beta-site APP-cleaving enzyme 2 [Source:HGNC Symbol;Acc:934]
ENSG00000112276	protein_coding	BVES	Inf	39.9653462	0	0.00712481	blood vessel epicardial substance [Source:HGNC Symbol;Acc:1152]
ENSG00000174672	protein_coding	BRSK2	-3.3894686	1.88288841	19.7313269	0.00229235	BR serine/threonine kinase 2 [Source:HGNC Symbol;Acc:11405]
ENSG00000121753	protein_coding	BAI2	6.3379963	106.412351	1.315421793	9.7215E-06	brain-specific angiogenesis inhibitor 2 [Source:HGNC Symbol;Acc:944]
ENSG00000233436	protein_coding	BTBD18	-2.1199211	11.49976	49.98602815	0.01410719	BTB (POZ) domain containing 18 [Source:HGNC Symbol;Acc:37214]
ENSG00000110852	protein_coding	CLEC2B	-3.173763	12.3904222	111.8108524	3.7786E-07	C-type lectin domain family 2, member B [Source:HGNC Symbol;Acc:2053]
ENSG00000133466	protein_coding	C1QTNF6	-2.2603521	32.1230756	153.9043498	0.00013379	C1q and tumor necrosis factor related protein 6 [Source:HGNC Symbol;Acc:14343]
ENSG00000081803	protein_coding	CADPS2	Inf	35.3458673	0	0.01306583	Ca++-dependent secretion activator 2 [Source:HGNC Symbol;Acc:16018]
ENSG00000140945	protein_coding	CDH13	2.9451537	253.270532	32.88554484	0.00106243	cadherin 13, H-cadherin (heart) [Source:HGNC Symbol;Acc:1753]
ENSG00000062038	protein_coding	CDH3	-3.5757805	1.65477556	19.7313269	0.0012904	cadherin 3, type 1, P-cadherin (placental) [Source:HGNC Symbol;Acc:1762]
ENSG00000113361	protein_coding	CDH6	Inf	70.6752898	0	0.00010632	cadherin 6, type 2, K-cadherin (fetal kidney) [Source:HGNC Symbol;Acc:1765]
ENSG00000099834	protein_coding	CDHR5	-2.6601696	8.53214138	53.93229353	0.00101853	cadherin-related family member 5 [Source:HGNC Symbol;Acc:7521]
ENSG00000103381	protein_coding	CPPED1	5.5911106	63.4098932	1.315421793	0.00137345	calcineurin-like phosphoesterase domain containing 1 [Source:HGNC Symbol;Acc:25632]

ENSG00000153956	protein_coding	CACNA2D1	8.9240153	638.941834	1.315421793	2.3E-14	calcium channel, voltage-dependent, alpha 2/delta subunit 1 [Source:HGNC Symbol;Acc:1399]
ENSG00000182389	protein_coding	CACNB4	Inf	37.5624915	0	0.00944115	calcium channel, voltage-dependent, beta 4 subunit [Source:HGNC Symbol;Acc:1404]
ENSG00000157388	protein_coding	CACNA1D	-4.4374088	0.54640465	11.83879614	0.00166901	calcium channel, voltage-dependent, L type, alpha 1D subunit [Source:HGNC Symbol;Acc:1391]
ENSG00000162545	protein_coding	CAMK2N1	-3.1863346	1.44504952	13.15421793	0.01495304	calcium/calmodulin-dependent protein kinase II inhibitor 1 [Source:HGNC Symbol;Acc:24190]
ENSG00000152495	protein_coding	CAMK4	3.25637	150.838196	15.78506152	0.00163453	calcium/calmodulin-dependent protein kinase IV [Source:HGNC Symbol;Acc:1464]
ENSG00000153132	protein_coding	CLGN	4.1797754	95.3592416	5.261687174	0.00066881	calmegin [Source:HGNC Symbol;Acc:2060]
ENSG00000117519	protein_coding	CNN3	5.7706021	359.053655	6.577108967	1.3033E-09	calponin 3, acidic [Source:HGNC Symbol;Acc:2157]
ENSG00000107159	protein_coding	CA9	-2.1038309	19.5852358	84.18699478	0.00264283	carbonic anhydrase IX [Source:HGNC Symbol;Acc:1383]
ENSG00000174990	protein_coding	CA5A	Inf	46.0604251	0	0.00238782	carbonic anhydrase VA, mitochondrial [Source:HGNC Symbol;Acc:1377]
ENSG00000074410	protein_coding	CA12	5.4109765	335.801399	7.892530761	7.9373E-09	carbonic anhydrase XII [Source:HGNC Symbol;Acc:1371]
ENSG00000185015	protein_coding	CA13	Inf	29.5630723	0	0.03188714	carbonic anhydrase XIII [Source:HGNC Symbol;Acc:14914]
ENSG00000128510	protein_coding	CPA4	-2.0099475	31.3531929	126.2804922	0.00262688	carboxypeptidase A4 [Source:HGNC Symbol;Acc:15740]
ENSG00000137752	protein_coding	CASP1	-3.8330304	7.29180443	103.9183217	4.5602E-10	caspase 1, apoptosis-related cysteine peptidase [Source:HGNC Symbol;Acc:1499]
ENSG00000196954	protein_coding	CASP4	-2.3143441	121.656655	605.094025	1.0328E-07	caspase 4, apoptosis-related cysteine peptidase [Source:HGNC Symbol;Acc:1505]
ENSG00000204397	protein_coding	CARD16	-3.5369356	1.47327791	17.10048332	0.00144972	caspase recruitment domain family, member 16 [Source:HGNC Symbol;Acc:33701]
ENSG00000179862	protein_coding	CITED4	-2.9179522	30.4587412	230.1988139	8.5314E-10	Cbp/p300-interacting transactivator, with Glu/Asp-rich carboxy-terminal domain, 4 [Source:HGNC Symbol;Acc:18696]
ENSG00000012124	protein_coding	CD22	-3.2056934	1.42578868	13.15421793	0.01307154	CD22 molecule [Source:HGNC Symbol;Acc:1643]
ENSG00000135218	protein_coding	CD36	-2.6604656	4.57728309	28.93927946	0.01023343	CD36 molecule (thrombospondin receptor) [Source:HGNC Symbol;Acc:1663]
ENSG00000182985	protein_coding	CADM1	Inf	32.8218226	0	0.02005857	cell adhesion molecule 1 [Source:HGNC Symbol;Acc:5951]
ENSG00000172292	protein_coding	CERS6	3.0983403	270.376944	31.57012304	0.00046624	ceramide synthase 6 [Source:HGNC Symbol;Acc:23826]
ENSG00000108691	protein_coding	CCL2	3.6465577	49.4207728	3.94626538	0.03476347	chemokine (C-C motif) ligand 2 [Source:HGNC Symbol;Acc:10618]
ENSG00000161570	protein_coding	CCL5	-3.1456899	4.31038458	38.14723201	0.00013056	chemokine (C-C motif) ligand 5 [Source:HGNC Symbol;Acc:10632]
ENSG00000163739	protein_coding	CXCL1	Inf	140.454748	0	6.2183E-08	chemokine (C-X-C motif) ligand 1 (melanoma growth stimulating activity, alpha) [Source:HGNC Symbol;Acc:4602]
ENSG00000144476	protein_coding	CXCR7	2.3771273	539.857004	103.9183217	0.00412006	chemokine (C-X-C motif) receptor 7 [Source:HGNC Symbol;Acc:23692]
ENSG00000155962	protein_coding	CLIC2	Inf	37.7081989	0	0.00939541	chloride intracellular channel 2 [Source:HGNC Symbol;Acc:2063]

ENSG00000135902	protein_coding	CHRND	-3.1101279	1.21874604	10.52337435	0.01731728	cholinergic receptor, nicotinic, delta (muscle) [Source:HGNC Symbol;Acc:1965]
ENSG00000100399	protein_coding	CHADL	-3.6719368	0.92884754	11.83879614	0.00627875	chondroadherin-like [Source:HGNC Symbol;Acc:25165]
ENSG00000198108	protein_coding	CHSY3	5.7628429	142.851099	2.630843587	1.9837E-06	chondroitin sulfate synthase 3 [Source:HGNC Symbol;Acc:24293]
ENSG00000166446	protein_coding	CDYL2	-2.4796731	67.4489317	376.2106329	1.014E-08	chromodomain protein, Y-like 2 [Source:HGNC Symbol;Acc:23030]
ENSG00000182795	protein_coding	C1orf116	-2.518494	9.8716843	56.56313712	0.00049845	chromosome 1 open reading frame 116 [Source:HGNC Symbol;Acc:28667]
ENSG00000239887	protein_coding	C1orf226	-2.6386507	5.06947826	31.57012304	0.00918516	chromosome 1 open reading frame 226 [Source:HGNC Symbol;Acc:34351]
ENSG00000148426	protein_coding	C10orf47	-2.1478678	27.3073927	121.018805	0.00082869	chromosome 10 open reading frame 47 [Source:HGNC Symbol;Acc:23728]
ENSG00000166780	protein_coding	C16orf45	2.8884241	126.622441	17.10048332	0.00599504	chromosome 16 open reading frame 45 [Source:HGNC Symbol;Acc:19213]
ENSG00000168675	protein_coding	C18orf1	5.9226313	239.37344	3.94626538	2.6499E-08	chromosome 18 open reading frame 1 [Source:HGNC Symbol;Acc:1224]
ENSG00000183397	protein_coding	C19orf71	-2.1003978	49.6932405	213.0983305	0.00014714	chromosome 19 open reading frame 71 [Source:HGNC Symbol;Acc:34496]
ENSG00000178803	protein_coding	C22orf45	-3.5745097	1.21457144	14.46963973	0.00465126	chromosome 22 open reading frame 45 [Source:HGNC Symbol;Acc:37122]
ENSG00000163633	protein_coding	C4orf36	-2.589632	17.7008096	106.5491653	8.5046E-05	chromosome 4 open reading frame 36 [Source:HGNC Symbol;Acc:28386]
ENSG00000197261	protein_coding	C6orf141	Inf	68.1128332	0	0.00012513	chromosome 6 open reading frame 141 [Source:HGNC Symbol;Acc:21351]
ENSG00000250733	protein_coding	C8orf17	-2.8241355	2.22893177	15.78506152	0.02590059	chromosome 8 open reading frame 17 [Source:HGNC Symbol;Acc:17737]
ENSG00000168333	protein_coding	C8orf22	-3.2423039	3.61416226	34.20096663	0.00010632	chromosome 8 open reading frame 22 [Source:HGNC Symbol;Acc:31745]
ENSG00000176907	protein_coding	C8orf4	2.7364201	394.477355	59.19398071	0.0008401	chromosome 8 open reading frame 4 [Source:HGNC Symbol;Acc:1357]
ENSG00000184785	protein_coding	CXorf69	-2.1538113	11.8239672	52.61687174	0.02018924	chromosome X open reading frame 69 [Source:HGNC Symbol;Acc:41913]
ENSG00000181885	protein_coding	CLDN7	-2.9418459	3.7662041	28.93927946	0.00607178	claudin 7 [Source:HGNC Symbol;Acc:2049]
ENSG00000120885	protein_coding	CLU	2.8300252	832.487092	117.0725396	0.00035424	clusterin [Source:HGNC Symbol;Acc:2095]
ENSG00000100473	protein_coding	COCH	4.1387824	46.3439096	2.630843587	0.02746169	coagulation factor C homolog, cochlin (Limulus polyphemus) [Source:HGNC Symbol;Acc:2180]
ENSG00000168542	protein_coding	COL3A1	3.5559077	897.279856	76.29446402	6.3204E-06	collagen, type III, alpha 1 [Source:HGNC Symbol;Acc:2201]
ENSG00000187498	protein_coding	COL4A1	2.5267015	1917.77867	332.8017137	0.00587294	collagen, type IV, alpha 1 [Source:HGNC Symbol;Acc:2202]
ENSG00000134871	protein_coding	COL4A2	2.229936	2264.6898	482.7597982	0.02473098	collagen, type IV, alpha 2 [Source:HGNC Symbol;Acc:2203]
ENSG00000130635	protein_coding	COL5A1	2.5880967	2452.00562	407.780756	0.00822715	collagen, type V, alpha 1 [Source:HGNC Symbol;Acc:2209]
ENSG00000080573	protein_coding	COL5A3	5.4815565	58.7730138	1.315421793	0.00212304	collagen, type V, alpha 3 [Source:HGNC Symbol;Acc:14864]

ENSG00000204291	protein_coding	COL15A1	-2.6203074	170.717015	1049.706591	2.513E-12	collagen, type XV, alpha 1 [Source:HGNC Symbol;Acc:2192]
ENSG00000084636	protein_coding	COL16A1	2.7571361	142.287152	21.0467487	0.00784127	collagen, type XVI, alpha 1 [Source:HGNC Symbol;Acc:2193]
ENSG00000188517	protein_coding	COL25A1	Inf	189.610865	0	2.3792E-09	collagen, type XXV, alpha 1 [Source:HGNC Symbol;Acc:18603]
ENSG00000198223	protein_coding	CSF2RA	-2.9714646	10.7335909	84.18699478	1.6453E-05	colony stimulating factor 2 receptor, alpha, low-affinity (granulocyte-macrophage) [Source:HGNC Symbol;Acc:2435]
ENSG00000123843	protein_coding	C4BPB	-3.3577113	97.0096638	994.4588759	0	complement component 4 binding protein, beta [Source:HGNC Symbol;Acc:1328]
ENSG00000224389	protein_coding	C4B	-3.6086789	4.20541672	51.30144995	7.5085E-06	complement component 4B (Chido blood group) [Source:HGNC Symbol;Acc:1324]
ENSG00000000971	protein_coding	CFH	3.0521027	207.297045	24.99301408	0.00092909	complement factor H [Source:HGNC Symbol;Acc:4883]
ENSG00000152910	protein_coding	CNTNAP4	-3.6033388	7.35971287	89.44868196	4.6558E-09	contactin associated protein-like 4 [Source:HGNC Symbol;Acc:18747]
ENSG00000163814	protein_coding	CDCP1	2.2109603	572.477282	123.6496486	0.0112139	CUB domain containing protein 1 [Source:HGNC Symbol;Acc:24357]
ENSG00000070729	protein_coding	CNGB1	-3.8191249	1.58431962	22.36217049	0.00010768	cyclic nucleotide gated channel beta 1 [Source:HGNC Symbol;Acc:2151]
ENSG00000116761	protein_coding	CTH	-2.2271359	42.9854764	201.2595344	0.00010543	cystathionase (cystathionine gamma-lyase) [Source:HGNC Symbol;Acc:2501]
ENSG00000182809	protein_coding	CRIP2	4.099887	135.333476	7.892530761	0.00010754	cysteine-rich protein 2 [Source:HGNC Symbol;Acc:2361]
ENSG00000121005	protein_coding	CRISPLD1	5.935664	80.5152125	1.315421793	0.00015335	cysteine-rich secretory protein LCCL domain containing 1 [Source:HGNC Symbol;Acc:18206]
ENSG00000140459	protein_coding	CYP11A1	-2.8467768	2.92563272	21.0467487	0.00715152	cytochrome P450, family 11, subfamily A, polypeptide 1 [Source:HGNC Symbol;Acc:2590]
ENSG00000019186	protein_coding	CYP24A1	5.8942156	78.2349383	1.315421793	0.00019289	cytochrome P450, family 24, subfamily A, polypeptide 1 [Source:HGNC Symbol;Acc:2602]
ENSG00000006016	protein_coding	CRLF1	Inf	312.84185	0	3.318E-12	cytokine receptor-like factor 1 [Source:HGNC Symbol;Acc:2364]
ENSG00000055163	protein_coding	CYFIP2	Inf	75.7399717	0	4.9067E-05	cytoplasmic FMR1 interacting protein 2 [Source:HGNC Symbol;Acc:13760]
ENSG00000165617	protein_coding	DACT1	Inf	35.9108613	0	0.01307154	dapper, antagonist of beta-catenin, homolog 1 (Xenopus laevis) [Source:HGNC Symbol;Acc:17748]
ENSG00000196730	protein_coding	DAPK1	-2.9765955	44.9544601	353.8484624	1.653E-12	death-associated protein kinase 1 [Source:HGNC Symbol;Acc:2674]
ENSG00000011465	protein_coding	DCN	-3.6512346	19.9971112	251.2455626	2E-14	decorin [Source:HGNC Symbol;Acc:2705]
ENSG00000134516	protein_coding	DOCK2	Inf	49.7108541	0	0.0017722	dedicator of cytokinesis 2 [Source:HGNC Symbol;Acc:2988]
ENSG00000181192	protein_coding	DHTKD1	-2.1438635	90.7815592	401.203647	1.7764E-06	dehydrogenase E1 and transketolase domain containing 1 [Source:HGNC Symbol;Acc:23537]
ENSG00000187908	protein_coding	DMBT1	5.481503	58.7708361	1.315421793	0.00229235	deleted in malignant brain tumors 1 [Source:HGNC Symbol;Acc:2926]
ENSG00000187957	protein_coding	DNER	2.0786296	188.918199	44.72434098	0.04352558	delta/notch-like EGF repeat containing [Source:HGNC Symbol;Acc:24456]
ENSG00000110042	protein_coding	DTX4	-2.1744367	20.1068234	90.76410375	0.00163453	deltex homolog 4 (Drosophila) [Source:HGNC Symbol;Acc:29151]

ENSG00000161249	protein_coding	DMKN	-4.1334141	5.17169013	90.76410375	4.2964E-11	dermokine [Source:HGNC Symbol;Acc:25063]
ENSG00000147202	protein_coding	DIAPH2	Inf	213.353843	0	5.3161E-10	diaphanous homolog 2 (Drosophila) [Source:HGNC Symbol;Acc:2877]
ENSG00000050165	protein_coding	DKK3	3.3312064	92.6736195	9.207952554	0.00667165	dickkopf 3 homolog (Xenopus laevis) [Source:HGNC Symbol;Acc:2893]
ENSG00000113657	protein_coding	DPYSL3	4.1539678	444.925753	24.99301408	6.0544E-07	dihydropyrimidinase-like 3 [Source:HGNC Symbol;Acc:3015]
ENSG00000133083	protein_coding	DCLK1	Inf	94.7650657	0	5.9686E-06	doublecortin-like kinase 1 [Source:HGNC Symbol;Acc:2700]
ENSG00000106976	protein_coding	DNM1	3.953469	163.030093	10.52337435	9.8397E-05	dynamin 1 [Source:HGNC Symbol;Acc:2972]
ENSG00000115423	protein_coding	DNAH6	-2.6390719	2.53399919	15.78506152	0.02785441	dynein, axonemal, heavy chain 6 [Source:HGNC Symbol;Acc:2951]
ENSG00000164330	protein_coding	EBF1	6.1966742	96.4827883	1.315421793	2.7656E-05	early B-cell factor 1 [Source:HGNC Symbol;Acc:3126]
ENSG00000164176	protein_coding	EDIL3	2.1607817	1329.33233	297.2853253	0.01531366	EGF-like repeats and discoidin I-like domains 3 [Source:HGNC Symbol;Acc:3173]
ENSG00000170571	protein_coding	EMB	4.0049737	253.433195	15.78506152	1.0197E-05	embigin [Source:HGNC Symbol;Acc:30465]
ENSG00000149218	protein_coding	ENDOD1	5.1408659	92.8217754	2.630843587	0.00015271	endonuclease domain containing 1 [Source:HGNC Symbol;Acc:29129]
ENSG00000171551	protein_coding	ECEL1	5.0763979	44.3826323	1.315421793	0.01307154	endothelin converting enzyme-like 1 [Source:HGNC Symbol;Acc:3147]
ENSG00000108515	protein_coding	ENO3	2.1688267	218.851057	48.67060636	0.02171043	enolase 3 (beta, muscle) [Source:HGNC Symbol;Acc:3354]
ENSG00000214860	protein_coding	EVPLL	-3.194672	2.29875627	21.0467487	0.01258814	envoplakin-like [Source:HGNC Symbol;Acc:35236]
ENSG00000145242	protein_coding	EPHA5	Inf	137.142915	0	1.2488E-07	EPH receptor A5 [Source:HGNC Symbol;Acc:3389]
ENSG00000133216	protein_coding	EPHB2	3.5132823	225.299194	19.7313269	0.00010517	EPH receptor B2 [Source:HGNC Symbol;Acc:3393]
ENSG00000125266	protein_coding	EFNB2	2.3065457	175.698581	35.51638842	0.0247032	ephrin-B2 [Source:HGNC Symbol;Acc:3227]
ENSG00000124882	protein_coding	EREG	-2.3499931	443.787507	2262.525485	1.3595E-11	epiregulin [Source:HGNC Symbol;Acc:3443]
ENSG00000182585	protein_coding	EPGN	-2.5063433	57.4159022	326.2246048	9.2408E-08	epithelial mitogen homolog (mouse) [Source:HGNC Symbol;Acc:17470]
ENSG00000196482	protein_coding	ESRRG	-3.8188446	4.66066919	65.77108967	2.7966E-08	estrogen-related receptor gamma [Source:HGNC Symbol;Acc:3474]
ENSG00000135373	protein_coding	EHF	-3.2624977	5.34588941	51.30144995	1.4707E-05	ets homologous factor [Source:HGNC Symbol;Acc:3246]
ENSG00000101210	protein_coding	EEF1A2	2.1011356	158.025771	36.83181022	0.04625777	eukaryotic translation elongation factor 1 alpha 2 [Source:HGNC Symbol;Acc:3192]
ENSG00000104313	protein_coding	EYA1	Inf	34.6371877	0	0.01428001	eyes absent homolog 1 (Drosophila) [Source:HGNC Symbol;Acc:3519]
ENSG00000116661	protein_coding	FBXO2	-2.8551057	2.90879135	21.0467487	0.01258814	F-box protein 2 [Source:HGNC Symbol;Acc:13581]
ENSG00000156804	protein_coding	FBXO32	3.9443417	364.504345	23.67759228	2.7786E-06	F-box protein 32 [Source:HGNC Symbol;Acc:16731]

ENSG00000158769	protein_coding	F11R	-2.2693966	34.6507243	167.0585678	0.00012071	F11 receptor [Source:HGNC Symbol;Acc:14685]
ENSG00000177096	protein_coding	FAM109B	5.4382992	57.0369395	1.315421793	0.00253062	family with sequence similarity 109, member B [Source:HGNC Symbol;Acc:27161]
ENSG00000135842	protein_coding	FAM129A	-2.0837116	30.4110496	128.9113358	0.00076989	family with sequence similarity 129, member A [Source:HGNC Symbol;Acc:16784]
ENSG00000154319	protein_coding	FAM167A	-2.8712414	3.05621685	22.36217049	0.0093871	family with sequence similarity 167, member A [Source:HGNC Symbol;Acc:15549]
ENSG00000144369	protein_coding	FAM171B	2.7861727	208.696137	30.25470125	0.00285713	family with sequence similarity 171, member B [Source:HGNC Symbol;Acc:29412]
ENSG00000204767	protein_coding	FAM196B	Inf	42.2697899	0	0.00568219	family with sequence similarity 196, member B [Source:HGNC Symbol;Acc:37271]
ENSG00000185112	protein_coding	FAM43A	3.406315	69.7329471	6.577108967	0.01545641	family with sequence similarity 43, member A [Source:HGNC Symbol;Acc:26888]
ENSG00000183508	protein_coding	FAM46C	-2.94171	12.840542	98.65663451	1.4394E-06	family with sequence similarity 46, member C [Source:HGNC Symbol;Acc:24712]
ENSG00000162670	protein_coding	FAM5C	-4.1286851	3.08312296	53.93229353	2.3016E-08	family with sequence similarity 5, member C [Source:HGNC Symbol;Acc:22393]
ENSG00000145945	protein_coding	FAM50B	5.1433125	46.4896599	1.315421793	0.01064152	family with sequence similarity 50, member B [Source:HGNC Symbol;Acc:18789]
ENSG00000126882	protein_coding	FAM78A	-4.0212326	2.9164592	47.35518456	3.0824E-07	family with sequence similarity 78, member A [Source:HGNC Symbol;Acc:25465]
ENSG00000147689	protein_coding	FAM83A	-2.6827357	23.7650705	152.588928	1.2505E-06	family with sequence similarity 83, member A [Source:HGNC Symbol;Acc:28210]
ENSG00000168672	protein_coding	FAM84B	-3.2772495	16.8242732	163.1123024	4.5602E-10	family with sequence similarity 84, member B [Source:HGNC Symbol;Acc:24166]
ENSG00000177138	protein_coding	FAM9B	-2.674409	2.06057151	13.15421793	0.04531521	family with sequence similarity 9, member B [Source:HGNC Symbol;Acc:18404]
ENSG00000170345	protein_coding	FOS	-2.1098214	202.050077	872.1246491	8.8828E-08	FBJ murine osteosarcoma viral oncogene homolog [Source:HGNC Symbol;Acc:3796]
ENSG00000114279	protein_coding	FGF12	5.3218706	52.6147737	1.315421793	0.00514925	fibroblast growth factor 12 [Source:HGNC Symbol;Acc:3668]
ENSG00000137440	protein_coding	FGFBP1	-2.908439	3.67923003	27.62385766	0.00528015	fibroblast growth factor binding protein 1 [Source:HGNC Symbol;Acc:19695]
ENSG00000164694	protein_coding	FNDC1	Inf	46.50841	0	0.00281586	fibronectin type III domain containing 1 [Source:HGNC Symbol;Acc:21184]
ENSG00000188738	protein_coding	FSIP2	3.1274601	172.431093	19.7313269	0.00146224	fibrous sheath interacting protein 2 [Source:HGNC Symbol;Acc:21675]
ENSG00000140092	protein_coding	FBLN5	5.2064797	194.281555	5.261687174	6.3612E-07	fibulin 5 [Source:HGNC Symbol;Acc:3602]
ENSG00000128591	protein_coding	FLNC	4.0173248	213.010151	13.15421793	2.0608E-05	filamin C, gamma [Source:HGNC Symbol;Acc:3756]
ENSG00000110195	protein_coding	FOLR1	9.63464	1045.63632	1.315421793	0	folate receptor 1 (adult) [Source:HGNC Symbol;Acc:3791]
ENSG00000053108	protein_coding	FSTL4	2.7007834	410.509368	63.14024609	0.00115727	follistatin-like 4 [Source:HGNC Symbol;Acc:21389]
ENSG00000176678	protein_coding	FOXL1	Inf	130.678225	0	1.8418E-07	forkhead box L1 [Source:HGNC Symbol;Acc:3817]
ENSG00000248905	protein_coding	FMN1	4.1519156	187.070871	10.52337435	3.0361E-05	formin 1 [Source:HGNC Symbol;Acc:3768]

ENSG00000155816	protein_coding	FMN2	Inf	61.7418251	0	0.0002285	formin 2 [Source:HGNC Symbol;Acc:14074]
ENSG00000134775	protein_coding	FHOD3	4.1216027	68.6929756	3.94626538	0.00624447	formin homology 2 domain containing 3 [Source:HGNC Symbol;Acc:26178]
ENSG00000111432	protein_coding	FZD10	-2.7171892	30.8056436	202.5749562	1.7692E-07	frizzled family receptor 10 [Source:HGNC Symbol;Acc:4039]
ENSG00000196371	protein_coding	FUT4	Inf	70.9873375	0	0.00011175	fucosyltransferase 4 (alpha (1,3) fucosyltransferase, myeloid-specific) [Source:HGNC Symbol;Acc:4015]
ENSG00000102302	protein_coding	FGD1	-2.0546587	31.6629322	131.5421793	0.0017722	FYVE, RhoGEF and PH domain containing 1 [Source:HGNC Symbol;Acc:3663]
ENSG00000111452	protein_coding	GPR133	Inf	65.2916152	0	0.00016524	G protein-coupled receptor 133 [Source:HGNC Symbol;Acc:19893]
ENSG00000166073	protein_coding	GPR176	2.5842312	354.98389	59.19398071	0.00264283	G protein-coupled receptor 176 [Source:HGNC Symbol;Acc:32370]
ENSG00000205336	protein_coding	GPR56	3.6959342	204.56594	15.78506152	6.1209E-05	G protein-coupled receptor 56 [Source:HGNC Symbol;Acc:4512]
ENSG00000099998	protein_coding	GGT5	-3.7624551	2.52015089	34.20096663	9.9166E-06	gamma-glutamyltransferase 5 [Source:HGNC Symbol;Acc:4260]
ENSG00000152661	protein_coding	GJA1	Inf	173.19351	0	5.7484E-09	gap junction protein, alpha 1, 43kDa [Source:HGNC Symbol;Acc:4274]
ENSG00000139278	protein_coding	GLIPR1	4.0332493	581.510858	35.51638842	6.6074E-07	GLI pathogenesis-related 1 [Source:HGNC Symbol;Acc:17001]
ENSG00000122694	protein_coding	GLIPR2	5.0573422	43.8002643	1.315421793	0.01486073	GLI pathogenesis-related 2 [Source:HGNC Symbol;Acc:18007]
ENSG00000174332	protein_coding	GLIS1	Inf	29.1115416	0	0.03463254	GLIS family zinc finger 1 [Source:HGNC Symbol;Acc:29525]
ENSG00000187210	protein_coding	GCNT1	Inf	33.0294661	0	0.01724189	glucosaminyl (N-acetyl) transferase 1, core 2 [Source:HGNC Symbol;Acc:4203]
ENSG00000135346	protein_coding	CGA	Inf	72.0908826	0	5.1271E-05	glycoprotein hormones, alpha polypeptide [Source:HGNC Symbol;Acc:1885]
ENSG00000112293	protein_coding	GPLD1	-2.3214402	6.31616021	31.57012304	0.02489121	glycosylphosphatidylinositol specific phospholipase D1 [Source:HGNC Symbol;Acc:4459]
ENSG00000185477	protein_coding	GPRIN3	Inf	44.4977465	0	0.00467125	GPRIN family member 3 [Source:HGNC Symbol;Acc:27733]
ENSG00000134317	protein_coding	GRHL1	-3.4090267	1.98137068	21.0467487	0.00222449	grainyhead-like 1 (Drosophila) [Source:HGNC Symbol;Acc:17923]
ENSG00000023171	protein_coding	GRAMD1B	-2.0895771	221.594412	943.1574259	2.4488E-07	GRAM domain containing 1B [Source:HGNC Symbol;Acc:29214]
ENSG00000166923	protein_coding	GREM1	8.6747435	537.555158	1.315421793	9.7E-14	gremlin 1 [Source:HGNC Symbol;Acc:2001]
ENSG00000183087	protein_coding	GAS6	3.4703035	72.8954591	6.577108967	0.01048074	growth arrest-specific 6 [Source:HGNC Symbol;Acc:4168]
ENSG00000130513	protein_coding	GDF15	-2.7127824	48.7576117	319.6474958	5.3411E-09	growth differentiation factor 15 [Source:HGNC Symbol;Acc:30142]
ENSG00000141738	protein_coding	GRB7	-3.2308816	2.10166748	19.7313269	0.0038394	growth factor receptor-bound protein 7 [Source:HGNC Symbol;Acc:4567]
ENSG00000114450	protein_coding	GNB4	2.0263019	605.509716	148.6426627	0.0226154	guanine nucleotide binding protein (G protein), beta polypeptide 4 [Source:HGNC Symbol;Acc:20731]
ENSG00000168243	protein_coding	GNG4	-2.7066748	2.41799819	15.78506152	0.0324573	guanine nucleotide binding protein (G protein), gamma 4 [Source:HGNC Symbol;Acc:4407]

ENSG00000117228	protein_coding	GBP1	Inf	223.972472	0	2.3825E-10	guanylate binding protein 1, interferon-inducible [Source:HGNC Symbol;Acc:4182]
ENSG00000162645	protein_coding	GBP2	6.6770534	134.604134	1.315421793	8.2507E-07	guanylate binding protein 2, interferon-inducible [Source:HGNC Symbol;Acc:4183]
ENSG00000189060	protein_coding	H1F0	-2.2516325	89.4954859	426.1966611	2.7727E-07	H1 histone family, member 0 [Source:HGNC Symbol;Acc:4714]
ENSG00000130600	lincRNA	H19	-3.5907888	1467.88046	17685.84601	0	H19, imprinted maternally expressed transcript (non-protein coding) [Source:HGNC Symbol;Acc:4713]
ENSG00000152137	protein_coding	HSPB8	2.4352533	234.781173	43.40891918	0.00750313	heat shock 22kDa protein 8 [Source:HGNC Symbol;Acc:30171]
ENSG00000100292	protein_coding	HMOX1	2.6661861	275.537656	43.40891918	0.00224518	heme oxygenase (decycling) 1 [Source:HGNC Symbol;Acc:5013]
ENSG00000143341	protein_coding	HMCN1	3.4553875	144.291353	13.15421793	0.00103774	hemicentin 1 [Source:HGNC Symbol;Acc:19194]
ENSG00000010704	protein_coding	HFE	-2.2017261	35.4569286	163.1123024	0.00040772	hemochromatosis [Source:HGNC Symbol;Acc:4886]
ENSG00000125430	protein_coding	HS3ST3B1	Inf	31.73391	0	0.02218831	heparan sulfate (glucosamine) 3-O-sulfotransferase 3B1 [Source:HGNC Symbol;Acc:5198]
ENSG00000171004	protein_coding	HS6ST2	2.1765057	350.841157	77.60988581	0.01355901	heparan sulfate 6-O-sulfotransferase 2 [Source:HGNC Symbol;Acc:19133]
ENSG00000185352	protein_coding	HS6ST3	Inf	32.5108944	0	0.02559833	heparan sulfate 6-O-sulfotransferase 3 [Source:HGNC Symbol;Acc:19134]
ENSG00000113070	protein_coding	HBEGF	3.4166441	112.374401	10.52337435	0.00249046	heparin-binding EGF-like growth factor [Source:HGNC Symbol;Acc:3059]
ENSG00000166503	protein_coding	RP11-382A20.3	3.910046	158.196232	10.52337435	0.00011342	Hepatoma-derived growth factor-related protein 3 [Source:UniProtKB/Swiss-Prot;Acc:Q9Y3E1]
ENSG00000156510	protein_coding	HKDC1	-2.7744342	3.46058572	23.67759228	0.01495304	hexokinase domain containing 1 [Source:HGNC Symbol;Acc:23302]
ENSG00000184357	protein_coding	HIST1H1B	2.2320961	1477.03506	314.3858086	0.02142213	histone cluster 1, H1b [Source:HGNC Symbol;Acc:4719]
ENSG00000124575	protein_coding	HIST1H1D	2.3850577	398.535439	76.29446402	0.00822715	histone cluster 1, H1d [Source:HGNC Symbol;Acc:4717]
ENSG00000198374	protein_coding	HIST1H2AL	2.2937028	406.330047	82.87157299	0.01695515	histone cluster 1, H2al [Source:HGNC Symbol;Acc:4730]
ENSG00000196532	protein_coding	HIST1H3C	3.1544913	105.415396	11.83879614	0.00950144	histone cluster 1, H3c [Source:HGNC Symbol;Acc:4768]
ENSG00000143452	protein_coding	HORMAD1	Inf	51.7292707	0	0.00102745	HORMA domain containing 1 [Source:HGNC Symbol;Acc:25245]
ENSG00000133328	protein_coding	HRASLS2	-3.7522299	1.26903806	17.10048332	0.0008102	HRAS-like suppressor 2 [Source:HGNC Symbol;Acc:17824]
ENSG00000166033	protein_coding	HTRA1	2.0131078	796.456628	197.313269	0.01694232	HtrA serine peptidase 1 [Source:HGNC Symbol;Acc:9476]
ENSG00000173805	protein_coding	HAP1	-2.7918404	3.22913547	22.36217049	0.00538712	huntingtin-associated protein 1 [Source:HGNC Symbol;Acc:4812]
ENSG00000010404	protein_coding	IDS	2.5309836	159.656395	27.62385766	0.01401613	iduronate 2-sulfatase [Source:HGNC Symbol;Acc:5389]
ENSG00000152580	protein_coding	IGSF10	7.3499131	858.359725	5.261687174	1.97E-13	immunoglobulin superfamily, member 10 [Source:HGNC Symbol;Acc:26384]
ENSG00000160223	protein_coding	ICOSLG	-2.9586296	18.1055849	140.7501319	1.8057E-07	inducible T-cell co-stimulator ligand [Source:HGNC Symbol;Acc:17087]

ENSG00000139269	protein_coding	INHBE	-3.9606745	3.04148511	47.35518456	1.761E-06	inhibin, beta E [Source:HGNC Symbol;Acc:24029]
ENSG00000117318	protein_coding	ID3	2.1363127	578.30806	131.5421793	0.00978342	inhibitor of DNA binding 3, dominant negative helix-loop-helix protein [Source:HGNC Symbol;Acc:5362]
ENSG00000165458	protein_coding	INPPL1	2.0642114	1837.38878	439.350879	0.03595535	inositol polyphosphate phosphatase-like 1 [Source:HGNC Symbol;Acc:6080]
ENSG00000068383	protein_coding	INPP5A	2.253621	163.09697	34.20096663	0.02931601	inositol polyphosphate-5-phosphatase, 40kDa [Source:HGNC Symbol;Acc:6076]
ENSG00000073792	protein_coding	IGF2BP2	-3.3707735	7.88411233	81.5561512	1.8469E-07	insulin-like growth factor 2 mRNA binding protein 2 [Source:HGNC Symbol;Acc:28867]
ENSG00000115461	protein_coding	IGFBP5	Inf	37.3208435	0	0.00970791	insulin-like growth factor binding protein 5 [Source:HGNC Symbol;Acc:5474]
ENSG00000137809	protein_coding	ITGA11	Inf	796.497857	0	0	integrin, alpha 11 [Source:HGNC Symbol;Acc:6136]
ENSG00000115232	protein_coding	ITGA4	Inf	68.0226747	0	0.000134	integrin, alpha 4 (antigen CD49D, alpha 4 subunit of VLA-4 receptor) [Source:HGNC Symbol;Acc:6140]
ENSG00000185885	protein_coding	IFITM1	-3.2675256	16.6648596	160.4814588	2.7799E-09	interferon induced transmembrane protein 1 [Source:HGNC Symbol;Acc:5412]
ENSG00000185201	protein_coding	IFITM2	-3.2081875	26.33153	243.3530318	1.2321E-10	interferon induced transmembrane protein 2 [Source:HGNC Symbol;Acc:5413]
ENSG00000142089	protein_coding	IFITM3	-2.7644204	125.448817	852.3933222	3.55E-13	interferon induced transmembrane protein 3 [Source:HGNC Symbol;Acc:5414]
ENSG00000115267	protein_coding	IFIH1	-2.2995242	69.4724491	342.0096663	5.6064E-07	interferon induced with helicase C domain 1 [Source:HGNC Symbol;Acc:18873]
ENSG00000184995	protein_coding	IFNE	4.6548158	66.2726302	2.630843587	0.00268876	interferon, epsilon [Source:HGNC Symbol;Acc:18163]
ENSG00000115594	protein_coding	IL1R1	2.209086	255.455634	55.24771533	0.01916106	interleukin 1 receptor, type I [Source:HGNC Symbol;Acc:5993]
ENSG00000095752	protein_coding	IL11	4.7557337	177.686131	6.577108967	4.4663E-06	interleukin 11 [Source:HGNC Symbol;Acc:5966]
ENSG00000174564	protein_coding	IL20RB	-3.034596	14.7689094	121.018805	1.2505E-06	interleukin 20 receptor beta [Source:HGNC Symbol;Acc:6004]
ENSG00000008517	protein_coding	IL32	Inf	39.2079933	0	0.00721176	interleukin 32 [Source:HGNC Symbol;Acc:16830]
ENSG00000168685	protein_coding	IL7R	3.1137285	364.368385	42.09349739	0.00022419	interleukin 7 receptor [Source:HGNC Symbol;Acc:6024]
ENSG00000169429	protein_coding	IL8	3.0552644	2449.2847	294.6544817	0.00123091	interleukin 8 [Source:HGNC Symbol;Acc:6025]
ENSG00000090376	protein_coding	IRAK3	Inf	35.6896929	0	0.01373839	interleukin-1 receptor-associated kinase 3 [Source:HGNC Symbol;Acc:17020]
ENSG00000148798	protein_coding	INA	Inf	50.1487034	0	0.00173822	internexin neuronal intermediate filament protein, alpha [Source:HGNC Symbol;Acc:6057]
ENSG00000114446	protein_coding	IFT57	2.847283	85.1971612	11.83879614	0.02391225	intraflagellar transport 57 homolog (Chlamydomonas) [Source:HGNC Symbol;Acc:17367]
ENSG00000011201	protein_coding	KAL1	Inf	73.5247816	0	6.6658E-05	Kallmann syndrome 1 sequence [Source:HGNC Symbol;Acc:6211]
ENSG00000102271	protein_coding	KLHL4	-3.2073843	9.82641833	90.76410375	6.0821E-07	kelch-like 4 (Drosophila) [Source:HGNC Symbol;Acc:6355]
ENSG00000131773	protein_coding	KHDRBS3	Inf	39.3616501	0	0.00675522	KH domain containing, RNA binding, signal transduction associated 3 [Source:HGNC Symbol;Acc:18117]

ENSG00000164976	protein_coding	KIAA1161	2.6405871	106.636112	17.10048332	0.02942024	KIAA1161 [Source:HGNC Symbol;Acc:19918]
ENSG00000103888	protein_coding	KIAA1199	-2.5779859	24.893868	148.6426627	5.7885E-06	KIAA1199 [Source:HGNC Symbol;Acc:29213]
ENSG00000112379	protein_coding	KIAA1244	3.2823089	76.7873549	7.892530761	0.0175954	KIAA1244 [Source:HGNC Symbol;Acc:21213]
ENSG00000110318	protein_coding	KIAA1377	-2.0360328	102.637976	420.9349739	5.7642E-06	KIAA1377 [Source:HGNC Symbol;Acc:29264]
ENSG00000130518	protein_coding	KIAA1683	-2.0565682	6.3242104	26.30843587	0.04158071	KIAA1683 [Source:HGNC Symbol;Acc:29350]
ENSG00000116852	protein_coding	KIF21B	-3.7025347	9.39666977	122.3342268	2.5793E-10	kinesin family member 21B [Source:HGNC Symbol;Acc:29442]
ENSG00000165115	protein_coding	KIF27	-2.4349594	6.81125647	36.83181022	0.01356113	kinesin family member 27 [Source:HGNC Symbol;Acc:18632]
ENSG00000223519	protein_coding	RP11-439E19.8	-3.190058	1.44132489	13.15421793	0.01123109	Kinesin-like protein KLP6 [Source:UniProtKB/Swiss-Prot;Acc:B7ZC32]
ENSG00000112769	protein_coding	LAMA4	7.3513064	1288.78361	7.892530761	9.8E-14	laminin, alpha 4 [Source:HGNC Symbol;Acc:6484]
ENSG00000050555	protein_coding	LAMC3	-2.0620578	22.3656814	93.39494734	0.00384391	laminin, gamma 3 [Source:HGNC Symbol;Acc:6494]
ENSG00000049323	protein_coding	LTBP1	6.061917	263.636248	3.94626538	6.815E-09	latent transforming growth factor beta binding protein 1 [Source:HGNC Symbol;Acc:6714]
ENSG00000119681	protein_coding	LTBP2	5.0331517	172.287856	5.261687174	2.6358E-06	latent transforming growth factor beta binding protein 2 [Source:HGNC Symbol;Acc:6715]
ENSG00000072071	protein_coding	LPHN1	-2.045354	17.5272915	72.34819864	0.00627875	latrophilin 1 [Source:HGNC Symbol;Acc:20973]
ENSG00000117114	protein_coding	LPHN2	2.7127145	129.349493	19.7313269	0.0130794	latrophilin 2 [Source:HGNC Symbol;Acc:18582]
ENSG00000233851	antisense	LATS2-AS1	-2.6400193	4.00953164	24.99301408	0.0275833	LATS2 antisense RNA 1 (non-protein coding) [Source:HGNC Symbol;Acc:39912]
ENSG00000110811	protein_coding	LEPREL2	Inf	80.8962711	0	2.5682E-05	leprecan-like 2 [Source:HGNC Symbol;Acc:19318]
ENSG00000073350	protein_coding	LLGL2	-2.4629398	17.1782446	94.71036913	0.00036655	lethal giant larvae homolog 2 (Drosophila) [Source:HGNC Symbol;Acc:6629]
ENSG00000172061	protein_coding	LRRC15	5.2418205	49.7748807	1.315421793	0.00713557	leucine rich repeat containing 15 [Source:HGNC Symbol;Acc:20818]
ENSG00000214425	pseudogene	LRRC37A4P	-3.0554494	1.74050708	14.46963973	0.0157957	leucine rich repeat containing 37, member A4, pseudogene [Source:HGNC Symbol;Acc:25479]
ENSG00000171488	protein_coding	LRRC8C	5.6397951	196.758627	3.94626538	2.4488E-07	leucine rich repeat containing 8 family, member C [Source:HGNC Symbol;Acc:25075]
ENSG00000170382	protein_coding	LRRN2	-2.5507012	3.14308047	18.41590511	0.0313073	leucine rich repeat neuronal 2 [Source:HGNC Symbol;Acc:16914]
ENSG00000244482	protein_coding	LILRA6	-2.9380263	1.54480445	11.83879614	0.02429036	leukocyte immunoglobulin-like receptor, subfamily A (with TM domain), member 6 [Source:HGNC Symbol;Acc:15495]
ENSG00000213626	protein_coding	LBH	4.8886724	77.934912	2.630843587	0.00079638	limb bud and heart development homolog (mouse) [Source:HGNC Symbol;Acc:29532]
ENSG00000103227	protein_coding	LMF1	-3.6877087	1.22499789	15.78506152	0.00369773	lipase maturation factor 1 [Source:HGNC Symbol;Acc:14154]
ENSG00000101670	protein_coding	LIPG	Inf	38.1744466	0	0.00922173	lipase, endothelial [Source:HGNC Symbol;Acc:6623]

ENSG00000234880	lincRNA	LINC00163	-2.4355843	6.07884566	32.88554484	0.01547055	long intergenic non-protein coding RNA 163 [Source:HGNC Symbol;Acc:33165]
ENSG00000226496	antisense	LINC00323	-3.581504	1.09881582	13.15421793	0.00566403	long intergenic non-protein coding RNA 323 [Source:HGNC Symbol;Acc:19720]
ENSG00000183674	lincRNA	LINC00518	-4.0472575	0.79564464	13.15421793	0.00083307	long intergenic non-protein coding RNA 518 [Source:HGNC Symbol;Acc:28626]
ENSG00000124466	protein_coding	LYPD3	-3.3883511	2.88933266	30.25470125	0.00067861	LY6/PLAUR domain containing 3 [Source:HGNC Symbol;Acc:24880]
ENSG00000171517	protein_coding	LPAR3	-2.4524522	4.56622121	24.99301408	0.00965731	lysophosphatidic acid receptor 3 [Source:HGNC Symbol;Acc:14298]
ENSG00000204642	protein_coding	HLA-F	Inf	30.0000783	0	0.02704707	major histocompatibility complex, class I, F [Source:HGNC Symbol;Acc:4963]
ENSG00000147676	lincRNA	MAL2	-3.3579193	73.9014702	757.682953	0	mal, T-cell differentiation protein 2 (gene/pseudogene) [Source:HGNC Symbol;Acc:13634]
ENSG00000071073	protein_coding	MGAT4A	-3.5998212	3.68883971	44.72434098	4.6544E-06	mannosyl (alpha-1,3-)-glycoprotein beta-1,4-N-acetylglucosaminyltransferase, isozyme A [Source:HGNC
ENSG00000214548	lincRNA	MEG3	Inf	150.244905	0	2.6499E-08	maternally expressed 3 (non-protein coding) [Source:HGNC Symbol;Acc:14575]
ENSG00000087245	protein_coding	MMP2	Inf	150.667508	0	2.9478E-08	matrix metallopeptidase 2 (gelatinase A, 72kDa gelatinase, 72kDa type IV collagenase) [Source:HGNC Symbol;Acc:7166]
ENSG00000125966	protein_coding	MMP24	-2.796169	4.73450192	32.88554484	0.00416029	matrix metallopeptidase 24 (membrane-inserted) [Source:HGNC Symbol;Acc:7172]
ENSG00000129270	protein_coding	MMP28	-4.3631923	0.63916544	13.15421793	0.00050146	matrix metallopeptidase 28 [Source:HGNC Symbol;Acc:14366]
ENSG00000167077	protein_coding	MEI1	Inf	47.7313612	0	0.00212653	meiosis inhibitor 1 [Source:HGNC Symbol;Acc:28613]
ENSG00000143995	protein_coding	MEIS1	2.1917966	318.520534	69.71735505	0.01428001	Meis homeobox 1 [Source:HGNC Symbol;Acc:7000]
ENSG00000213401	protein_coding	MAGEA12	-3.3940096	2.37749671	24.99301408	0.00071501	melanoma antigen family A, 12 [Source:HGNC Symbol;Acc:6799]
ENSG00000099399	protein_coding	MAGEB2	-3.6450897	13.6686949	171.0048332	7.01E-13	melanoma antigen family B, 2 [Source:HGNC Symbol;Acc:6809]
ENSG00000179222	protein_coding	MAGED1	-3.2978145	7.2230016	71.03277685	5.6064E-07	melanoma antigen family D, 1 [Source:HGNC Symbol;Acc:6813]
ENSG00000150054	protein_coding	MPP7	-2.0925164	49.3485964	210.467487	0.00011193	membrane protein, palmitoylated 7 (MAGUK p55 subfamily member 7) [Source:HGNC Symbol;Acc:26542]
ENSG00000106484	protein_coding	MEST	2.3205429	321.969058	64.45566788	0.00841493	mesoderm specific transcript homolog (mouse) [Source:HGNC Symbol;Acc:7028]
ENSG00000100060	protein_coding	MFNG	-4.1275301	0.90310013	15.78506152	0.00106679	MFNG O-fucosylpeptide 3-beta-N-acetylglucosaminyltransferase [Source:HGNC Symbol;Acc:7038]
ENSG00000117122	protein_coding	MFAP2	5.0257279	42.850892	1.315421793	0.01378713	microfibrillar-associated protein 2 [Source:HGNC Symbol;Acc:7033]
ENSG00000209707	miRNA	MIR24-2	-2.3081006	4.24988802	21.0467487	0.04000376	microRNA 24-2 [Source:HGNC Symbol;Acc:31608]
ENSG00000166963	protein_coding	MAP1A	5.267463	50.6674899	1.315421793	0.00611583	microtubule-associated protein 1A [Source:HGNC Symbol;Acc:6835]
ENSG00000131711	protein_coding	MAP1B	5.4696408	1457.23972	32.88554484	7.1222E-10	microtubule-associated protein 1B [Source:HGNC Symbol;Acc:6836]
ENSG00000249669	lincRNA	MIR143HG	Inf	57.5843668	0	0.00090602	MIR143 host gene (non-protein coding) [Source:HGNC Symbol;Acc:42872]

ENSG00000171889	lincRNA	MIR31HG	2.7358986	227.837883	34.20096663	0.00377537	MIR31 host gene (non-protein coding) [Source:HGNC Symbol;Acc:37187]
ENSG00000198899	protein_coding	MT-ATP6	-2.7284912	907.033937	6011.477596	1E-15	mitochondrially encoded ATP synthase 6 [Source:HGNC Symbol;Acc:7414]
ENSG00000198727	protein_coding	MT-CYB	-2.4021138	1218.92081	6442.935944	2.0628E-11	mitochondrially encoded cytochrome b [Source:HGNC Symbol;Acc:7427]
ENSG00000198712	protein_coding	MT-CO2	-2.2563408	2681.62311	12812.20827	3.2182E-07	mitochondrially encoded cytochrome c oxidase II [Source:HGNC Symbol;Acc:7421]
ENSG00000198888	protein_coding	MT-ND1	-2.0897091	1199.96149	5107.782824	3.1597E-09	mitochondrially encoded NADH dehydrogenase 1 [Source:HGNC Symbol;Acc:7455]
ENSG00000198763	protein_coding	MT-ND2	-2.1284821	1019.22834	4456.649036	2.4642E-09	mitochondrially encoded NADH dehydrogenase 2 [Source:HGNC Symbol;Acc:7456]
ENSG00000198886	protein_coding	MT-ND4	-2.5215909	2245.68862	12895.07984	3.3823E-10	mitochondrially encoded NADH dehydrogenase 4 [Source:HGNC Symbol;Acc:7459]
ENSG00000212907	protein_coding	MT-ND4L	-2.02524	427.530293	1740.303033	4.7624E-09	mitochondrially encoded NADH dehydrogenase 4L [Source:HGNC Symbol;Acc:7460]
ENSG00000198786	protein_coding	MT-ND5	-2.0491406	2157.19845	8927.767712	8.367E-07	mitochondrially encoded NADH dehydrogenase 5 [Source:HGNC Symbol;Acc:7461]
ENSG00000198695	protein_coding	MT-ND6	-2.6087341	262.019791	1598.237479	5E-15	mitochondrially encoded NADH dehydrogenase 6 [Source:HGNC Symbol;Acc:7462]
ENSG00000141639	protein_coding	MAPK4	-2.7379962	8.87275667	59.19398071	0.000145	mitogen-activated protein kinase 4 [Source:HGNC Symbol;Acc:6878]
ENSG00000180815	protein_coding	MAP3K15	-2.6481767	3.35741014	21.0467487	0.01694232	mitogen-activated protein kinase kinase kinase 15 [Source:HGNC Symbol;Acc:31689]
ENSG00000130675	protein_coding	MNX1	-4.0711056	1.17390074	19.7313269	0.00058115	motor neuron and pancreas homeobox 1 [Source:HGNC Symbol;Acc:4979]
ENSG00000226416	antisense	MRPL23-AS1	-4.2712761	1.43054686	27.62385766	5.757E-06	MRPL23 antisense RNA 1 (non-protein coding) [Source:HGNC Symbol;Acc:42812]
ENSG00000181143	protein_coding	MUC16	5.3616937	649.048151	15.78506152	7.7469E-10	mucin 16, cell surface associated [Source:HGNC Symbol;Acc:15582]
ENSG00000117983	protein_coding	MUC5B	-2.6801957	2.87325225	18.41590511	0.00960386	mucin 5B, oligomeric mucus/gel-forming [Source:HGNC Symbol;Acc:7516]
ENSG00000184956	protein_coding	MUC6	-3.4850261	4.93420215	55.24771533	5.8421E-06	mucin 6, oligomeric mucus/gel-forming [Source:HGNC Symbol;Acc:7517]
ENSG00000106780	protein_coding	MEGF9	2.3026315	577.585358	117.0725396	0.00679516	multiple EGF-like-domains 9 [Source:HGNC Symbol;Acc:3234]
ENSG00000105695	protein_coding	MAG	-4.4183084	0.49216607	10.52337435	0.00105692	myelin associated glycoprotein [Source:HGNC Symbol;Acc:6783]
ENSG00000149573	protein_coding	MPZL2	-2.8716131	25.1623603	184.1590511	4.6718E-08	myelin protein zero-like 2 [Source:HGNC Symbol;Acc:3496]
ENSG00000186487	protein_coding	MYT1L	-2.828945	4.44302725	31.57012304	0.00290717	myelin transcription factor 1-like [Source:HGNC Symbol;Acc:7623]
ENSG00000141052	protein_coding	MYOCD	Inf	288.477819	0	1.5465E-11	myocardin [Source:HGNC Symbol;Acc:16067]
ENSG00000081189	protein_coding	MEF2C	3.6987734	85.4037201	6.577108967	0.00385886	myocyte enhancer factor 2C [Source:HGNC Symbol;Acc:6996]
ENSG00000138347	protein_coding	MYPN	-2.5671051	129.850986	769.5217492	1.3488E-11	myopalladin [Source:HGNC Symbol;Acc:23246]
ENSG00000167306	protein_coding	MYO5B	-2.1403339	36.9983199	163.1123024	0.00036661	myosin VB [Source:HGNC Symbol;Acc:7603]

ENSG00000101335	protein_coding	MYL9	2.580984	283.348634	47.35518456	0.00311116	myosin, light chain 9, regulatory [Source:HGNC Symbol;Acc:15754]
ENSG00000104419	protein_coding	NDRG1	-2.0662484	266.98153	1118.108524	9.863E-08	N-myc downstream regulated 1 [Source:HGNC Symbol;Acc:7679]
ENSG00000113389	protein_coding	NPR3	4.8221202	707.003349	24.99301408	8.7981E-09	natriuretic peptide receptor C/guanylate cyclase C (atrionatriuretic peptide receptor C) [Source:HGNC Symbol;Acc:7945]
ENSG00000176771	protein_coding	NCKAP5	4.996337	41.9867564	1.315421793	0.02058217	NCK-associated protein 5 [Source:HGNC Symbol;Acc:29847]
ENSG00000184613	protein_coding	NELL2	Inf	44.1856664	0	0.00330617	NEL-like 2 (chicken) [Source:HGNC Symbol;Acc:7751]
ENSG00000154654	protein_coding	NCAM2	Inf	46.5724366	0	0.00265484	neural cell adhesion molecule 2 [Source:HGNC Symbol;Acc:7657]
ENSG00000182379	protein_coding	NXPH4	-2.6787675	2.46522699	15.78506152	0.04091653	neurexophilin 4 [Source:HGNC Symbol;Acc:8078]
ENSG00000067798	protein_coding	NAV3	2.427973	226.520603	42.09349739	0.01258814	neuron navigator 3 [Source:HGNC Symbol;Acc:15998]
ENSG00000144460	protein_coding	NYAP2	-2.0263914	28.0918074	114.441696	0.00265484	neuronal tyrosine-phosphorylated phosphoinositide-3-kinase adaptor 2 [Source:HGNC Symbol;Acc:29291]
ENSG00000056291	protein_coding	NPFFR2	-3.0741261	2.03050573	17.10048332	0.00435438	neuropeptide FF receptor 2 [Source:HGNC Symbol;Acc:4525]
ENSG00000101188	protein_coding	NTSR1	-3.7563366	1.07074916	14.46963973	0.0017722	neurotensin receptor 1 (high affinity) [Source:HGNC Symbol;Acc:8039]
ENSG00000116962	protein_coding	NID1	6.8764416	154.553985	1.315421793	1.8097E-07	nidogen 1 [Source:HGNC Symbol;Acc:7821]
ENSG00000172548	protein_coding	NIPAL4	Inf	41.4084	0	0.00574195	NIPA-like domain containing 4 [Source:HGNC Symbol;Acc:28018]
ENSG00000198929	protein_coding	NOS1AP	-3.0954992	6.15583411	52.61687174	0.00016639	nitric oxide synthase 1 (neuronal) adaptor protein [Source:HGNC Symbol;Acc:16859]
ENSG00000091592	protein_coding	NLRP1	Inf	93.9984114	0	5.9247E-06	NLR family, pyrin domain containing 1 [Source:HGNC Symbol;Acc:14374]
ENSG00000188505	protein_coding	NCCRP1	-3.7832347	2.19748411	30.25470125	2.9123E-05	non-specific cytotoxic cell receptor protein 1 homolog (zebrafish) [Source:HGNC Symbol;Acc:33739]
ENSG00000246181	NOT FOUND	NOT FOUND	-3.6879147	3.47033184	44.72434098	1.6737E-06	NOT FOUND
ENSG00000248119	NOT FOUND	NOT FOUND	-3.5023431	40.2795097	456.4513623	0	NOT FOUND
ENSG00000253769	NOT FOUND	NOT FOUND	-3.4056424	2.23427698	23.67759228	0.00176639	NOT FOUND
ENSG00000248959	NOT FOUND	NOT FOUND	-3.2563912	1.2388996	11.83879614	0.03590843	NOT FOUND
ENSG00000239660	NOT FOUND	NOT FOUND	-3.1776507	1.59915121	14.46963973	0.01713475	NOT FOUND
ENSG00000248940	NOT FOUND	NOT FOUND	-2.8740382	1.61486362	11.83879614	0.02833588	NOT FOUND
ENSG00000244846	NOT FOUND	NOT FOUND	-2.522651	18.084167	103.9183217	4.091E-05	NOT FOUND
ENSG00000074181	protein_coding	NOTCH3	8.6317601	521.775578	1.315421793	1.66E-13	notch 3 [Source:HGNC Symbol;Acc:7883]
ENSG00000185269	protein_coding	NOTUM	-2.4167099	4.68076112	24.99301408	0.03590843	notum pectinacetylesterase homolog (Drosophila) [Source:HGNC Symbol;Acc:27106]

ENSG00000100968	protein_coding	NFATC4	5.5323342	121.756897	2.630843587	7.7962E-06	nuclear factor of activated T-cells, cytoplasmic, calcineurin-dependent 4 [Source:HGNC Symbol;Acc:7778]
ENSG00000176046	protein_coding	NUPR1	-2.2302453	28.0345966	131.5421793	0.00123803	nuclear protein, transcriptional regulator, 1 [Source:HGNC Symbol;Acc:29990]
ENSG00000130558	protein_coding	OLFM1	3.3275028	356.538918	35.51638842	5.7508E-05	olfactomedin 1 [Source:HGNC Symbol;Acc:17187]
ENSG00000185585	protein_coding	OLFML2A	2.1264163	315.895047	72.34819864	0.01855685	olfactomedin-like 2A [Source:HGNC Symbol;Acc:27270]
ENSG00000021762	protein_coding	OSBPL5	-2.2999929	155.193784	764.260062	2.329E-08	oxysterol binding protein-like 5 [Source:HGNC Symbol;Acc:16392]
ENSG00000007372	protein_coding	PAX6	3.9050986	98.5341699	6.577108967	0.00120372	paired box 6 [Source:HGNC Symbol;Acc:8620]
ENSG00000204174	protein_coding	PPYR1	-3.6946139	14.4265931	186.7898947	3.07E-13	pancreatic polypeptide receptor 1 [Source:HGNC Symbol;Acc:9329]
ENSG00000243444	protein_coding	PALM2	2.6003356	127.633312	21.0467487	0.01953723	paralemmin 2 [Source:HGNC Symbol;Acc:15845]
ENSG00000139946	protein_coding	PELI2	5.2686465	202.836294	5.261687174	4.2699E-07	pellino E3 ubiquitin protein ligase family member 2 [Source:HGNC Symbol;Acc:8828]
ENSG00000147872	protein_coding	PLIN2	Inf	42.9671212	0	0.00377537	perilipin 2 [Source:HGNC Symbol;Acc:248]
ENSG00000154217	protein_coding	PITPNC1	2.6361729	155.376644	24.99301408	0.00910255	phosphatidylinositol transfer protein, cytoplasmic 1 [Source:HGNC Symbol;Acc:21045]
ENSG00000107242	protein_coding	PIP5K1B	-3.6406331	3.58594971	44.72434098	1.6553E-05	phosphatidylinositol-4-phosphate 5-kinase, type I, beta [Source:HGNC Symbol;Acc:8995]
ENSG00000186642	protein_coding	PDE2A	8.4157508	2246.09682	6.577108967	2.8E-14	phosphodiesterase 2A, cGMP-stimulated [Source:HGNC Symbol;Acc:8777]
ENSG00000160191	protein_coding	PDE9A	-3.9126838	2.96967391	44.72434098	6.3029E-07	phosphodiesterase 9A [Source:HGNC Symbol;Acc:8795]
ENSG00000100889	protein_coding	PCK2	-2.248917	36.2530745	172.3202549	0.00013187	phosphoenolpyruvate carboxykinase 2 (mitochondrial) [Source:HGNC Symbol;Acc:8725]
ENSG00000165434	protein_coding	PGM2L1	2.6334625	228.546336	36.83181022	0.00350179	phosphoglucomutase 2-like 1 [Source:HGNC Symbol;Acc:20898]
ENSG00000153246	protein_coding	PLA2R1	2.2103207	176.537053	38.14723201	0.0298762	phospholipase A2 receptor 1, 180kDa [Source:HGNC Symbol;Acc:9042]
ENSG00000176485	protein_coding	PLA2G16	-2.9564539	51.517799	399.8882252	3.49E-12	phospholipase A2, group XVI [Source:HGNC Symbol;Acc:17825]
ENSG00000138193	protein_coding	PLCE1	4.6336448	65.3072047	2.630843587	0.00392312	phospholipase C, epsilon 1 [Source:HGNC Symbol;Acc:17175]
ENSG00000197943	protein_coding	PLCG2	-2.199828	5.15374958	23.67759228	0.044694	phospholipase C, gamma 2 (phosphatidylinositol-specific) [Source:HGNC Symbol;Acc:9066]
ENSG00000154864	protein_coding	PIEZO2	8.4362366	455.643618	1.315421793	6.47E-13	piezo-type mechanosensitive ion channel component 2 [Source:HGNC Symbol;Acc:26270]
ENSG00000223573	lincRNA	PLAC2	-3.620328	8.45015722	103.9183217	1.1353E-09	placenta-specific 2 (non-protein coding) [Source:HGNC Symbol;Acc:14607]
ENSG00000145287	protein_coding	PLAC8	-2.6582055	15.6288389	98.65663451	4.1769E-05	placenta-specific 8 [Source:HGNC Symbol;Acc:19254]
ENSG00000057294	protein_coding	PKP2	-2.9097876	28.8812255	217.0445959	1.6683E-09	plakophilin 2 [Source:HGNC Symbol;Acc:9024]
ENSG00000145431	protein_coding	PDGFC	2.5191509	203.595786	35.51638842	0.00960386	platelet derived growth factor C [Source:HGNC Symbol;Acc:8801]

ENSG00000156011	protein_coding	PSD3	4.447629	401.848924	18.41590511	2.3821E-07	pleckstrin and Sec7 domain containing 3 [Source:HGNC Symbol;Acc:19093]
ENSG00000105559	protein_coding	PLEKHA4	-2.2536665	3.86164069	18.41590511	0.04692274	pleckstrin homology domain containing, family A (phosphoinositide binding specific) member 4 [Source:HGNC Symbol;Acc:14339]
ENSG00000143850	protein_coding	PLEKHA6	-2.5229189	44.6298457	256.5072497	2.7247E-07	pleckstrin homology domain containing, family A member 6 [Source:HGNC Symbol;Acc:17053]
ENSG00000166689	protein_coding	PLEKHA7	-2.2973841	42.8157436	210.467487	1.1896E-05	pleckstrin homology domain containing, family A member 7 [Source:HGNC Symbol;Acc:27049]
ENSG00000120594	protein_coding	PLXDC2	3.853664	247.216153	17.10048332	2.2355E-05	plexin domain containing 2 [Source:HGNC Symbol;Acc:21013]
ENSG00000132429	protein_coding	POPDC3	3.9895291	41.7890933	2.630843587	0.04540258	popeye domain containing 3 [Source:HGNC Symbol;Acc:17649]
ENSG00000148985	protein_coding	PGAP2	-2.9673485	95.3646008	745.8441569	4E-15	post-GPI attachment to proteins 2 [Source:HGNC Symbol;Acc:17893]
ENSG00000178695	protein_coding	KCTD12	2.5068042	1330.78624	234.1450792	0.0039377	potassium channel tetramerisation domain containing 12 [Source:HGNC Symbol;Acc:14678]
ENSG00000151364	protein_coding	KCTD14	-3.1074312	3.05256566	26.30843587	0.00218562	potassium channel tetramerisation domain containing 14 [Source:HGNC Symbol;Acc:23295]
ENSG00000143473	protein_coding	KCNH1	3.2562137	75.4109264	7.892530761	0.02131745	potassium voltage-gated channel, subfamily H (eag-related), member 1 [Source:HGNC Symbol;Acc:6250]
ENSG00000126838	protein_coding	PZP	-3.4547489	66.2248621	726.11283	0	pregnancy-zone protein [Source:HGNC Symbol;Acc:9750]
ENSG00000137819	protein_coding	PAQR5	-2.0051733	106.823137	428.8275047	7.9936E-06	progestin and adipoQ receptor family member V [Source:HGNC Symbol;Acc:29645]
ENSG00000140479	protein_coding	PCSK6	-2.1883004	31.7478011	144.6963973	0.0001572	proprotein convertase subtilisin/kexin type 6 [Source:HGNC Symbol;Acc:8569]
ENSG00000088899	protein_coding	RP5-1187M17.10	-2.0928916	8.01706171	34.20096663	0.0430699	ProSAP-interacting protein 1 [Source:UniProtKB/Swiss-Prot;Acc:O60299]
ENSG00000171522	protein_coding	PTGER4	2.0548001	273.269715	65.77108967	0.03862207	prostaglandin E receptor 4 (subtype EP4) [Source:HGNC Symbol;Acc:9596]
ENSG00000225937	antisense	PCA3	Inf	39.2069546	0	0.00713557	prostate cancer antigen 3 (non-protein coding) [Source:HGNC Symbol;Acc:8637]
ENSG00000124225	protein_coding	PMEPA1	2.550431	570.233856	97.34121272	0.00166901	prostate transmembrane protein, androgen induced 1 [Source:HGNC Symbol;Acc:14107]
ENSG00000101000	protein_coding	PROCR	3.2961468	77.5274242	7.892530761	0.01166427	protein C receptor, endothelial [Source:HGNC Symbol;Acc:9452]
ENSG00000158528	protein_coding	PPP1R9A	-2.0639337	61.6616494	257.8226715	8.8724E-05	protein phosphatase 1, regulatory subunit 9A [Source:HGNC Symbol;Acc:14946]
ENSG00000074211	protein_coding	PPP2R2C	Inf	37.8735819	0	0.01239371	protein phosphatase 2, regulatory subunit B, gamma [Source:HGNC Symbol;Acc:9306]
ENSG00000111679	protein_coding	PTPN6	-2.7949831	4.16978779	28.93927946	0.00278731	protein tyrosine phosphatase, non-receptor type 6 [Source:HGNC Symbol;Acc:9658]
ENSG00000138650	protein_coding	PCDH10	-4.1152759	2.8842118	49.98602815	5.3878E-08	protocadherin 10 [Source:HGNC Symbol;Acc:13404]
ENSG00000184226	protein_coding	PCDH9	-2.9912195	10.5876165	84.18699478	1.8682E-06	protocadherin 9 [Source:HGNC Symbol;Acc:8661]
ENSG00000106772	protein_coding	PRUNE2	Inf	1096.96124	0	0	prune homolog 2 (Drosophila) [Source:HGNC Symbol;Acc:25209]
ENSG00000204540	protein_coding	PSORS1C1	-2.0262349	10.33374	42.09349739	0.032459	psoriasis susceptibility 1 candidate 1 [Source:HGNC Symbol;Acc:17202]

ENSG00000166387	protein_coding	PPFIBP2	-3.4840373	1.4107386	15.78506152	0.00392312	PTPRF interacting protein, binding protein 2 (liprin beta 2) [Source:HGNC Symbol;Acc:9250]
ENSG00000250305	protein_coding	KIAA1456	-4.1353825	1.94609579	34.20096663	2.3304E-06	Putative methyltransferase KIAA1456 [Source:UniProtKB/Swiss-Prot;Acc:Q9P272]
ENSG00000171631	protein_coding	P2RY6	-3.0515782	2.06248984	17.10048332	0.01545641	pyrimidinergic receptor P2Y, G-protein coupled, 6 [Source:HGNC Symbol;Acc:8543]
ENSG00000004799	protein_coding	PDK4	3.3342741	504.156243	49.98602815	3.2304E-05	pyruvate dehydrogenase kinase, isozyme 4 [Source:HGNC Symbol;Acc:8812]
ENSG00000124839	protein_coding	RAB17	-3.2743133	11.4203073	110.4954307	3.672E-08	RAB17, member RAS oncogene family [Source:HGNC Symbol;Acc:16523]
ENSG00000118508	protein_coding	RAB32	2.3044027	233.917052	47.35518456	0.00966898	RAB32, member RAS oncogene family [Source:HGNC Symbol;Acc:9772]
ENSG00000169213	protein_coding	RAB3B	5.1673449	47.2705703	1.315421793	0.00922173	RAB3B, member RAS oncogene family [Source:HGNC Symbol;Acc:9778]
ENSG00000134321	protein_coding	RSAD2	-3.174345	2.76850661	24.99301408	0.00105993	radical S-adenosyl methionine domain containing 2 [Source:HGNC Symbol;Acc:30908]
ENSG00000165105	protein_coding	RASEF	Inf	40.9839371	0	0.00625926	RAS and EF-hand domain containing [Source:HGNC Symbol;Acc:26464]
ENSG00000136653	protein_coding	RASSF5	-2.9778965	26.7146098	210.467487	1.1143E-09	Ras association (RalGDS/AF-6) domain family member 5 [Source:HGNC Symbol;Acc:17609]
ENSG00000177105	protein_coding	RHOG	-2.5961127	79.6229594	481.4443764	8.5314E-10	ras homolog family member G [Source:HGNC Symbol;Acc:672]
ENSG00000116574	protein_coding	RHOU	-2.5316838	4.77717939	27.62385766	0.01937693	ras homolog family member U [Source:HGNC Symbol;Acc:17794]
ENSG00000104140	protein_coding	RHOV	-3.0910282	8.33617055	71.03277685	4.829E-06	ras homolog family member V [Source:HGNC Symbol;Acc:18313]
ENSG00000143333	protein_coding	RGS16	-2.2857173	6.74427967	32.88554484	0.02077583	regulator of G-protein signaling 16 [Source:HGNC Symbol;Acc:9997]
ENSG00000186907	protein_coding	RTN4RL2	-4.0818451	1.16519463	19.7313269	0.00035281	reticulon 4 receptor-like 2 [Source:HGNC Symbol;Acc:23053]
ENSG00000039560	protein_coding	RAI14	2.5396347	994.293525	171.0048332	0.00208974	retinoic acid induced 14 [Source:HGNC Symbol;Acc:14873]
ENSG00000133321	protein_coding	RARRES3	-3.0098079	2.61301889	21.0467487	0.00484685	retinoic acid receptor responder (tazarotene induced) 3 [Source:HGNC Symbol;Acc:9869]
ENSG00000031081	protein_coding	ARHGAP31	5.2786208	51.0608727	1.315421793	0.00689948	Rho GTPase activating protein 31 [Source:HGNC Symbol;Acc:29216]
ENSG00000089820	protein_coding	ARHGAP4	-2.7837992	2.1011191	14.46963973	0.04742567	Rho GTPase activating protein 4 [Source:HGNC Symbol;Acc:674]
ENSG00000129667	protein_coding	RHBDF2	-2.4877268	15.4784893	86.81783837	0.00061099	rhomboid 5 homolog 2 (Drosophila) [Source:HGNC Symbol;Acc:20788]
ENSG00000071242	protein_coding	RPS6KA2	7.9199413	318.570036	1.315421793	3.4585E-11	ribosomal protein S6 kinase, 90kDa, polypeptide 2 [Source:HGNC Symbol;Acc:10431]
ENSG00000101695	protein_coding	RNF125	-2.7961486	4.16642064	28.93927946	0.00574489	ring finger protein 125, E3 ubiquitin protein ligase [Source:HGNC Symbol;Acc:21150]
ENSG00000137393	protein_coding	RNF144B	-2.4990859	9.77269858	55.24771533	0.00278731	ring finger protein 144B [Source:HGNC Symbol;Acc:21578]
ENSG00000178222	protein_coding	RNF212	Inf	102.866047	0	1.9693E-06	ring finger protein 212 [Source:HGNC Symbol;Acc:27729]
ENSG00000248124	pseudogene	RRN3P1	Inf	48.2154502	0	0.00201564	RNA polymerase I transcription factor homolog (S. cerevisiae) pseudogene 1 [Source:HGNC Symbol;Acc:30548]

ENSG00000238304	snRNA	RNU7-50P	-3.2555353	1.37737199	13.15421793	0.02159979	RNA, U7 small nuclear 50 pseudogene [Source:HGNC Symbol;Acc:34146]
ENSG00000020633	protein_coding	RUNX3	6.4909174	118.311206	1.315421793	2.7261E-06	runt-related transcription factor 3 [Source:HGNC Symbol;Acc:10473]
ENSG00000196754	protein_coding	S100A2	2.7233949	95.5611266	14.46963973	0.01855685	S100 calcium binding protein A2 [Source:HGNC Symbol;Acc:10492]
ENSG00000163993	protein_coding	S100P	-3.7062509	4.43429944	57.87855891	1.5354E-06	S100 calcium binding protein P [Source:HGNC Symbol;Acc:10504]
ENSG00000168079	protein_coding	SCARA5	-3.2728726	3.5383888	34.20096663	0.00058115	scavenger receptor class A, member 5 (putative) [Source:HGNC Symbol;Acc:28701]
ENSG00000172716	protein_coding	SLFN11	-3.5527752	20.7369483	243.3530318	1.6E-14	schlafen family member 11 [Source:HGNC Symbol;Acc:26633]
ENSG00000006747	protein_coding	SCIN	-3.574517	14.353954	171.0048332	6.588E-12	scinderin [Source:HGNC Symbol;Acc:21695]
ENSG00000133488	protein_coding	SEC14L4	-3.6623265	1.35063578	17.10048332	0.00418964	SEC14-like 4 (S. cerevisiae) [Source:HGNC Symbol;Acc:20627]
ENSG00000075213	protein_coding	SEMA3A	3.6827632	168.922391	13.15421793	0.00017283	sema domain, immunoglobulin domain (Ig), short basic domain, secreted, (semaphorin) 3A [Source:HGNC Symbol;Acc:10723]
ENSG00000122862	protein_coding	SRGN	Inf	150.128484	0	2.3016E-08	serglycin [Source:HGNC Symbol;Acc:9361]
ENSG00000178172	protein_coding	SPINK6	4.3616132	1703.65627	82.87157299	2.0805E-07	serine peptidase inhibitor, Kazal type 6 [Source:HGNC Symbol;Acc:29486]
ENSG00000152953	protein_coding	STK32B	Inf	33.4562418	0	0.01891156	serine/threonine kinase 32B [Source:HGNC Symbol;Acc:14217]
ENSG00000130413	protein_coding	STK33	Inf	48.3670242	0	0.00201564	serine/threonine kinase 33 [Source:HGNC Symbol;Acc:14568]
ENSG00000170542	protein_coding	SERPINB9	-2.1285807	14.7398978	64.45566788	0.00706935	serpin peptidase inhibitor, clade B (ovalbumin), member 9 [Source:HGNC Symbol;Acc:8955]
ENSG00000106366	protein_coding	SERPINE1	2.4998233	520.816652	92.07952554	0.00280785	serpin peptidase inhibitor, clade E (nexin, plasminogen activator inhibitor type 1), member 1 [Source:HGNC Symbol;Acc:8583]
ENSG00000082497	protein_coding	SERTAD4	-2.7751659	4.80393217	32.88554484	0.0020365	SERTA domain containing 4 [Source:HGNC Symbol;Acc:25236]
ENSG00000149212	protein_coding	SESN3	Inf	415.024915	0	1.91E-13	sestrin 3 [Source:HGNC Symbol;Acc:23060]
ENSG00000144681	protein_coding	STAC	3.1761503	154.569891	17.10048332	0.00161399	SH3 and cysteine rich domain [Source:HGNC Symbol;Acc:11353]
ENSG00000154447	protein_coding	SH3RF1	2.2539789	232.157121	48.67060636	0.01935274	SH3 domain containing ring finger 1 [Source:HGNC Symbol;Acc:17650]
ENSG00000172985	protein_coding	SH3RF3	-3.2443283	12.6318302	119.7033832	4.2814E-08	SH3 domain containing ring finger 3 [Source:HGNC Symbol;Acc:24699]
ENSG00000170786	protein_coding	SDR16C5	-2.1062351	13.7479393	59.19398071	0.00866945	short chain dehydrogenase/reductase family 16C, member 5 [Source:HGNC Symbol;Acc:30311]
ENSG00000198053	protein_coding	SIRPA	Inf	31.9591212	0	0.02142213	signal-regulatory protein alpha [Source:HGNC Symbol;Acc:9662]
ENSG00000179542	protein_coding	SLITRK4	Inf	108.350065	0	1.0303E-06	SLIT and NTRK-like family, member 4 [Source:HGNC Symbol;Acc:23502]
ENSG00000196876	protein_coding	SCN8A	-3.7828793	13.1881528	181.5282075	4E-14	sodium channel, voltage gated, type VIII, alpha subunit [Source:HGNC Symbol;Acc:10596]
ENSG00000144285	protein_coding	SCN1A	-2.2965503	25.4365437	124.9650704	0.00022584	sodium channel, voltage-gated, type I, alpha subunit [Source:HGNC Symbol;Acc:10585]

ENSG00000106688	protein_coding	SLC1A1	3.8649641	76.6646476	5.261687174	0.0047816	solute carrier family 1 (neuronal/epithelial high affinity glutamate transporter, system Xag), member 1 [Source:HGNC Symbol;Acc:10939]
ENSG00000113504	protein_coding	SLC12A7	4.7307183	69.8526923	2.630843587	0.00207361	solute carrier family 12 (potassium/chloride transporters), member 7 [Source:HGNC Symbol;Acc:10915]
ENSG00000070915	protein_coding	SLC12A3	-3.5318321	35.4839706	410.4115996	0	solute carrier family 12 (sodium/chloride transporters), member 3 [Source:HGNC Symbol;Acc:10912]
ENSG00000174327	protein_coding	SLC16A13	-2.6052469	2.59411251	15.78506152	0.04213619	solute carrier family 16, member 13 (monocarboxylic acid transporter 13) [Source:HGNC Symbol;Acc:31037]
ENSG00000146411	protein_coding	SLC2A12	3.3192916	78.7812025	7.892530761	0.01306583	solute carrier family 2 (facilitated glucose transporter), member 12 [Source:HGNC Symbol;Acc:18067]
ENSG00000059804	protein_coding	SLC2A3	-2.7254077	249.023248	1646.908085	3E-15	solute carrier family 2 (facilitated glucose transporter), member 3 [Source:HGNC Symbol;Acc:11007]
ENSG00000160326	protein_coding	SLC2A6	4.7823233	72.3965375	2.630843587	0.00164107	solute carrier family 2 (facilitated glucose transporter), member 6 [Source:HGNC Symbol;Acc:11011]
ENSG00000170482	protein_coding	SLC23A1	-2.3265722	4.720299	23.67759228	0.04746296	solute carrier family 23 (nucleobase transporters), member 1 [Source:HGNC Symbol;Acc:10974]
ENSG00000197506	protein_coding	SLC28A3	-3.5935139	0.87176526	10.52337435	0.00788913	solute carrier family 28 (sodium-coupled nucleoside transporter), member 3 [Source:HGNC Symbol;Acc:16484]
ENSG00000168917	protein_coding	SLC35G2	2.6052263	136.070883	22.36217049	0.01378713	solute carrier family 35, member G2 [Source:HGNC Symbol;Acc:28480]
ENSG00000188338	lincRNA	SLC38A3	-2.7927717	2.27791876	15.78506152	0.02623094	solute carrier family 38, member 3 [Source:HGNC Symbol;Acc:18044]
ENSG00000165915	protein_coding	SLC39A13	2.4097616	167.759373	31.57012304	0.01741662	solute carrier family 39 (zinc transporter), member 13 [Source:HGNC Symbol;Acc:20859]
ENSG00000149150	protein_coding	SLC43A1	-2.554163	3.13554751	18.41590511	0.03590843	solute carrier family 43, member 1 [Source:HGNC Symbol;Acc:9225]
ENSG00000063127	protein_coding	SLC6A16	-3.4332644	2.31367873	24.99301408	0.00096166	solute carrier family 6, member 16 [Source:HGNC Symbol;Acc:13622]
ENSG00000137642	protein_coding	SORL1	4.7638161	357.368757	13.15421793	7.557E-08	sortilin-related receptor, L(DLR class) A repeats containing [Source:HGNC Symbol;Acc:11185]
ENSG00000184985	protein_coding	SORCS2	-2.2237443	7.04030234	32.88554484	0.02429036	sortilin-related VPS10 domain containing receptor 2 [Source:HGNC Symbol;Acc:16698]
ENSG00000086300	protein_coding	SNX10	-3.9154206	2.70251239	40.7780756	2.6358E-06	sorting nexin 10 [Source:HGNC Symbol;Acc:14974]
ENSG00000178996	protein_coding	SNX18	2.0552844	213.221943	51.30144995	0.04797686	sorting nexin 18 [Source:HGNC Symbol;Acc:19245]
ENSG00000152377	protein_coding	SPOCK1	2.8926451	1142.94103	153.9043498	0.00048526	sparc/osteonectin, cwcv and kazal-like domains proteoglycan (testican) 1 [Source:HGNC Symbol;Acc:11251]
ENSG00000088826	protein_coding	SMOX	-2.0981775	22.1198997	94.71036913	0.00270012	spermine oxidase [Source:HGNC Symbol;Acc:15862]
ENSG00000176170	protein_coding	SPHK1	3.6499558	511.885593	40.7780756	4.6751E-06	sphingosine kinase 1 [Source:HGNC Symbol;Acc:11240]
ENSG00000134668	protein_coding	SPOCD1	6.4074816	223.325941	2.630843587	1.1502E-08	SPOC domain containing 1 [Source:HGNC Symbol;Acc:26338]
ENSG00000164056	protein_coding	SPRY1	-3.3865771	2.26399916	23.67759228	0.00099663	sprouty homolog 1, antagonist of FGF signaling (Drosophila) [Source:HGNC Symbol;Acc:11269]
ENSG00000017373	protein_coding	SRCIN1	-3.1765544	1.30939112	11.83879614	0.03279342	SRC kinase signaling inhibitor 1 [Source:HGNC Symbol;Acc:29506]
ENSG00000127954	protein_coding	STEAP4	-2.7187568	127.884423	841.8699478	7.4E-14	STEAP family member 4 [Source:HGNC Symbol;Acc:21923]

ENSG00000187634	protein_coding	SAMD11	2.9201097	149.347142	19.7313269	0.00339229	sterile alpha motif domain containing 11 [Source:HGNC Symbol;Acc:28706]
ENSG00000165730	protein_coding	STOX1	-3.496642	0.93231117	10.52337435	0.0313127	storkhead box 1 [Source:HGNC Symbol;Acc:23508]
ENSG00000090402	protein_coding	SI	Inf	28.3731574	0	0.0423781	sucrase-isomaltase (alpha-glucosidase) [Source:HGNC Symbol;Acc:10856]
ENSG00000099994	protein_coding	SUSD2	-3.1050669	6.42090209	55.24771533	0.00019023	sushi domain containing 2 [Source:HGNC Symbol;Acc:30667]
ENSG00000165124	protein_coding	SVEP1	-3.3789978	101.910841	1060.229966	0	sushi, von Willebrand factor type A, EGF and pentraxin domain containing 1 [Source:HGNC Symbol;Acc:15985]
ENSG00000143858	protein_coding	SYT2	-2.7970483	5.29941134	36.83181022	0.00219214	synaptotagmin II [Source:HGNC Symbol;Acc:11510]
ENSG00000147041	protein_coding	SYTL5	Inf	36.3994809	0	0.01064568	synaptotagmin-like 5 [Source:HGNC Symbol;Acc:15589]
ENSG00000164506	protein_coding	STXBP5	2.0337103	1308.81756	319.6474958	0.02909901	syntaxin binding protein 5 (tomosyn) [Source:HGNC Symbol;Acc:19665]
ENSG00000145335	protein_coding	SNCA	5.0182133	42.6282748	1.315421793	0.01486073	synuclein, alpha (non A4 component of amyloid precursor) [Source:HGNC Symbol;Acc:11138]
ENSG00000135111	protein_coding	TBX3	-2.596706	151.134439	914.2181465	2.308E-12	T-box 3 [Source:HGNC Symbol;Acc:11602]
ENSG00000164458	protein_coding	T	Inf	45.5629376	0	0.00268197	T, brachyury homolog (mouse) [Source:HGNC Symbol;Acc:11515]
ENSG00000135090	protein_coding	TAOK3	-2.2223817	57.5031504	268.3460459	1.3864E-05	TAO kinase 3 [Source:HGNC Symbol;Acc:18133]
ENSG00000041982	protein_coding	TNC	2.5249343	295.264073	51.30144995	0.0043879	tenascin C [Source:HGNC Symbol;Acc:5318]
ENSG00000168477	protein_coding	TNXB	-2.2545088	6.89176175	32.88554484	0.02931263	tenascin XB [Source:HGNC Symbol;Acc:11976]
ENSG00000079308	protein_coding	TNS1	-2.5717533	136.955919	814.2460902	7.024E-12	tensin 1 [Source:HGNC Symbol;Acc:11973]
ENSG00000157570	protein_coding	TSPAN18	Inf	29.4629305	0	0.03222865	tetraspanin 18 [Source:HGNC Symbol;Acc:20660]
ENSG00000117289	protein_coding	TXNIP	-2.473538	86.9204501	482.7597982	9.1746E-09	thioredoxin interacting protein [Source:HGNC Symbol;Acc:16952]
ENSG00000154096	protein_coding	THY1	Inf	69.2543337	0	0.00010935	Thy-1 cell surface antigen [Source:HGNC Symbol;Acc:11801]
ENSG00000198846	protein_coding	TOX	5.7416863	70.3857666	1.315421793	0.00062423	thymocyte selection-associated high mobility group box [Source:HGNC Symbol;Acc:18988]
ENSG00000105825	protein_coding	TFPI2	4.7691853	107.610372	3.94626538	0.00010396	tissue factor pathway inhibitor 2 [Source:HGNC Symbol;Acc:11761]
ENSG00000050730	protein_coding	TNIP3	Inf	33.3576533	0	0.02073178	TNFAIP3 interacting protein 3 [Source:HGNC Symbol;Acc:19315]
ENSG00000136869	protein_coding	TLR4	2.9064205	226.83634	30.25470125	0.00175888	toll-like receptor 4 [Source:HGNC Symbol;Acc:11850]
ENSG00000196628	protein_coding	TCF4	-4.7253294	0.59673348	15.78506152	5.0691E-05	transcription factor 4 [Source:HGNC Symbol;Acc:11634]
ENSG00000137203	protein_coding	TFAP2A	-2.255838	136.606641	652.4492096	2.8131E-08	transcription factor AP-2 alpha (activating enhancer binding protein 2 alpha) [Source:HGNC Symbol;Acc:11742]
ENSG00000065717	protein_coding	TLE2	-3.1254888	1.50729815	13.15421793	0.01751071	transducin-like enhancer of split 2 (E(sp1) homolog, Drosophila) [Source:HGNC Symbol;Acc:11838]

ENSG00000140682	protein_coding	TGFB1I1	3.4263557	98.9917331	9.207952554	0.00478396	transforming growth factor beta 1 induced transcript 1 [Source:HGNC Symbol;Acc:11767]
ENSG00000092969	protein_coding	TGFB2	3.084763	245.523973	28.93927946	0.00066228	transforming growth factor, beta 2 [Source:HGNC Symbol;Acc:11768]
ENSG00000120708	protein_coding	TGFBI	3.5474135	7382.17172	631.4024609	0.01439638	transforming growth factor, beta-induced, 68kDa [Source:HGNC Symbol;Acc:11771]
ENSG00000103534	protein_coding	TMC5	-3.3000181	22.9714835	226.2525485	1.48E-11	transmembrane channel-like 5 [Source:HGNC Symbol;Acc:22999]
ENSG00000141524	protein_coding	TMC6	-2.2164625	9.62326093	44.72434098	0.01428001	transmembrane channel-like 6 [Source:HGNC Symbol;Acc:18021]
ENSG00000215367	pseudogene	TMED11P	Inf	41.0906863	0	0.00563174	transmembrane emp24 protein transport domain containing 11, pseudogene [Source:HGNC Symbol;Acc:35401]
ENSG00000166157	protein_coding	TPTE	-3.4523109	2.8842118	31.57012304	5.9111E-05	transmembrane phosphatase with tensin homology [Source:HGNC Symbol;Acc:12023]
ENSG00000160183	protein_coding	TMPRSS3	-2.0634328	46.5770068	194.6824254	0.00015271	transmembrane protease, serine 3 [Source:HGNC Symbol;Acc:11877]
ENSG00000170006	protein_coding	TMEM154	Inf	33.8091297	0	0.01551972	transmembrane protein 154 [Source:HGNC Symbol;Acc:26489]
ENSG00000249992	protein_coding	TMEM158	4.5540441	61.8014838	2.630843587	0.00416029	transmembrane protein 158 (gene/pseudogene) [Source:HGNC Symbol;Acc:30293]
ENSG00000101255	protein_coding	TRIB3	-3.0646401	74.5237647	623.5099301	1.2E-14	tribbles homolog 3 (Drosophila) [Source:HGNC Symbol;Acc:16228]
ENSG00000108448	protein_coding	TRIM16L	2.3779227	464.943318	89.44868196	0.00330617	tripartite motif containing 16-like [Source:HGNC Symbol;Acc:32670]
ENSG00000137699	protein_coding	TRIM29	-3.1326956	72.28975	634.0333045	0	tripartite motif containing 29 [Source:HGNC Symbol;Acc:17274]
ENSG00000167333	protein_coding	TRIM68	-2.1345886	17.3747206	76.29446402	0.00611583	tripartite motif containing 68 [Source:HGNC Symbol;Acc:21161]
ENSG00000179046	protein_coding	TRIML2	-4.6283846	0.42547342	10.52337435	0.00163453	tripartite motif family-like 2 [Source:HGNC Symbol;Acc:26378]
ENSG00000140416	protein_coding	TPM1	2.1149393	2096.87724	484.07522	0.03346424	tropomyosin 1 (alpha) [Source:HGNC Symbol;Acc:12010]
ENSG00000166402	protein_coding	TUB	3.4501638	258.785732	23.67759228	0.00010422	tubby homolog (mouse) [Source:HGNC Symbol;Acc:12406]
ENSG00000156414	protein_coding	TDRD9	Inf	155.906056	0	1.8291E-08	tudor domain containing 9 [Source:HGNC Symbol;Acc:20122]
ENSG00000181634	protein_coding	TNFSF15	-3.3935641	6.38367285	67.08651147	2.732E-06	tumor necrosis factor (ligand) superfamily, member 15 [Source:HGNC Symbol;Acc:11931]
ENSG00000164761	protein_coding	TNFRSF11B	Inf	97.8067041	0	2.7251E-06	tumor necrosis factor receptor superfamily, member 11b [Source:HGNC Symbol;Acc:11909]
ENSG00000127863	protein_coding	TNFRSF19	Inf	65.7414703	0	0.00015335	tumor necrosis factor receptor superfamily, member 19 [Source:HGNC Symbol;Acc:11915]
ENSG00000104723	protein_coding	TUSC3	6.3577751	323.64381	3.94626538	4.9018E-10	tumor suppressor candidate 3 [Source:HGNC Symbol;Acc:30242]
ENSG00000169902	protein_coding	TPST1	3.0386062	226.984359	27.62385766	0.00069354	tyrosylprotein sulfotransferase 1 [Source:HGNC Symbol;Acc:12020]
ENSG00000202077	snRNA	U1	-2.6901805	2.44580179	15.78506152	0.02494498	U1 spliceosomal RNA [Source:RFAM;Acc:RF00003]
ENSG00000036672	protein_coding	USP2	-2.3322176	6.26915213	31.57012304	0.0228987	ubiquitin specific peptidase 2 [Source:HGNC Symbol;Acc:12618]

ENSG00000139629	protein_coding	GALNT6	-3.4467265	13.0292897	142.0655537	4.5602E-10	UDP-N-acetyl-alpha-D-galactosamine:polypeptide N-acetylgalactosaminyltransferase 6 (GalNAc-T6) [Source:HGNC
ENSG00000092929	protein_coding	UNC13D	2.5183831	504.950372	88.13326016	0.00224518	unc-13 homolog D (C. elegans) [Source:HGNC Symbol;Acc:23147]
ENSG00000107731	protein_coding	UNC5B	-3.7714969	1.44484947	19.7313269	0.00050146	unc-5 homolog B (C. elegans) [Source:HGNC Symbol;Acc:12568]
ENSG00000253250	protein_coding	RP11-122A3.2	Inf	31.4890866	0	0.02168606	uncharacterized protein LOC100127983 [Source:RefSeq peptide;Acc:NP_001177901]
ENSG00000176515	protein_coding	AL033381.1	-2.5049748	3.4760318	19.7313269	0.04176263	Uncharacterized protein; cDNA FLJ34594 fis, clone KIDNE2009109 [Source:UniProtKB/TrEMBL;Acc:Q8NAX6]
ENSG00000197813	protein_coding	AC011450.1	-3.4010691	1.61876781	17.10048332	0.00459656	Uncharacterized protein; cDNA FLJ40032 fis, clone STOMA2009256 [Source:UniProtKB/TrEMBL;Acc:Q8N843]
ENSG00000065361	protein_coding	ERBB3	-2.7753383	4.41908949	30.25470125	0.00520944	v-erb-b2 erythroblastic leukemia viral oncogene homolog 3 (avian) [Source:HGNC Symbol;Acc:3431]
ENSG00000150630	protein_coding	VEGFC	2.5904136	110.913718	18.41590511	0.03051445	vascular endothelial growth factor C [Source:HGNC Symbol;Acc:12682]
ENSG00000168140	protein_coding	VASN	2.7374624	263.174971	39.4626538	0.00179898	vasorin [Source:HGNC Symbol;Acc:18517]
ENSG00000206538	protein_coding	VGLL3	-2.1664799	217.123983	974.727549	3.1611E-08	vestigial like 3 (Drosophila) [Source:HGNC Symbol;Acc:24327]
ENSG00000205221	protein_coding	VIT	-3.3658066	5.74207405	59.19398071	1.8682E-06	vitrin [Source:HGNC Symbol;Acc:12697]
ENSG00000110002	protein_coding	VWA5A	-2.884171	30.1113165	222.3062831	8.1794E-09	von Willebrand factor A domain containing 5A [Source:HGNC Symbol;Acc:6658]
ENSG00000187667	pseudogene	WHAMMP3	-3.0900238	1.6992914	14.46963973	0.01242598	WAS protein homolog associated with actin, golgi membranes and microtubules pseudogene 3 [Source:HGNC Symbol;Acc:27892]
ENSG00000243710	protein_coding	WDR65	-3.2750029	1.63069288	15.78506152	0.01040909	WD repeat domain 65 [Source:HGNC Symbol;Acc:26485]
ENSG00000166415	protein_coding	WDR72	Inf	44.1132082	0	0.00373297	WD repeat domain 72 [Source:HGNC Symbol;Acc:26790]
ENSG00000103489	protein_coding	XYLT1	-3.3798478	1.13729195	11.83879614	0.00689948	xylosyltransferase I [Source:HGNC Symbol;Acc:15516]
ENSG00000199366	misc_RNA	Y_RNA	-3.3560198	2.05552326	21.0467487	0.00263448	Y RNA [Source:RFAM;Acc:RF00019]
ENSG00000207286	misc_RNA	Y_RNA	-2.5934613	2.61539104	15.78506152	0.03399288	Y RNA [Source:RFAM;Acc:RF00019]
ENSG00000137693	protein_coding	YAP1	-2.1505441	1149.22884	5102.521137	5.8531E-08	Yes-associated protein 1 [Source:HGNC Symbol;Acc:16262]
ENSG00000197020	protein_coding	ZNF100	6.2714566	101.615879	1.315421793	1.7027E-05	zinc finger protein 100 [Source:HGNC Symbol;Acc:12880]
ENSG00000165512	protein_coding	ZNF22	Inf	39.9417848	0	0.00526264	zinc finger protein 22 (KOX 15) [Source:HGNC Symbol;Acc:13012]
ENSG00000185947	protein_coding	ZNF267	2.4926035	318.333174	56.56313712	0.00502019	zinc finger protein 267 [Source:HGNC Symbol;Acc:13060]
ENSG00000198538	protein_coding	ZNF28	6.125591	91.8441872	1.315421793	5.2342E-05	zinc finger protein 28 [Source:HGNC Symbol;Acc:13073]
ENSG00000182986	protein_coding	ZNF320	6.0482574	87.0506323	1.315421793	7.1904E-05	zinc finger protein 320 [Source:HGNC Symbol;Acc:13842]
ENSG00000138311	protein_coding	ZNF365	Inf	57.0843715	0	0.00063247	zinc finger protein 365 [Source:HGNC Symbol;Acc:18194]

ENSG00000186918	protein_coding	ZNF395	-2.9778845	18.7003833	147.3272409	1.0703E-07	zinc finger protein 395 [Source:HGNC Symbol;Acc:18737]
ENSG00000204604	protein_coding	ZNF468	2.5738546	93.9839572	15.78506152	0.03996331	zinc finger protein 468 [Source:HGNC Symbol;Acc:33105]
ENSG00000225614	protein_coding	ZNF469	Inf	145.129866	0	5.5618E-08	zinc finger protein 469 [Source:HGNC Symbol;Acc:23216]
ENSG00000173258	protein_coding	ZNF483	Inf	28.6708268	0	0.04375892	zinc finger protein 483 [Source:HGNC Symbol;Acc:23384]
ENSG00000198795	protein_coding	ZNF521	-2.573041	14.5897027	86.81783837	0.00015914	zinc finger protein 521 [Source:HGNC Symbol;Acc:24605]
ENSG00000203326	protein_coding	ZNF525	Inf	60.0706817	0	0.00038033	zinc finger protein 525 [Source:HGNC Symbol;Acc:29423]
ENSG00000245680	protein_coding	ZNF585B	Inf	28.2617727	0	0.04066168	zinc finger protein 585B [Source:HGNC Symbol;Acc:30948]
ENSG00000167562	protein_coding	ZNF701	Inf	35.0810541	0	0.01495304	zinc finger protein 701 [Source:HGNC Symbol;Acc:25597]
ENSG00000227124	protein_coding	ZNF717	Inf	30.9839505	0	0.02979131	zinc finger protein 717 [Source:HGNC Symbol;Acc:29448]
ENSG00000167766	protein_coding	ZNF83	3.3190206	78.7664096	7.892530761	0.01268897	zinc finger protein 83 [Source:HGNC Symbol;Acc:13158]
ENSG00000235786	sense_intronic	ZNRF3-IT1	-3.2208097	1.41092748	13.15421793	0.00939541	ZNRF3 intronic transcript 1 (non-protein coding) [Source:HGNC Symbol;Acc:41440]
ENSG00000231419	lincRNA	AC004863.6	-2.4033153	10.1948046	53.93229353	0.00566403	
ENSG00000240350	lincRNA	AC017002.1	-2.0649202	19.806268	82.87157299	0.00538379	
ENSG00000181495	protein_coding	AC026703.1	4.7932712	583.584053	21.0467487	1.1395E-08	
ENSG00000213963	processed_transcript	AC074286.1	-2.1318707	12.6054191	55.24771533	0.01495304	
ENSG00000215196	lincRNA	AC091878.1	-2.0506443	9.52534783	39.4626538	0.03767869	
ENSG00000227189	antisense	AC092535.3	Inf	41.1142541	0	0.00585057	
ENSG00000253864	lincRNA	AC131025.8	Inf	36.9839718	0	0.01191974	
ENSG00000225746	lincRNA	AL132709.5	Inf	40.9156751	0	0.00578886	
ENSG00000225745	processed_transcript	AL773572.7	-2.6765595	34.1545378	218.3600177	1.4592E-07	
ENSG00000255414	lincRNA	AP000783.2	-3.0606016	24.7532264	206.5212216	2.5904E-09	
ENSG00000249647	processed_transcript	CTC-349C3.2	-3.4649685	1.07213157	11.83879614	0.02361883	
ENSG00000246082	pseudogene	NUDT16P	Inf	28.8512698	0	0.04195639	
ENSG00000229950	antisense	RP1-290I10.6	-3.233076	4.33684467	40.7780756	0.00038452	
ENSG00000258285	lincRNA	RP11-103B5.2	-3.227635	1.54469515	14.46963973	0.02332455	

ENSG00000237248	lincRNA	RP11-118B22.2	-2.8550738	2.90885561	21.0467487	0.03540604
ENSG00000250208	lincRNA	RP11-143E21.7	-2.7456797	16.6706303	111.8108524	1.1701E-05
ENSG00000254290	lincRNA	RP11-150O12.3	-4.1108783	0.60905618	10.52337435	0.00266616
ENSG00000249306	processed_transcript	RP11-267A15.1	4.3202308	105.110011	5.261687174	0.00031534
ENSG00000254101	lincRNA	RP11-30J20.1	-3.0035033	3.44460758	27.62385766	0.00212505
ENSG00000229098	antisense	RP11-348G8.3	-3.7740244	0.76923757	10.52337435	0.00420108
ENSG00000256893	antisense	RP11-392P7.6	-2.1969937	8.03270927	36.83181022	0.02979131
ENSG00000233052	sense_intronic	RP11-398B16.2	-4.1403669	0.59673348	10.52337435	0.00182518
ENSG00000225778	antisense	RP11-401F24.4	-2.923866	3.46675911	26.30843587	0.00465126
ENSG00000227953	processed_transcript	RP11-439E19.3	-3.2774165	1.627967	15.78506152	0.00496803
ENSG00000257496	processed_transcript	RP11-474P2.4	-2.1369987	5.98128262	26.30843587	0.03035427
ENSG00000253972	lincRNA	RP11-4K16.2	-3.5279109	1.48252285	17.10048332	0.00264283
ENSG00000236163	sense_intronic	RP11-501I18.2	-2.4818724	4.47404745	24.99301408	0.03941808
ENSG00000214145	lincRNA	RP11-513G11.1	-3.3822352	4.41548922	46.03976277	1.9045E-05
ENSG00000237781	antisense	RP11-54A4.2	-3.24326	2.22262647	21.0467487	0.00205161
ENSG00000223617	lincRNA	RP11-54H7.2	-3.3391636	1.2998003	13.15421793	0.01258814
ENSG00000254945	pseudogene	RP11-556O5.3	-2.6562657	3.96463302	24.99301408	0.0148241
ENSG00000254967	processed_transcript	RP11-680F20.6	-3.5942094	0.98026323	11.83879614	0.00507267
ENSG00000249395	lincRNA	RP11-697M17.1	-3.8888281	2.48638948	36.83181022	2.6433E-06
ENSG00000244128	lincRNA	RP11-85M11.2	-5.0472575	0.59673348	19.7313269	4.053E-06
ENSG00000247287	lincRNA	RP11-902B17.1	-2.7765623	6.71899831	46.03976277	0.00198502
ENSG00000232803	antisense	RP11-93B14.5	-2.5841851	6.14194385	36.83181022	0.00566403
ENSG00000255367	processed_transcript	RP13-726E6.2	-3.462295	2.38693391	26.30843587	0.00016077
ENSG00000254872	lincRNA	RP13-870H17.3	-3.6070672	1.94313113	23.67759228	0.00056231
ENSG00000257531	pseudogene	RP3-405J10.2	-2.8219895	10.9752284	77.60988581	0.00019289

ENSG00000213742	antisense	RP4-694B14.5	-2.0476547	10.8177878	44.72434098	0.04106965	
ENSG00000227964	lincRNA	RP5-1112F19.2	-4.3435622	1.16625909	23.67759228	1.1896E-05	

(b)

Gene ID	Feature	Gene Symbol	Log Fold Change	Read Count (1hpi	Read Count (1 hpi mock)	FDR	Gene Description
ENSG00000131016	protein_coding	AKAP12	4.8899928	173.5199	5.852140868	0.00065963	A kinase (PRKA) anchor protein 12 [Source:HGNC Symbol;Acc:370]
ENSG00000135074	protein_coding	ADAM19	5.5291203	62.3625309	1.350494046	0.0028429	ADAM metallopeptidase domain 19 [Source:HGNC Symbol;Acc:197]
ENSG00000156140	protein_coding	ADAMTS3	Inf	16.0121471	0	0.0472617	ADAM metallopeptidase with thrombospondin type 1 motif, 3 [Source:HGNC Symbol;Acc:219]
ENSG00000049192	protein_coding	ADAMTS6	5.2400386	153.11676	4.051482139	0.00025194	ADAM metallopeptidase with thrombospondin type 1 motif, 6 [Source:HGNC Symbol;Acc:222]
ENSG00000150594	protein_coding	ADRA2A	Inf	24.2220286	0	0.0186754	adrenoceptor alpha 2A [Source:HGNC Symbol;Acc:281]
ENSG00000166825	protein_coding	ANPEP	Inf	25.6382893	0	0.01213716	alanyl (membrane) aminopeptidase [Source:HGNC Symbol;Acc:500]
ENSG00000151632	protein_coding	AKR1C2	3.712248	82.6033114	6.30230555	0.01349981	aldo-keto reductase family 1, member C2 (dihydrodiol dehydrogenase 2; bile acid binding protein; 3-alpha hydroxysteroid dehydrogenase, type III)
ENSG00000163283	protein_coding	ALPP	-3.6776184	34.3006692	438.9105651	6.5443E-05	alkaline phosphatase, placental [Source:HGNC Symbol;Acc:439]
ENSG00000163286	protein_coding	ALPPL2	-4.0096199	1.90048507	30.61119839	0.02347392	alkaline phosphatase, placental-like 2 [Source:HGNC Symbol;Acc:441]
ENSG00000184867	protein_coding	ARMCX2	7.8506464	103.908591	0.450164682	3.4714E-06	armadillo repeat containing, X-linked 2 [Source:HGNC Symbol;Acc:16869]
ENSG00000165029	protein_coding	ABCA1	5.364166	55.6246816	1.350494046	0.00407039	ATP-binding cassette, sub-family A (ABC1), member 1 [Source:HGNC Symbol;Acc:29]
ENSG00000023839	protein_coding	ABCC2	-2.9902299	44.6991759	355.1799342	0.03356533	ATP-binding cassette, sub-family C (CFTR/MRP), member 2 [Source:HGNC Symbol;Acc:53]
ENSG00000143515	protein_coding	ATP8B2	3.5263869	98.5537703	8.553128961	0.04661868	ATPase, aminophospholipid transporter, class I, type 8B, member 2 [Source:HGNC Symbol;Acc:13534]
ENSG00000169255	protein_coding	B3GALNT1	Inf	27.2471832	0	0.00649981	beta-1,3-N-acetylgalactosaminyltransferase 1 (globoside blood group) [Source:HGNC Symbol;Acc:918]
ENSG00000153956	protein_coding	CACNA2D1	6.9106732	162.484749	1.350494046	6.5291E-06	calcium channel, voltage-dependent, alpha 2/delta subunit 1 [Source:HGNC Symbol;Acc:1399]
ENSG00000153132	protein_coding	CLGN	4.1231923	39.2234676	2.250823411	0.02790062	calmegin [Source:HGNC Symbol;Acc:2060]
ENSG00000117519	protein_coding	CNN3	4.2858651	87.810284	4.501646822	0.00407039	calponin 3, acidic [Source:HGNC Symbol;Acc:2157]
ENSG00000074410	protein_coding	CA12	4.0821136	99.1181992	5.852140868	0.0074028	carbonic anhydrase XII [Source:HGNC Symbol;Acc:1371]
ENSG00000198108	protein_coding	CHSY3	6.4816969	40.2306083	0.450164682	0.00404145	chondroitin sulfate synthase 3 [Source:HGNC Symbol;Acc:24293]

ENSG00000168675	protein_coding	C18orf1	5.6709245	68.8035821	1.350494046	0.00152876	chromosome 18 open reading frame 1 [Source:HGNC Symbol;Acc:1224]
ENSG00000176907	protein_coding	C8orf4	3.9035398	107.788884	7.202634914	0.00698707	chromosome 8 open reading frame 4 [Source:HGNC Symbol;Acc:1357]
ENSG00000188517	protein_coding	COL25A1	Inf	61.9750734	0	3.7362E-05	collagen, type XXV, alpha 1 [Source:HGNC Symbol;Acc:18603]
ENSG00000121005	protein_coding	CRISPLD1	Inf	16.2605666	0	0.03328614	cysteine-rich secretory protein LCCL domain containing 1 [Source:HGNC Symbol;Acc:18206]
ENSG00000006016	protein_coding	CRLF1	5.9095126	81.1770411	1.350494046	0.00017159	cytokine receptor-like factor 1 [Source:HGNC Symbol;Acc:2364]
ENSG00000147202	protein_coding	DIAPH2	Inf	63.5376506	0	1.6604E-05	diaphanous homolog 2 (Drosophila) [Source:HGNC Symbol;Acc:2877]
ENSG00000113657	protein_coding	DPYSL3	3.9173437	115.626745	7.652799597	0.01553256	dihydropyrimidinase-like 3 [Source:HGNC Symbol;Acc:3015]
ENSG00000133083	protein_coding	DCLK1	Inf	24.7361752	0	0.0072682	doublecortin-like kinase 1 [Source:HGNC Symbol;Acc:2700]
ENSG00000164330	protein_coding	EBF1	Inf	25.2596318	0	0.00622024	early B-cell factor 1 [Source:HGNC Symbol;Acc:3126]
ENSG00000170571	protein_coding	EMB	3.9512468	55.7064169	3.601317457	0.0275176	embigin [Source:HGNC Symbol;Acc:30465]
ENSG00000149218	protein_coding	ENDOD1	6.1563019	32.1072384	0.450164682	0.00698707	endonuclease domain containing 1 [Source:HGNC Symbol;Acc:29129]
ENSG00000145242	protein_coding	EPHA5	6.0410659	29.6424062	0.450164682	0.01973934	EPH receptor A5 [Source:HGNC Symbol;Acc:3389]
ENSG00000156804	protein_coding	FBXO32	4.9939238	100.413089	3.151152775	0.00131259	F-box protein 32 [Source:HGNC Symbol;Acc:16731]
ENSG00000144369	protein_coding	FAM171B	3.7132626	59.0438721	4.501646822	0.04207142	family with sequence similarity 171, member B [Source:HGNC Symbol;Acc:29412]
ENSG00000188738	protein_coding	FSIP2	3.8977668	80.5188202	5.401976186	0.0169419	fibrous sheath interacting protein 2 [Source:HGNC Symbol;Acc:21675]
ENSG00000110195	protein_coding	FOLR1	9.7460157	386.557839	0.450164682	9.9649E-08	folate receptor 1 (adult) [Source:HGNC Symbol;Acc:3791]
ENSG00000248905	protein_coding	FMN1	6.4483604	39.3116549	0.450164682	0.00500445	formin 1 [Source:HGNC Symbol;Acc:3768]
ENSG00000152661	protein_coding	GJA1	6.7101871	47.1345122	0.450164682	0.00132077	gap junction protein, alpha 1, 43kDa [Source:HGNC Symbol;Acc:4274]
ENSG00000139278	protein_coding	GLIPR1	4.8586398	117.546645	4.051482139	0.00040848	GLI pathogenesis-related 1 [Source:HGNC Symbol;Acc:17001]
ENSG00000166923	protein_coding	GREM1	7.0804709	121.852809	0.900329364	8.2707E-06	gremlin 1 [Source:HGNC Symbol;Acc:2001]
ENSG00000117228	protein_coding	GBP1	Inf	61.9473693	0	1.5487E-05	guanylate binding protein 1, interferon-inducible [Source:HGNC Symbol;Acc:4182]
ENSG00000162645	protein_coding	GBP2	5.2783727	34.9421746	0.900329364	0.00925182	guanylate binding protein 2, interferon-inducible [Source:HGNC Symbol;Acc:4183]
ENSG00000143341	protein_coding	HMCN1	4.2763476	43.6164519	2.250823411	0.04323898	hemicentin 1 [Source:HGNC Symbol;Acc:19194]
ENSG00000166503	protein_coding	RP11-382A20.3	4.089296	38.3126494	2.250823411	0.0353038	Hepatoma-derived growth factor-related protein 3 [Source:UniProtKB/Swiss-Prot;Acc:Q9Y3E1]
ENSG00000143452	protein_coding	HORMAD1	Inf	17.1377238	0	0.03533073	HORMA domain containing 1 [Source:HGNC Symbol;Acc:25245]

ENSG00000152580	protein_coding	IGSF10	7.3334553	217.812443	1.350494046	3.6521E-06	immunoglobulin superfamily, member 10 [Source:HGNC Symbol;Acc:26384]
ENSG00000137809	protein_coding	ITGA11	Inf	163.530188	0	3.9365E-07	integrin, alpha 11 [Source:HGNC Symbol;Acc:6136]
ENSG00000115232	protein_coding	ITGA4	Inf	17.5643042	0	0.04433547	integrin, alpha 4 (antigen CD49D, alpha 4 subunit of VLA-4 receptor) [Source:HGNC Symbol;Acc:6140]
ENSG00000169429	protein_coding	IL8	3.679814	40.38349	3.151152775	0.02768494	interleukin 8 [Source:HGNC Symbol;Acc:6025]
ENSG00000112769	protein_coding	LAMA4	7.716541	378.740477	1.800658729	1.257E-06	laminin, alpha 4 [Source:HGNC Symbol;Acc:6484]
ENSG00000049323	protein_coding	LTBP1	3.9995264	64.8024374	4.051482139	0.02933329	latent transforming growth factor beta binding protein 1 [Source:HGNC Symbol;Acc:6714]
ENSG00000147676	lincRNA	MAL2	-3.2266602	18.5144428	173.3134026	0.00483944	mal, T-cell differentiation protein 2 (gene/pseudogene) [Source:HGNC Symbol;Acc:13634]
ENSG00000214548	lincRNA	MEG3	Inf	30.237962	0	0.00511047	maternally expressed 3 (non-protein coding) [Source:HGNC Symbol;Acc:14575]
ENSG00000087245	protein_coding	MMP2	5.5240341	41.4287072	0.900329364	0.00990079	matrix metallopeptidase 2 (gelatinase A, 72kDa gelatinase, 72kDa type IV collagenase) [Source:HGNC Symbol;Acc:7166]
ENSG00000117122	protein_coding	MFAP2	Inf	18.9118879	0	0.03336016	microfibrillar-associated protein 2 [Source:HGNC Symbol;Acc:7033]
ENSG00000131711	protein_coding	MAP1B	7.3638452	296.599011	1.800658729	3.1945E-06	microtubule-associated protein 1B [Source:HGNC Symbol;Acc:6836]
ENSG00000181143	protein_coding	MUC16	5.2413022	221.362445	5.852140868	0.00070119	mucin 16, cell surface associated [Source:HGNC Symbol;Acc:15582]
ENSG00000141052	protein_coding	MYOCD	Inf	73.7488934	0	1.6604E-05	myocardin [Source:HGNC Symbol;Acc:16067]
ENSG00000113389	protein_coding	NPR3	4.8985884	161.129367	5.401976186	0.00070119	natriuretic peptide receptor C/guanylate cyclase C (atrionatriuretic peptide receptor C) [Source:HGNC Symbol;Acc:7945]
ENSG00000252331	NOT FOUND	NOT FOUND	Inf	45.4607286	0	0.0072682	NOT FOUND
ENSG00000074181	protein_coding	NOTCH3	5.9130059	108.498455	1.800658729	0.00023482	notch 3 [Source:HGNC Symbol;Acc:7883]
ENSG00000100968	protein_coding	NFATC4	Inf	45.9632973	0	0.0002467	nuclear factor of activated T-cells, cytoplasmic, calcineurin-dependent 4 [Source:HGNC Symbol;Acc:7778]
ENSG00000139946	protein_coding	PELI2	5.7386652	48.0741681	0.900329364	0.00394207	pellino E3 ubiquitin protein ligase family member 2 [Source:HGNC Symbol;Acc:8828]
ENSG00000186642	protein_coding	PDE2A	10.514973	658.708924	0.450164682	3.0761E-07	phosphodiesterase 2A, cGMP-stimulated [Source:HGNC Symbol;Acc:8777]
ENSG00000154864	protein_coding	PIEZO2	Inf	120.920845	0	5.9836E-07	piezo-type mechanosensitive ion channel component 2 [Source:HGNC Symbol;Acc:26270]
ENSG00000156011	protein_coding	PSD3	4.1468066	119.612303	6.752470232	0.00698707	pleckstrin and Sec7 domain containing 3 [Source:HGNC Symbol;Acc:19093]
ENSG00000120594	protein_coding	PLXDC2	4.263195	51.8647442	2.700988093	0.01718206	plexin domain containing 2 [Source:HGNC Symbol;Acc:21013]
ENSG00000126838	protein_coding	PZP	-3.6462224	6.83128956	85.53128961	0.00257955	pregnancy-zone protein [Source:HGNC Symbol;Acc:9750]
ENSG00000106772	protein_coding	PRUNE2	9.990984	458.096822	0.450164682	2.636E-07	prune homolog 2 (Drosophila) [Source:HGNC Symbol;Acc:25209]
ENSG00000250305	protein_coding	KIAA1456	-5.1624714	0.35194168	12.6046111	0.01875889	Putative methyltransferase KIAA1456 [Source:UniProtKB/Swiss-Prot;Acc:Q9P272]

ENSG00000004799	protein_coding	PDK4	4.3533989	478.496113	23.40856347	0.00649981	pyruvate dehydrogenase kinase, isozyme 4 [Source:HGNC Symbol;Acc:8812]
ENSG00000165105	protein_coding	RASEF	Inf	19.9417574	0	0.02727153	RAS and EF-hand domain containing [Source:HGNC Symbol;Acc:26464]
ENSG00000071242	protein_coding	RPS6KA2	Inf	54.1433961	0	0.00013733	ribosomal protein S6 kinase, 90kDa, polypeptide 2 [Source:HGNC Symbol;Acc:10431]
ENSG00000178222	protein_coding	RNF212	Inf	36.3920398	0	0.00130864	ring finger protein 212 [Source:HGNC Symbol;Acc:27729]
ENSG00000006747	protein_coding	SCIN	-3.5851859	4.12587062	49.51811504	0.02962233	scinderin [Source:HGNC Symbol;Acc:21695]
ENSG00000178172	protein_coding	SPINK6	3.7481695	429.478283	31.96169243	0.0134844	serine peptidase inhibitor, Kazal type 6 [Source:HGNC Symbol;Acc:29486]
ENSG00000130413	protein_coding	STK33	Inf	18.8605924	0	0.04207142	serine/threonine kinase 33 [Source:HGNC Symbol;Acc:14568]
ENSG00000149212	protein_coding	SESN3	Inf	152.290166	0	2.636E-07	sestrin 3 [Source:HGNC Symbol;Acc:23060]
ENSG00000179542	protein_coding	SLITRK4	6.3607582	36.9956299	0.450164682	0.00347259	SLIT and NTRK-like family, member 4 [Source:HGNC Symbol;Acc:23502]
ENSG00000070915	protein_coding	SLC12A3	-3.4106512	12.1067938	128.7470991	0.00939689	solute carrier family 12 (sodium/chloride transporters), member 3 [Source:HGNC Symbol;Acc:10912]
ENSG00000146411	protein_coding	SLC2A12	4.5654977	42.6366574	1.800658729	0.022715	solute carrier family 2 (facilitated glucose transporter), member 12 [Source:HGNC Symbol;Acc:18067]
ENSG00000137642	protein_coding	SORL1	4.8898683	93.425727	3.151152775	0.00257955	sortilin-related receptor, L(DLR class) A repeats containing [Source:HGNC Symbol;Acc:11185]
ENSG00000134668	protein_coding	SPOCD1	5.376056	37.3900045	0.900329364	0.01553256	SPOC domain containing 1 [Source:HGNC Symbol;Acc:26338]
ENSG00000156414	protein_coding	TDRD9	6.4929255	40.5449479	0.450164682	0.00257955	tudor domain containing 9 [Source:HGNC Symbol;Acc:20122]
ENSG00000164761	protein_coding	TNFRSF11B	Inf	16.50604	0	0.04207142	tumor necrosis factor receptor superfamily, member 11b [Source:HGNC Symbol;Acc:11909]
ENSG00000104723	protein_coding	TUSC3	6.2499493	102.781488	1.350494046	1.6604E-05	tumor suppressor candidate 3 [Source:HGNC Symbol;Acc:30242]
ENSG00000182986	protein_coding	ZNF320	6.0345804	29.5094506	0.450164682	0.01389319	zinc finger protein 320 [Source:HGNC Symbol;Acc:13842]
ENSG00000181495	protein_coding	AC026703.1	5.8143527	126.658984	2.250823411	4.4366E-05	
