# Supplementary material for: Simultaneous Transcriptional Profiling of Bacteria and Their Host Cells
Source: PLoS One. 2013 Dec 4;8(12):e80597. doi: 10.1371/journal.pone.0080597 (PMC3851178; doi:10.1371/journal.pone.0080597)
Supplement: Table S6 — Primer and probe sets used in this study. (a) Custom Taqman primers and probes used for Chlamydia qRT-PCR assays. (b) Predesigned Taqman primer and probe sets used for human qRT-PCR assays. (PDF) [file pone.0080597.s010.pdf]

Table S6. (a) Custom Taqman primers and probes used for *Chlamydia* qRT-PCR assays. (b) Predesigned Taqman primer and probe sets used for human qRT-PCR assays.

(a)

Assay Target	Forward Primer Sequence (5'-3')	Reverse Primer Sequence (5'-3')	Probe Sequence (5'-3')
16S	GTAGCGGTGAAATGCGTAGATATGT	GCGCCTTAGCGTCAGGTATAAATTA	FAM - CCACTGGTGTTCTTCC - NFQ
CT001	CCATGTGGACACAAGCAGCTTAT	ACTTCAAGAAGTTTAGGCAGACCAT	FAM - CCCCAGCGATTCCCGT - NFQ
CT018	CCTCTGTCAAAATGACCCGAACATT	GCACTTGTGGCCATTCTTTTCTAAT	FAM - CCTTGCGATAAGTTCC - NFQ
CT038	CCCATTTAAAAGAAGCTCTCGAGAAAC	CTTTCGGATCATCCCAAGAAGCTA	FAM - ATGGGTGCTCAACTCT - NFQ
CT080	AGTTCGATGTGCTAGTACATTCGTT	CACAAAGCCATTCTGGTACGTAAAA	FAM - CACGTTCCTCAAAATT - NFQ
CT081	GGGCTACAGAGATGCACTTTATTG	CTCTGGGCCTTGCGAAAAAA	FAM - CCCGCTTTTCTTTTCC - NFQ
CT088	AGGATGGTAATTCAGGATTTTTAGTTGCT	AGCAGCTTTGAAAATTTTTTGTCTAAAGGT	FAM - ATGCTTGGAAAACTT - NFQ
CT214	GCAGCTCCCTTTCTATCTTTGATTGA	GAAAGAAGGGAAGCCTCTAAAGCTA	FAM - TACTCACCAAAATCCC - NFQ
CT216	CCAATTTGGCTTCGGATATGGAA	GGCAAGAGTGGATACAGCTCCTA	FAM - CCCAAAGCCGTTTTCA - NFQ
CT229	AGGACTTTTAGTTGCTACTGCGG	GGAACCAACCATAACTAAGATCATACCA	FAM - CTACTGCTGCAATCAC - NFQ
CT391	GTAACCAACAACGCGAATTCGT	CGCGCGGAGTTTATTTGTAATTTCT	FAM - AACCCCACGAACCTT - NFQ
CT398.1	ATCTCCTCCGTATTCAGACAATCCT	GGTGTGTCTGTGTAGTAAATGAGCTT	FAM - CCATCTGTGAATATCC - NFQ
CT416	TGCAGCATTTCCTATCACTGTAAAGG	AGCAGCTTCATGATCTTCTTGTGTA	FAM - CAGTGTCGCCAACCTT - NFQ
CT444	TGCAACTCTTGTAACGGGTATGTC	GTTTAGCATCTTGGTGCGTATCC	FAM - CCAGCTTGCAAACCT - NFQ
CT446	GGAGAGCTTCTGTTTGATAACGAGA	GCGTGTAGCATAGTAAATCTTCTGTTCA	FAM - ATGGACGCCACTTGTC - NFQ
CT480.1	CTTTTACTCGCAATGTTAACTTCTGCT	TTTTTCTTCTTCAGTCTCATCAGCGTAA	FAM - CCGCTGCTGGTTCCGT - NFQ
CT500	TCTCTGGTCCTGTTGTAGTAATGGT	GCTCCCATCAACTCTCTGTTACG	FAM - TCGACAGCGTTTTCC - NFQ
CT565	TGGTGCTTTGATTGTTTTTGGGATT	TCCCGGAGGCAACAAATAACC	FAM - CTCCGACCCAAGACAC - NFQ
CT576	CCAAGGGAAATATCAGGAAGCTATCG	TGATAGCAAGAGCTAAGACCTAAGATGT	FAM - CAGCCTCCAAACCTCA - NFQ
CT577	ACTCAGCGGAATGTTAGACTCTCT	GGCTTCCTCTTTAGGATTGTCAAAC	FAM - TCCTGTCGAAACCTTG - NFQ
CT579	GGTCTCTGTAGGAGGAGGGATTTTA	GCGCTTGCAGTTTCATTAGTGA	FAM - CCTCCCAAACTCTTCG - NFQ
CT659	GATAAGCCAGAGGAAGTGCATCT	CTCCCTTAGCAACAGTCAATTCGTA	FAM - TCCCTGCACCTCTTTC - NFQ
CT660	TCCTTCCACAACTACAGAGACTCT	GGTCCGTAGAGAAATCTTGAATCGA	FAM - TTTTGCTGCGTTTTCG - NFQ
CT665	GCCCACGAAGTAAATGCTAATGTAC	ACTCTTTAGAAGCCCCTTCTCGAA	FAM - ACGCTGACATCTTCTC - NFQ
CT734	CAGATCCAATGACAGATTCCGAAAGA	TGAGCCATAGCCAAAGCAATAGAA	FAM - CTGCTGCTGATAAGTC - NFQ
CT798	TCTCCGCGGGATTTTAAATGGAA	TCGCTAAATTGGGATCCGTTTCA	FAM - TCGACACGACAATTTG - NFQ
CT814	CCCTAAAAGTGGGATGCAAATCAGA	GGCTCGGCCTTTCTTCGT	FAM - CTCCGCGTCAAGACAT - NFQ
CT814.1	TGAAACTTGTCAGTCGAATTTCTTCTCT	GCATACTTCTTGCGTAACCAATTTAGAAT	FAM - CTCGCTGGCTTGCACC - NFQ
CT834	CGCGTTTCAAATTGACTGGTTCTG	GCGTTTCTGTTGCGAAGATCTTTT	FAM - TTCCCCGGACGAGTTC - NFQ
CT864	GCGTTACTGGTAAAGGAAGAAAGAC	GCGTCAATAGCTTGTTTGGCTTT	FAM - CTCGTGCCCATCAGCA - NFQ
CT875	ACCCCAACGAGTGGAGAGA	CCTCTTTGGAAACGACTGAACAC	FAM - TCCACGCGAAGCTCT - NFQ

(b)

Gene Symbol	ENSEMBL ID	Assay ID
BIRC3	ENSG00000023445	Hs00985031
CCL20	ENSG00000115009	Hs01011368
CXCL3	ENSG00000163734	Hs00171061
ELF3	ENSG00000163435	Hs00963881
ERRFI1	ENSG00000116285	Hs00219060
ETS1	ENSG00000134954	Hs00428293
GREM1	ENSG00000166923	Hs01879841
IL1A	ENSG00000115008	Hs00174092

IL8	ENSG00000169429	Hs00174103
LAMB3	ENSG00000196878	Hs00165078
SERPINE1	ENSG00000106366	Hs01126606
TNC	ENSG00000041982	Hs01115665
TNNC1	ENSG00000114854	Hs00896999
TRPV3	ENSG00000167723	Hs00376854
