# Supplementary material for: Simultaneous Transcriptional Profiling of Bacteria and Their Host Cells
Source: PLoS One. 2013 Dec 4;8(12):e80597. doi: 10.1371/journal.pone.0080597 (PMC3851178; doi:10.1371/journal.pone.0080597)
Supplement: Text S1 — (PDF) [file pone.0080597.s011.pdf]

SUPPORTING TEXT

Attachment and Entry. The precise mechanism of chlamydial attachment and entry into host cells is uncharacterized, but is likely to rely upon host cell outer membrane and ECM molecules. As entry into the host cell is an absolute requirement for completion of the developmental cycle, multiple redundant chlamydial adhesins are likely, putatively including OmcB and members of the diverse Pmp family of membrane proteins [1]. Entry into the host cell via clathrin-based endocytosis has been demonstrated, although the current consensus is that multiple mechanisms are likely [2]. Accordingly, several possible host receptors have been proposed [1], particularly ECM and membrane-bound proteoglycans. Heperan sulfate proteoglycans are involved in chlamydial attachment [3], and host sulfation pathways are needed for successful chlamydial attachment [4]. While the 1 hpi time may be outside of the window of attachment and initial invasion, several sulfation enzymes that modify heperan sulfate (heperan sulfates 3-O-sulfotransferase 3B1, 6-O-sulfotransferase 2 and 6-O-sulfotransferase 3, and iduronate-2-sulfatase) or chondroitin sulfate (chondroitin sulfate synthase 3; *CHSY3*) are highly expressed at 1 hpi.

Transcriptional evidence for attempted dampening of epithelial innate immune responses. The pathology associated with chlamydial disease is an adverse outcome of host inflammation (reviewed by [5]). Repeated stimulation, either from long-term infection or successive re-infections, leads to tissue damage and scarring. There is evidence that *Chlamydia* exploits immune and inflammatory pathways for survival [6-8]. Under the cellular paradigm of chlamydial pathogenesis [9], infected epithelial cells are the first responders to chlamydial infection, initiating and promoting the immune response [6-8,10]. Thus, subverting or dampening this response may contribute to the adverse consequences of infection. We identify several *in vitro* host cell transcriptional responses to infection, including putative modulation of innate responses through cytokines, chemokines and the inflammasome, which can be interpreted in the context of immune dampening.

Mucins are heavily glycosylated proteins produced by epithelial cells that are secreted or membrane-bound [11]. *MUC-5B* and *MUC-6* are down regulated at 1 hpi, and *MUC-16* (CA-125) is up-regulated at both 1 and 24 hpi. In the guinea pig model of chlamydial infection, heavy mucus production is observed early in infection, demonstrating a possible *in vivo* correlate of increased mucin gene expression. In humans, MUC-16 is produced by corneal and conjunctival cells, and by epithelial cells within the respiratory and female reproductive tracts [12]. If the observed differential expression of mucin genes translates to mucin protein production, chlamydial pathogenesis may be impacted in several ways. Up-regulation of *MUC-16* has been found to aid immune evasion by ovarian tumors by suppressing the natural killer (NK) cell response [13], although there is no evidence of such immune suppression associated with chlamydial infection. Increased mucin production may obstruct infection by forming a

physical barrier against pathogen invasion [14]. Mucins and TLRs may cooperate to strengthen the mucosal immune response to pathogens, as several mucins are TLR ligands and there is evidence of crosstalk between mucin and TLR signaling pathways [15]. Alternatively, increased mucin expression may hinder the immune response by sequestering other TLR ligands or by physically "masking" the TLR [15], preventing or delaying activation.

Members of these putative feedback loops may be useful diagnostic markers for chlamydial tissue damage. For example, increased tenascin-C mRNA and protein levels often precede obvious tissue damage or inflammation, and are used as predictive markers for fibrosis and inflammation in myocarditis [16,17]. Furthermore, serum tenascin-C levels in inflammatory bowel disease are found to correlate with disease severity [16,17]. Thus, there is potential for the paracrine moieties identified above to be markers of chlamydial disease in both local tissues and serum. In addition to this diagnostic potential, members of these putative positive feedback loops could also be targets for therapeutic intervention to prevent scarring.

Tissue damage releases factors that act as damage-associated molecular patterns (DAMPs), and stimulate inflammatory processes [18,19]. Decorin is a proteoglycan in the extracellular matrix (ECM) in the small leucine-rich proteoglycan (SLRP) family; it participates in collagen fibril formation but also acts as a powerful DAMP on release from the ECM [20]. Soluble decorin induces proinflammatory signaling and is recognized as an endogenous ligand of TLR2 and TLR4 [20,21]. Decorin-induced signaling appears to orchestrate multiple pathways and may be a crucial regulator of the inflammatory response to cellular damage [20]. We find that decorin (*DCN*) is strongly down-regulated at 1 hpi, providing support for the attenuation of some inflammatory signals on chlamydial infection.

Production of antimicrobial peptides and proteins (AMPs) is an evolutionarily conserved host response to infection [22]. The AMP azurocidin 1 (AZU1), also known as heparin-binding protein (HBP) [23-25], is a chemoattractant and activator of monocytes and macrophages that enhances cytokine release and phagocytosis [22]. Azurocidin may also act as an alarmin [26]. At 1hpi, the gene encoding azurocidin (*AZU1*) is strongly down regulated compared to mock-infected cells; reduced AZU1 production may attenuate the adaptive immune response.

At 1 hpi a secreted semaphorin is upregulated (*SEMA3A*). Semaphorins bind to membrane-bound plexin receptors and were originally identified as factors of axon repulsion or attraction in neuronal development [27]. Members of the semaphorin family are involved in immune responses, particularly in dendritic cell

(DC) trafficking and migration [28,29]. SEMA3A is a negative regulator of DC migration, as it inhibits migration along chemokine gradients and also suppresses T-cell proliferation [28]. Increased expression of *SEMA3A* in the early chlamydial-infected epithelial cell may have a spatiotemporal dampening effect on the attraction of DC subsets or other activated immune cells. *SEMA3A* expression has also been reported to enhance innate immune responses by exacerbating TLR-induced cytokine expression [28,30]. In turn, TLR activation induces *SEMA3A* [30], establishing a possible positive feedback loop that could contribute to the deleterious effects of chlamydial infection through differential inhibition of the adaptive response, and by amplification of innate responses that further promote inflammation.

During infection, the Golgi apparatus is fragmented into ministacks that surround the inclusion [31]. Chlamydial appropriation of the Golgi apparatus may enable transfer of sphingomyelin to the inclusion [32]. Synthesis of sphingomyelin is a multistep process, beginning with ceramide synthesis on the ER, followed by CERT-mediated transfer to the Golgi and subsequent conversion to sphingomyelin by sphingomyelin synthases [33,34]. We find acyl-CoA-dependent ceramide synthase 6 (*CERS6*), which acts upon sphingosine to form ceramide, exhibits increased expression at 1hpi. We also find increased expression of sphingosine kinase 1 (*SphK1*), which also acts upon sphingosine to form the metabolite sphingosine 1-phosphate (S1P). S1P is a major signaling molecule involved in many biological processes, particularly immunity [35-37]. S1P acts both as a secondary messenger intracellularly, including epigenetic modifications of histone deacetylase (HDAC), and as an extracellular ligand of a closely related family of GPCRs ($S1P_1$ – $S1P_5$) that are expressed on immune cells [35]. Each of these receptors has a different but partially overlapping signaling pathway – thus the pattern of S1P receptors can confer either pro- or anti-inflammatory effects, depending on the cellular context [35,38]. A major outcome of S1P binding is recruitment of immune cells, with immune cell subsets responding differently to varying concentrations of S1P – low concentrations promote chemotaxis, whereas high concentrations of S1P are differentially inhibitory to immune cell subsets [39,40]. The observed up-regulation of *SphK1* in early chlamydial infection and a putative concomitant increase in S1P may influence the recruitment and migration of different immune cell subsets to the site of infection.

To summarize – consistent with the known biology of chlamydial infection, we observe overt induction of an early innate response, exemplified by transcriptional evidence for chemokine expression and TLR activation through known PRR sensing pathways. We also see transcriptional evidence of a putative dampening effect on several innate and signaling mechanisms that could subdue aspects of the immune response to chlamydial infection. However, we note that *in vivo* responses in animal models or human infections may not correlate with the optimized *in vitro* infections of immortalized epithelial cells used in

this study. We also note that these putative attenuating effects may not be a specific chlamydial strategy but a more general cellular response to intracellular infection. RNA-Seq analysis of additional infection times and differentiation of the general phagocytic response will help to better define specific *Chlamydia*-induced changes to the dynamic innate response.

Chlamydiae are recognized by a variety of membrane-bound and cytoplasmic sensors contained within the epithelial cell (reviewed by [7]). These sensors, termed pathogen recognition receptors (PRRs) detect a diversity of microbial conserved structures (pathogen-associated molecular patterns, PAMPs) [41]. Recognition of chlamydial PAMPs by host PRRs triggers upregulation of proinflammatory cytokines and chemokines that recruit immune cells to the infection site [7]. Recruitment and subsequent infiltration of immune cells to the site of infection is a feature of the *in vivo* immune response to *Chlamydia*, possibly playing a role in both the destruction and dissemination of infected cells [8]. The Toll-like receptors (TLRs) are key PRRs, with TLR2 and TLR4 activation associated with host cell detection of chlamydiae [7]. We detect up-regulation of TLR4 at 1 hpi, but we do not observe TLR2 induction in this dataset. However, we do find up-regulation of IRAK3 (interleukin-1 receptor-associated kinase; IRAK-M), which may function as a TLR2-specific negative regulator of TLR-signaling through the alternative NFκB pathway [42], suggesting TLR2 involvement. Conversely, TRIB3 (tribbles 3; SINK) is down-regulated. TRIB3 has been shown to inhibit NFκB via p65 phosphorylation [43,44]. Members of the tribbles family interact with several key intracellular signaling pathways, including the MAPK and PI3K signaling pathways [45] and appear to be critical checkpoints in coordinating cellular responses [45]. Repression of TRIB3 increases the TLR2/NFκB-mediated inflammatory response to *Helicobacter pylori* [46], suggesting TRIB3 may be a target for pathogens to modulate inflammation. In addition, PELI2, a Pellino E3 ubiquitin protein ligase family member, is strongly up-regulated at 1hpi. Members of the Pellino family of proteins function as upstream mediators of TLR signaling via polyubiquitylation of IRAKs [47] and thus may be part of the innate response to chlamydial infection. Further RNA-Seq analyses at a finer granularity over the course of infection will reveal more details of this rapid innate response.

Innate immune processes are rapidly induced in chlamydial infection of epithelial cells via an NFκB-dependent host response arising from TLR2 and TLR4 activation [5,7,48]. We find numerous components of the NFκB and MAPK complexes and TNF-α pathways are differentially expressed at 1 hpi, including factors that precede an interferon type I response, consistent with known biology [7]. Three genes encoding members of the small interferon-induced transmembrane protein family (IFITM1, IFITM2 and IFITM3) are down-regulated, relative to mock-infected cells. This is unusual as IFITM expression is typically activated in response to bacterial or viral infection [49].

We also observe differential expression of constituents of a chlamydial-induced inflammasome complex. The cytoplasmic NOD (nucleotide oligomerization domain)-like receptors (NLRP) sense intracellular pathogens to induce an inflammatory response [7,50]. NLRP1 is up regulated at 1 hpi. Inflammasome components caspase-1, and caspase-4 (which activates caspase-1), and a caspase-1 inhibitor (caspase recruitment domain family protein; CARD-16; PSEUDO-ICE), are down regulated relative to mock-infected cells at 1hpi. In addition to the TLR, CLR and NOD PRR sensors, host epithelial cells also possess cytosolic Rig-like receptors (RLR) that recognize nucleic acid PAMPs (typically viral) and subsequently induce innate immune processes [7,51]. Chlamydial infection induces a type 1 IFN response, presumably through recognition of chlamydial nucleic acid PAMPs [7]. As this response is not beneficial to the host, Nagarajan (2012) [35] hypothesized that the intracellular *Chlamydia* may be modulating these pathways for its own benefit. Consistent with this hypothesis, we observe down-regulation of the RLRs *Rig-1* (*RARRES3*) and *MDA5* (*IFIH1*) at 1hpi. RNF125, an E3 ubiquitin ligase that negatively regulates Rig-1 [52] is also down-regulated at that time.

At 3 hpi, Rank et al detected *in vivo* expression of numerous chemokines (*CCL3, CCL20, CCL24, CCL25, CXCL15*), chemokine receptors (*CCR2, CCR6*), and cytokines (*IL1F8, IL-13* and *TNF-α*) in *C. muridarum*-infected mice [8]. Using *C. trachomatis* in human epithelial cells *in vitro*, we find *IFN-ε*, *IL11*, and *IL32* are up regulated at 1hpi, while *IL8* up-regulation is observed at both 1 and 24 hpi, consistent with previous findings [10]. The *ILR1* and *IL7R* receptors are also up-regulated at 1 hpi, while the *IL20RB* receptor is down-regulated. We observe up-regulation of the chemokine *CCL2*, which recruits monocytes, memory T-cells and dendritic cells, and *CXCL1*, which attracts polymorphonuclear neutrophils (PMNs) at 1hpi. PMN activation and recruitment is associated with ascending infection of the genital tract and increased disease severity in mice (reviewed by [5]). The chemokine receptor *CXCR7* is also up-regulated at 1hpi. Conversely, the chemokine *CCL5* (*RANTES*) is down-regulated at 1 hpi. Differential expression of these various chemokine genes at 1 hpi supports rapid establishment of chemokine gradients *in vitro*.

Differential expression of growth factors. We observe differential expression of many host growth factor genes at 1 hpi. Amphiregulin (*AREG*), epiregulin (*EREG*), epithelial mitogen homolog (*EPGN*), growth differentiation factor 15 (*GDF15*) and inhibin beta E (*INHBE*) are all down-regulated. Conversely, fibroblast growth factor 12 (*FGF12*), growth arrest-specific growth factor 6 (GAS6), heparin-binding EGF-like growth factor (*HB-EGF*), platelet derived growth factor C (*PDGFC*), an isoform of the profibrogenic transforming growth factor family (*TGFB2*, discussed further below) and vascular endothelial growth factor C (*VEGFC*) are all up-regulated. Thus, it is readily apparent that

chlamydial infection induces the expression of a complex array of growth factors, which are likely to have wide-ranging effects on infected and adjacent cells – for brevity we focus on two, amphiregulin and GAS6.

One outcome of growth factor induction is the modulation of apoptosis – chlamydiae inhibit apoptosis to avoid destruction of the host cell prior to completion of the developmental cycle, and to minimize apoptotic immune activation from release of host cell components [53]. Amphiregulin (as well as epiregulin & HB-EGF) is a ligand of the ErbB family of tyrosine kinase receptors [54,55], which regulate diverse cellular signaling pathways that control complex cellular outcomes such as apoptosis or adhesion; notably, we also observe down-regulation of the ErbB3 receptor. Amphiregulin, which is down-regulated here, is an abundant membrane-anchored glycoprotein that is activated by ADAM proteinase cleavage in response to external stimulus, and subsequently binds to ErbB receptors [56]. Amphiregulin also has anti-microbial activity *in vitro* [57]. Amphiregulin dysregulation has been observed in several other bacterial infections, including *Shigella flexneri* [58], EHEC [59], *Helicobacter pylori* [60] and *Neisseria gonorrhoeae* [61], and has been associated with interference with host apoptosis pathways in these pathogens.

Putative interference with apoptotic pathways is also apparent for the up-regulated GAS6 growth factor. GAS6 is a ligand for Axl, a member of the TAM (Tyro3, Axl and Mertk) tyrosine kinase receptor family. GAS6 binding to Axl activates phosphoinositide 3-kinase (PI3K) and its downstream target, the serine/threonine protein kinase Akt [62-64]. The Gas6/Axl/PI3K/Akt pathway protects cells from apoptosis via multiple mechanisms [63,64]. For example, Akt activates ribosomal protein S6K (S6 kinase) of the mTOR (mammalian target of rapamycin) pathway and phosphorylates BAD (Bcl2-associated agonist of cell death), a pro-apoptotic protein [64]. In addition, *C. trachomatis* induces the PI3K/Akt pathway as part of an anti-apoptotic strategy where phosphorylated BAD is also sequestered to the chlamydial inclusion, possibly as part of a strategy to prevent mitochondrial-induced apoptosis [65]. However, the inducer of the PI3K/Akt pathway in chlamydial infection has not been identified. Our RNA-Seq data suggests that GAS6 may be one inducer. Strongly increased expression of S6 kinase (*RPS6KA2*) at 1hpi provides additional support for induction of Gas6/Axl/PI3K/Akt. The GAS6/TAM system is also a negative regulator of TLR signaling [66]; thus, increased expression of *GAS6* may also help attenuate induction of TLR-induced inflammation.

Disruption to cell-cell adhesion and signaling. Loss of epithelial adhesion is part of the reversible epithelial-to-mesenchymal transition developmental transition [67], implicated in the fibrotic sequelae of

chronic chlamydial infection [68]. In the preceding section, we noted significant differential expression of many ECM constituents that may contribute to the long-term adverse fibrotic sequelae of chlamydial disease. Combined with PMN-mediated displacement of infected cells [8], these ECM alterations may also contribute to sloughing and subsequent dissemination of infected cells, and cellular signaling. Cellular rounding and retraction from intercellular junctions have been observed both *in vitro* [69] and *in vivo* [70-72], also possibly contributing to sloughing and dissemination [73]. Consistent with this, we find disruption of inter-cell adhesion and communication mediated by the various intercellular junctions.

Intercellular junctions preserve epithelial cell polarity and integrity, and mediate adhesion and signaling between cells, often by interaction with the actin cytoskeleton. Components of several different types of junctions are differentially expressed. For example, claudin-7 (*CLDN7*), a key component of the epithelial tight junction [74] is down-regulated, whereas *GJA1* (connexin-43), a major element of the gap junction [75], is up-regulated. Several constituents of the epithelial adherans junction [76] exhibit differential expression at 1 hpi. *AbLIM3*, a member of the actin binding LIM family of proteins is up-regulated at 1 hpi. The cadherins *CDH6* and *CDH13* are up-regulated at 1 hpi, but *CDH3* and *CDHR5* are down-regulated at the same time. Two members of the transmembrane protocadherin family (*PCDH9* and *PCDH10*), which may participate in adhesion or signaling [77], are also down-regulated. Tensin (*TNS1*), which is typically localized to focal adhesions and interacts with the cytoplasmic tails of integrin β subunits and actin [78], exhibits decreased expression at 1hpi.

Another major component of the adherans junction complex, β-catenin (Armadillo), links cadherins with the cytoskeleton to regulate cellular adhesion processes [79]. β-catenin functionally integrates external interactions with the nucleus by transducing canonical Wnt signals to the nucleus, with subsequent transcriptional activation of numerous Wnt-responsive genes [79]. The Wnt-β-catenin signaling cascade and its myriad regulatory feedback loops are linked to many biological processes, including cellular development and differentiation, inflammatory responses and apoptosis [80]. In a recent study using an *ex vivo* fallopian tube model of *C. trachomatis* infection, β-catenin was sequestered to the chlamydial inclusion, with concomitant disruption to Wnt signaling and loss of epithelial homeostasis [68]. Our RNA-Seq data shows that members of this key signaling pathway are differentially expressed in *Chlamydia*-infected cells early in infection. FZD10, a member of the *frizzled* G-protein coupled receptor family that forms the Wnt signaling receptor complex [81], is down-regulated at 1 hpi. The cytoplasmic domain of the *frizzled* receptor complex activates *disheveled*, which modulates β-catenin levels [82]. *Dapper1* is a negative regulator of *disheveled* [83,84]; we find increased *Dapper1* expression in *Chlamydia*-infected cells at 1 hpi. DKK3 (*dickkopf 3*), a secreted glycoprotein that antagonizes Wnt

signaling [85], also exhibits increased expression at 1hpi. Runt-related transcription factor-3 (*RUNX3*), up-regulated at 1 hpi, forms a ternary complex with β-catenin/transcription factor-4 (TCF4; down-regulated) to attenuate Wnt signaling [86]. Thus, several factors of the Wnt-β-catenin signaling cascade are dysregulated in early chlamydial infection. Modulation of Wnt pathways is consistent with our recurring theme of chlamydial attenuation of the early immune response to infection. Supporting this, altered expression of another Wnt antagonist, DKK1, has been linked to attenuation or evasion of the immune response by *Rickettsia conorii* [87].

Integrins are heterodimeric membrane proteins that connect the actin cytoskeleton to the ECM, and are integral to signal transduction [88]. Integrins are widely expressed, and every nucleated cell in the body possesses a specific integrin "signature". 18 possible α-subunits and eight β-subunits assemble into 24 different integrins, which bind collagens, laminins, or RGD-containing proteins [88,89]. Some integrins will also bind soluble ligands or cellular receptors [88,89]. We find that two genes encoding the integrin α4 (*ITGA4*, *CD49d*) and α11 subunits (*ITGA11*) are up-regulated at both 1 and 24 hpi. Integrin α11 typically forms a heterodimer with β1 (α11β1). As part of the collagen-binding subfamily of integrins, it may participate in the formation of focal adhesions typically formed around a transmembrane core of an integrin heterodimer [88]. Integrin α4 is found as a heterodimer with β1 (α4β1, VLA4) or β7 (α4β7, LPAM-1); these heterodimers are typically found on leukocytes and are associated with leukocyte activation, homing and trafficking, as well as interactions with the ECM via fibronectin [88]. α4β7+ memory T-cells have been demonstrated to accumulate in the endocervix of women infected with *C. trachomatis* [90], but it is not clear why integrin α4 would be expressed on an epithelial cell. However, irrespective of function, this unusual pattern of increased α4 and α11 integrin expression may be useful for immunophenotyping of *Chlamydia*-infected epithelial cells.

Our data also shows that the conserved Notch signaling cascade is altered by chlamydial infection. We find that the Notch3 receptor is strongly expressed at both 1 and 24 hpi relative to mock-infected cells. At 1 hpi, we also observe increased expression of delta and Notch-like epidermal growth factor-related receptor (DNER), a known Notch ligand [91]. The Notch signaling cascade is initiated by ligand binding to the Notch receptor, which triggers ADAM/γ-secretase-mediated cleavage [92]; as noted above, we observe differential expression of several ADAM proteases that could participate in this cleavage. Notch signaling determines diverse aspects of cell fate, including growth, differentiation and survival [93-95], however recent evidence suggests that there is also crosstalk between Notch and inflammatory pathways, including the NF-κB, TLR and Wnt pathways [93]. Notch expression has been shown to negatively regulate the TLR4-mediated inflammatory response [96]. Notch signaling has not previously been linked

with chlamydial infection; the Notch3/DNER induction observed here may represent another facet of putative chlamydial interference in the immune response through attenuating the pro-inflammatory outcomes of TLR and Wnt pathway activation.

Remodeling of the cytoskeleton, membrane and lipid trafficking mechanisms. Upon attachment and invasion, chlamydiae co-opt the host cytoskeleton through secreted effectors that interact with host actin, or manipulate cytoskeletal regulatory factors [97,98]. Some of these host factors have been previously identified in chlamydial infection, including Rho GTPases, and phosphoinositides (PIs) [97,98]. Many of the differentially expressed host genes identified by our RNA-Seq data are directly relevant to chlamydial-induced cytoskeletal rearrangements, reflecting the central role of the cytoskeleton to infection and pathogenesis.

Following invasion of the host cell, the chlamydiae-bearing endosome is rapidly remodeled into the chlamydial inclusion (reviewed by [32]). This is likely to occur by modification of the cytoskeleton and incorporation of chlamydial proteins into the inclusion membrane. A primary outcome of this remodeling is the dissociation of the modified inclusion from endosomal trafficking pathways, thus avoiding phagolysomal fusion. Once established, the mature chlamydial inclusion is stabilized by the formation of an actin and intermediate filament scaffold that encapsulates it [99]. At 1hpi, expression of α-actin (*ACTC1*) itself is strongly up-regulated, as is α-internexin (*INA*), a class IV intermediate filament. Several components of myosin are also differentially expressed; myosin VB (*MY5B*) is down-regulated, whereas light chain myosin 9 (*MYL9*) and tropomyosin-α (*TPM1*) increase. We observe decreased expression of three Rho GTPases (*RhoU* and *RhoV*, and the Rac subfamily member *RhoG*). The Rho GTPase-activating proteins *ARHGAP4* and *ARHGAP31* exhibit decreased and increased expression respectively. The Golgi-localized guanine nucleotide exchange factor *FGD1* is also down-regulated at 1hpi. Both FGD1 and ARHGAP31 activate the Rho GTPase Cdc42 [100,101]. Cdc42 is part of membrane trafficking regulation from the Golgi complex and actin dynamics of the early endosome, and is a factor in chlamydial entry [102]. DIAPH2 (diaphanous homolog 2), which interacts with RhoD, and is thus involved with endosome dynamics and actin cytoskeletal reorganization [103], is up-regulated. The actin scaffolding protein, AKAP12 (A-Kinase-Anchoring Protein 12) is up-regulated at 1 hpi. The GTPase dynamin (*DNM1*), involved in scissioning of newly formed endocytic vesicles from the cell surface (particularly clathrin-mediated endocytosis) or the Golgi apparatus [104], exhibits increased expression at 1 hpi.

Filamin C (*FLNC*), which crosslinks actin filaments into networks, acts as a scaffold for the binding of Rho GTPases and their regulators, and also aids in membrane anchoring of the cytoskeleton [105] is up-

regulated at 1 hpi. Filamins and other components of the cytoskeleton also interact with signaling pathways that regulate the cytoskeleton and other cellular activities [105]. The phosphoinositide INPPL1 (inositol polyphosphate phosphatase-like 1; SHIP-2), forms complexes with FLNC that are localized to membrane ruffles [106]; *INPPL1* expression is up-regulated at 1hpi. INPPL1 may also participate in vesicle trafficking (see below). β-arrestin, which participates in clathrin-mediated endocytosis and actin reorganization [107] exhibits decreased expression at 1 hpi. Several other genes that are likely to regulate or alter cytoskeletal function have either increased (ankyrin-G; *ANK3*) or decreased (scinderin, *SCIN*) expression at 1hpi. The actin binding protein calponin 3 (*CNN3*) is up-regulated at both 1 and 24 hpi. Scinderin, an actin severing protein is down-regulated at 24 hpi as well.

Formins have a critical role in nucleation and catalysis of actin polymerization. Formins are found at the fast-growing ("barbed") end of the actin filament [108,109]. Formins also directly bind and regulate microtubule networks [110]. Chlamydial inclusions interact with microtubules through the chlamydial inclusion membrane proteins (Incs); this interaction enables inclusion trafficking to the microtubule-organizing center (MTOC) [111,112]. Formin 1 (*FMN1*) and formin 2 (*FMN2*) are both up-regulated at 1 hpi. *FMN1* is also up-regulated at 24 hpi. The microtubule-associated proteins *MAP1A* and *MAP1B* are also up-regulated at 1 hpi. Three members (*KIF21B*, *KIF27* and *KLA6*) of the kinesin superfamily of microtubule motor proteins, likely involved with intracellular vesicle transport [113,114], are down-regulated at 1hpi.

In addition to trafficking of the chlamydial inclusion to the MTOC, the inclusion also intercepts both vesicular and non-vesicular mediated pathways to obtain host-derived lipids (reviewed by[115]). Pathogens that reside in intracellular vacuoles often use subsets of host Rab GTPases to form and maintain these membrane-bound spaces [116]. Rabs are regulated by antagonizing enzymes that convert inactivating GDP-bound Rabs to the active GTP-bound forms. Chlamydiae target multiple Rab-dependent pathways at various times over the course of the developmental cycle, either by recruiting or excluding them from the inclusion; numerous Rabs decorate the inclusion and are likely to impact inclusion trafficking and avoidance of lysosomal fusion (reviewed by [98]). At 1hpi, we find differential expression of several Rab GTPases and related ADP-ribosylation-like factors (ARLs) that have not previously been linked to chlamydial infection. *RAB32* and *RAB3B* demonstrate increased expression, whereas *RAB17* expression is decreased. RAB32 is a critical component of the mitochondrial-associated membrane (MAM) of the smooth endoplasmic reticulum [117]. The MAM is a major cellular signaling hub that controls cellular metabolism and apoptosis via the controlled exchange of calcium between the ER and mitochondria [118]. RAB3B negatively regulates IgA transport by directly binding the polymeric

IgA receptor (pIgR) [119]. pIgR binds and transports dimeric IgA to the apical surfaces for processing into the IgA secretory component [119], which is a major defense molecule against mucosal pathogens. Thus increased expression of both *RAB32* and *RAB3B* has obvious consequences for putative chlamydial subversion of mitochondrial-mediated metabolism or apoptosis mechanisms, and mucosal immunity respectively. Similar to RAB3B, RAB17 is also involved with receptor-mediated transcytosis to apical membranes [120,121], and down-regulation of RAB17 has been linked to cancer cells exhibiting a more mesenchymal morphology [122]. Decreased expression of *RAB17* in chlamydial-infected cells may be part of an epithelial-to-mesenchymal transition [73] that leads to loss of adhesion and subsequent sloughing of infected cells, contributing to ascending infection.

The GTP-controlled ADP-ribosylation-like factors (ARLs), *ARL10* and *ARL15* are both strongly expressed at 1 hpi, whereas *ARL4D* shows decreased expression. In general, ARLs function in vesicle trafficking from the Golgi, regulation of membrane lipid composition, and interaction with cytoskeletal factors [123]. *ARL4D* is tightly associated with the mitochondrial inner membrane, where it causes mitochondrial fragmentation on activation [124]. Down-regulation of *ARL4D* may be part of the chlamydial anti-apoptotic strategy that confers apoptotic resistance to infected cells. An early chlamydial impact on mitochondrial function is further supported by decreased expression of numerous mitochondrial genes, including multiple components of the mitochondrial NADH dehydrogenase complex (MT-ND1, MT-ND2, MT-ND4, MT-ND4L, MT-ND5, MT-ND6), cytochrome b (MT-CYB), cytochrome c oxidase II, (MT-CO2), phosphoenolpyruvate carboxykinase 2 (PCK2) and ATP synthase 6 (MT-ATP6).

Together with Rabs and ARLs, phosphoinositides (PIs) mediate membrane trafficking, particularly for the spatiotemporally regulated endolysosomal pathway [125]. Specific combinations of Rabs and PIs tag endolysosomal vesicles and distinguish their direction and specificity [125]. Similar to Rab regulation by GTP/GDP interconversion, phosphoinositides are interconverted between inactive and active forms by competing phospho-regulatory phosphatases and kinases. One of these interconversion steps is catalyzed by PIP5K and INPP5 [126]. Phosphatidylinositol-4-phosphate 5-kinase (PIP5K1B) phosphorylates phosphatidylinositol-4-phosphate (PtdIns(4)P) to form PtdIns(4,5)P_2 (PIP_2); inositol polyphosphate-5-phosphatase (INPP5A) catalyzes the opposite phosphatase reaction of PIP_2 to PtdIns(4)P. The kinase *PIP5K1B* is down-regulated at 1hpi, whereas the phosphatase *INPP5A* is up-regulated, indicating a putative enrichment of PtdIns(4)P and a concomitant decrease of PIP_2. These phosphoinositide moieties have previously been associated with regulation of early endosomal trafficking [126]; PtdIns(4)P is also typically localized to the Golgi [127]. PtdIns(4)P is enriched on the chlamydial inclusion membrane

[128]. This inclusion localization, combined with the differential expression of PIP5K1B and INPP5 observed here, firstly supports the concept that an altered PI profile on the chlamydial inclusion membrane may contribute to lysosomal avoidance by conferring a Golgi-like identity to the inclusion [128], and secondly, indicates that this "identity shift" is occurring early in infection.

Several other genes encoding phosphoinositol-interacting proteins exhibit differential expression. Sorting nexins are cytoplasmic PI-binding proteins that play central roles in endosomal trafficking pathways, beginning at endocytosis [129]. Sorting nexin 10 (*SNX10*) and sorting nexin 18 (*SNX18*) are down-regulated and up-regulated respectively at 1 hpi. In addition to the PI binding domain, SNX18 also contains a BAR domain, which interacts with membrane lipids and functions as a curvature sensor [130,131]. Three genes encoding proteins with pleckstrin homology (PH) domains (*PLEKHA4*, *PLEKHA6* and *PLEKHA7*) are all down-regulated at 1 hpi. The PH domain confers a core fold that enables binding of various phosphatidylinositol phosphate isoforms [132]; down-regulation of these proteins is further support for putative chlamydial dysregulation or co-opting of PI signaling and endosomal trafficking.

Chlamydiae require host-derived lipids, particularly for growth and maintenance of the dynamic inclusion, thus chlamydial acquisition and exploitation of host sphingolipids, glycerophospholipids and cholesterol has been intensively studied (reviewed by [133]). Host cytosolic phospholipase A2 is activated by chlamydiae to deacylate host phospholipids, which are then reacylated using chlamydial branched chain fatty acids and incorporated into the inclusion membrane [134]. We observe differential expression of several host phospholipase genes at 1 hpi: phospholipase A2 group XVI (*PLA2G16*) is down-regulated; phospholipase A2 receptor (*PLA2R1*) is up-regulated. Two variants of phospholipase C are differentially expressed – *PLCE1* is up-regulated whereas *PLCG2* is down-regulated. Glycosylphosphatidylinositol-specific phospholipase D1 (*GPLD1*) is also down-regulated in infected cells. This variability of host phospholipase expression may reflect a varying chlamydial requirement for the lipid precursors generated by each, or may represent targeting of host lipid-mediated signaling pathways.

Transcriptional regulation. In the preceding sections, we noted dysregulation of several host-signaling pathways in early chlamydial infection. Consistent with these disruptions, we observe expression changes for thirty-four transcription factors. These are likely to have diverse and wide-ranging effects on the host cell and thus the invading pathogen. However, while this highlights the complexity of the cellular response to chlamydial infection, their function remains mostly opaque. Many are either poorly

characterized or have been examined with a specific phenotype in mind (for example, carcinogenesis) that are not immediately applicable to infection processes. Several are known to be controlled by some of the dysregulated signaling pathways noted earlier, for example *TCF4*, *RUNX3*, *TLE2*, *LBH*, and *TBX3* are all Wnt-responsive [135-138]. The transcription factor *TGFB1I1* (*Hic-5*), which is up-regulated at 1 hpi, functions as a molecular adapter coordinating multiple protein-protein interactions at the focal adhesion complex and in the nucleus. It links intracellular signaling with plasma membrane receptors, and plays a role in the regulation of Wnt and TGFβ signaling [139-141].

The reversible epithelial-to-mesenchymal transition (EMT), which is the process of induced dedifferentiation of epithelial cells to cells with a mesenchymal phenotype [142], may play a role in chlamydial disease outcomes, as outlined above and by others [68,73]. EMT may contribute to chlamydial dissemination and ascending infection by enabling detachment of infected cells from the ECM. However, physical detachment of epithelial cells from their matrix may induce anoikis, a specialized apoptotic response that is normally induced in detached cells to prevent metastasis. Inhibition of anoikis is a key step in tumorigenesis [143], and may be required for ascending chlamydial infection. We find several differentially expressed transcription factors that regulate anoikis in other systems. In an oncogenic context, the pro-apoptotic Maged1 (NRAGE) transcription factor interacts with a cytoplasmic component of the E-cadherin complex at the adherans junction, ankyrin-G, which binds and sequesters NRAGE to the cytoplasm [144]. EMT processes cause decreased expression of ankyrin-G, which in turn enables nuclear localization of NRAGE. The repressive activity of nuclear NRAGE on a variety of tumor suppressor genes then confers anoikis resistance [144]. In the infection context at 1 hpi, we find that ankyrin-G is up-regulated, while Maged1/RAGE is down-regulated. Thus at this early time prior to epithelial detachment, chlamydial-infected cells are still anoikis-sensitive by this mechanism. Study of later times in the infection cycle may reveal if anoikis inhibition plays a role in ascending infection.

Evidence for epigenetic modification and non-coding RNA expression. Pathogen-mediated histone modification to amend host transcription is an emerging theme in bacterial pathogenesis [145], and has been identified in *Chlamydia*. The chlamydial effector, NUE, possesses histone methyltransferase activity; NUE translocates to the nucleus and methylates the histones H2B, H3 and H4, presumably altering host transcription [146]. In our data, several host genes encoding histones are differentially expressed in infected cells at 1 hpi, indicating that higher order chromatin composition might be altered by chlamydial infection. The linker histone *H1F0* is down-regulated compared to mock-infected cells, whereas the linker histones *HIST1H1B* and *HIST1H1D* are up-regulated. The core histones *HIST1H2AL* and *HIST1H3C* are also up-regulated. While RNA-Seq does not reveal covalent modifications of histones,

this observed differential expression of histones and putative chromatin reorganization supports chlamydial orchestration of higher order chromatin dynamics.

Transcriptomic analyses have revealed many non-coding RNAs (ncRNAs) in eukaryotic genomes. ncRNA overlap with exons, introns and intergenic regions and often exhibit complex expression patterns [147]. Once considered "noise", many ncRNAs are now known as key transcriptional, post-transcriptional and epigenetic modulators of eukaryotic gene expression programs [148,149]. Many of the DE host transcripts detected by RNA-Seq are predicted ncRNA moieties, including micro-RNAs (miRNA), long non-coding RNAs (lncRNA), small nucleolar RNAs (snoRNA), antisense RNAs and apparent pseudogenes. Most of these ncRNAs are uncharacterized and may represent novel biological functionality relevant to chlamydial infection. For example, the miRNA miR24-2 is down-regulated at 1 hpi. miR24-2 is known to modulate several apoptotic pathways; over-expression of miR24-2 will induce apoptosis by down-regulation of the histone variant *H2AFX*, as well as BCL-2, MDM2 and P21 [150]. Down-regulation of the pro-apoptotic miR24-2 may be linked to chlamydial modulation of apoptosis.

We find down-regulation of an lncRNA within the *MAL2* gene at both 1 and 24 hpi, suggesting regulation of *MAL2* transcription is altered by chlamydial infection. MAL2 is an integral membrane protein found in endosome vesicles being transported via transcytosis [151]. MAL2 interacts with the Rho GTPase Cdc42 [152] and is thus a key element in endosome trafficking and lumen formation. Other cytoskeletal components that also interact with Cdc42 were identified above - both the Rho GTPase *ARHGAP31* and the guanine nucleotide exchange factor *FGD1* are differentially expressed; both are activators of Cdc42. Down-regulation of a MAL2 lincRNA lends further weight to the concept that dysregulation of factors that govern endosome dynamics is central to the avoidance of phagolysomal fusion by the chlamydial inclusion.

Curiously, we find increased expression of two lncRNAs that are well known imprinted alleles under epigenetic control. Both *MEG3* and *H19* lncRNAs exhibit increased expression at both 1 and 24 hpi. The differentially methylated *MEG3* (maternally expressed gene 3) is found within the human DLK1-DIO3 genomic region, which also includes one of the largest miRNA clusters in the human genome [153,154]. The function of *MEG3* is not understood but is thought to be involved in fundamental cell biology processes, including signaling, proliferation [155] and phagocytosis [156]. Deregulation of *MEG3* has been reported in several tumors and it has been proposed to be a tumor suppressor [155]. It is not clear what role *MEG3* plays in the context of chlamydial infection, however, increased expression will induce apoptosis [155], suggesting it may be participating in a cellular response to infection. Increased

expression of the imprinted *H19* lncRNA is similarly enigmatic. *H19* is a highly transcribed differentially methylated lncRNA that is silenced in the methylated paternal copy and expressed in the hypomethylated maternal copy [157]; several miRNAs with biological activity are also encoded within *H19* [158]. *H19* expression is tightly coupled to, and alternately regulated with, insulin growth factor 2 (IGF2) [157]. Its physiological role is unknown but may act as a tumor suppressor or as a regulator of a network of imprinted genes, as it is deregulated in many cancers and fetal growth syndromes in humans [157,158]. As with *MEG3*, it is not yet apparent what role is served by increased expression of *H19* in *Chlamydia*-infected cells. Nevertheless, it is evident that epigenetic and imprinting processes are relevant to chlamydial infection.

REFERENCES

1. Hegemann JH, Moelleken K (2012) Chlamydial Adhesion and Adhesins. In: Bavoil PM, Tan M, editors. Intracellular Pathogens I: Chlamydiales. Washington DC: ASM Press. pp. 97-125.
2. Hybiske K, Stephens RS (2007) Mechanisms of *Chlamydia trachomatis* entry into nonphagocytic cells. Infect Immun 75: 3925-3934.
3. Zhang JP, Stephens RS (1992) Mechanism of *C. trachomatis* attachment to eukaryotic host cells. Cell 69: 861-869.
4. Rosmarin DM, Carette JE, Olive AJ, Starnbach MN, Brummelkamp TR, et al. (2012) Attachment of *Chlamydia trachomatis* L2 to host cells requires sulfation. Proc Natl Acad Sci U S A 109: 10059-10064.
5. Darville T, O'Connell CM (2012) *Chlamydia* immunopathogenesis. In: Bavoil PM, Tan M, editors. Intracellular Pathogens I: Chlamydiales. Washington DC: ASM Press. pp. 241-264.
6. Darville T, Hiltke TJ (2010) Pathogenesis of genital tract disease due to *Chlamydia trachomatis*. J Infect Dis 201 Suppl 2: S114-125.
7. Nagarajan UM (2012) Immune Recognition and Host Cell Response during *Chlamydia* infection. In: Bavoil PM, Tan M, editors. Intracellular Pathogens I: Chlamydiales. Washington DC: ASM Press. pp. 217-239.
8. Rank RG, Whittimore J, Bowlin AK, Wyrick PB (2011) *In vivo* ultrastructural analysis of the intimate relationship between polymorphonuclear leukocytes and the chlamydial developmental cycle. Infect Immun 79: 3291-3301.
9. Stephens RS (2003) The cellular paradigm of chlamydial pathogenesis. Trends Microbiol 11: 44-51.
10. Rasmussen SJ, Eckmann L, Quayle AJ, Shen L, Zhang YX, et al. (1997) Secretion of proinflammatory cytokines by epithelial cells in response to *Chlamydia* infection suggests a central role for epithelial cells in chlamydial pathogenesis. J Clin Invest 99: 77-87.
11. Rose MC, Voynow JA (2006) Respiratory tract mucin genes and mucin glycoproteins in health and disease. Physiol Rev 86: 245-278.
12. Perez BH, Gipson IK (2008) Focus on Molecules: human mucin MUC16. Exp Eye Res 87: 400-401.
13. Patankar MS, Jing Y, Morrison JC, Belisle JA, Lattanzio FA, et al. (2005) Potent suppression of natural killer cell response mediated by the ovarian tumor marker CA125. Gynecol Oncol 99: 704-713.
14. Hollingsworth MA, Swanson BJ (2004) Mucins in cancer: protection and control of the cell surface. Nat Rev Cancer 4: 45-60.
15. Tarang S, Kumar S, Batra SK (2012) Mucins and toll-like receptors: kith and kin in infection and cancer. Cancer Lett 321: 110-119.

16. Midwood K, Sacre S, Piccinini AM, Inglis J, Trebaul A, et al. (2009) Tenascin-C is an endogenous activator of Toll-like receptor 4 that is essential for maintaining inflammation in arthritic joint disease. Nat Med 15: 774-780.
17. Midwood KS, Hussenet T, Langlois B, Orend G (2011) Advances in tenascin-C biology. Cell Mol Life Sci 68: 3175-3199.
18. Nace G, Evankovich J, Eid R, Tsung A (2012) Dendritic cells and damage-associated molecular patterns: endogenous danger signals linking innate and adaptive immunity. J Innate Immun 4: 6-15.
19. Newton K, Dixit VM (2012) Signaling in innate immunity and inflammation. Cold Spring Harb Perspect Biol 4.
20. Moreth K, Iozzo RV, Schaefer L (2012) Small leucine-rich proteoglycans orchestrate receptor crosstalk during inflammation. Cell Cycle 11: 2084-2091.
21. Merline R, Moreth K, Beckmann J, Nastase MV, Zeng-Brouwers J, et al. (2011) Signaling by the matrix proteoglycan decorin controls inflammation and cancer through PDCD4 and MicroRNA-21. Sci Signal 4: ra75.
22. Pasupuleti M, Schmidtchen A, Malmsten M (2012) Antimicrobial peptides: key components of the innate immune system. Crit Rev Biotechnol 32: 143-171.
23. Flodgaard H, Ostergaard E, Bayne S, Svendsen A, Thomsen J, et al. (1991) Covalent structure of two novel neutrophile leucocyte-derived proteins of porcine and human origin. Neutrophile elastase homologues with strong monocyte and fibroblast chemotactic activities. Eur J Biochem 197: 535-547.
24. Gabay JE, Scott RW, Campanelli D, Griffith J, Wilde C, et al. (1989) Antibiotic proteins of human polymorphonuclear leukocytes. Proc Natl Acad Sci U S A 86: 5610-5614.
25. Shafer WM, Martin LE, Spitznagel JK (1984) Cationic antimicrobial proteins isolated from human neutrophil granulocytes in the presence of diisopropyl fluorophosphate. Infect Immun 45: 29-35.
26. Soehnlein O, Lindbom L (2009) Neutrophil-derived azurocidin alarms the immune system. J Leukoc Biol 85: 344-351.
27. Kolodkin AL, Matthes DJ, Goodman CS (1993) The semaphorin genes encode a family of transmembrane and secreted growth cone guidance molecules. Cell 75: 1389-1399.
28. Takamatsu H, Kumanogoh A (2012) Diverse roles for semaphorin-plexin signaling in the immune system. Trends Immunol 33: 127-135.
29. Tamagnone L (2012) Emerging role of semaphorins as major regulatory signals and potential therapeutic targets in cancer. Cancer Cell 22: 145-152.
30. Wen H, Lei Y, Eun SY, Ting JP (2010) Plexin-A4-semaphorin 3A signaling is required for Toll-like receptor- and sepsis-induced cytokine storm. J Exp Med 207: 2943-2957.
31. Heuer D, Rejman Lipinski A, Machuy N, Karlas A, Wehrens A, et al. (2009) *Chlamydia* causes fragmentation of the Golgi compartment to ensure reproduction. Nature 457: 731-735.
32. Kokes M, Valdivia RH (2012) Cell Biology of the Chlamydial Inclusion. In: Bavoil PM, Tan M, editors. Intracellular Pathogens I: Chlamydiales. Washington DC: ASM Press. pp. 170-191.
33. Gault CR, Obeid LM, Hannun YA (2010) An overview of sphingolipid metabolism: from synthesis to breakdown. Adv Exp Med Biol 688: 1-23.
34. Perry RJ, Ridgway ND (2005) Molecular mechanisms and regulation of ceramide transport. Biochim Biophys Acta 1734: 220-234.
35. Bode C, Graler MH (2012) Immune regulation by sphingosine 1-phosphate and its receptors. Arch Immunol Ther Exp (Warsz) 60: 3-12.
36. Maceyka M, Harikumar KB, Milstien S, Spiegel S (2012) Sphingosine-1-phosphate signaling and its role in disease. Trends Cell Biol 22: 50-60.
37. Pyne NJ, Tonelli F, Lim KG, Long JS, Edwards J, et al. (2012) Sphingosine 1-phosphate signalling in cancer. Biochem Soc Trans 40: 94-100.
38. Rivera J, Proia RL, Olivera A (2008) The alliance of sphingosine-1-phosphate and its receptors in immunity. Nat Rev Immunol 8: 753-763.

39. Dorsam G, Graeler MH, Seroogy C, Kong Y, Voice JK, et al. (2003) Transduction of multiple effects of sphingosine 1-phosphate (S1P) on T cell functions by the S1P1 G protein-coupled receptor. J Immunol 171: 3500-3507.
40. Matloubian M, Lo CG, Cinamon G, Lesneski MJ, Xu Y, et al. (2004) Lymphocyte egress from thymus and peripheral lymphoid organs is dependent on S1P receptor 1. Nature 427: 355-360.
41. Janeway CA, Jr. (1989) Approaching the asymptote? Evolution and revolution in immunology. Cold Spring Harb Symp Quant Biol 54 Pt 1: 1-13.
42. Su J, Zhang T, Tyson J, Li L (2009) The interleukin-1 receptor-associated kinase M selectively inhibits the alternative, instead of the classical NFkappaB pathway. J Innate Immun 1: 164-174.
43. Huang J, Teng L, Liu T, Li L, Chen D, et al. (2003) Identification of a novel serine/threonine kinase that inhibits TNF-induced NF-kappaB activation and p53-induced transcription. Biochem Biophys Res Commun 309: 774-778.
44. Wu M, Xu LG, Zhai Z, Shu HB (2003) SINK is a p65-interacting negative regulator of NF-kappaB-dependent transcription. J Biol Chem 278: 27072-27079.
45. Hegedus Z, Czibula A, Kiss-Toth E (2006) Tribbles: novel regulators of cell function; evolutionary aspects. Cell Mol Life Sci 63: 1632-1641.
46. Smith SM, Moran AP, Duggan SP, Ahmed SE, Mohamed AS, et al. (2011) Tribbles 3: a novel regulator of TLR2-mediated signaling in response to *Helicobacter pylori* lipopolysaccharide. J Immunol 186: 2462-2471.
47. Moynagh PN (2009) The Pellino family: IRAK E3 ligases with emerging roles in innate immune signalling. Trends Immunol 30: 33-42.
48. Hatada EN, Krappmann D, Scheidereit C (2000) NF-kappaB and the innate immune response. Curr Opin Immunol 12: 52-58.
49. Siegrist F, Ebeling M, Certa U (2011) The small interferon-induced transmembrane genes and proteins. J Interferon Cytokine Res 31: 183-197.
50. Magalhaes JG, Sorbara MT, Girardin SE, Philpott DJ (2011) What is new with Nods? Curr Opin Immunol 23: 29-34.
51. Loo YM, Gale M, Jr. (2011) Immune signaling by RIG-I-like receptors. Immunity 34: 680-692.
52. Oshiumi H, Matsumoto M, Seya T (2012) Ubiquitin-mediated modulation of the cytoplasmic viral RNA sensor RIG-I. J Biochem 151: 5-11.
53. Sharma M, Rudel T (2009) Apoptosis resistance in *Chlamydia*-infected cells: a fate worse than death? FEMS Immunol Med Microbiol 55: 154-161.
54. Schlessinger J (2004) Common and distinct elements in cellular signaling via EGF and FGF receptors. Science 306: 1506-1507.
55. Yarden Y, Sliwkowski MX (2001) Untangling the ErbB signalling network. Nat Rev Mol Cell Biol 2: 127-137.
56. Scheller J, Chalaris A, Garbers C, Rose-John S (2011) ADAM17: a molecular switch to control inflammation and tissue regeneration. Trends Immunol 32: 380-387.
57. Malmsten M, Davoudi M, Walse B, Rydengard V, Pasupuleti M, et al. (2007) Antimicrobial peptides derived from growth factors. Growth Factors 25: 60-70.
58. Pedron T, Thibault C, Sansonetti PJ (2003) The invasive phenotype of *Shigella flexneri* directs a distinct gene expression pattern in the human intestinal epithelial cell line Caco-2. J Biol Chem 278: 33878-33886.
59. Kim Y, Oh S, Park S, Kim SH (2009) Interactive transcriptome analysis of enterohemorrhagic *Escherichia coli* (EHEC) O157:H7 and intestinal epithelial HT-29 cells after bacterial attachment. Int J Food Microbiol 131: 224-232.
60. Tuccillo C, Manzo BA, Nardone G, D'Argenio G, Rocco A, et al. (2002) Up-regulation of heparin binding epidermal growth factor-like growth factor and amphiregulin expression in Helicobacter pylori-infected human gastric mucosa. Dig Liver Dis 34: 498-505.
61. Lofmark S, de Klerk N, Aro H (2011) *Neisseria gonorrhoeae* infection induces altered amphiregulin processing and release. PLoS One 6: e16369.

62. Fernandez-Fernandez L, Bellido-Martin L, Garcia de Frutos P (2008) Growth arrest-specific gene 6 (GAS6). An outline of its role in haemostasis and inflammation. Thromb Haemost 100: 604-610.
63. Rothlin CV, Lemke G (2010) TAM receptor signaling and autoimmune disease. Curr Opin Immunol 22: 740-746.
64. Korshunov VA (2012) Axl-dependent signalling: a clinical update. Clin Sci (Lond) 122: 361-368.
65. Verbeke P, Welter-Stahl L, Ying S, Hansen J, Hacker G, et al. (2006) Recruitment of BAD by the *Chlamydia trachomatis* vacuole correlates with host-cell survival. PLoS Pathog 2: e45.
66. Deng T, Zhang Y, Chen Q, Yan K, Han D (2012) Toll-like receptor-mediated inhibition of Gas6 and ProS expression facilitates inflammatory cytokine production in mouse macrophages. Immunology 135: 40-50.
67. Thiery JP, Acloque H, Huang RY, Nieto MA (2009) Epithelial-mesenchymal transitions in development and disease. Cell 139: 871-890.
68. Kessler M, Zielecki J, Thieck O, Mollenkopf HJ, Fotopoulou C, et al. (2012) *Chlamydia trachomatis* disturbs epithelial tissue homeostasis in fallopian tubes via paracrine Wnt signaling. Am J Pathol 180: 186-198.
69. Moulder JW, Hatch TP, Byrne GI, Kellogg KR (1976) Immediate toxicity of high multiplicities of *Chlamydia psittaci* for mouse fibroblasts (L cells). Infect Immun 14: 277-289.
70. Doughri AM, Altera KP, Storz J, Eugster AK (1973) Ultrastructural changes in the chlamydia-infected ileal mucosa of newborn calves. Vet Pathol 10: 114-123.
71. Doughri AM, Storz J, Altera KP (1972) Mode of entry and release of chlamydiae in infections of intestinal epithelial cells. J Infect Dis 126: 652-657.
72. Soloff BL, Rank RG, Barron AL (1985) Electron microscopic observations concerning the in vivo uptake and release of the agent of guinea-pig inclusion conjunctivitis (Chlamydia psittaci) in guinea-pig exocervix. J Comp Pathol 95: 335-344.
73. Ramsey KH (2006) Alternative Mechanisms of Pathogenesis. In: Bavoil PM, Wyrick PB, editors. Chlamydia: Genomics and Pathogenesis. Norfolk UK: Horizon Bioscience. pp. 435-473.
74. Shen L (2012) Tight junctions on the move: molecular mechanisms for epithelial barrier regulation. Ann N Y Acad Sci 1258: 9-18.
75. Kar R, Batra N, Riquelme MA, Jiang JX (2012) Biological role of connexin intercellular channels and hemichannels. Arch Biochem Biophys 524: 2-15.
76. Brasch J, Harrison OJ, Honig B, Shapiro L (2012) Thinking outside the cell: how cadherins drive adhesion. Trends Cell Biol 22: 299-310.
77. Morishita H, Yagi T (2007) Protocadherin family: diversity, structure, and function. Curr Opin Cell Biol 19: 584-592.
78. Lo SH (2004) Tensin. Int J Biochem Cell Biol 36: 31-34.
79. Valenta T, Hausmann G, Basler K (2012) The many faces and functions of beta-catenin. EMBO J 31: 2714-2736.
80. Clevers H, Nusse R (2012) Wnt/beta-catenin signaling and disease. Cell 149: 1192-1205.
81. Schulte G, Bryja V (2007) The Frizzled family of unconventional G-protein-coupled receptors. Trends Pharmacol Sci 28: 518-525.
82. Angers S, Moon RT (2009) Proximal events in Wnt signal transduction. Nat Rev Mol Cell Biol 10: 468-477.
83. Chen H, Liu L, Ma B, Ma TM, Hou JJ, et al. (2011) Protein kinase A-mediated 14-3-3 association impedes human Dapper1 to promote dishevelled degradation. J Biol Chem 286: 14870-14880.
84. Gao X, Wen J, Zhang L, Li X, Ning Y, et al. (2008) Dapper1 is a nucleocytoplasmic shuttling protein that negatively modulates Wnt signaling in the nucleus. J Biol Chem 283: 35679-35688.
85. Niehrs C (2006) Function and biological roles of the Dickkopf family of Wnt modulators. Oncogene 25: 7469-7481.
86. Ito K, Lim AC, Salto-Tellez M, Motoda L, Osato M, et al. (2008) RUNX3 attenuates beta-catenin/T cell factors in intestinal tumorigenesis. Cancer Cell 14: 226-237.

87. Astrup E, Lekva T, Davi G, Otterdal K, Santilli F, et al. (2012) A Complex Interaction between Rickettsia conorii and Dickkopf-1 - Potential Role in Immune Evasion Mechanisms in Endothelial Cells. PLoS One 7: e43638.
88. Barczyk M, Carracedo S, Gullberg D (2010) Integrins. Cell Tissue Res 339: 269-280.
89. Margadant C, Monsuur HN, Norman JC, Sonnenberg A (2011) Mechanisms of integrin activation and trafficking. Curr Opin Cell Biol 23: 607-614.
90. Kelly KA, Wiley D, Wiesmeier E, Briskin M, Butch A, et al. (2009) The combination of the gastrointestinal integrin (alpha4beta7) and selectin ligand enhances T-Cell migration to the reproductive tract during infection with *Chlamydia trachomatis*. Am J Reprod Immunol 61: 446-452.
91. Eiraku M, Hirata Y, Takeshima H, Hirano T, Kengaku M (2002) Delta/notch-like epidermal growth factor (EGF)-related receptor, a novel EGF-like repeat-containing protein targeted to dendrites of developing and adult central nervous system neurons. J Biol Chem 277: 25400-25407.
92. Groot AJ, Vooijs MA (2012) The role of Adams in Notch signaling. Adv Exp Med Biol 727: 15-36.
93. Andersen P, Uosaki H, Shenje LT, Kwon C (2012) Non-canonical Notch signaling: emerging role and mechanism. Trends Cell Biol 22: 257-265.
94. Ito T, Connett JM, Kunkel SL, Matsukawa A (2012) Notch system in the linkage of innate and adaptive immunity. J Leukoc Biol 92: 59-65.
95. Louvi A, Artavanis-Tsakonas S (2012) Notch and disease: a growing field. Semin Cell Dev Biol 23: 473-480.
96. Zhang Q, Wang C, Liu Z, Liu X, Han C, et al. (2012) Notch signal suppresses Toll-like receptor-triggered inflammatory responses in macrophages by inhibiting extracellular signal-regulated kinase 1/2-mediated nuclear factor kappaB activation. J Biol Chem 287: 6208-6217.
97. Dunn JD, Valdivia RH (2010) Uncivil engineers: *Chlamydia*, *Salmonella* and *Shigella* alter cytoskeleton architecture to invade epithelial cells. Future Microbiol 5: 1219-1232.
98. Scidmore MA (2011) Recent advances in *Chlamydia* subversion of host cytoskeletal and membrane trafficking pathways. Microbes Infect 13: 527-535.
99. Kumar Y, Valdivia RH (2008) Actin and intermediate filaments stabilize the *Chlamydia trachomatis* vacuole by forming dynamic structural scaffolds. Cell Host Microbe 4: 159-169.
100. Olson MF, Pasteris NG, Gorski JL, Hall A (1996) Faciogenital dysplasia protein (FGD1) and Vav, two related proteins required for normal embryonic development, are upstream regulators of Rho GTPases. Curr Biol 6: 1628-1633.
101. Southgate L, Machado RD, Snape KM, Primeau M, Dafou D, et al. (2011) Gain-of-function mutations of ARHGAP31, a Cdc42/Rac1 GTPase regulator, cause syndromic cutis aplasia and limb anomalies. Am J Hum Genet 88: 574-585.
102. Subtil A, Wyplosz B, Balana ME, Dautry-Varsat A (2004) Analysis of *Chlamydia caviae* entry sites and involvement of Cdc42 and Rac activity. J Cell Sci 117: 3923-3933.
103. Gasman S, Kalaidzidis Y, Zerial M (2003) RhoD regulates endosome dynamics through Diaphanous-related Formin and Src tyrosine kinase. Nat Cell Biol 5: 195-204.
104. Campelo F, Malhotra V (2012) Membrane fission: the biogenesis of transport carriers. Annu Rev Biochem 81: 407-427.
105. Nakamura F, Stossel TP, Hartwig JH (2011) The filamins: organizers of cell structure and function. Cell Adh Migr 5: 160-169.
106. Dyson JM, O'Malley CJ, Becanovic J, Munday AD, Berndt MC, et al. (2001) The SH2-containing inositol polyphosphate 5-phosphatase, SHIP-2, binds filamin and regulates submembraneous actin. J Cell Biol 155: 1065-1079.
107. Min J, Defea K (2011) beta-arrestin-dependent actin reorganization: bringing the right players together at the leading edge. Mol Pharmacol 80: 760-768.
108. Chesarone MA, DuPage AG, Goode BL (2010) Unleashing formins to remodel the actin and microtubule cytoskeletons. Nat Rev Mol Cell Biol 11: 62-74.

109. Firat-Karalar EN, Welch MD (2011) New mechanisms and functions of actin nucleation. Curr Opin Cell Biol 23: 4-13.
110. Bartolini F, Gundersen GG (2010) Formins and microtubules. Biochim Biophys Acta 1803: 164-173.
111. Grieshaber SS, Grieshaber NA, Hackstadt T (2003) *Chlamydia trachomatis* uses host cell dynein to traffic to the microtubule-organizing center in a p50 dynamitin-independent process. J Cell Sci 116: 3793-3802.
112. Mital J, Miller NJ, Fischer ER, Hackstadt T (2010) Specific chlamydial inclusion membrane proteins associate with active Src family kinases in microdomains that interact with the host microtubule network. Cell Microbiol 12: 1235-1249.
113. Hehnly H, Stamnes M (2007) Regulating cytoskeleton-based vesicle motility. FEBS Lett 581: 2112-2118.
114. Horgan CP, McCaffrey MW (2011) Rab GTPases and microtubule motors. Biochem Soc Trans 39: 1202-1206.
115. Saka HA, Valdivia RH (2010) Acquisition of nutrients by Chlamydiae: unique challenges of living in an intracellular compartment. Curr Opin Microbiol 13: 4-10.
116. Seabra MC, Mules EH, Hume AN (2002) Rab GTPases, intracellular traffic and disease. Trends Mol Med 8: 23-30.
117. Bui M, Gilady SY, Fitzsimmons RE, Benson MD, Lynes EM, et al. (2010) Rab32 modulates apoptosis onset and mitochondria-associated membrane (MAM) properties. J Biol Chem 285: 31590-31602.
118. Lynes EM, Simmen T (2011) Urban planning of the endoplasmic reticulum (ER): how diverse mechanisms segregate the many functions of the ER. Biochim Biophys Acta 1813: 1893-1905.
119. Smythe E (2002) Direct interactions between rab GTPases and cargo. Mol Cell 9: 205-206.
120. Hunziker W, Peters PJ (1998) Rab17 localizes to recycling endosomes and regulates receptor-mediated transcytosis in epithelial cells. J Biol Chem 273: 15734-15741.
121. Zacchi P, Stenmark H, Parton RG, Orioli D, Lim F, et al. (1998) Rab17 regulates membrane trafficking through apical recycling endosomes in polarized epithelial cells. J Cell Biol 140: 1039-1053.
122. Lutcke A, Jansson S, Parton RG, Chavrier P, Valencia A, et al. (1993) Rab17, a novel small GTPase, is specific for epithelial cells and is induced during cell polarization. J Cell Biol 121: 553-564.
123. Donaldson JG, Jackson CL (2011) ARF family G proteins and their regulators: roles in membrane transport, development and disease. Nat Rev Mol Cell Biol 12: 362-375.
124. Li CC, Wu TS, Huang CF, Jang LT, Liu YT, et al. (2012) GTP-binding-defective ARL4D alters mitochondrial morphology and membrane potential. PLoS One 7: e43552.
125. Jean S, Kiger AA (2012) Coordination between RAB GTPase and phosphoinositide regulation and functions. Nat Rev Mol Cell Biol 13: 463-470.
126. Kanaho Y, Kobayashi-Nakano A, Yokozeki T (2007) The phosphoinositide kinase PIP5K that produces the versatile signaling phospholipid PI4,5P(2). Biol Pharm Bull 30: 1605-1609.
127. D'Angelo G, Vicinanza M, Di Campli A, De Matteis MA (2008) The multiple roles of PtdIns(4)P -- not just the precursor of PtdIns(4,5)P2. J Cell Sci 121: 1955-1963.
128. Moorhead AM, Jung JY, Smirnov A, Kaufer S, Scidmore MA (2010) Multiple host proteins that function in phosphatidylinositol-4-phosphate metabolism are recruited to the chlamydial inclusion. Infect Immun 78: 1990-2007.
129. Cullen PJ, Korswagen HC (2012) Sorting nexins provide diversity for retromer-dependent trafficking events. Nat Cell Biol 14: 29-37.
130. Haberg K, Lundmark R, Carlsson SR (2008) SNX18 is an SNX9 paralog that acts as a membrane tubulator in AP-1-positive endosomal trafficking. J Cell Sci 121: 1495-1505.
131. Wang JT, Kerr MC, Karunaratne S, Jeanes A, Yap AS, et al. (2010) The SNX-PX-BAR family in macropinocytosis: the regulation of macropinosome formation by SNX-PX-BAR proteins. PLoS One 5: e13763.

132. Scheffzek K, Welti S (2012) Pleckstrin homology (PH) like domains - versatile modules in protein-protein interaction platforms. FEBS Lett 586: 2662-2673.
133. Elwell CA, Engel JN (2012) Lipid acquisition by intracellular Chlamydiae. Cell Microbiol 14: 1010-1018.
134. Vignola MJ, Kashatus DF, Taylor GA, Counter CM, Valdivia RH (2010) cPLA2 regulates the expression of type I interferons and intracellular immunity to *Chlamydia trachomatis*. J Biol Chem 285: 21625-21635.
135. Archbold HC, Yang YX, Chen L, Cadigan KM (2012) How do they do Wnt they do?: regulation of transcription by the Wnt/beta-catenin pathway. Acta Physiol (Oxf) 204: 74-109.
136. Buechling T, Boutros M (2011) Wnt signaling signaling at and above the receptor level. Curr Top Dev Biol 97: 21-53.
137. Mosimann C, Hausmann G, Basler K (2009) Beta-catenin hits chromatin: regulation of Wnt target gene activation. Nat Rev Mol Cell Biol 10: 276-286.
138. Shitashige M, Hirohashi S, Yamada T (2008) Wnt signaling inside the nucleus. Cancer Sci 99: 631-637.
139. Li X, Martinez-Ferrer M, Botta V, Uwamariya C, Banerjee J, et al. (2011) Epithelial Hic-5/ARA55 expression contributes to prostate tumorigenesis and castrate responsiveness. Oncogene 30: 167-177.
140. Pignatelli J, Tumbarello DA, Schmidt RP, Turner CE (2012) Hic-5 promotes invadopodia formation and invasion during TGF-beta-induced epithelial-mesenchymal transition. J Cell Biol 197: 421-437.
141. Thomas SM, Hagel M, Turner CE (1999) Characterization of a focal adhesion protein, Hic-5, that shares extensive homology with paxillin. J Cell Sci 112 (Pt 2): 181-190.
142. Thiery JP (2002) Epithelial-mesenchymal transitions in tumour progression. Nat Rev Cancer 2: 442-454.
143. Tiwari N, Gheldof A, Tatari M, Christofori G (2012) EMT as the ultimate survival mechanism of cancer cells. Semin Cancer Biol 22: 194-207.
144. Kumar S, Park SH, Cieply B, Schupp J, Killiam E, et al. (2011) A pathway for the control of anoikis sensitivity by E-cadherin and epithelial-to-mesenchymal transition. Mol Cell Biol 31: 4036-4051.
145. Hamon MA, Cossart P (2008) Histone modifications and chromatin remodeling during bacterial infections. Cell Host Microbe 4: 100-109.
146. Pennini ME, Perrinet S, Dautry-Varsat A, Subtil A (2010) Histone methylation by NUE, a novel nuclear effector of the intracellular pathogen Chlamydia trachomatis. PLoS Pathog 6: e1000995.
147. Mattick JS (2009) The genetic signatures of noncoding RNAs. PLoS Genet 5: e1000459.
148. Taft RJ, Pang KC, Mercer TR, Dinger M, Mattick JS (2010) Non-coding RNAs: regulators of disease. J Pathol 220: 126-139.
149. O'Connell RM, Rao DS, Baltimore D (2012) microRNA Regulation of Inflammatory Responses. Annu Rev Immunol 30: 295-312.
150. Srivastava N, Manvati S, Srivastava A, Pal R, Kalaiarasan P, et al. (2011) miR-24-2 controls H2AFX expression regardless of gene copy number alteration and induces apoptosis by targeting antiapoptotic gene BCL-2: a potential for therapeutic intervention. Breast Cancer Res 13: R39.
151. Wilson SH, Bailey AM, Nourse CR, Mattei MG, Byrne JA (2001) Identification of MAL2, a novel member of the mal proteolipid family, though interactions with TPD52-like proteins in the yeast two-hybrid system. Genomics 76: 81-88.
152. Madrid R, Aranda JF, Rodriguez-Fraticelli AE, Ventimiglia L, Andres-Delgado L, et al. (2010) The formin INF2 regulates basolateral-to-apical transcytosis and lumen formation in association with Cdc42 and MAL2. Dev Cell 18: 814-827.
153. Benetatos L, Voulgaris E, Vartholomatos G (2012) DLK1-MEG3 imprinted domain microRNAs in cancer biology. Crit Rev Eukaryot Gene Expr 22: 1-15.
154. McMurray EN, Schmidt JV (2012) Identification of imprinting regulators at the Meg3 differentially methylated region. Genomics 100: 184-194.

155. Zhou Y, Zhang X, Klibanski A (2012) MEG3 noncoding RNA: a tumor suppressor. J Mol Endocrinol 48: R45-53.
156. Jeon H, Go Y, Seo M, Lee WH, Suk K (2010) Functional selection of phagocytosis-promoting genes: cell sorting-based selection. J Biomol Screen 15: 949-955.
157. Gabory A, Ripoche MA, Yoshimizu T, Dandolo L (2006) The H19 gene: regulation and function of a non-coding RNA. Cytogenet Genome Res 113: 188-193.
158. Keniry A, Oxley D, Monnier P, Kyba M, Dandolo L, et al. (2012) The H19 lincRNA is a developmental reservoir of miR-675 that suppresses growth and Igf1r. Nat Cell Biol 14: 659-665.
